# Modular synthesis of unsaturated aza-heterocycles via copper catalyzed multicomponent cascade reaction

**DOI:** 10.1016/j.isci.2023.106137

**Published:** 2023-02-09

**Authors:** Siqi Wei, Guocong Zhang, Yahui Wang, Mengwei You, Yanan Wang, Liejin Zhou, Zuxiao Zhang

**Affiliations:** 1 Key Laboratory of the Ministry of Education for Advanced Catalysis Materials, Department of Chemistry, Zhejiang Normal University, 688 Yingbin Road, Jinhua, China

**Keywords:** Chemical reaction, Catalysis, Chemical synthesis

## Abstract

The unsaturated aza-heterocycles such as tetrahydropyridines pose significant applications in both drug discovery and development. However, the methods to construct polyfunctionalized tetrahydropyridines are still limited. Herein, we report a modular synthesis of tetrahydropyridines via copper catalyzed multicomponent radical cascade reaction. The reaction features mild conditions and broad substrate scope. In addition, the reaction could scale up to gram scale with similar yield. A variety of 1,2,5,6-tetrahydropyridines with C3 and C5 substituents could be assembled from simple starting materials. More importantly, the products could serve as versatile intermediate to access various functionalized aza-heterocycles which further demonstrates its utility.

## Highlights

• A copper catalyzed multi-component radical cascade reaction is realized to access 1,2,5,6-tetrahydropyridines
• The reaction features with mild conditions and broad substrate scope
• A various functionalized unsaturated piperidines could be assembled from readily available starting materials
• Additionally, the products are versatile intermediates to access various functionalized aza-heterocycles which further demonstrates its utility

## Graphical abstract

**Figure:**
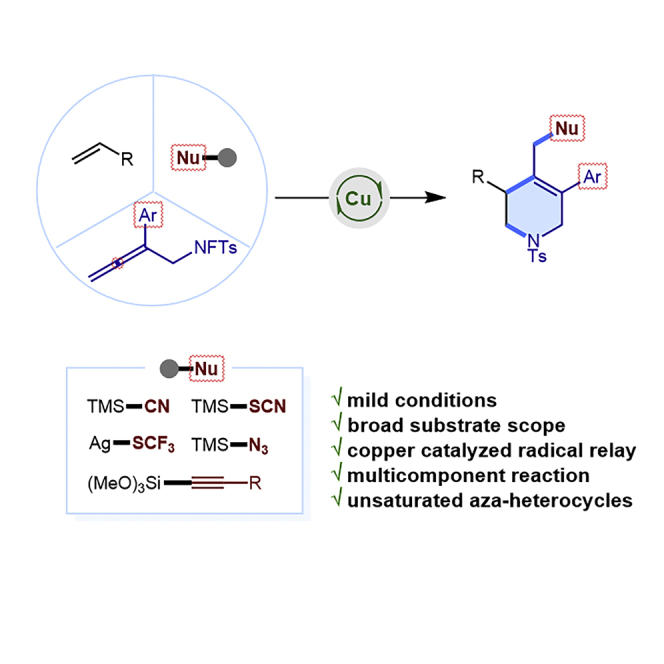

## Introduction

**Figure 1:**
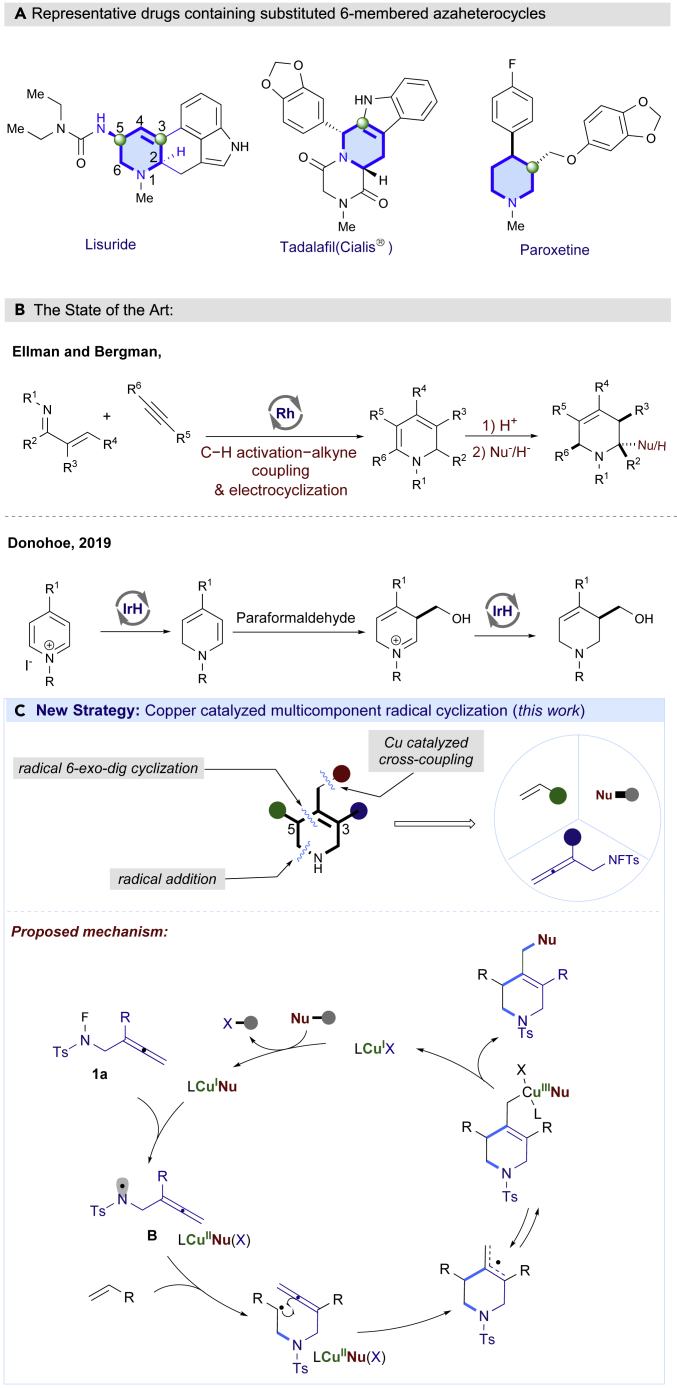
Background and methods to construct unsaturated Aza-heterocycles (A) Representative drugs containing substituted 6-membered azaheterocycles. (B) Or each panel or group of panels can be described separately The State-of-the-Art to construct unsaturated aza-heterocycles. (C) Rational of copper catalyzed multicomponent radical cyclization (this work).

Non-aromatic aza-heterocycles are widely existent in a majority of bioactive natural products and pharmaceutical compounds.^1^^,^^2^^,^^3^^,^^4^ These aza-heterocycles such as tetrahydropyridines and piperidines can be found in a number of FDA approved drugs such as Lisuride, Tadalafil, and Paroxetine (Figure 1A).^5^^,^^6^^,^^7^ Moreover, tetrahydropyridines are versatile intermediates to provide a wide range of functionalized piperidines via well-established olefin functionalization,^8^^,^^9^^,^^10^^,^^11^^,^^12^ which are the most prevalent nitrogen heterocycles found in pharmaceutical drugs.^13^^,^^14^^,^^15^^,^^16^^,^^17^ As a result, methods for the rapid assembly of diverse tetrahydropyridines from readily available starting materials are of high value to drug discovery and development.^18^^,^^19^ Albeit considerable effort has been devoted to developing efficient methods of constructing azaheterocycles, the synthesis of 1,2,5,6-tetrahydropyridines with C3 or C5 substituents continues to be a significant challenge. Traditional methods mainly relied on the partial reduction of pre-functionalized pyridines and further reduction of N-alkyl-1,2-dihydropyridines via iminium intermediates.^20^^,^^21^^,^^22^^,^^23^^,^^24^^,^^25^^,^^26^^,^^27^^,^^28^^,^^29^^,^^30^ Recently, Ellman and Bergman developed an elegant cascade strategy which involving Rh-catalyzed C−H activation of α,β-unsaturated imines followed by addition across alkynes, *in situ* electrocyclization, and reduction or nucleophilic addition in the presence of acid to obtain highly substituted 1,2,5,6-tetrahydropyridines.^31^^,^^32^^,^^33^^,^^34^ Alternatively, Donohoe et al. developed Iridium catalyzed reductive functionalization of pyridinium salts by harnessing the formation of a nucleophilic enamine intermediate to access C3 functionalized 1,2,5,6-tetrahydropyridines (Figure 1B)^35^ Notwithstanding these elegant reports, a strategy for the quick assembly of 1,2,5,6-tetrahydropyridines with C3 and C5 substituents is still highly desirable.

Multicomponent reactions represent one of the most efficient and practical synthetic methods in terms of atom- and step-economy for the expeditious construction of polyfunctionalized molecules with structural diversity from readily available starting materials.^36^^,^^37^^,^^38^^,^^39^^,^^40^^,^^41^ Thus, a multicomponent reaction approach would offer us an alternative solution to the modular synthesis of 1,2,5,6-tetrahydropyridines with various functionalities at C3, C4 and C5 positions. In the light of recent progress of copper catalyzed radical relay process which was initiated by generating N-centered radicals in C-H functionalization^42^^,^^43^^,^^44^^,^^45^^,^^46^^,^^47^^,^^48^^,^^49^^,^^50^^,^^51^^,^^52^^,^^53^^,^^54^^,^^55^^,^^56^^,^^57^^,^^58^^,^^59^^,^^60^^,^^61^^,^^62^^,^^63^^,^^64^^,^^65^^,^^66^^,^^67^^,^^68^^,^^69^ and difunctionalization of alkenes,^70^^,^^71^^,^^72^^,^^73^^,^^74^^,^^75^^,^^76^^,^^77^^,^^78^^,^^79^^,^^80^^,^^81^^,^^82^^,^^83^^,^^84^^,^^85^^,^^86^^,^^87^ we envisioned that a modular synthesis of valuable 1,2,5,6-tetrahydropyridines could be accomplished by a copper catalyzed radical cascade reaction involving three simple components (Figure 1C). Specifically, an F-masked 4-methyl-N-(2-phenylbuta-2,3-dien-1-yl)benzene-sulfonamide was designed to generate electrophilic N-centered radical via single electron transfer from copper catalyst. Followed by the N centered radical addition toward alkenes to trigger a radical 6-*exo*-dig cyclization. Finally, the formed allylic carbon radical reacted with a bulky Cu(II) complex to give the 1,2,5,6-tetrahydropyridines with various substituents at C3, C4, and C5 positions, which could provide a wealth of opportunities in both drug discovery and development (Figure 1C). Hence, we report a modular synthesis of functionalized tetrahydropyridines via copper catalyzed three components radical cascade reaction.

## Results and discussion

### Discovery

To test the feasibility of the proposed strategy, substrate **1a**, **1b** and TMSCN were as the model substrate. After a careful evaluation of various reaction parameters, the desired cyclization product **1c** was isolated in 65% yield with Cu(CH_3_CN)_4_PF_6_ as catalyst, L3 as the ligand, **1b** (2 equiv) as coupling partner, TMSCN (2.5 equiv) as nucleophile and fluorobenzene as solvent (see equation in Table 1, entry 1). As expected, the ligand played a pivotal role in this reaction. While using ligands L1 and L2, no desired product was formed. The BOX ligand (L3) was proved to be optimal to give good yield and excellent regioselectivity while installing the cyanide group at less steric hindered primary carbon site. In addition, in line with the proposed mechanism, the use of a chiral ligand did not impart any enantioselectivity and the product was found to be racemic. Other solvents instead of fluorobenzene were less effective and gave diminished yield (entries 2–6). Lots of copper salts could be employed as catalyst to furnish the desired product albeit with decreased yield (entries 7–10). Elevating the reaction temperature to 50°C made the reaction sluggish (entry 11). On the other hand, there was no reaction occurred at 0°C (entry 12). In addition, no matter increasing or decreasing the loading of TMSCN would diminish the yield of the desired product (entries 13–14). Gratifyingly, while using the alkene as limiting reagent, the reaction proceeded smoothly to give the desired product with comparable yield (entries 15–17).

**Table 1:** Optimization of copper catalyzed multicomponent cascade reaction^a^


Entry	Variation from standard conditions	**1c** Yield [%]
1	None	69 (65)^b^
2	DME instead of PhF	33
3	DCM instead of PhF	46
4	CH_3_CN instead of PhF	46
5	benzene instead of PhF	62
6	PhCF_3_ instead of PhF	55
7	CuOAc instead of Cu(CH_3_CN)_4_PF_6_	44
8	CuI instead of Cu(CH_3_CN)_4_PF_6_	39
9	CuSCN instead of Cu(CH_3_CN)_4_PF_6_	20
10	Cu(OTf)_2_ instead of Cu(CH_3_CN)_4_PF_6_	22
11	50°C instead of 36°C	40
12	0°C instead of 36°C	ND
13	TMSCN 1.5 equiv	48
14	TMSCN 3.0 equiv	51
15	**1a**:**1b** = 1:1	56
16	**1a**:**1b** = 1.2:1	58
17	**1a**:**1b** = 1.5:1	61

ND stands for No desired product. Please define any abbreviations used.

a General conditions: **1a** (0.1 mmol), **1b** (0.2 mmol), TMSCN (0.25 mmol), solvent (1.0 mL), 36°C, 12 h.

b Isolated yield.

### Synthetic evaluation

**Scheme 1:**
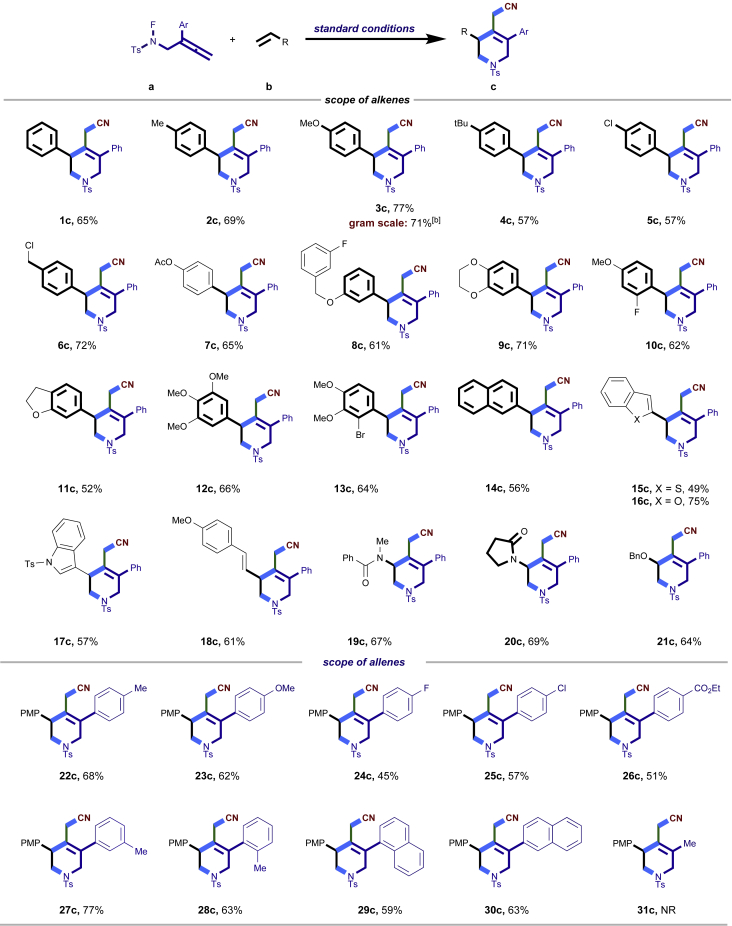
Scope of alkenes and allenes^a,b^ ^a^Reaction conditions: 1a (0.2 mmol), 1b (0.4 mmol), TMSCN (0.5 mmol), solvent (2.0 mL), 36°C, 12 h. Isolated yield. ^b^Gram-scale reaction (5 mmol).

After having established the optimal reaction conditions, we then investigated the generality of this copper catalyzed multicomponent radical cyclization reaction. To install different functional groups at C5 position of the 1,2,5,6-tetrahydropyridines, monosubstituted styrenes were first examined (Scheme 1), and the desired products (**1c**−**8c**) were obtained in moderate to good yields. Different substituents on the benzene ring, including the alkyl group (**2c**), halide (**5c**), alkyl halide group (**6c**)**,** and alkoxyl group (**3c**, **7c**, **8c**) were all tolerated. The substrates bearing two or three substituents on the aromatic ring reacted smoothly with **1a** to afford the desired products (**9c**-**13c**) in acceptable yields. An extended aromatic ring, such as 2- vinylnaphthalene, was a viable substrate and furnished the desired product **14c** in a moderate yield. Alkenes bearing heterocyclic moieties worked nicely in this reaction, gave the corresponding products (**15c**−**17c)** in 49–75% yield. Notably, heterocycles commonly existing in therapeutics, such as benzofuran, benzothiophene and indole, were well tolerated under the current conditions, which once again showcased the robustness of our method. To our delight, the radical cyclization took place regio-selectively and obtained **18c** in 61% yield when we commit our study with diene substrate. Furthermore, hetero atom substituents could be installed at the C5 position while using *N*-vinyl benzamide, N-vinly lactam and vinyl aryl ether as substrates, and the corresponding products **19c**–**21c** could be obtained in medium yield.

Next, a variety of F-masked sulfonamides were investigated to install different substituents on the C3 position of the 1,2,5,6-tetrahydropyridines (Scheme 1). A variety of functional groups including both electron donating groups and electron withdrawing groups on the para position of the aryl group were all compatible with the reaction conditions and furnished the corresponding products **22c**–**26c** in moderate yields. While changing the substituent to meta and otho position, the reaction went smoothly to give the desired products **27c**–**28c** in 77% and 63% yield respectively. The steric more hindered substituents such as naphthalene were tolerated as well **29c-30c**. Unfortunately, there was no reaction occurred while changing the aryl group into an alkyl group **31c**.

**Scheme 2:**
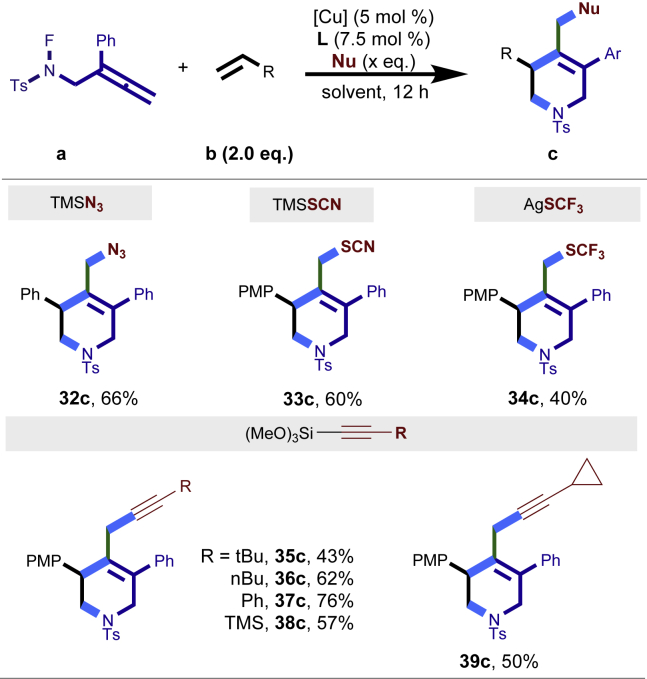
Scope of nucleophiles^a^ ^a^Detailed reaction conditions see supplemental information.

Encouraged by the above results that copper catalyst could trap the allylic carbon radical efficiently and regioselectively, we turned our attention to testing the coupling of allylic carbon radical with other nucleophiles (Scheme 2). TMSN_3_ and TMSSCN were used as nucleophiles in this reaction, affording the desired product **32c-33c** in medium yield. Considering the attractive properties of SCF_3_ group, AgSCF_3_ was then examined which could install SCF_3_ group **34c** under slightly modified conditions with acceptable yield as well. At last, a wide array of alkynylating reagents (alkynyltrimethoxysilane) with different substituents on the C−C triple bond, such as *t*Bu, nBu, phenyl, TMS, and cyclopropyl groups, were applied to this multicomponent radical cyclization reaction. As shown in Scheme 2, the reactions proceeded smoothly and yielded the corresponding alkynylation products (**35c**-**39c**) in moderate to good yields (43−76%).

### Synthetic utility

**Figure 2:**
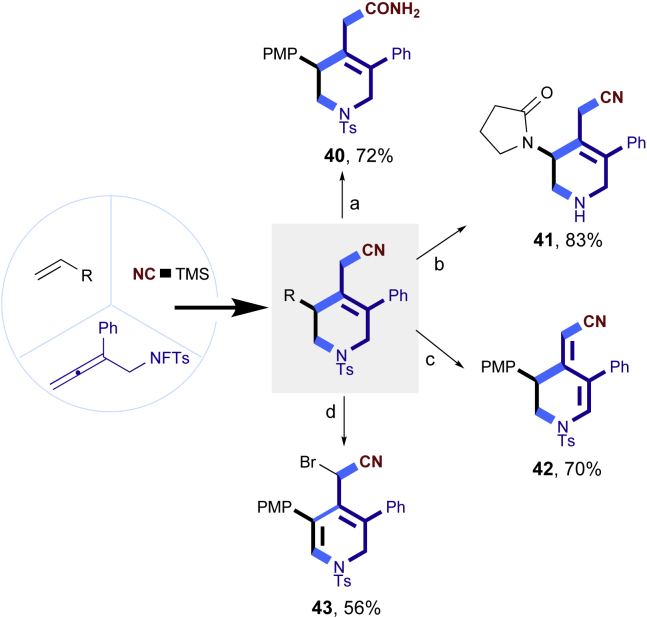
Transformations of the 1,2,5,6-tetrahydropyridines (A) 30% H2O2, NaOH (aq.), MeOH; (B) mg, MeOH; (C) DDQ, PhMe; (D) oxone, HBr, DCM.

To demonstrate the potential synthetic utility of our present method, further transformations of the tetrahydropyridines products were conducted (Figure 2). For example, the reaction of **3c** and H_2_O_2_ could produce the corresponding amide **40** compound in 72% yield. The Ts group on the nitrogen atom could be removed by Mg/MeOH to give **41** in 83% yield. The Compound **3c** could also undergo a dehydrogenation process by DDQ to form **42** in 70% yield. Finally, the **3c** treated with oxone and HBr led to an unexpected dihydropyridine **43** in 56% yield. The structure and configuration of **43** were determined by single-crystal X-ray diffraction analysis (see supplemental information).

### Conclusions

In summary, we have developed a copper catalyzed multicomponent radical cascade reaction. Various alkenes, F-masked allenes sufonylamides, and nucleophiles could be assembled to give important unsaturated nitrogen-containing heterocycles under mild conditions. The reaction involved an N-centered radical addition toward alkenes, 6-*exo*-dig cyclization, and regioselective cross coupling of the formed allylic radical. The transformations of the products and gram scale set up were further showcased the utility of this reaction.

### Limitations of the study

This work reports a highly efficient and regioselective method for the preparation of tetrahydropyridines via copper catalyzed multicomponent radical cascade reaction. Although a good substrate scope of alkenes and nucleophiles has been demonstrated, this method shows limitations on substrates of F-masked sulfonamides. Further optimization of catalysts and reaction conditions is needed to expand the scope of F-masked sulfonamides and improve the applicability of the method.

## STAR★Methods

### Key resources table

**Table:** 

REAGENT or RESOURCE	SOURCE	IDENTIFIER
**Chemical reagents and characterization instruments**		

Cu(CH_3_CN)_4_PF_6_	Energy Chemical	CAS: 64443-05-6
CuOAc	Energy Chemical	CAS: 598-54-9
CuI	Energy Chemical	CAS: 7681-65-4
CuSCN	Energy Chemical	CAS: 1111-67-7
Cu(OTf)_2_	Energy Chemical	CAS: 34946-82-2
TMSCN	bidepharm	CAS: 7677-24-9
TMSN_3_	Energy Chemical	CAS: 4648-54-8
AgSCF_3_	Energy Chemical	CAS: 811-68-7
Styrene	Energy Chemical	CAS: 100-42-5
4-Acetoxystyrene	Energy Chemical	CAS: 2628-16-2
4-Chlorostyrene	Energy Chemical	CAS: 1073-67-2
4-tert-Butylstyrene	Energy Chemical	CAS: 1746-23-2
4-Methylstyrene	Energy Chemical	CAS: 622-97-9
4-Methoxystyrene	Energy Chemical	CAS: 637-69-4
2-Vinylnaphthalene	Energy Chemical	CAS: 827-54-3
4-Vinylbenzyl chloride	Energy Chemical	CAS: 1592-20-7
DME	Energy Chemical	CAS: 110-71-4
DCM	Energy Chemical	CAS: 75-09-2
CH_3_CN	Energy Chemical	CAS: 75-05-8
Benzene	Energy Chemical	CAS: 71-43-2
PhCF_3_	Energy Chemical	CAS: 98-08-8
PhF	Energy Chemical	CAS: 462-06-6
2,2′-Bipyridine	Energy Chemical	CAS: 366-18-7
o-Phenanthroline	bidepharm	CAS: 66-71-7

**Deposited data**		

CIF of 3a	CCDC 2192306	https://www.ccdc.cam.ac.uk/structures/
CIF of 43	CCDC 2192304	https://www.ccdc.cam.ac.uk/structures/

**Other**		

Silica gel (200-300 mesh)	Shanxi Nuotai	
thin layer chromatography using TLC silica gel plates	Shanxi Nuotai	
AVIII 400 MHz	Bruker	https://bruker.com
AVIII 600 MHz	Bruker	https://bruker.com
X-ray diffraction	Bruker	https://bruker.com
HRMS	Agilent	https://www.agilent.com.cn/

### Resource availability

#### Lead contact

Further information and requests for resources should be directed to and will be fulfilled by the lead contact, Zuxiao Zhang (zhangzx@zjnu.edu.cn).

#### Materials availability

All other datasupporting the findings of this study are available within the article and the supplemental information or from the lead contact upon reasonable request.

### Method details

#### Preparation of substrates

General procedure for the synthesis of N-F.

These substrates can be synthesized from Mitsunobu Reaction with 2-Aryl-2,3-butadien-1-ol and TsNHBoc, then the Boc protecting group can be removed by TFA.1.

**Figure:**
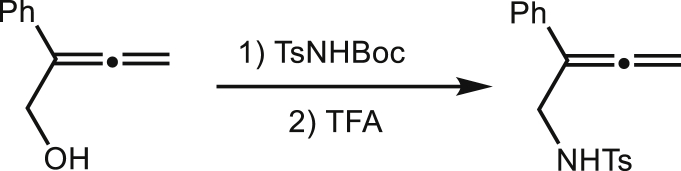

2-Phenyl-2, 3-butadien-1-ol (1.46g, 10 mmol), TsNHBoc (3.53g, 13 mmol) and PPh3 (3.41g 13 mmol) were suspended in THF (15 ml). The mixture was cooled to 0°C and diethyl azodicarboxylate (DEAD 2.61g, 15 mmol) was added dropwisely. Then the reaction mixture was allowed to warm to room temperature. Water was added when the starting material was disappeared and the mixture was extracted with Et_2_O. The combined organic extracts were dried overMgSO_4_. After solvent evaporated, the residue was purified through silica gel to give the allenyl imide product. The allenyl imide was treated with TFA following the process described as above to give the product (1.94g, 65% for two steps).

**Figure:**
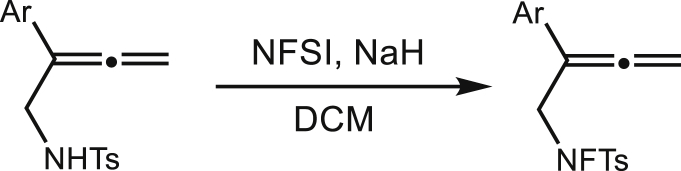

In an oven dried round bottom flask with stir bar, sodium hydride (10 mmol, 2 equiv.) was taken. The sodium hydride was washed with pentane (2 times) and dried under vacuum and filled with nitrogen. Then dry DCM (40 mL) was added to it. A solution of sulfonamide (1 equiv.) in dry DCM (0.5 M) was added dropwise to the NaH suspension in DCM. The total reaction was stirred at room temperature for 30 mins. Then, a solution of NFSI (3 eq.) in dry DCM (0.5 M) was added to dropwise to the reaction mixture at room temperature. The total reaction mixture was stirred for overnight at room temperature. The reaction was quenched with ice with constant stirring. Then 50 mL of water was added to the reaction mixture. The organic part was washed with 30 mL NaHCO_3_, and 30 mL brine solution respectively. The organic part was concentrated in rotary evaporator and performed silica gel flash column chromatography to isolate the desired N-F (fluorosulfonamide, 25%-50% yield) using hexanes/ethyl acetate mixture as eluent.

#### Preparation of products

General procedure for the synthesis of products. Related to Scheme 1, 2.

**Figure:**
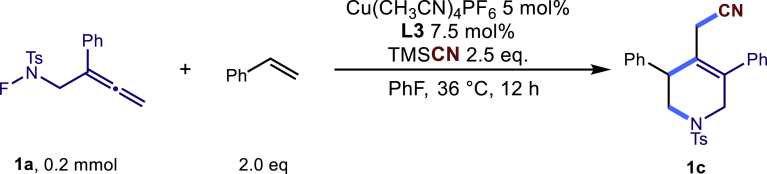

##### Procedure A

In a dried sealed 10 mL Schlenk tube, Cu(CH_3_CN)_4_PF_6_ (5 mol%), bisoxazoline ligand L3 (7.5 mol %) were dissolved in a mixed solvent of PhF (2.0 mL) under a N2 atmosphere, and the mixture was stirred for 30 min. Then substrate N-F (**1a**, 63.4 mg, 0.2 mmol, 1.0 eq.), styrene (**1b**, 42.0mg, 0.4 mmol, 2.0 eq.) and TMSCN (67 μl, 0.5 mmol, 2.5 equiv.) were added sequentially into the above solution. The tube was sealed with Teflon septum and the reaction mixture was stirred at 36°C for another 12 hours. After the reaction was completed, the mixture was quenched by a short pad of silica gel with a gradient eluent of petroleum ether and ethyl acetate, solvent was removed under vacuum, and the residue was purified by column chromatography on silica gel with a gradient eluent of petroleum ether and ethyl acetate (Petroleum ether: EtOAc = 10:1) to give the desired product **1c** in 65% yield (55.8 mg).

**Figure:**
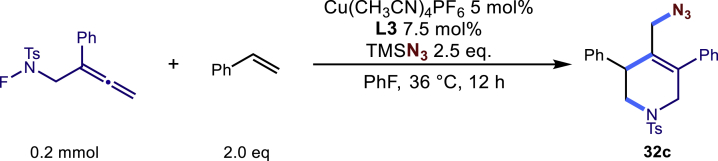

##### Procedure A-1

In a dried sealed 10 mL Schlenk tube, Cu(CH3CN)4PF6 (5 mol%), bisoxazoline ligand L3 (7.5 mol %) were dissolved in a mixed solvent of PhF (2.0 mL) under a N2 atmosphere, and the mixture was stirred for 30 min. Then substrate N-F (1a, 63.4 mg, 0.2 mmol, 1.0 eq.), styrene (1b 42.0mg, 0.4 mmol, 2.0 eq.) and TMSN3 (0.5 mmol, 2.5 equiv.) were added sequentially into the above solution. The tube was sealed with Teflon septum and the reaction mixture was stirred at 36°C for another 12 hours. After the reaction was completed, the mixture was quenched by a short pad of silica gel with a gradient eluent of petroleum ether and ethyl acetate, solvent was removed under vacuum, and the residue was purified by column chromatography on silica gel with a gradient eluent of petroleum ether and ethyl acetate (Petroleum ether: EtOAc = 10:1) to give the desired product 32c in 66% yield (58.9mg).

**Figure:**
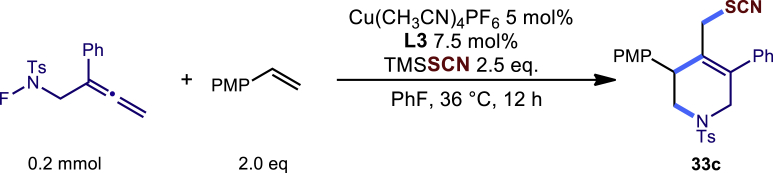

##### Procedure A-2

In a dried sealed 10 mL Schlenk tube, Cu(CH_3_CN)_4_PF_6_ (5 mol%), bisoxazoline ligand L3 (7.5 mol %) were dissolved in a mixed solvent of PhF (2.0 mL) under a N_2_ atmosphere, and the mixture was stirred for 30 min. Then substrate N-F (**1a**, 63.4 mg, 0.2 mmol, 1.0 eq.), 4-methoxystyrene (54 mg, 0.4 mmol, 2.0 eq.) and TMSSCN (0.5 mmol, 2.5 equiv.) were added sequentially into the above solution. The tube was sealed with Teflon septum and the reaction mixture was stirred at 36°C for another 12 hours. After the reaction was completed, the mixture was quenched by a short pad of silica gel with a gradient eluent of petroleum ether and ethyl acetate, solvent was removed under vacuum, and the residue was purified by column chromatography on silica gel with a gradient eluent of petroleum ether and ethyl acetate (Petroleum ether: EtOAc =5:1) to give the desired product **33c** in 60% yield (59.0 mg).

**Figure:**
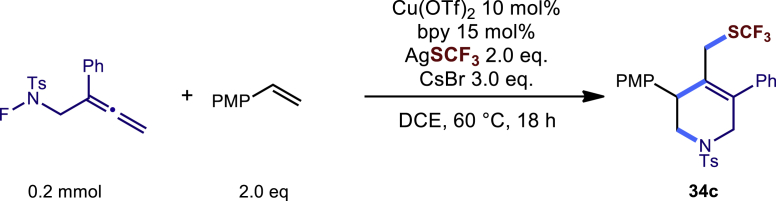

##### Procedure B

In a dried sealed 10 mL Schlenk tube, Cu(OTf)_2_ (10 mol %), bpy (15 mol %) were dissolved in a mixed solvent of DCE (2.0 mL) under a N_2_ atmosphere, and the mixture was stirred for 30 min. Then substrate N-F (**1a**, 63.4 mg, 0.2 mmol, 1.0 eq.), 4-methoxystyrene (54 mg, 0.4 mmol, 2.0 eq.), AgSCF_3_ (83 mg, 0.4 mmol, 2.0 eq.) and CsBr (127 mg, 0.6 mmol, 3.0 eq.) were added sequentially into the above solution. The tube was sealed with Teflon septum and the reaction mixture was stirred at 60°C for another 18 hours. After the reaction was completed, the mixture was quenched by a short pad of silica gel with a gradient eluent of petroleum ether and ethyl acetate, solvent was removed under vacuum, and the residue was purified by column chromatography on silica gel with a gradient eluent of petroleum ether and diethyl ether (Petroleum ether: diethyl ether = 15:1) to give the desired product **34c** in 40% yield (42.7 mg).

**Figure:**
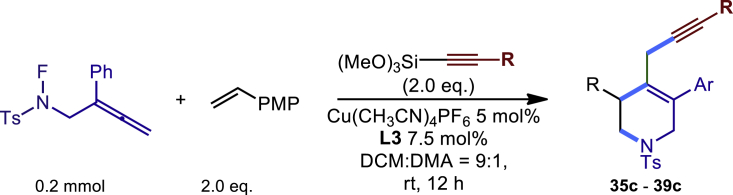

##### Procedure C

In a dried sealed 10 mL Schlenk tube, Cu(CH_3_CN)_4_PF_6_ (5 mol%), bisoxazoline ligand L3 (7.5 mol %) were dissolved in a mixed solvent of DCM and DMA (9:1, 2.0 mL, v/v = 9:1) under a N2 atmosphere, and the mixture was stirred for 30 min. Then substrate N-F (**1a**, 63.4 mg, 0.2 mmol, 1.0 eq.), 4-methoxystyrene (54 mg, 0.4 mmol, 2.0 eq.), and alkynyltrimethoxysilane (0.4 mmol, 2.0 eq.) were added sequentially into the above solution. The tube was sealed with Teflon septum and the reaction mixture was stirred at room temperature for another 12 hours. After the reaction was completed, the mixture was quenched by a short pad of silica gel with a gradient eluent of petroleum ether and ethyl acetate, solvent was removed under vacuum, and the residue was purified by column chromatography on silica gel with a gradient eluent of petroleum ether and ethyl acetate to give the desired product **35c-39c**.

#### Transformations of products

General procedure for the transformations of **3c** and **20c**. Related to Figure 2.

**Figure:**
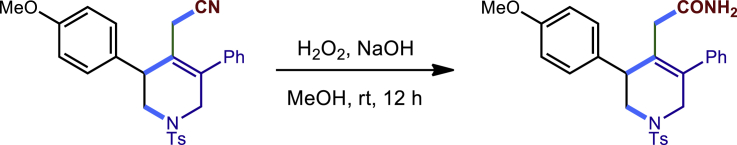

**3c** (45.8 mg, 0.1 mmol) was dissolved in 1 mL methanol followed by the addition of 0.15 mL 30% H_2_O_2_, and the pH of the solution is adjusted to 8 by 1 drop of 2 M NaOH solution. The mixture was stirred for 12hat room temperature. Then, the solvent was removed under vacuum and the residue was subject to a short plug of silica gel, eluted with EtOAc in petroleum ether to give the product **40** in 72% yield as a white solid (34.3 mg). Rf = 0.15 (Petroleum ether: EtOAc = 1:1)

**Figure:**
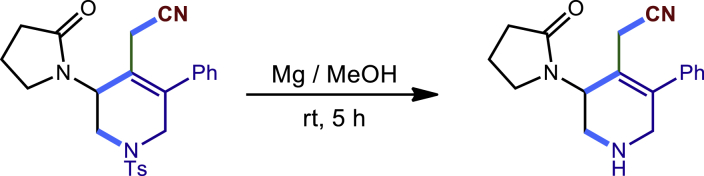

To a solution of **20c** (87.0 mg, 0.2 mmol) in anhydride methanol (2 mL) was added Mg turnings (6.0 eq.) and the reaction mixture was stirred under sonication for 5h at room temperature. After the completion of the reaction, the mixture was quenched with brine, and extracted with DCM. The combined organic layers were dried overNa_2_SO_4_ and concentrated in vacuum. The residue was purified by column chromatography to provide the desired product **41** as a yellow solid (46.6 mg, 83% yield). Rf = 0.1 (MeOH: DCM =1:9)

**Figure:**
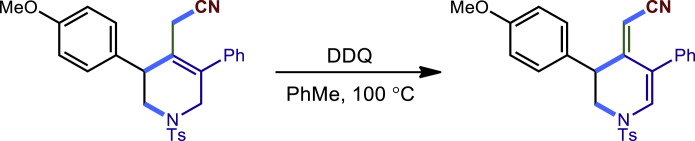

To a solution of **3c** (45.8 mg, 0.1 mmol) in 2 mL of PhMe was added DDQ (45.4mg, 0.20 mmol), and the reaction was stirred at 100°C for 12 hourstill 3c was completely consumed (monitored by TLC). The mixture was cooled to room temperature and concentrated under reduced pressure. The resulting crude residue was purified via column chromatography on silica gel (8:1 hexanes/EtOAc) to afford the desired product **42** with 70% (32.0 mg) yield.

**Figure:**
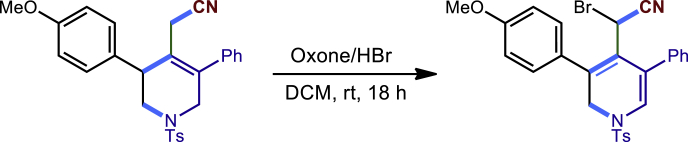

To a solution of **3c** (45.8 mg, 0.1 mmol) in 2 mL of DCM was added oxone (1.6 eq.), was added 2N HBr (2 eq.) in one portion result in dark colored solution. The reaction was stirred at room temperature for 18 hourstill **3c** was completely consumed (monitored by TLC). Then, the reaction was quenched with 5 mL sodium thiosulfate saturated solution, and extracted with DCM. The combined organic layers were dried overNa_2_SO_4_ and concentrated in vacuum. The residue was purified via column chromatography on silica gel (10:1 hexanes/EtOAc) to afford the desired product **43** with 56% (30 mg) yield.

#### Characterization of substrates 2a-10a

##### N-fluoro-4-methyl-N-(2-(p-tolyl)buta-2,3-dien-1-yl)benzenesulfonamide (2a)

**Figure:**
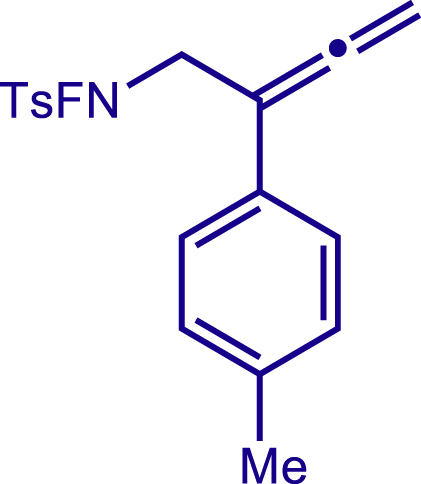

^1^H NMR (400 MHz, CDCl_3_) δ 7.87 (d, *J* = 7.6 Hz, 2H), 7.42 (d, *J* = 7.7 Hz, 2H), 7.34 (d, *J* = 7.4 Hz, 2H), 7.16 (d, *J* = 7.4 Hz, 2H), 5.16 (s, 2H), 4.26 (d, *J* = 39.8 Hz, 2H), 2.49 (s, 3H), 2.34 (s, 3H).

^13^C NMR (151 MHz, CDCl_3_) δ 210.62, 146.45, 137.15, 130.21, 130.07, 130.00, 129.34, 128.91, 126.06, 98.13, 79.24, 54.74 (d, *J* = 13.1 Hz), 21.85, 21.14.

^19^F NMR (377 MHz, Chloroform-d) δ -46.98 (t, *J* = 39.8 Hz).

HRMS (ESI) for C_18_H_19_FNO_2_S[M+H]^+^m/z: calcd 332.1115, found 332.1100.

##### N-fluoro-N-(2-(4-methoxyphenyl)buta-2,3-dien-1-yl)-4-methylbenzenesulfonamide (3a)

**Figure:**
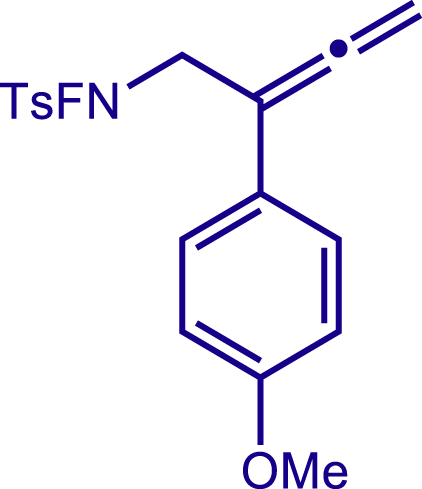

^1^H NMR (400 MHz, CDCl_3_) δ 7.87 (d, *J* = 8.1 Hz, 2H), 7.42 (d, *J* = 8.1 Hz, 2H), 7.38 (d, *J* = 8.7 Hz, 2H), 6.89 (d, *J* = 8.7 Hz, 2H), 5.15 (s, 2H), 4.24 (d, J = 39.9 Hz, 2H), 3.81 (s, 3H), 2.49 (s, 3H).

^13^C NMR (101 MHz, CDCl_3_) δ 210.46, 158.95, 146.48, 130.10, 130.01, 128.88, 127.40, 125.39, 114.11, 97.82, 79.26, 55.35, 54.94 (d, *J* = 13.3 Hz), 21.86.

^19^F NMR (377 MHz, CDCl_3_) δ -47.01 (t, *J* = 39.9 Hz).

##### N-fluoro-N-(2-(4-fluorophenyl)buta-2,3-dien-1-yl)-4-methylbenzenesulfonamide (4a)

**Figure:**
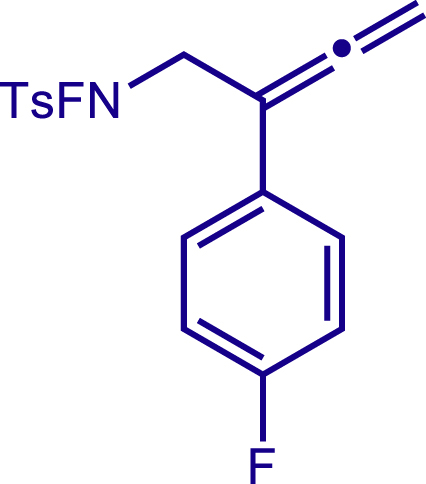

^1^H NMR (400 MHz, CDCl_3_) δ 7.87 (d, *J* = 8.3 Hz, 2H), 7.46 – 7.39 (m, 4H), 7.04 (t, *J* = 8.7 Hz, 2H), 5.18 (s, 2H), 4.25 (d, *J* = 39.6 Hz, 2H), 2.49 (s, 3H).

^13^C NMR (101 MHz, Chloroform-d) δ 210.71, 162.12 (d, *J* = 247.2 Hz), 146.57, 130.12, 130.00, 129.21 (d, *J* = 3.2 Hz), 128.85, 127.87 (d, *J* = 8.1 Hz), 115.57 (d, *J* = 21.7 Hz), 97.53, 79.48, 54.82 (d, *J* = 13.2 Hz), 21.84.

^19^F NMR (377 MHz, Chloroform-d) δ -46.99 (t, *J* = 39.6 Hz),-114.91 – -115.03 (m).

HRMS (ESI) for C_17_H_16_F_2_NO_2_S[M+H]^+^m/z: calcd 336.0864, found 336.0869.

##### N-fluoro-N-(2-(4-chlorophenyl)buta-2,3-dien-1-yl)- 4-methylbenzenesulfonamide (5a)

**Figure:**
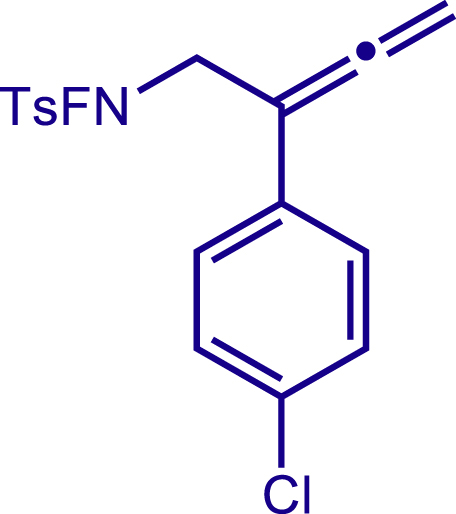

^1^H NMR (400 MHz, Chloroform-d) δ 7.86 (d, *J* = 8.2 Hz, 2H), 7.43 (d, *J* = 8.1 Hz, 2H), 7.38 (d, *J* = 8.6 Hz, 2H), 7.31 (d, *J* = 8.7 Hz, 2H), 5.19 (s, 2H), 4.25 (d, J = 39.6 Hz, 2H), 2.49 (s, 3H).

^13^C NMR (101 MHz, Chloroform-d) δ 210.84, 146.63, 133.15, 131.78, 130.15, 130.00, 128.77, 127.49, 97.56, 79.73, 54.58 (d, *J* = 13.3 Hz), 21.87.

^19^F NMR (377 MHz, Chloroform-d) δ -47.09 (t, *J* = 39.6 Hz).

HRMS (ESI) for C_17_H_16_ClFNO_2_S[M+H]^+^m/z: calcd 352.0569, found 352.0561.

##### ethyl 4-(1-((N-fluoro-4-methylphenyl)sulfonamido)buta-2,3-dien-2-yl)benzoate (6a)

**Figure:**
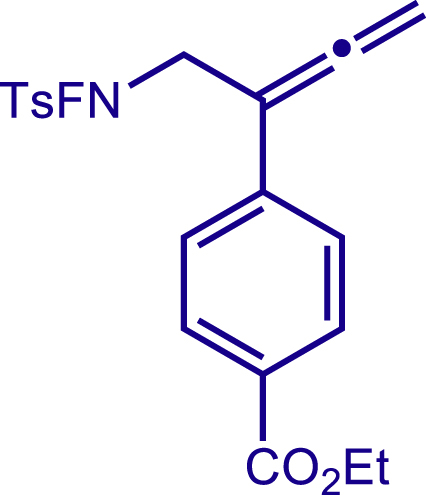

^1^H NMR (400 MHz, CDCl_3_) δ 8.02 (d, *J* = 8.5 Hz, 2H), 7.87 (d, *J* = 8.3 Hz, 2H), 7.51 (d, *J* = 8.5 Hz, 2H), 7.43 (d, *J* = 8.1 Hz, 2H), 5.25 (s, 2H), 4.46 – 4.31 (m, 2H), 4.26 (s, 2H), 2.49 (s, 3H), 1.39 (t, *J* = 7.1 Hz, 3H).

^13^C NMR (101 MHz, CDCl_3_) δ 211.59, 166.35, 146.61, 138.03, 130.13, 130.00, 129.83, 129.19, 128.90, 126.02, 98.04, 79.84, 60.98, 54.32 (d, *J* = 12.7 Hz), 21.86, 14.35.

^19^F NMR (377 MHz, CDCl_3_) δ -46.95 (t, *J* = 39.5 Hz).

HRMS (ESI) for C_20_H_21_FNO_4_S[M+H]^+^m/z: calcd 390.1170, found 390.1145.

##### N-fluoro-4-methyl-N-(2-(m-tolyl)buta-2,3-dien-1-yl)benzenesulfonamide (7a)

**Figure:**
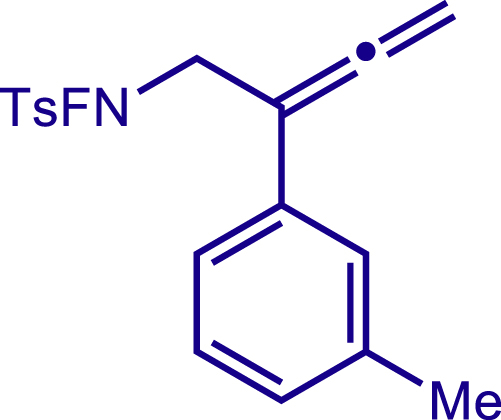

^1^H NMR (600 MHz, CDCl_3_) δ 7.88 (d, *J* = 8.3 Hz, 2H), 7.42 (d, *J* = 8.1 Hz, 2H), 7.28 – 7.24 (m, 3H), 7.07 (s, 1H), 5.17 (s, 2H), 4.27 (d, *J* = 39.8 Hz, 2H), 2.49 (s, 3H), 2.36 (s, 3H).

^13^C NMR (151 MHz, CDCl_3_) δ 210.81, 146.47, 138.20, 133.14, 130.09, 130.00, 128.91, 128.52, 128.17, 126.97, 123.17, 98.28, 79.25, 54.73 (d, *J* = 12.9 Hz), 21.85, 21.54.

^19^F NMR (377 MHz, Chloroform-d) δ -46.99.

HRMS (ESI) for C_18_H_19_FNO_2_S[M+H]^+^m/z: calcd 332.1115, found 332.1100.

##### N-fluoro-4-methyl-N-(2-(o-tolyl)buta-2,3-dien-1-yl)benzenesulfonamide (8a)

**Figure:**
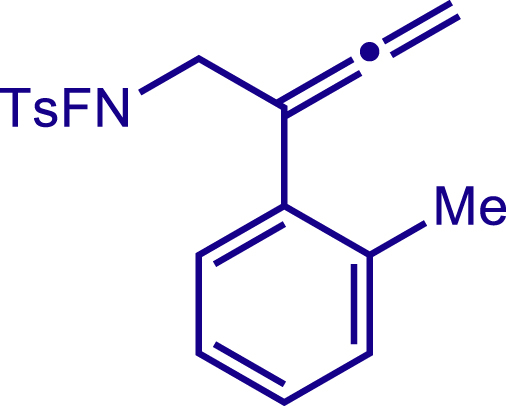

^1^H NMR (400 MHz, CDCl_3_) δ 7.83 (d, *J* = 7.9 Hz, 2H), 7.40 (d, *J* = 7.9 Hz, 2H), 7.26 – 7.16 (m, 4H), 4.94 (s, 2H), 4.13 (d, *J* = 40.1 Hz, 2H), 2.48 (s, 3H), 2.33 (s, 3H).

^13^C NMR (101 MHz, CDCl3) δ 209.20, 146.43, 136.66, 133.71, 130.67, 130.07, 129.99, 128.93, 128.30, 127.77, 126.05, 97.20, 77.01, 57.23 (d, *J* = 12.3 Hz), 21.83, 20.26.

^19^F NMR (377 MHz, CDCl_3_) δ -46.78 (t, *J* = 40.1 Hz).

HRMS (ESI) for C_18_H_19_FNO_2_S[M+H]^+^m/z: calcd 332.1115, found 332.1101.

##### N-fluoro-4-methyl-N-(2-(naphthalen-1-yl)buta-2,3-dien-1-yl)benzenesulfonamide (9a)

**Figure:**
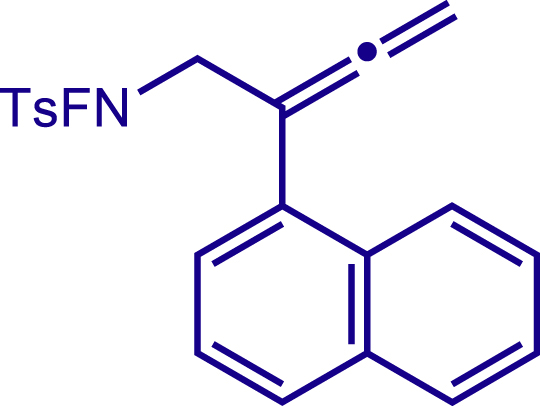

^1^H NMR (400 MHz, Chloroform-d) δ 8.08 – 8.03 (m, 1H), 7.88 – 7.84 (m, 1H), 7.81 (d, *J* = 8.2 Hz, 3H), 7.51 – 7.46 (m, 4H), 7.38 (d, *J* = 7.8 Hz, 2H), 5.03 (t, *J* = 2.4 Hz, 2H), 4.25 (d, *J* = 39.9 Hz, 2H), 2.47 (s, 3H).

^13^C NMR (101 MHz, Chloroform-d) δ 209.70, 146.40, 133.96, 132.21, 131.34, 130.04, 129.99, 128.97, 128.54, 128.37, 126.35, 126.29, 125.91, 125.42, 125.00, 96.29, 77.17, 57.49 (d, *J* = 12.5 Hz), 21.81.

^19^F NMR (377 MHz, Chloroform-d) δ -46.28 (t, *J* = 39.9 Hz).

HRMS (ESI) for C_21_H_19_FNO_2_S[M+H]^+^m/z: calcd 368.1115, found 368.1092.

##### N-fluoro-4-methyl-N-(2-(naphthalen-2-yl)buta-2,3-dien-1-yl)benzenesulfonamide (10a)

**Figure:**
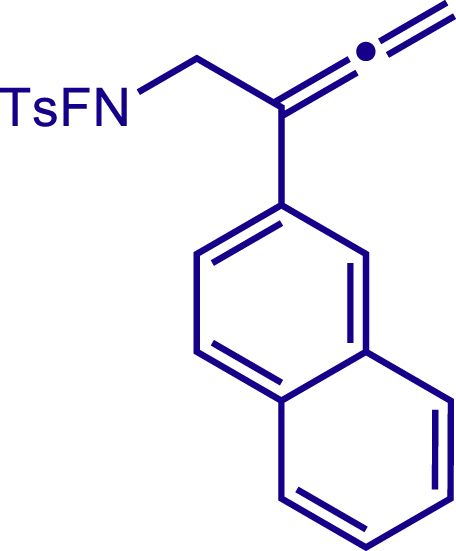

^1^H NMR (400 MHz, Chloroform-d) δ 8.00 – 7.83 (m, 4H), 7.79 (d, *J* = 8.6 Hz, 2H), 7.59 (d, *J* = 8.6 Hz, 1H), 7.53 – 7.45 (m, 2H), 7.43 (d, *J* = 8.1 Hz, 2H), 5.27 (s, 2H), 4.41 (d, *J* = 39.7 Hz, 2H), 2.49 (s, 3H).

^13^C NMR (101 MHz, Chloroform-d) δ 211.42, 146.51, 133.48, 132.57, 130.58, 130.11, 130.03, 129.03, 128.29, 128.15, 127.56, 126.31, 126.09, 124.80, 124.44, 98.58, 79.69, 54.67 (d, *J* = 13.1 Hz), 21.84.

^19^F NMR (377 MHz, Chloroform-d) δ -46.48 (t, *J* = 39.8 Hz).

HRMS (ESI) for C_21_H_19_FNO2S[M+H]^+^m/z: calcd 368.1115, found 368.1095.

#### Characterization of products 1c-39c

##### 2-(3,5-diphenyl-1-tosyl-1,2,3,6-tetrahydropyridin-4-yl)acetonitrile (1c)

**Figure:**
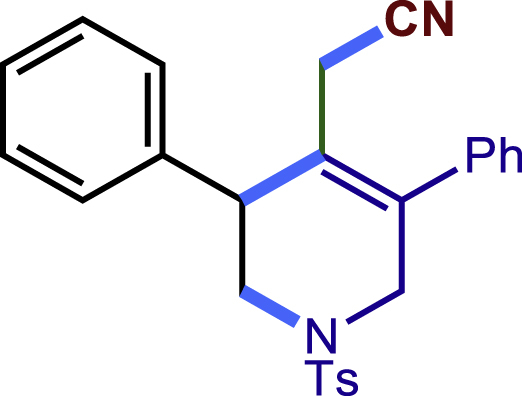

Prepared following general **procedure A.**

^1^H NMR (400 MHz, CDCl_3_) δ 7.61 (d, *J* = 8.3 Hz, 2H), 7.45 – 7.27 (m, 10H), 7.25 – 7.21 (m, 2H), 4.07 (d, *J* = 16.7 Hz, 1H), 3.80 (s, 1H), 3.66 (d, *J* = 16.7 Hz, 1H), 3.48 (dd, *J* = 11.8, 4.5 Hz, 1H), 3.35 (dd, *J* = 11.8, 4.9 Hz, 1H), 2.97 (d, *J* = 17.2 Hz, 1H), 2.58 (d, *J* = 17.3 Hz, 1H), 2.42 (s, 3H).

^13^C NMR (101 MHz, CDCl_3_) δ 143.99, 139.30, 137.22, 136.81, 132.74, 129.84, 129.13, 129.07, 128.68, 128.55, 128.37, 127.93, 127.74, 124.33, 117.29, 49.91, 49.66, 44.09, 21.58, 20.42.

HRMS (ESI) for C_26_H_25_N_2_O_2_S[M+H]^+^m/z: calcd 429.1631, found 429.1638.

##### 2-(5-phenyl-3-(p-tolyl)-1-tosyl-1,2,3,6-tetrahydropyridin-4-yl)acetonitrile (2c)

**Figure:**
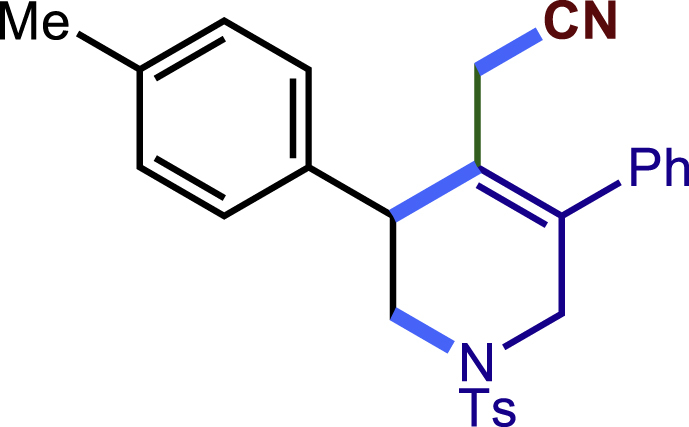

Prepared following general **Procedure A** using **1a** (63.4 mg, 0.2 mmol), 2b (48 mg, 0.4 mmol). After 12 hours, the reaction mixture was purified by column chromatography to provide the compound **2c** (61.0 mg, 69% yield) as a white solid. Rf = 0.2 (Petroleum ether: EtOAc =10:1).

^1^H NMR (400 MHz, CDCl_3_) δ 7.61 (d, *J* = 8.2 Hz, 2H), 7.46 – 7.35 (m, 3H), 7.29 (d, *J* = 8.0 Hz, 2H), 7.24 – 7.14 (m, 6H), 4.04 (d, *J* = 16.6 Hz, 1H), 3.77 (s, 1H), 3.67 (d, *J* = 16.6 Hz, 1H), 3.42 (dd, *J* = 11.8, 4.7 Hz, 1H), 3.36 (dd, *J* = 11.8, 5.0 Hz, 1H), 2.95 (d, *J* = 17.2 Hz, 1H), 2.59 (d, *J* = 17.2 Hz, 1H), 2.42 (s, 3H), 2.35 (s, 3H).

^13^C NMR (101 MHz, CDCl_3_) δ 143.95, 137.60, 137.30, 136.60, 136.22, 132.81, 129.82, 129.74, 129.11, 128.64, 128.41, 128.38, 127.73, 124.55, 117.33, 50.04, 49.68, 43.74, 21.57, 21.15, 20.36.

HRMS (ESI) for C_27_H_27_N_2_O_2_S[M+H]+ m/z: calcd 443.1788, found 443.1176.

##### 2-(3-(4-methoxyphenyl)-5-phenyl-1-tosyl-1,2,3,6-tetrahydropyridin-4-yl)acetonitrile (3c)

**Figure:**
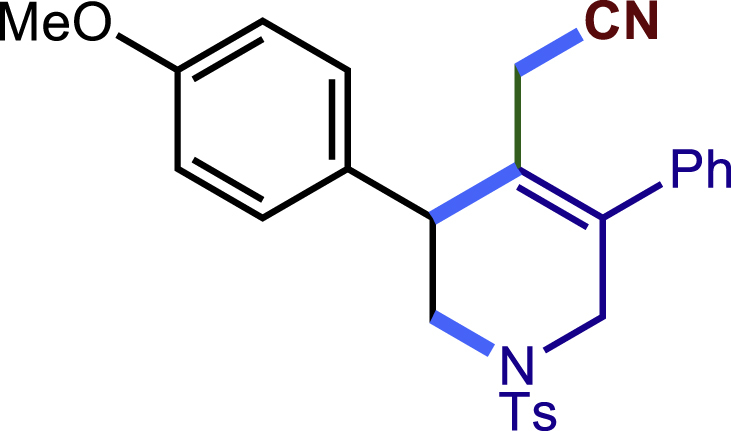

Prepared following general **Procedure A** using **1a** (63.4 mg, 0.2 mmol), 3b (54 mg, 0.4 mmol). After 12 hours, the reaction mixture was purified by column chromatography to provide the compound **3c** (70.7 mg, 77% yield) as a white solid. Rf = 0.2 (Petroleum ether: EtOAc = 5:1).

^1^H NMR (400 MHz, CDCl_3_) δ 7.61 (d, *J* = 8.2 Hz, 2H), 7.46 – 7.35 (m, 3H), 7.30 (d, *J* = 8.1 Hz, 2H), 7.23 (t, *J* = 8.3 Hz, 4H), 6.89 (d, *J* = 8.6 Hz, 2H), 4.05 (d, *J* = 16.6 Hz, 1H), 3.81 (s, 3H), 3.76 (s, 1H), 3.65 (d, *J* = 16.7 Hz, 1H), 3.43 (dd, *J* = 11.8, 4.5 Hz, 1H), 3.33 (dd, *J* = 11.7, 4.9 Hz, 1H), 2.95 (d, *J* = 17.2 Hz, 1H), 2.60 (d, *J* = 17.2 Hz, 1H), 2.42 (s, 3H).

^13^C NMR (101 MHz, CDCl_3_) δ 159.24, 143.98, 137.29, 136.47, 132.77, 131.27, 129.84, 129.58, 129.12, 128.65, 128.38, 127.73, 124.69, 117.35, 114.43, 55.31, 50.09, 49.67, 43.32, 21.57, 20.36.

HRMS (ESI) for C_27_H_27_N_2_O_3_S[M+H]^+^m/z: calcd 459.1737, found 459.1724.

##### 2-(3-(4-(tert-butyl)phenyl)-5-phenyl-1-tosyl-1,2,3,6-tetrahydropyridin-4-yl)acetonitrile (4c)

**Figure:**
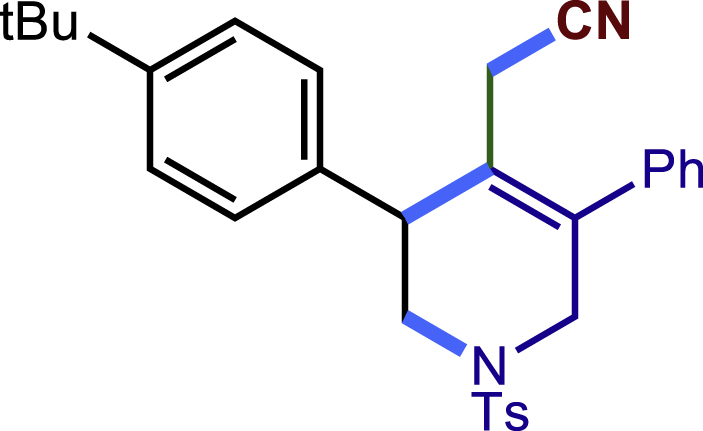

Prepared following general **Procedure A** using **1a** (63.4 mg, 0.2 mmol), 4b (64 mg, 0.4 mmol). After 12 hours, the reaction mixture was purified by column chromatography to provide the compound **4c** (55.2 mg, 57% yield) as a white solid. Rf = 0.3 (Petroleum ether: EtOAc = 5:1).

^1^H NMR (400 MHz, CDCl_3_) δ 7.61 (d, *J* = 8.3 Hz, 2H), 7.45 – 7.34 (m, 5H), 7.30 (d, *J* = 8.0 Hz, 2H), 7.25 – 7.20 (m, 4H), 4.04 (d, *J* = 16.6 Hz, 1H), 3.78 (s, 1H), 3.67 (d, *J* = 16.6 Hz, 1H), 3.45 (dd, *J* = 11.7, 4.7 Hz, 1H), 3.37 (dd, *J* = 11.8, 5.0 Hz, 1H), 2.95 (d, *J* = 17.2 Hz, 1H), 2.59 (d, *J* = 17.2 Hz, 1H), 2.42 (s, 3H), 1.32 (s, 9H).

^13^C NMR (101 MHz, CDCl_3_) δ 150.72, 143.97, 137.34, 136.40, 136.21, 132.71, 129.83, 129.09, 128.62, 128.39, 128.20, 127.79, 125.95, 124.67, 117.38, 49.90, 49.66, 43.56, 34.55, 31.37, 21.58, 20.38.

##### 2-(3-(4-chlorophenyl)-5-phenyl-1-tosyl-1,2,3,6-tetrahydropyridin-4-yl)acetonitrile(5c)

**Figure:**
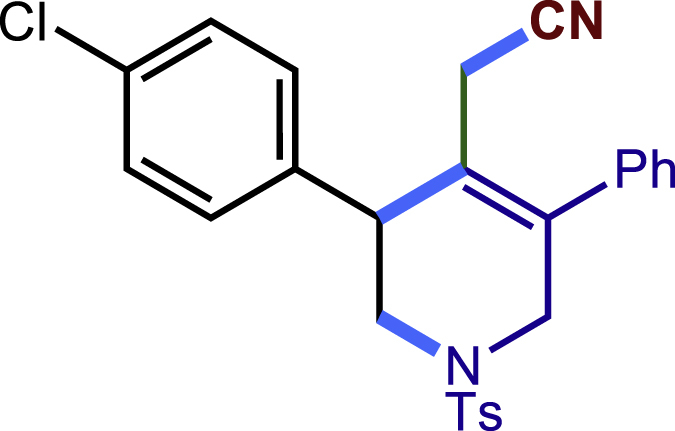

Prepared following general **Procedure A** using **1a** (63.4 mg, 0.2 mmol), 5b (56 mg, 0.4 mmol). After 12 hours, the reaction mixture was purified by column chromatography to provide the title compound **5c** (52.8 mg, 57% yield) as a white solid. Rf = 0.25 (Petroleum ether: EtOAc =10:1).

^1^H NMR (400 MHz, CDCl_3_) δ 7.59 (d, *J* = 8.3 Hz, 2H), 7.46 – 7.36 (m, 3H), 7.36 – 7.26 (m, 6H), 7.25 – 7.20 (m, 2H), 4.15 (d, J = 16.7 Hz, 1H), 3.77 (s, 1H), 3.61 – 3.50 (m, 2H), 3.24 (dd, *J* = 11.8, 4.7 Hz, 1H), 2.99 (d, *J* = 17.3 Hz, 1H), 2.56 (d, *J* = 17.4 Hz, 1H), 2.42 (s, 3H).

^13^C NMR (101 MHz, CDCl_3_) δ 144.12, 137.91, 137.20, 136.95, 133.84, 132.66, 129.87, 129.82, 129.23, 129.19, 128.81, 128.31, 127.70, 123.79, 117.17, 49.69, 49.59, 43.42, 21.58, 20.48.

HRMS (ESI) for C_26_H_24_ClN_2_O_2_S[M+H]+ m/z: calcd 463.1242, found 463.1227.

##### 2-(3-(4-(chloromethyl)phenyl)-5-phenyl-1-tosyl-1,2,3,6-tetrahydropyridin-4-yl)acetonitrile (6c)

**Figure:**
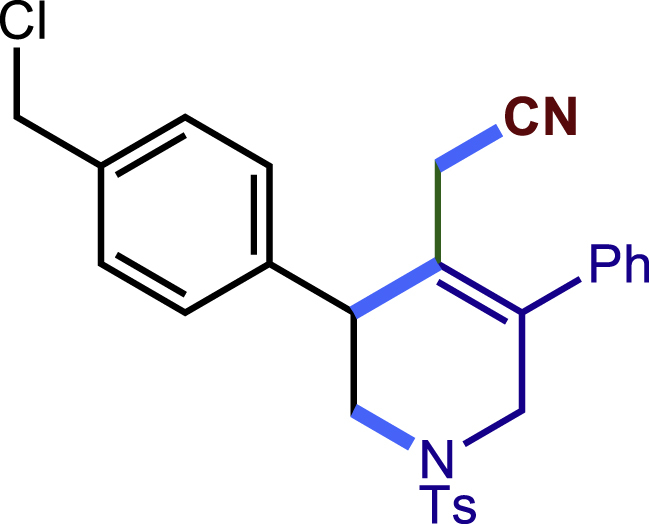

Prepared following general **Procedure A** using **1a** (63.4 mg, 0.2 mmol), 6b (61 mg, 0.4 mmol). After 12 hours, the reaction mixture was purified by column chromatography to provide the compound **6c** (68.4 mg, 72% yield) as a white solid. Rf = 0.2 (Petroleum ether: EtOAc =10:1).

^1^H NMR (400 MHz, CDCl3) δ 7.60 (d, *J* = 8.2 Hz, 2H), 7.46 – 7.37 (m, 5H), 7.35 – 7.29 (m, 4H), 7.24 – 7.21 (m, 2H), 4.59 (s, 2H), 4.11 (d, *J* = 16.6 Hz, 1H), 3.81 (s, 1H), 3.61 (d, *J* = 16.7 Hz, 1H), 3.51 (dd, *J* = 11.8, 4.0 Hz, 1H), 3.29 (dd, *J* = 11.8, 4.8 Hz, 1H), 2.98 (d, *J* = 17.3 Hz, 1H), 2.57 (d, *J* = 17.3 Hz, 1H), 2.42 (s, 3H).

^13^C NMR (101 MHz, CDCl3) δ 144.07, 139.68, 137.16, 137.07, 137.01, 132.65, 129.87, 129.29, 129.16, 128.92, 128.75, 128.33, 127.73, 124.02, 117.22, 49.76, 49.62, 45.84, 43.74, 21.57, 20.47.

##### 4-(4-(cyanomethyl)-5-phenyl-1-tosyl-1,2,3,6-tetrahydropyridin-3-yl)phenyl acetate (7c)

**Figure:**
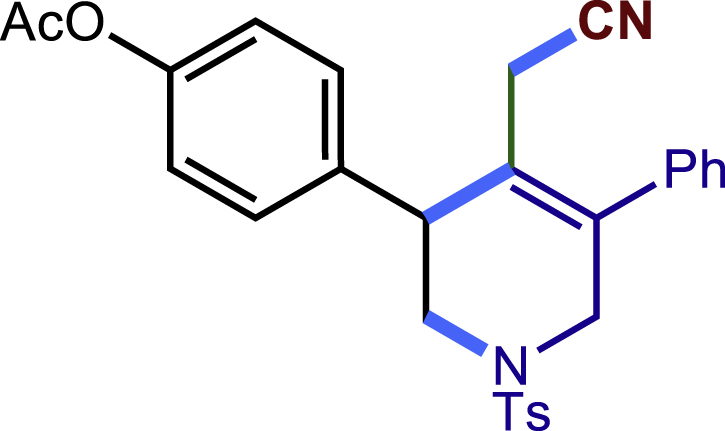

Prepared following general **Procedure A** using **1a** (63.4 mg, 0.2 mmol), 7b (65 mg, 0.4 mmol). After 12 hours, the reaction mixture was purified by column chromatography to provide the compound **7c** (63.3 mg, 65% yield) as a white solid. Rf = 0.2 (Petroleum ether: EtOAc =5:1).

^1^H NMR (400 MHz, CDCl_3_) δ 7.60 (d, *J* = 8.2 Hz, 2H), 7.46 – 7.33 (m, 5H), 7.30 (d, *J* = 8.0 Hz, 2H), 7.24 – 7.19 (m, 2H), 7.09 (d, *J* = 8.5 Hz, 2H), 4.08 (d, *J* = 16.7 Hz, 1H), 3.81 (s, 1H), 3.62 (d, *J* = 16.7 Hz, 1H), 3.48 (dd, *J* = 11.8, 4.2 Hz, 1H), 3.31 (dd, *J* = 11.8, 4.8 Hz, 1H), 2.98 (d, *J* = 17.3 Hz, 1H), 2.59 (d, *J* = 17.3 Hz, 1H), 2.42 (s, 3H), 2.31 (s, 3H).

^13^C NMR (101 MHz, CDCl_3_) δ 169.42, 150.30, 144.10, 137.08, 136.91, 136.86, 132.56, 129.89, 129.55, 129.16, 128.74, 128.33, 127.75, 124.17, 122.16, 117.24, 49.82, 49.64, 43.43, 21.57, 21.21, 20.44.

HRMS (ESI) for C_28_H_27_N_2_O_4_S[M+H]+ m/z: calcd 487.1686, found 487.1668.

##### 2-(3-(3-((3-fluorobenzyl)oxy)phenyl)-5-phenyl-1-tosyl-1,2,3,6-tetrahydropyridin-4-yl)acetonitrile (8c)

**Figure:**
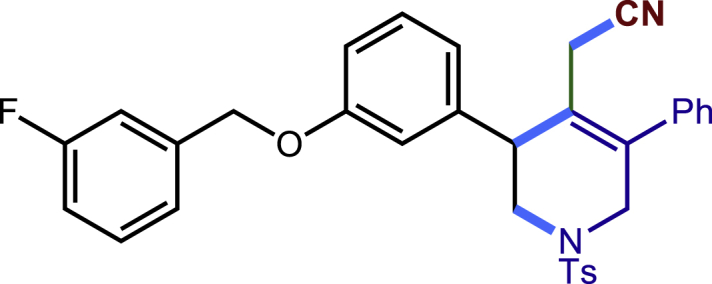

Prepared following general **Procedure A** using **1a** (63.4 mg, 0.2 mmol), **8b** (92 mg, 0.4 mmol). After 12 hours, the reaction mixture was purified by column chromatography to provide the compound **8c** (67.2 mg, 61% yield) as a white solid. Rf = 0.2 (Petroleum ether: EtOAc =5:1).

^1^H NMR (400 MHz, CDCl_3_) δ 7.61 (d, *J* = 8.3 Hz, 2H), 7.46 – 7.33 (m, 4H), 7.32 – 7.26 (m, 3H), 7.26 – 7.15 (m, 5H), 7.02 (td, *J* = 8.3, 2.0 Hz, 1H), 6.98 – 6.93 (m, 2H), 5.06 (s, 2H), 4.07 (d, *J* = 16.6 Hz, 1H), 3.76 (s, 1H), 3.63 (d, *J* = 16.6 Hz, 1H), 3.46 (dd, *J* = 11.7, 4.4 Hz, 1H), 3.31 (dd, *J* = 11.7, 4.8 Hz, 1H), 2.96 (d, *J* = 17.2 Hz, 1H), 2.60 (d, *J* = 17.2 Hz, 1H), 2.42 (s, 3H).

^13^C NMR (101 MHz, CDCl_3_) δ 164.24, 161.80, 158.21, 144.01, 139.56 (d, J = 7.2 Hz), 137.24, 136.52, 132.70, 131.87, 130.21 (d, J = 8.3 Hz), 129.85, 129.68, 129.12, 128.67, 128.38, 127.74, 124.59, 122.80 (d, J = 2.9 Hz), 117.36, 115.30, 114.90 (d, J = 21.0 Hz), 114.28 (d, J = 21.9 Hz), 69.27, 50.03, 49.67, 43.31, 21.58, 20.40.

^19^F NMR (377 MHz, CDCl3) δ -112.71 (td, *J* = 9.2, 6.0 Hz).

HRMS (ESI) for C_33_H_30_FN_2_O_3_S[M+H]+ m/z: calcd 553.1956, found 553.1937.

##### 2-(3-(2,3-dihydrobenzo[b][1,4]dioxin-6-yl)-5-phenyl-1-tosyl-1,2,3,6-tetrahydropyridin-4-yl)acetonitrile (9c)

**Figure:**
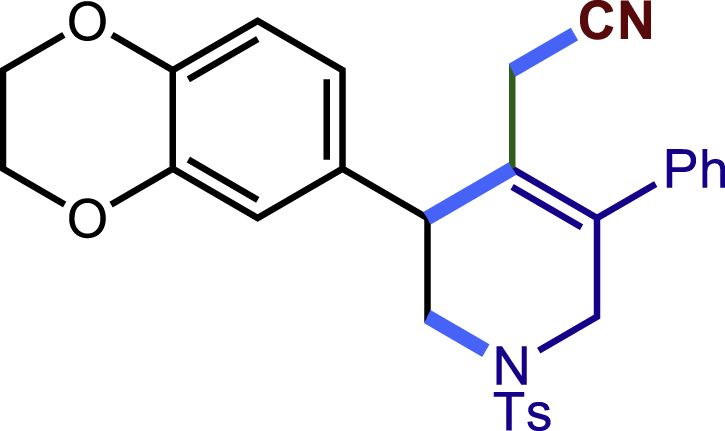

Prepared following general Procedure A using **1a** (63.4 mg, 0.2 mmol), **9b** (65 mg, 0.4 mmol). After 16 hours, the reaction mixture was purified by column chromatography to provide the compound **9c** (69.0 mg, 71% yield) as a white solid. Rf = 0.2 (Petroleum ether: EtOAc =6:1).

^1^H NMR (400 MHz, CDCl_3_) δ 7.61 (d, *J* = 8.2 Hz, 2H), 7.45 – 7.34 (m, 3H), 7.30 (d, *J* = 8.1 Hz, 2H), 7.24 – 7.19 (m, 2H), 6.87 – 6.75 (m, 3H), 4.31 – 4.20 (m, 4H), 4.02 (d, *J* = 16.6 Hz, 1H), 3.69 (s, 1H), 3.63 (d, *J* = 16.7 Hz, 1H), 3.41 (dd, *J* = 11.7, 4.6 Hz, 1H), 3.32 (dd, *J* = 11.7, 4.9 Hz, 1H), 2.95 (d, *J* = 17.2 Hz, 1H), 2.62 (d, *J* = 17.2 Hz, 1H), 2.42 (s, 3H).

^13^C NMR (101 MHz, CDCl_3_) δ 143.97, 143.75, 143.26, 137.25, 136.62, 132.70, 132.44, 129.83, 129.09, 128.64, 128.39, 127.77, 124.49, 121.60, 117.76, 117.35, 117.11, 64.37, 64.32, 50.01, 49.65, 43.39, 21.58, 20.34.

HRMS (ESI) for C_28_H_27_N_2_O_4_S[M+H]+ m/z: calcd 487.1686, found 487.1680.

##### 2-(3-(2-fluoro-4-methoxyphenyl)-5-phenyl-1-tosyl-1,2,3,6-tetrahydropyridin-4-yl)acetonitrile (10c)

**Figure:**
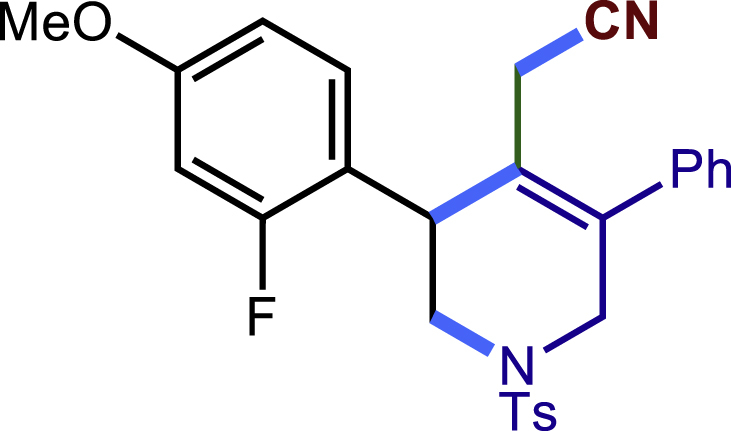

Prepared following general **Procedure A** using **1a** (63.4 mg, 0.2 mmol), **10b** (61 mg, 0.4 mmol). After 16 hours, the reaction mixture was purified by column chromatography to provide the compound **10c** (59.0 mg, 62% yield) as a white solid. Rf = 0.2 (Petroleum ether: EtOAc =8:1).

^1^H NMR (400 MHz, CDCl_3_) δ 7.60 (d, *J* = 8.2 Hz, 2H), 7.46 – 7.35 (m, 3H), 7.30 (d, *J* = 8.2 Hz, 2H), 7.26 – 7.17 (m, 3H), 6.70 (dd, *J* = 8.5, 2.5 Hz, 1H), 6.65 (dd, *J* = 12.0, 2.5 Hz, 1H), 4.10 (d, *J* = 14.9 Hz, 2H), 3.80 (s, 3H), 3.60 (d, *J* = 16.4 Hz, 1H), 3.53 (dd, *J* = 11.8, 4.2 Hz, 1H), 3.25 (dd, *J* = 11.7, 4.5 Hz, 1H), 2.97 (d, *J* = 17.2 Hz, 1H), 2.63 (d, *J* = 17.3 Hz, 1H), 2.42 (s, 3H).

^13^C NMR (101 MHz, CDCl_3_) δ 162.66, 160.55 (d, *J* = 11.1 Hz), 160.22, 144.01, 137.22 (d, *J* = 17.4 Hz), 132.82, 130.39 (d, *J* = 5.7 Hz), 129.85, 129.14, 128.72, 128.34, 127.68, 123.56, 117.57 (d, *J* = 14.5 Hz), 117.15, 110.34 (d, *J* = 2.9 Hz), 102.02 (d, *J* = 25.8 Hz), 55.61, 49.58, 48.79, 36.65, 21.57, 20.48.

^19^F NMR (565 MHz, Chloroform-d) δ -116.30 (t, *J* = 10.3 Hz).

HRMS (ESI) for C_27_H_26_FN2O_3_S[M+H]+ m/z: calcd 477.1643, found 477.1635.

##### 2-(3-(2,3-dihydrobenzofuran-6-yl)-5-phenyl-1-tosyl-1,2,3,6-tetrahydropyridin-4-yl)acetonitrile (11c)

**Figure:**
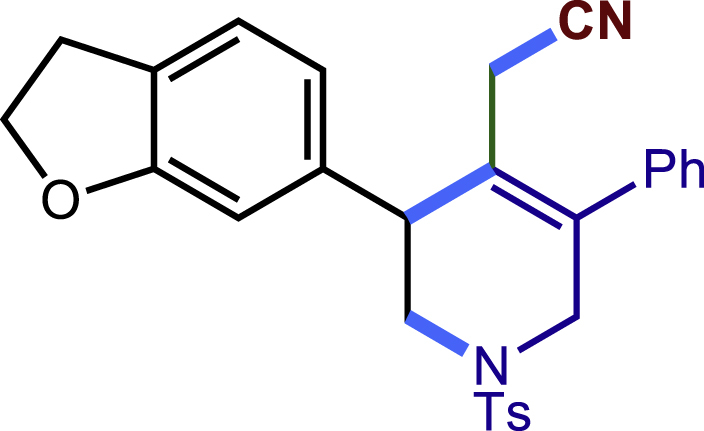

Prepared following general **Procedure A** using **1a** (63.4 mg, 0.2 mmol), **11b** (59 mg, 0.4 mmol). After 18 hours, the reaction mixture was purified by column chromatography to provide the title compound **11c** (49.0 mg, 52% yield) as a white solid. Rf = 0.2 (Petroleum ether: EtOAc =5:1).

^1^H NMR (400 MHz, CDCl3) δ 7.61 (d, *J* = 8.2 Hz, 2H), 7.45 – 7.35 (m, 3H), 7.30 (d, *J* = 8.1 Hz, 2H), 7.25 – 7.20 (m, 2H), 7.16 (s, 1H), 7.01 (dd, *J* = 8.2, 1.6 Hz, 1H), 6.75 (d, *J* = 8.2 Hz, 1H), 4.58 (t, *J* = 8.8 Hz, 2H), 4.03 (d, *J* = 16.6 Hz, 1H), 3.73 (s, 1H), 3.65 (d, *J* = 16.6 Hz, 1H), 3.41 (dd, *J* = 11.7, 4.6 Hz, 1H), 3.33 (dd, *J* = 11.7, 4.9 Hz, 1H), 3.27 – 3.16 (m, 2H), 2.95 (d, *J* = 17.2 Hz, 1H), 2.63 (d, *J* = 17.2 Hz, 1H), 2.42 (s, 3H).

^13^C NMR (101 MHz, CDCl3) δ 159.86, 143.98, 137.32, 136.37, 132.73, 131.19, 129.82, 129.10, 128.63, 128.39, 128.20, 127.87, 127.76, 125.10, 124.84, 117.41, 109.57, 71.43, 50.24, 49.68, 43.58, 29.77, 21.58, 20.36.

HRMS (ESI) for C_28_H_27_N_2_O_3_S[M+H]^+^m/z: calcd 471.1737, found 471.1726.

##### 2-(5-phenyl-1-tosyl-3-(3,4,5-trimethoxyphenyl)-1,2,3,6-tetrahydropyridin-4-yl)acetonitrile (12c)

**Figure:**
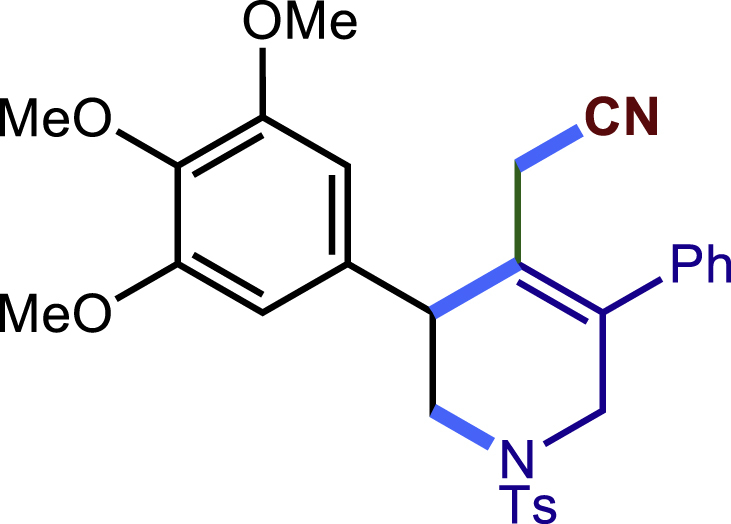

Prepared following general **Procedure A** using **1a** (63.4 mg, 0.2 mmol), **12b** (78 mg, 0.4 mmol). After 8 hours, the reaction mixture was purified by column chromatography to provide the compound **12c** (68.5 mg, 66% yield) as a white solid. Rf = 0.2 (Petroleum ether: EtOAc =2:1).

^1^H NMR (400 MHz, CDCl3) δ 7.61 (d, *J* = 8.3 Hz, 2H), 7.46 – 7.35 (m, 3H), 7.30 (d, *J* = 8.0 Hz, 2H), 7.23 – 7.17 (m, 2H), 6.60 (s, 2H), 4.12 (d, *J* = 16.6 Hz, 1H), 3.88 (s, 6H), 3.85 (s, 3H), 3.69 (s, 1H), 3.63 – 3.55 (m, 2H), 3.28 (dd, *J* = 11.8, 4.8 Hz, 1H), 2.97 (d, *J* = 17.2 Hz, 1H), 2.65 (d, *J* = 17.3 Hz, 1H), 2.42 (s, 3H).

^13^C NMR (101 MHz, CDCl3) δ 153.52, 144.06, 137.59, 137.17, 136.48, 135.19, 132.82, 129.88, 129.17, 128.74, 128.30, 127.66, 124.59, 117.53, 105.52, 60.89, 56.27, 49.57, 49.54, 44.16, 21.57, 20.43.

HRMS (ESI) for C_29_H_31_N_2_O_5_S[M+H]^+^m/z: calcd 519.1948, found 519.1927.

##### 2-(3-(2-bromo-3,4-dimethoxyphenyl)-5-phenyl-1-tosyl-1,2,3,6-tetrahydropyridin-4-yl)acetonitrile (13c)

**Figure:**
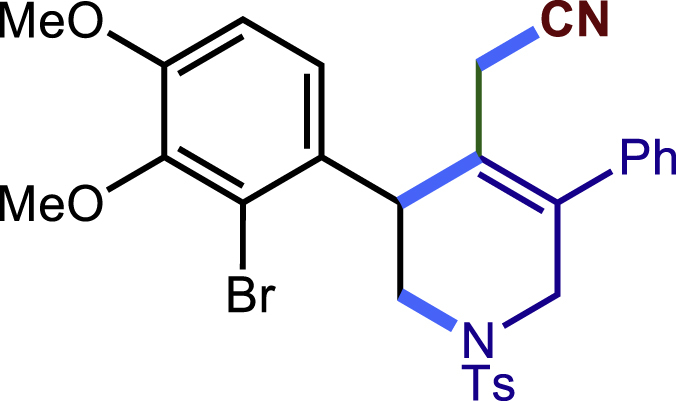

Prepared following general **Procedure A** using **1a** (63.4 mg, 0.2 mmol), **13b** (98 mg, 0.4 mmol). After 5 hours, the reaction mixture was purified by column chromatography to provide the compound **13c** (72.4 mg, 64% yield) as a white solid. Rf = 0.2 (Petroleum ether: EtOAc =4:1).

^1^H NMR (400 MHz, CDCl_3_) δ 7.59 (d, *J* = 8.2 Hz, 2H), 7.48 – 7.36 (m, 3H), 7.29 (d, *J* = 8.1 Hz, 2H), 7.25 – 7.21 (m, 2H), 7.06 (s, 1H), 6.93 (s, 1H), 4.24 (s, 2H), 3.88 (s, 6H), 3.71 (d, *J* = 11.2 Hz, 1H), 3.52 (d, *J* = 17.0 Hz, 1H), 3.18 (s, 1H), 2.93 (d, *J* = 17.1 Hz, 1H), 2.62 (d, *J* = 17.2 Hz, 1H), 2.42 (s, 3H).

^13^C NMR (101 MHz, CDCl_3_) δ 149.21, 148.65, 144.03, 137.37, 137.00, 133.06, 130.10, 129.87, 129.24, 128.82, 128.21, 127.53, 124.42, 117.36, 115.68, 114.77, 112.39, 56.20, 53.50, 49.49, 48.41, 42.56, 21.58, 20.51.

HRMS (ESI) for C_28_H_28_BrN_2_O_4_S[M+H]^+^m/z: calcd 567.0948, found 567.0933.

##### 2-(3-(naphthalen-2-yl)-5-phenyl-1-tosyl-1,2,3,6-tetrahydropyridin-4-yl)acetonitril-e (14c)

**Figure:**
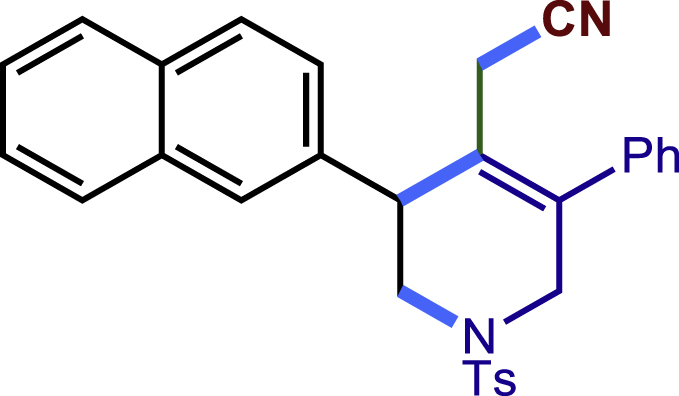

Prepared following general **Procedure A** using **1a** (63.4 mg, 0.2 mmol), **14b** (62 mg, 0.4 mmol). After 12 hours, the reaction mixture was purified by column chromatography to provide the compound **14c** (53.0 mg, 56% yield) as a white solid. Rf = 0.2 (Petroleum ether: EtOAc = 10:1).

^1^H NMR (400 MHz, Chloroform-d) δ 7.88 – 7.78 (m, 3H), 7.74 (s, 1H), 7.58 (d, *J* = 8.3 Hz, 2H), 7.54 – 7.37 (m, 6H), 7.28 (d, *J* = 6.6 Hz, 2H), 7.23 (d, *J* = 8.0 Hz, 2H), 4.14 (d, *J* = 16.8 Hz, 1H), 3.99 (s, 1H), 3.74 (d, *J* = 16.7 Hz, 1H), 3.56 (dd, *J* = 11.9, 4.5 Hz, 1H), 3.45 (dd, *J* = 11.9, 5.0 Hz, 1H), 3.02 (d, *J* = 17.2 Hz, 1H), 2.61 (d, *J* = 17.3 Hz, 1H), 2.39 (s, 3H).

^13^C NMR (101 MHz, Chloroform-d) δ 143.99, 137.22, 137.13, 136.74, 133.46, 132.99, 132.78, 129.79, 129.17, 129.03, 128.74, 128.39, 127.90, 127.78, 127.74, 127.71, 126.45, 126.21, 125.94, 124.22, 117.31, 49.88, 49.67, 44.21, 21.56, 20.49.

HRMS (ESI) for C_30_H_27_N_2_O_2_S[M+H]^+^m/z: calcd 479.1788, found 478.1812.

##### 2-(3-(benzo[b]thiophen-2-yl)-5-phenyl-1-tosyl-1,2,3,6-tetrahydropyridin-4-yl)acetonitrile (15c)

**Figure:**
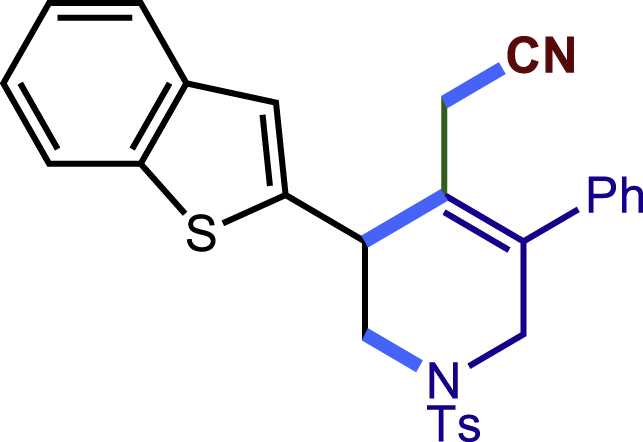

Prepared following general **Procedure A** using **1a** (63.4 mg, 0.2 mmol), **15b** (64 mg, 0.4 mmol). After 16 hours, the reaction mixture was purified by column chromatography to provide the compound **15c** (47.3 mg, 49% yield) as a yellow solid. Rf = 0.25 (Petroleum ether: EtOAc =10:1).

^1^H NMR (400 MHz, CDCl_3_) δ 7.80 (d, *J* = 7.6 Hz, 1H), 7.74 (dd, *J* = 7.0, 1.3 Hz, 1H), 7.63 (d, *J* = 8.3 Hz, 2H), 7.46 – 7.29 (m, 5H), 7.29-7.25 (m, 3H), 7.24 – 7.20 (m, 2H), 4.26 – 4.14 (m, 2H), 3.86 (dd, *J* = 11.7, 3.2 Hz, 1H), 3.55 (d, *J* = 16.7 Hz, 1H), 3.26 (dd, *J* = 11.8, 4.2 Hz, 1H), 3.05 (d, *J* = 17.4 Hz, 1H), 2.80 (d, *J* = 17.4 Hz, 1H), 2.40 (s, 3H).

^13^C NMR (101 MHz, CDCl_3_) δ 144.14, 143.26, 140.16, 139.17, 136.87, 136.80, 132.63, 129.85, 129.17, 128.83, 128.24, 127.77, 124.46, 124.41, 124.06, 123.47, 123.35, 122.45, 117.28, 49.52, 49.26, 40.17, 21.58, 20.39.

HRMS (ESI) for C_28_H_25_N_2_O_2_S_2_[M+H]^+^m/z: calcd 485.1352, found 485.1337.

##### 2-(3-(benzofuran-2-yl)-5-phenyl-1-tosyl-1,2,3,6-tetrahydropyridin-4-yl)acetonitril-e (16c)

**Figure:**
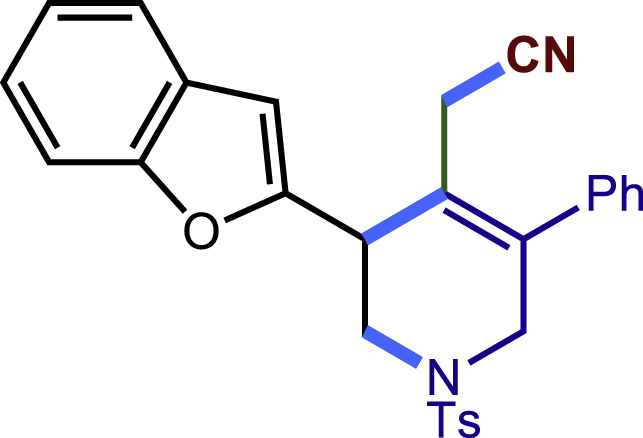

Prepared following general **Procedure A** using **1a** (63.4 mg, 0.2 mmol), **16b** (58 mg, 0.4 mmol). After 10 hours, the reaction mixture was purified by column chromatography to provide the compound **16c** (70.3 mg, 75% yield) as a white solid. Rf = 0.2 (Petroleum ether: EtOAc =10:1).

^1^H NMR (400 MHz, CDCl_3_) δ 7.62 (d, *J* = 8.1 Hz, 2H), 7.55 (d, *J* = 7.3 Hz, 1H), 7.48 – 7.36 (m, 4H), 7.31 – 7.20 (m, 6H), 6.75 (s, 1H), 4.04 (d, *J* = 17.9 Hz, 2H), 3.81 (dd, *J* = 11.9, 4.8 Hz, 1H), 3.71 (d, *J* = 16.6 Hz, 1H), 3.40 (dd, *J* = 12.0, 4.5 Hz, 1H), 3.10 (d, *J* = 17.3 Hz, 1H), 2.92 (d, *J* = 17.3 Hz, 1H), 2.40 (s, 3H).

^13^C NMR (101 MHz, CDCl_3_) δ 155.04, 154.87, 144.13, 137.73, 136.86, 132.79, 129.87, 129.18, 128.83, 128.23, 128.21, 127.68, 124.30, 123.01, 121.98, 121.12, 117.16, 111.16, 105.49, 49.40, 46.82, 38.23, 21.59, 20.78.

HRMS (ESI) for C_28_H_25_N_2_O_3_S[M+H]^+^m/z: calcd 469.1580, found 469.1537.

##### 2-(5-phenyl-1-tosyl-3-(1-tosyl-1H-indol-3-yl)-1,2,3,6-tetrahydropyridin-4-yl)acetonitrile (17c)

**Figure:**
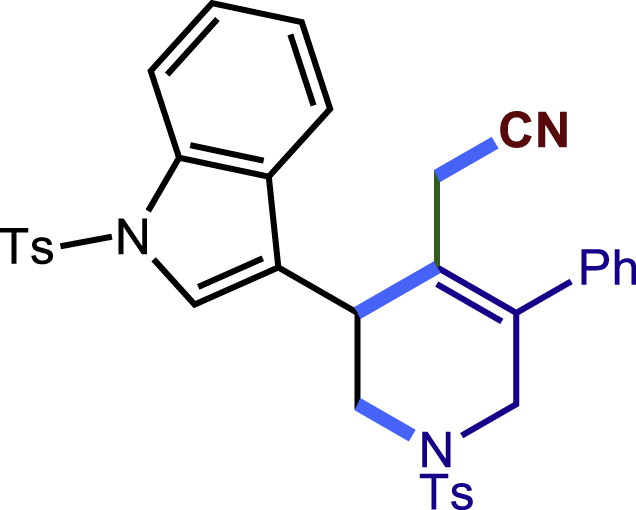

Prepared following general **Procedure A** using **1a** (63.4 mg, 0.2 mmol), **17b** (120 mg, 0.4 mmol). After 12 hours, the reaction mixture was purified by column chromatography to provide the compound **17c** (70.8 mg, 57% yield) as a white solid. Rf = 0.2 (Petroleum ether: EtOAc =5:1).

^1^H NMR (400 MHz, CDCl_3_) δ 7.97 (d, *J* = 8.2 Hz, 1H), 7.82 (d, *J* = 8.4 Hz, 2H), 7.64 – 7.51 (m, 4H), 7.49 – 7.38 (m, 3H), 7.33 (t, *J* = 7.3 Hz, 1H), 7.28 (d, *J* = 8.4 Hz, 5H), 7.25 (s, 2H), 4.25 (d, *J* = 16.7 Hz, 1H), 4.06 (s, 1H), 3.68 – 3.52 (m, 2H), 3.19 (dd, *J* = 11.7, 4.1 Hz, 1H), 3.03 (d, *J* = 17.3 Hz, 1H), 2.66 (d, *J* = 17.3 Hz, 1H), 2.41 (s, 3H), 2.32 (s, 3H).

^13^C NMR (101 MHz, CDCl_3_) δ 145.07, 144.11, 136.84, 136.73, 135.22, 134.79, 132.85, 130.12, 129.89, 129.57, 129.20, 128.87, 128.39, 127.61, 127.04, 125.53, 125.15, 123.78, 123.57, 120.33, 118.82, 117.45, 114.03, 49.51, 48.16, 34.94, 21.63, 21.59, 20.64.

HRMS (ESI) for C_35_H_32_N_3_O_4_S_2_[M+H]^+^m/z: calcd 622.1829, found 622.1769.

##### (E)-2-(3-(4-methoxystyryl)-5-phenyl-1-tosyl-1,2,3,6-tetrahydropyridin-4-yl)acetonitrile (18c)

**Figure:**
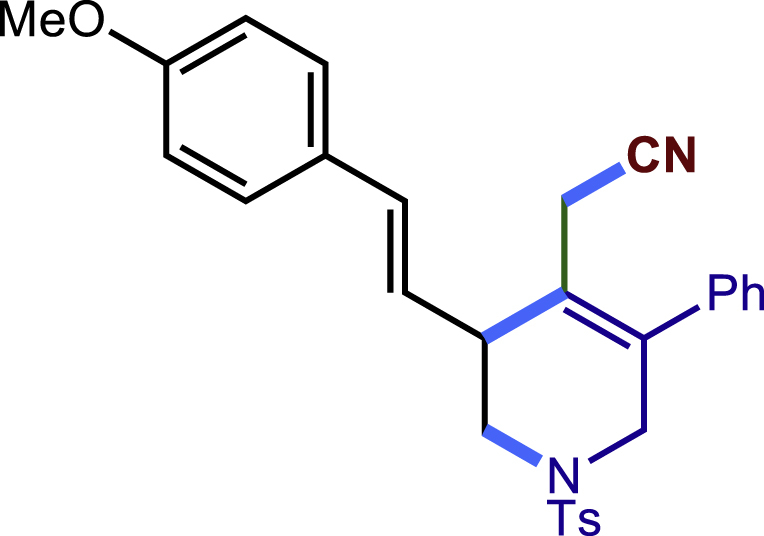

Prepared following general **Procedure A** using **1a** (63.4 mg, 0.2 mmol), **18b** (64 mg, 0.4 mmol). After 12 hours, the reaction mixture was purified by column chromatography to provide the compound **18c** (59.3 mg, 61% yield) as a white solid. Rf = 0.2 (Petroleum ether: EtOAc =5:1).

^1^H NMR (400 MHz, CDCl_3_) δ 7.66 (d, *J* = 8.2 Hz, 2H), 7.44 – 7.30 (m, 7H), 7.20 (d, *J* = 6.6 Hz, 2H), 6.87 (d, *J* = 8.7 Hz, 2H), 6.59 (d, *J* = 15.8 Hz, 1H), 6.08 (dd, *J =*15.8, 9.3 Hz, 1H), 4.03 (d, *J* = 16.6 Hz, 1H), 3.82 (s, 3H), 3.58 – 3.48 (m, 2H), 3.33 – 3.24 (m, 1H), 3.14 (dd, *J* = 11.6, 4.2 Hz, 1H), 2.96 (s, 2H), 2.43 (s, 3H).

^13^C NMR (101 MHz, CDCl_3_) δ 159.51, 144.03, 137.28, 135.94, 133.16, 132.67, 129.89, 129.12, 129.08, 128.62, 128.35, 127.83, 127.76, 124.94, 124.23, 117.77, 114.06, 55.37, 49.64, 48.13, 42.42, 21.59, 20.20.

HRMS (ESI) for C_29_H_29_N_2_O_3_S[M+H]^+^m/z: calcd 485.1893, found 485.1853.

##### N-(4-(cyanomethyl)-5-phenyl-1-tosyl-1,2,3,6-tetrahydropyridin-3-yl)-N-methylbenzamide (19c)

**Figure:**
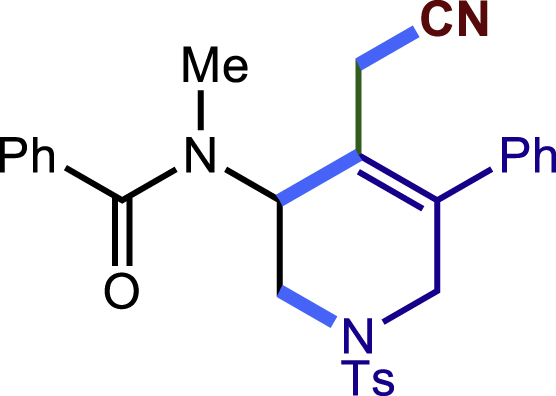

Prepared following general **Procedure A** using **1a** (63.4 mg, 0.2 mmol), **19b** (65 mg, 0.4 mmol). After 12 hours, the reaction mixture was purified by column chromatography to provide the compound **19c** (65.0 mg, 67% yield) as a yellow solid. Rf = 0.25 (Petroleum ether: EtOAc =1:1).

^1^H NMR (600 MHz, CDCl_3_) δ 7.66 (d, *J* = 8.1 Hz, 2H), 7.53 (dd, *J* = 6.4, 2.8 Hz, 2H), 7.47 – 7.38 (m, 6H), 7.35 (d, *J* = 8.1 Hz, 2H), 7.21 (d, *J* = 6.9 Hz, 2H), 5.47 (s, 1H), 4.21 (d, *J* = 16.7 Hz, 1H), 3.99 (d, *J* = 12.4 Hz, 1H), 3.36 (d, *J* = 16.6 Hz, 1H), 3.15 (s, 3H), 3.08 – 2.96 (m, 3H), 2.44 (s, 3H).

^13^C NMR (101 MHz, CDCl_3_) δ 172.92, 144.43, 141.27, 136.57, 135.72, 131.88, 130.06, 130.03, 129.29, 129.03, 128.56, 127.82 (d, *J* = 9.9 Hz), 127.09, 120.76, 117.12, 50.72, 49.54, 48.14, 35.15, 21.62, 19.88.

HRMS (ESI) for C_28_H_28_N_3_O_3_S[M+H]^+^m/z: calcd 486.1846, found 486.1833.

##### 2-(3-(2-oxopyrrolidin-1-yl)-5-phenyl-1-tosyl-1,2,3,6-tetrahydropyridin-4-yl)acetonitrile (20c)

**Figure:**
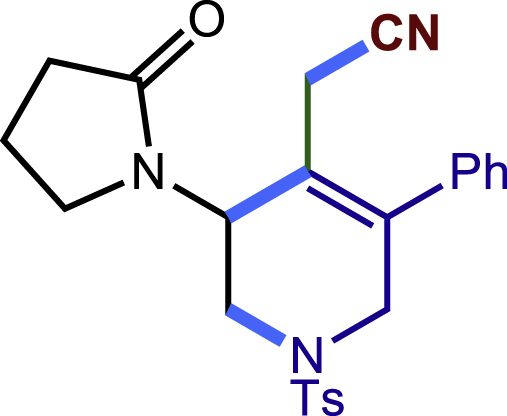

Prepared following general **Procedure A** using **1a** (63.4 mg, 0.2 mmol), **20b** (45 mg, 0.4 mmol). After 18 hours, the reaction mixture was purified by column chromatography to provide the compound **20c** (60.0 mg, 69% yield) as a yellow solid. Rf = 0.15 (Petroleum ether: EtOAc =1:1).

^1^H NMR (400 MHz, CDCl_3_) δ 7.61 (d, *J* = 8.2 Hz, 2H), 7.45 – 7.37 (m, 3H), 7.33 (d, *J* = 8.1 Hz, 2H), 7.20 – 7.15 (m, 2H), 4.91 (s, 1H), 4.20 (d, *J* = 16.7 Hz, 1H), 3.94 – 3.81 (m, 2H), 3.48 (ddd, *J* = 9.8, 7.8, 5.3 Hz, 1H), 3.24 (d, *J* = 16.6 Hz, 1H), 2.95 (d, *J* = 17.0 Hz, 1H), 2.85 – 2.75 (m, 2H), 2.47 (t, *J* = 8.1 Hz, 2H), 2.42 (s, 3H), 2.14 – 2.04 (m, 2H).

^13^C NMR (101 MHz, CDCl_3_) δ 176.07, 144.44, 140.77, 136.46, 131.75, 130.05, 129.26, 129.01, 127.82, 127.73, 120.58, 117.00, 49.49, 48.24, 48.05, 45.08, 30.94, 21.59, 19.71, 18.24.

HRMS (ESI) for C_24_H_26_N_3_O_3_S[M+H]^+^m/z: calcd 436.1689, found 436.1674.

##### 2-(3-(benzyloxy)-5-phenyl-1-tosyl-1,2,3,6-tetrahydropyridin-4-yl)acetonitrile (21c)

**Figure:**
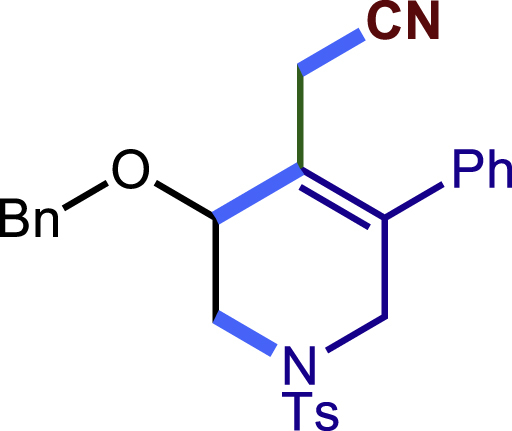

Prepared following general **Procedure A** using **1a** (63.4 mg, 0.2 mmol), **21b** (54 mg, 0.4 mmol). After 12 hours, the reaction mixture was purified by column chromatography to provide the compound **21c** (58.4 mg, 64% yield) as a yellow solid. Rf = 0.25 (Petroleum ether: EtOAc =5:1).

^1^H NMR (400 MHz, CDCl_3_) δ 7.67 (d, *J* = 6.0 Hz, 2H), 7.47 – 7.28 (m, 10H), 7.15 (d, *J* = 6.7 Hz, 2H), 4.79 (dd, *J* = 11.1, 2.1 Hz, 1H), 4.65 (dd, *J =*11.1, 2.2 Hz, 1H), 4.29 (s, 1H), 3.86 (d, *J* = 16.7 Hz, 1H), 3.66 (d, *J =*17.0 Hz, 1H), 3.56 – 3.45 (m, 1H), 3.37 (d, J = 12.0 Hz, 1H), 3.14 (d, *J* = 17.1 Hz, 1H), 2.93 (dd, *J* = *17*.1, 1.9 Hz, 1H), 2.43 (s, 1H).

^13^C NMR (101 MHz, CDCl_3_) δ 144.14, 138.54, 137.25, 136.44, 132.96, 129.93, 129.10, 128.86, 128.70, 128.43, 128.27, 128.10, 127.72, 123.52, 117.56, 72.25, 71.54, 49.46, 46.09, 21.60, 18.76.

HRMS (ESI) for C_27_H_27_N_2_O_3_S[M+H]^+^m/z: calcd 459.1737, found 459.1671.

##### 2-(3-(4-methoxyphenyl)-5-(p-tolyl)-1-tosyl-1,2,3,6-tetrahydropyridin-4-yl)acetonitrile (22c)

**Figure:**
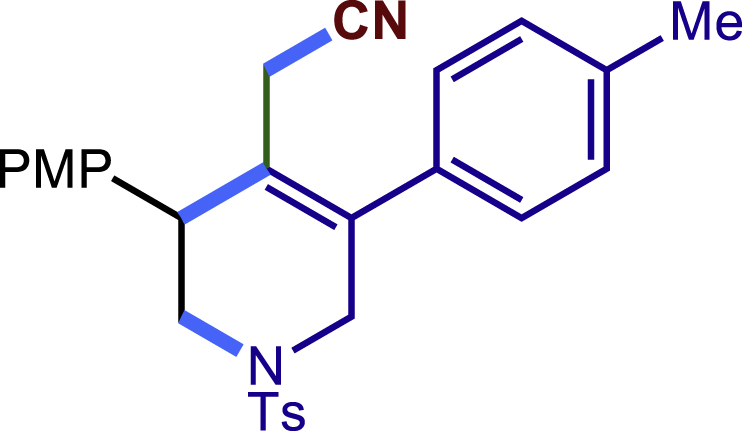

Prepared following general **Procedure A** using N-F (66.2 mg, 0.2 mmol). After 12 hours, the reaction mixture was purified by column chromatography to provide the compound **22c** (64.3 mg, 68% yield) as a white solid. Rf = 0.25 (Petroleum ether: EtOAc = 5:1).

^1^H NMR (400 MHz, CDCl_3_) δ 7.62 (d, *J* = 8.2 Hz, 2H), 7.31 (d, *J* = 8.1 Hz, 2H), 7.28 – 7.22 (m, 4H), 7.13 (d, *J* = 8.0 Hz, 2H), 6.91 (d, *J* = 8.6 Hz, 2H), 4.06 (d, *J* = 16.6 Hz, 1H), 3.83 (s, 3H), 3.76 (s, 1H), 3.64 (d, J = 16.6 Hz, 1H), 3.45 (dd, *J* = 11.7, 4.5 Hz, 1H), 3.33 (dd, *J* = 11.7, 4.9 Hz, 1H), 3.00 (d, *J* = 17.2 Hz, 1H), 2.61 (d, *J* = 17.2 Hz, 1H), 2.44 (s, 3H), 2.40 (s, 3H).

^13^C NMR (101 MHz, CDCl3) δ 159.22, 143.193, 138.60, 136.40, 134.27, 132.77, 131.35, 129.80, 129.74, 129.58, 128.27, 127.73, 124.41, 117.45, 114.193, 55.30, 50.08, 49.71, 43.34, 21.56, 21.23, 20.193.

HRMS (ESI) for C_28_H_29_N_2_O_3_S[M+H]^+^m/z: calcd 473.1893, found 473.1864.

##### 2-(3,5-bis(4-methoxyphenyl)-1-tosyl-1,2,3,6-tetrahydropyridin-4-yl)acetonitrile (23c)

**Figure:**
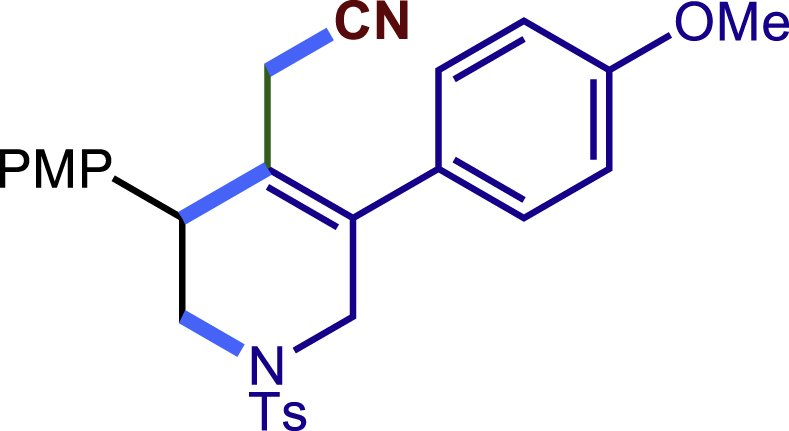

Prepared following general **Procedure A** using N-F (69.4 mg, 0.2 mmol). After 12 hours, the reaction mixture was purified by column chromatography to provide the compound **23c** (62.5 mg, 64% yield) as a yellow solid. Rf = 0.2 (Petroleum ether: EtOAc =5:1).

^1^H NMR (400 MHz, CDCl_3_) δ 7.60 (d, *J* = 8.1 Hz, 2H), 7.29 (d, *J* = 8.0 Hz, 2H), 7.23 (d, *J* = 8.5 Hz, 2H), 7.15 (d, *J* = 8.5 Hz, 2H), 6.93 (d, *J* = 8.5 Hz, 2H), 6.88 (d, *J* = 8.5 Hz, 2H), 4.03 (d, *J* = 16.6 Hz, 1H), 3.83 (s, 3H), 3.81 (s, 3H), 3.73 (s, 1H), 3.60 (d, *J* = 16.7 Hz, 1H), 3.42 (dd, *J* = 11.7, 4.4 Hz, 1H), 3.29 (dd, *J* = 11.7, 4.8 Hz, 1H), 2.98 (d, *J* = 17.2 Hz, 1H), 2.59 (d, *J* = 17.2 Hz, 1H), 2.42 (s, 3H).

^13^C NMR (101 MHz, CDCl_3_) δ 159.73, 159.20, 143.94, 136.04, 132.69, 131.37, 129.82, 129.67, 129.58, 129.29, 127.74, 124.45, 117.54, 114.44, 114.38, 55.40, 55.31, 50.07, 49.75, 43.39, 21.58, 20.40.

HRMS (ESI) for C_28_H_29_N_2_O_4_S[M+H]^+^m/z: calcd 489.1843, found 489.1848.

##### 2-(5-(4-fluorophenyl)-3-(4-methoxyphenyl)-1-tosyl-1,2,3,6-tetrahydropyridin-4-yl)acetonitrile (24c)

**Figure:**
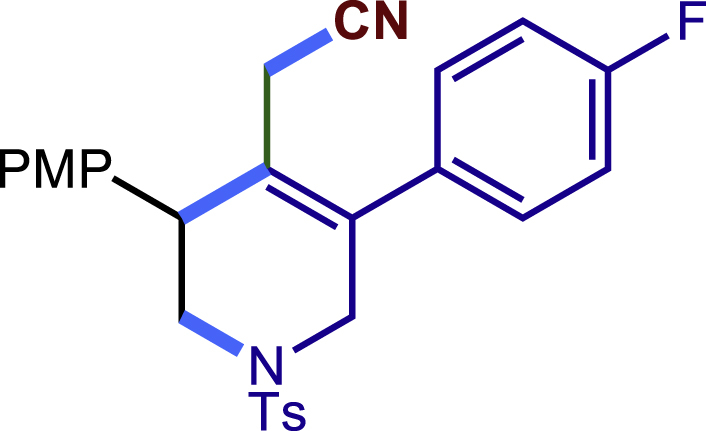

Prepared following general **Procedure A** using substrate (67.0 mg, 0.2 mmol). After 12 hours, the reaction mixture was purified by column chromatography to provide the compound **24c** (43.0 mg, 45% yield) as a white solid. Rf = 0.2 (Petroleum ether: EtOAc = 8:1).

^1^H NMR (400 MHz, CDCl_3_) δ 7.60 (d, *J* = 8.1 Hz, 2H), 7.30 (d, *J* = 8.0 Hz, 2H), 7.25 – 7.17 (m, 4H), 7.11 (t, *J* = 8.5 Hz, 2H), 6.89 (d, *J* = 8.5 Hz, 2H), 4.02 (d, *J* = 16.7 Hz, 1H), 3.81 (s, 3H), 3.74 (s, 1H), 3.61 (d, *J* = 16.6 Hz, 1H), 3.43 (dd*, J* = 11.8, 4.4 Hz, 1H), 3.31 (dd, J = 11.7, 4.8 Hz, 1H), 2.91 (d, J = 17.2 Hz, 1H), 2.60 (d, J = 17.2 Hz, 1H), 2.42 (s, 3H).

^13^C NMR (101 MHz, CDCl_3_) δ 162.69 (d, *J* = 248.7 Hz), 159.29, 144.02, 135.51, 133.14 (d, *J* = 3.5 Hz), 132.72, 131.08, 130.24 (d, *J =*8.3 Hz), 129.85, 129.54, 127.72, 125.38, 117.19, 116.22 (d, *J* = 21.7 Hz), 114.46, 55.30, 50.01, 49.69, 43.40, 21.57, 20.34.

19F NMR (377 MHz, CDCl_3_) δ -112.22 – -112.37 (m).

HRMS (ESI) for C_27_H_26_FN_2_O_3_S[M+H]^+^m/z: calcd 477.1643, found 477.1607.

##### 2-(5-(4-chlorophenyl)-3-(4-methoxyphenyl)-1-tosyl-1,2,3,6-tetrahydropyridin-4-yl)acetonitrile (25c)

**Figure:**
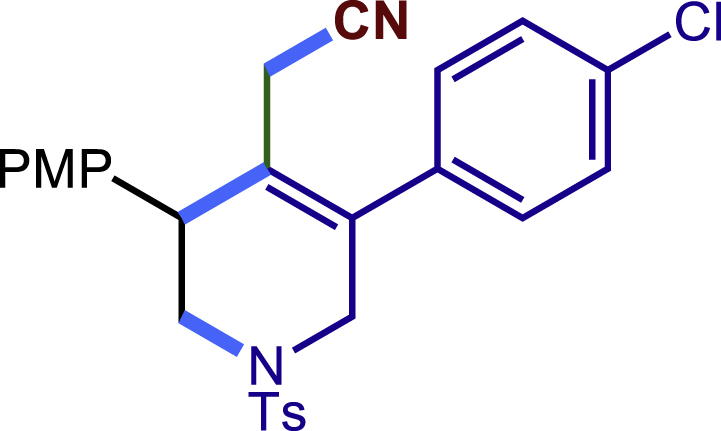

Prepared following general **Procedure A** using N-F (70.4 mg, 0.2 mmol). After 12 hours, the reaction mixture was purified by column chromatography to provide the compound **25c** (56.1 mg, 57% yield) as a white solid. Rf = 0.2 (Petroleum ether: EtOAc = 5:1).

^1^H NMR (400 MHz, CDCl_3_) δ 7.60 (d, *J* = 8.2 Hz, 2H), 7.39 (d, *J* = 8.3 Hz, 2H), 7.30 (d, *J* = 8.1 Hz, 2H), 7.22 (d, *J* = 8.6 Hz, 2H), 7.17 (d, *J* = 8.3 Hz, 2H), 6.88 (d, *J* = 8.6 Hz, 2H), 4.00 (d, *J* = 16.6 Hz, 1H), 3.80 (s, 3H), 3.74 (s, 1H), 3.61 (d, *J* = 16.6 Hz, 1H), 3.41 (dd, *J* = 11.8, 4.5 Hz, 1H), 3.32 (dd, *J* = 11.8, 4.9 Hz, 1H), 2.90 (d, *J* = 17.2 Hz, 1H), 2.61 (d, *J* = 17.3 Hz, 1H), 2.42 (s, 3H).

^13^C NMR (101 MHz, CDCl_3_) δ 159.29, 144.08, 135.61, 135.38, 134.76, 132.64, 131.00, 129.88, 129.81, 129.56, 129.40, 127.72, 125.52, 117.12, 114.47, 55.31, 50.01, 49.54, 43.40, 21.59, 20.35.

HRMS (ESI) for C_27_H_26_ClN_2_O_3_S[M+H]^+^m/z: calcd 493.1347, found 493.1323.

##### ethyl-4-(4-(cyanomethyl)-5-(4-methoxyphenyl)-1-tosyl-1,2,5,6-tetrahydropyridin-3-yl)benzoate (26c)

**Figure:**
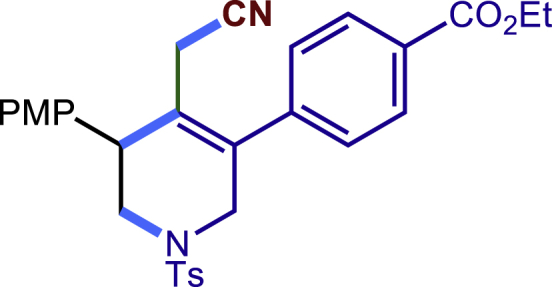

Prepared following general **Procedure A** using N-F (77.8 mg, 0.2 mmol). After 12 hours, the reaction mixture was purified by column chromatography to provide the compound **26c** (54.1 mg, 51% yield) as a white solid. Rf = 0.2 (Petroleum ether: EtOAc = 8:1).

^1^H NMR (600 MHz, CDCl_3_) δ 8.09 (d, *J* = 8.2 Hz, 2H), 7.59 (t, *J* = 9.3 Hz, 2H), 7.30 (d, *J* = 8.1 Hz, 4H), 7.23 (d, *J* = 8.6 Hz, 2H), 6.89 (d, *J* = 8.6 Hz, 2H), 4.39 (q, *J* = 7.1 Hz, 2H), 4.04 (d, *J* = 16.7 Hz, 1H), 3.81 (s, 3H), 3.76 (s, 1H), 3.64 (d, *J* = 16.7 Hz, 1H), 3.43 (dd, *J* = 11.8, 4.5 Hz, 1H), 3.34 (dd, *J* = 11.8, 4.9 Hz, 1H), 2.88 (d, *J* = 17.3 Hz, 1H), 2.61 (d, *J* = 17.4 Hz, 1H), 2.42 (s, 3H), 1.40 (t, *J* = 7.1 Hz, 3H).

^13^C NMR (151 MHz, CDCl_3_) δ 165.85, 159.30, 144.07, 141.74, 135.70, 132.68, 130.93, 130.80, 130.31, 129.86, 129.54, 128.46, 127.70, 125.63, 116.96, 114.48, 61.30, 55.30, 50.00, 49.35, 43.36, 21.56, 20.34, 14.33.

HRMS (ESI) for C_30_H_31_N_2_O_5_S[M+H]^+^m/z: calcd 531.1948, found 531.1917.

##### 2-(3-(4-methoxyphenyl)-5-(m-tolyl)-1-tosyl-1,2,3,6-tetrahydropyridin-4-yl)acetonitrile (27c)

**Figure:**
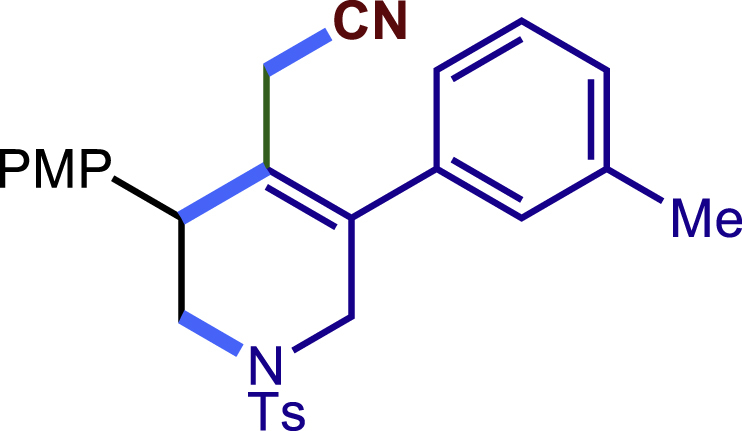

Prepared following general **Procedure A** using N-F (66.2 mg, 0.2 mmol). After 12 hours, the reaction mixture was purified by column chromatography to provide the compound **27c** (72.8 mg, 77% yield) as a white solid. Rf = 0.25 (Petroleum ether: EtOAc = 5:1).

^1^H NMR (400 MHz, CDCl_3_) δ 7.60 (d*, J* = 8.2 Hz, 2H), 7.32 – 7.27 (m, 3H), 7.24 (d, *J* = 8.7 Hz, 2H), 7.18 (d, *J* = 7.6 Hz, 1H), 7.03 (s, 1H), 7.00 (d, *J* = 7.6 Hz, 1H), 6.89 (d, *J* = 8.7 Hz, 2H), 4.04 (d, *J* = 16.6 Hz, 1H), 3.81 (s, 3H), 3.75 (s, 1H), 3.62 (d, *J* = 16.6 Hz, 1H), 3.43 (dd, *J* = 11.7, 4.5 Hz, 1H), 3.31 (dd, *J* = 11.7, 4.9 Hz, 1H), 2.97 (d, *J* = 17.2 Hz, 1H), 2.58 (d, *J* = 17.2 Hz, 1H), 2.42 (s, 3H), 2.38 (s, 3H).

^13^C NMR (101 MHz, CDCl_3_) δ 159.22, 143.93, 138.95, 137.24, 136.58, 132.77, 131.33, 129.81, 129.57, 129.35, 128.96, 128.94, 127.74, 125.42, 124.40, 117.42, 114.41, 55.30, 50.09, 49.70, 43.28, 21.56, 21.39, 20.37.

HRMS (ESI) for C_28_H_29_N_2_O_3_S[M+H]^+^m/z: calcd 473.1893, found 473.1862.

##### 2-(3-(4-methoxyphenyl)-5-(o-tolyl)-1-tosyl-1,2,3,6-tetrahydropyridin-4-yl)acetonitrile (28c)

**Figure:**
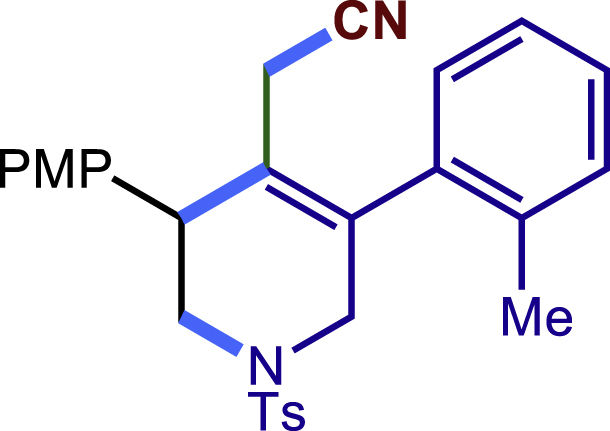

Prepared following general **Procedure A** using N-F (66.2 mg, 0.2 mmol). After 12 hours, the reaction mixture was purified by column chromatography to provide the compound **28c** (59.6 mg, 63% yield) as a white solid. Rf = 0.25 (Petroleum ether: EtOAc = 5:1).

^1^H NMR (400 MHz, Chloroform-d) major: δ 7.66 – 7.59 (t, 2H), 7.36 – 7.21 (m, 7H), 7.10 (d, *J* = 7.3 Hz, 1H), 6.93 (d, *J* = 3.6 Hz, 2H), 3.98 (d, *J* = 16.5 Hz, 1H), 3.89 – 3.80 (m, 4H), 3.75 (s, 1H), 3.58 – 3.53 (m, 1H), 3.34 (d, *J* = 5.2 Hz, 1H), 2.73 (d, *J* = 17.1 Hz, 1H), 2.57 (s, 1H), 2.45 (s, 3H), 2.27 (s, 3H). minor: 1H NMR (400 MHz, Chloroform-d) δ 7.65 – 7.53 (t, 2H), 7.34 – 7.16 (m, 7H), 7.02 (d, *J* = 7.4 Hz, 1H), 6.89 (d, *J* = 3.6 Hz, 2H), 3.88 – 3.77 (m, 5H), 3.66 (d, *J* = 16.8 Hz, 1H), 3.49 – 3.44 (m, 1H), 3.29 (d, *J* = 5.1 Hz, 1H), 2.77 (d, *J* = 17.3 Hz, 1H), 2.50 (s, 1H), 2.42 (s, 3H), 2.26 (s, 3H).

^13^C NMR (101 MHz, Chloroform-d) δ 159.25, 159.23, 143.96, 143.88, 136.51, 136.45, 136.23, 135.93, 135.76, 135.43, 132.92, 132.86, 131.62, 131.06, 130.87, 130.72, 129.82, 129.79, 129.56, 129.53, 128.76, 128.72, 128.64, 127.72, 126.70, 126.46, 125.09, 125.03, 116.97, 116.78, 114.47, 55.30, 50.28, 50.09, 49.17, 43.24, 43.16, 21.57, 19.94, 19.89, 19.36, 19.20.

HRMS (ESI) for C_28_H_29_N_2_O_3_S[M+H]^+^m/z: calcd 473.1893, found 473.1862.

##### 2-(3-(4-methoxyphenyl)-5-(naphthalen-1-yl)-1-tosyl-1,2,3,6-tetrahydropyridin-4-yl)acetonitrile (29c)

**Figure:**
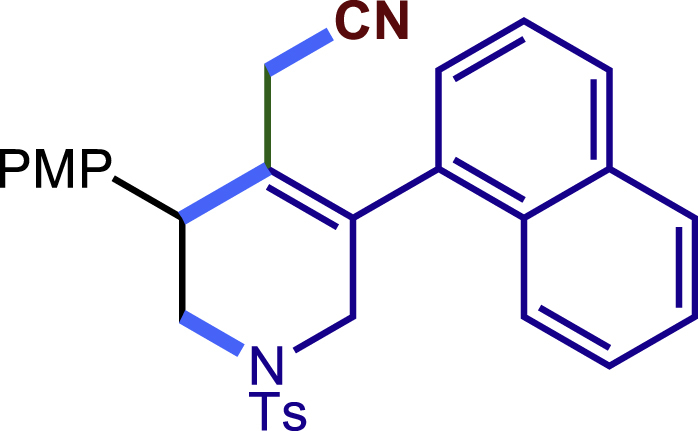

Prepared following general **Procedure A** using N-F (73.4 mg, 0.2 mmol). After 12 hours, the reaction mixture was purified by column chromatography to provide the compound **29c** (60 mg, 59% yield) as a white solid. Rf = 0.2 (Petroleum ether: EtOAc = 5:1).

^1^H NMR (600 MHz, Chloroform-d) major: δ 7.94 – 7.84 (m, 2H), 7.73 (d, *J* = 8.1 Hz, 1H), 7.62 – 7.41 (m, 6H), 7.36 – 7.27 (m, 4H), 6.97 (d, *J* = 8.6 Hz, 2H), 4.19 (d, *J* = 16.7 Hz, 1H), 3.94 (d, *J* = 17.0 Hz, 1H), 3.84 (s, 3H), 3.69 – 3.61 (m, 1H), 3.59 (d, *J* = 16.7 Hz, 1H), 3.39 – 3.33 (m, 1H), 2.69 (d, *J* = 3.6 Hz, 1H), 2.51 (d, J = 17.3 Hz, 1H), 2.43 (s, 3H). minor: δ 7.94 – 7.84 (m, 2H), 7.78 (d, *J* = 9.5 Hz, 1H), 7.62 – 7.41 (m, 6H), 7.36 – 7.27 (m, 4H), 6.92 (d, *J* = 8.6 Hz, 2H), 4.00 (s, 1H), 3.87 (s, 1H), 3.82 (s, 4H), 3.69 – 3.61 (m, 1H), 3.39 – 3.33 (m, 1H), 2.72 (d, *J* = 3.6 Hz, 1H), 2.56 (d, *J =*17.3 Hz, 1H), 2.42 (s, 3H).

^13^C NMR (151 MHz, Chloroform-d) major: δ 159.29, 143.99, 134.70, 134.40, 133.85, 131.72, 130.65, 129.85, 129.59, 129.08, 129.00, 128.61, 127.75, 127.38, 126.70, 126.65, 125.69, 125.40, 123.87, 117.15, 114.58, 55.34, 50.21, 49.81, 43.29, 21.57, 20.29. minor: 159.29, 143.90, 134.98, 134.59, 133.81, 130.95, 130.58, 129.85, 129.67, 129.05, 128.76, 128.03, 127.72, 127.50, 126.77, 126.50, 125.71, 125.40, 124.33, 116.78, 114.51, 55.32, 50.44, 49.85, 43.52, 21.57, 20.40.

HRMS (ESI) for C_31_H_29_N_2_O_3_S[M+H]^+^m/z: calcd 509.1893, found 509.1858.

##### 2-(3-(4-methoxyphenyl)-5-(naphthalen-2-yl)-1-tosyl-1,2,3,6-tetrahydropyridin-4-yl)acetonitrile (30c)

**Figure:**
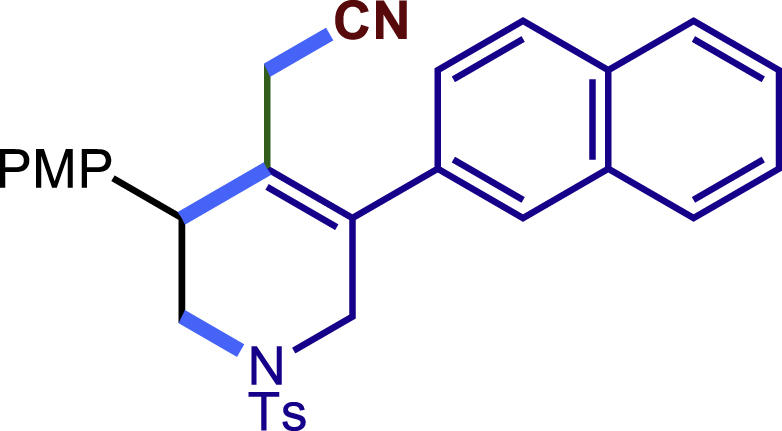

Prepared following general **Procedure A** using N-F (73.4 mg, 0.2 mmol). After 12 hours, the reaction mixture was purified by column chromatography to provide the compound **30c** (64.0 mg, 63% yield) as a white solid. Rf = 0.2 (Petroleum ether: EtOAc = 5:1).

^1^H NMR (400 MHz, CDCl_3_) δ 7.95 – 7.82 (m, 3H), 7.71 (s, 1H), 7.62 (d, *J* = 8.0 Hz, 2H), 7.58 – 7.51 (m, 2H), 7.35 – 7.27 (m, 5H), 6.92 (d, *J =*8.4 Hz, 2H), 4.16 (d, *J =*16.6 Hz, 1H), 3.82 (s, 4H), 3.73 (d, *J* = 16.7 Hz, 1H), 3.48 (dd, *J* = 11.7, 4.4 Hz, 1H), 3.37 (dd, *J =*11.7, 4.9 Hz, 1H), 3.01 (d, *J* = 17.2 Hz, 1H), 2.64 (d, 1H), 2.42 (s, 3H).

^13^C NMR (101 MHz, CDCl_3_) δ 159.27, 144.01, 136.45, 134.58, 133.19, 132.97, 132.69, 131.28, 129.86, 129.63, 129.03, 128.01, 127.83, 127.76, 127.70, 126.94, 126.90, 125.87, 125.12, 117.37, 114.46, 55.33, 50.15, 49.76, 43.41, 21.58, 20.48.

HRMS (ESI) for C_31_H_29_N_2_O_3_S[M+H]^+^m/z: calcd 509.1893, found 509.1856.

##### 4-(azidomethyl)-3,5-diphenyl-1-tosyl-1,2,3,6-tetrahydropyridine (32c)

**Figure:**
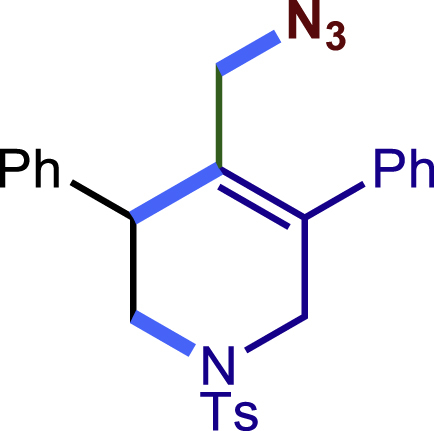

^1^H NMR (400 MHz, CDCl3) δ 7.60 (d, *J =*8.2 Hz, 2H), 7.44 – 7.27 (m, 10H), 7.21 (d, *J =*6.6 Hz, 2H), 4.04 (d, *J =*16.7 Hz, 1H), 3.85 – 3.75 (m, 2H), 3.70 (d, *J* = 16.7 Hz, 1H), 3.44 (dd, *J =*11.7, 4.6 Hz, 1H), 3.33 (dd, *J* = 11.7, 4.8 Hz, 1H), 3.22 (d, *J* = 13.1 Hz, 1H), 2.42 (s, 3H).

^13^C NMR (101 MHz, CDCl3) δ 143.85, 140.16, 137.50, 136.74, 132.74, 129.80, 129.46, 128.75 (t, J = 12.1 Hz), 128.31, 127.77, 127.52, 50.57, 49.94, 49.62, 42.82, 21.60.

HRMS (ESI) for C_25_H_25_N_4_O_2_S[M+H]^+^m/z: calcd 445.1693, found 445.1671.

##### 3-(4-methoxyphenyl)-5-phenyl-4-(thiocyanatomethyl)-1-tosyl-1,2,3,6-tetrahydropyridine (33c)

**Figure:**
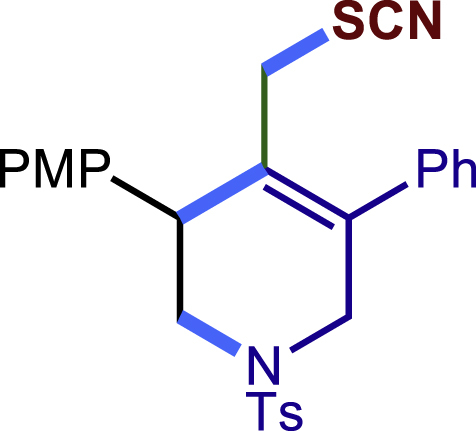

^1^H NMR (400 MHz, CDCl_3_) δ 7.60 (d, *J =*8.1 Hz, 2H), 7.45 – 7.34 (m, 3H), 7.30 (d, *J* = 8.0 Hz, 2H), 7.24 (d, *J* = 8.1 Hz, 4H), 6.89 (d, *J =*8.5 Hz, 2H), 4.01 (d, *J* = 16.9 Hz, 1H), 3.91 (s, 1H), 3.81 (s, 3H), 3.76 – 3.68 (m, 2H), 3.42 (dd, *J =*11.7, 4.7 Hz, 1H), 3.35 (dd, *J* = 11.7, 4.8 Hz, 1H), 2.92 (d, J = 12.7 Hz, 1H), 2.43 (s, 3H).

^13^C NMR (101 MHz, CDCl_3_) δ 159.17, 143.99, 138.78, 137.03, 132.67, 131.47, 129.89, 129.62, 129.01, 128.78, 128.54, 128.17, 127.73, 114.44, 111.72, 55.33, 50.08, 41.57, 34.74, 21.61.

HRMS (ESI) for C_27_H_27_N_2_O_3_S_2_[M+H]^+^m/z: calcd 491.1458, found 491.1413.

##### 3-(4-methoxyphenyl)-5-phenyl-1-tosyl-4-(((trifluoromethyl)thio)methyl)-1,2,3,6-tetrahydropyridine (34c)

**Figure:**
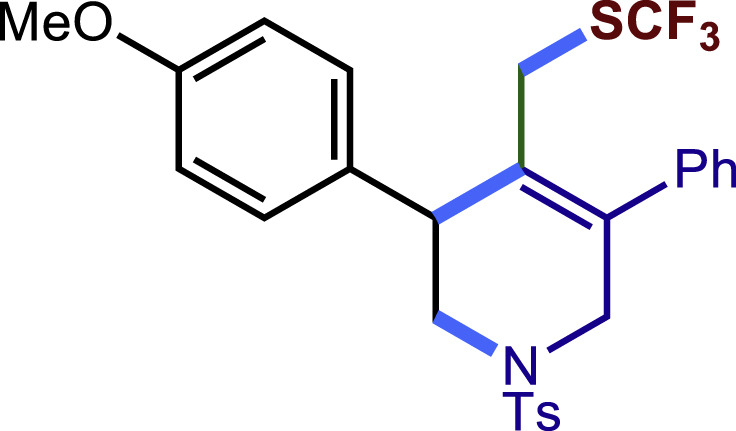

^1^H NMR (400 MHz, CDCl_3_) δ 7.59 (d, *J* = 8.2 Hz, 2H), 7.43 – 7.32 (m, 3H), 7.29 (d, *J* = 8.1 Hz, 2H), 7.22 (d, *J* = 8.6 Hz, 2H), 7.21 – 7.16 (m, 2H), 6.88 (d, *J* = 8.6 Hz, 2H), 3.98 (d, *J* = 16.7 Hz, 1H), 3.89 (s, 1H), 3.81 (s, 3H), 3.67 (d, *J* = 16.7 Hz, 1H), 3.57 (d, *J* = 12.8 Hz, 1H), 3.34 (ddd, *J* = 28.9, 11.7, 4.9 Hz, 2H), 2.91 (d, *J* = 12.9 Hz, 1H), 2.42 (s, 3H).

^13^C NMR (101 MHz, CDCl_3_) δ 159.03, 143.81, 137.62, 136.39, 132.93, 131.89, 129.77, 129.62, 128.79, 128.63, 128.42, 128.32, 127.74, 114.27, 55.27, 50.17, 49.90, 41.78, 30.84, 21.55.

19F NMR (377 MHz, CDCl3) δ -41.21 (s).

HRMS (ESI) for C_27_H_27_F_3_NO_3_S_2_[M+H]^+^m/z: calcd 534.1379, found 534.1383.

##### 4-(4,4-dimethylpent-2-yn-1-yl)-3-(4-methoxyphenyl)-5-phenyl-1-tosyl-1,2,3,6-tetrahydropyridine (35c)

**Figure:**
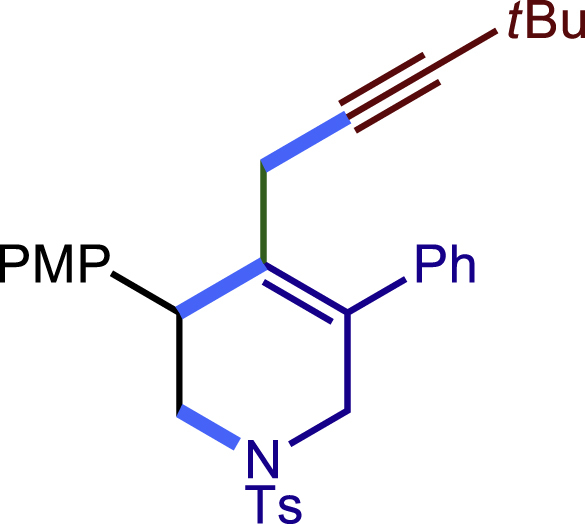

Prepared following general **Procedure C** using **1a** (63.4 mg, 0.2 mmol), 4-methoxystyrene (54 mg, 0.4 mmol, 2.0 eq.), and (3,3-dimethylbut-1-yn-1-yl)trimethoxysilane (81 mg, 0.4 mmol, 2.0 eq.). After 12 hours, the reaction mixture was purified by column chromatography to provide the title compound 35c (44.5 mg, 43% yield) as a white solid. Rf = 0.3 (Petroleum ether: EtOAc =5:1)

^1^H NMR (400 MHz, CDCl_3_) δ 7.60 (d, *J* = 8.2 Hz, 2H), 7.39 – 7.27 (m, 5H), 7.26 – 7.22 (m, 4H), 6.86 (d, *J* = 8.7 Hz, 2H), 3.99 (d, *J* = 15.9 Hz, 1H), 3.90 (s, 1H), 3.81 (s, 3H), 3.59 (d, *J* = 15.9 Hz, 1H), 3.38 (dd, *J* = 11.4, 4.5 Hz, 1H), 3.26 (dd, *J =*11.4, 4.8 Hz, 1H), 2.80 (d, *J* = 17.1 Hz, 1H), 2.42 – 2.38 (m, 4H), 1.16 (s, 9H).

^13^C NMR (101 MHz, CDCl_3_) δ 158.69, 143.58, 138.72, 133.04, 132.83, 131.20, 131.04, 129.71, 129.67, 128.80, 128.52, 127.85, 127.71, 113.89, 89.85, 75.55, 55.25, 50.43, 49.49, 42.74, 31.25, 27.37, 21.58, 21.55.

HRMS (ESI) for C_32_H_36_NO_3_S[M+H]^+^m/z: calcd 514.2410, found 514.2342.

##### 4-(hept-2-yn-1-yl)-3-(4-methoxyphenyl)-5-phenyl-1-tosyl-1,2,3,6-tetrahydropyridine (36c)

**Figure:**
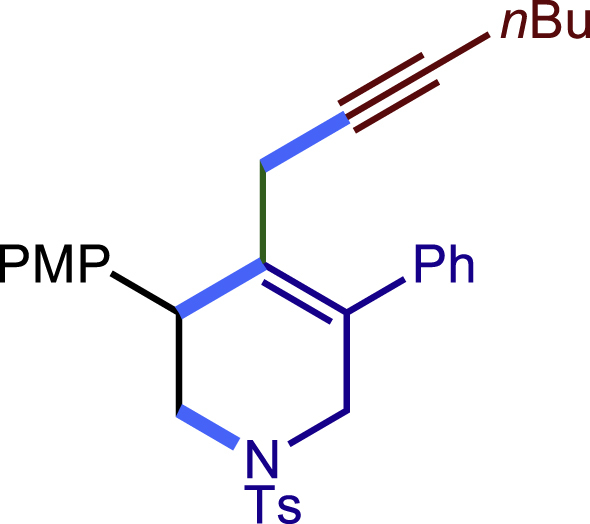

Prepared following general **Procedure C** using **1a** (63.4 mg, 0.2 mmol), 4-methoxystyrene (54 mg, 0.4 mmol, 2.0 eq.), and hex-1-yn-1-yltrimethoxysilane (81 mg, 0.4 mmol, 2.0 eq.). After 12 hours, the reaction mixture was purified by column chromatography to provide the title compound **36c** (63.7 mg, 62% yield) as a white solid. Rf = 0.3 (Petroleum ether: EtOAc =5:1)

^1^H NMR (400 MHz, CDCl_3_) δ 7.62 (d, *J =*8.1 Hz, 2H), 7.42 – 7.32 (m, 3H), 7.31 – 7.26 (m, 6H), 6.89 (d, *J =*8.5 Hz, 2H), 4.08 (d, *J =*16.0 Hz, 1H), 3.93 (s, 1H), 3.83 (s, 3H), 3.56 (d, *J =*16.0 Hz, 1H), 3.48 (dd, *J =*11.4, 3.9 Hz, 1H), 3.23 (dd, *J* = 11.4, 4.7 Hz, 1H), 2.83 (d, *J* = 17.0 Hz, 1H), 2.43 – 2.39 (m, 4H), 2.13 (t, *J =*6.7 Hz, 2H), 1.52 – 1.35 (m, 4H), 0.94 (t, *J* = 7.0 Hz, 3H).

^13^C NMR (101 MHz, CDCl_3_) δ 158.68, 143.58, 138.66, 133.09, 132.78, 131.21, 130.96, 129.65, 128.78, 128.54, 127.84, 127.75, 113.90, 81.27, 77.21, 55.25, 50.29, 49.41, 42.57, 31.09, 21.99, 21.60, 21.55, 18.48, 13.67.

HRMS (ESI) for C_32_H_36_NO_3_S[M+H]^+^m/z: calcd 514.2410, found 514.2338.

##### 3-(4-methoxyphenyl)-5-phenyl-4-(3-phenylprop-2-yn-1-yl)-1-tosyl-1,2,3,6-tetrahydropyridine (37c)

**Figure:**
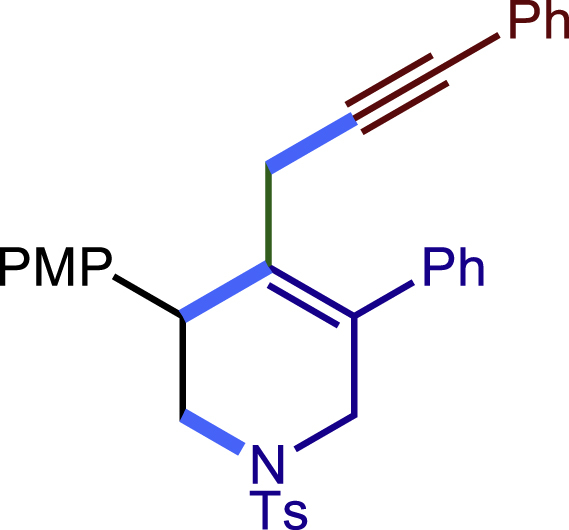

Prepared following general **Procedure C** using **1a** (63.4 mg, 0.2 mmol), 4-methoxystyrene (54 mg, 0.4 mmol, 2.0 eq.), and trimethoxy(phenylethynyl)silane (90 mg, 0.4 mmol, 2.0 eq.). After 12 hours, the reaction mixture was purified by column chromatography using 7% EtOAc in PE to provide the title compound **37c** (81.2 mg, 76% yield) as a white solid. Rf = 0.25 (Petroleum ether: EtOAc =6:1)

^1^H NMR (400 MHz, CDCl_3_) δ 7.61 (d, *J* = 8.2 Hz, 2H), 7.43 – 7.26 (m, 14H), 6.89 (d, *J =*8.6 Hz, 2H), 4.07 (d, *J* = 16.1 Hz, 1H), 3.98 (s, 1H), 3.81 (s, 3H), 3.62 (d, *J* = 16.1 Hz, 1H), 3.46 (dd, *J* = 11.5, 4.3 Hz, 1H), 3.29 (dd, *J =*11.5, 4.8 Hz, 1H), 3.07 (d, *J* = 17.3 Hz, 1H), 2.68 (d, *J* = 17.4 Hz, 1H), 2.40 (s, 3H).

^13^C NMR (101 MHz, CDCl_3_) δ 158.80, 143.66, 138.54, 132.86, 132.79, 132.13, 131.58, 130.22, 129.71 (d, J = 1.8 Hz), 128.80, 128.67, 128.24, 127.92, 127.83, 127.80, 123.67, 114.02, 87.35, 81.42, 55.28, 50.37, 49.55, 42.90, 22.33, 21.55.

HRMS (ESI) for C_34_H_32_NO_3_S[M+H]^+^m/z: calcd 534.2097, found 534.2024.

##### 3-(4-methoxyphenyl)-5-phenyl-1-tosyl-4-(3-(trimethylsilyl)prop-2-yn-1-yl)-1,2,3,6-tetrahydropyridine (38c)

**Figure:**
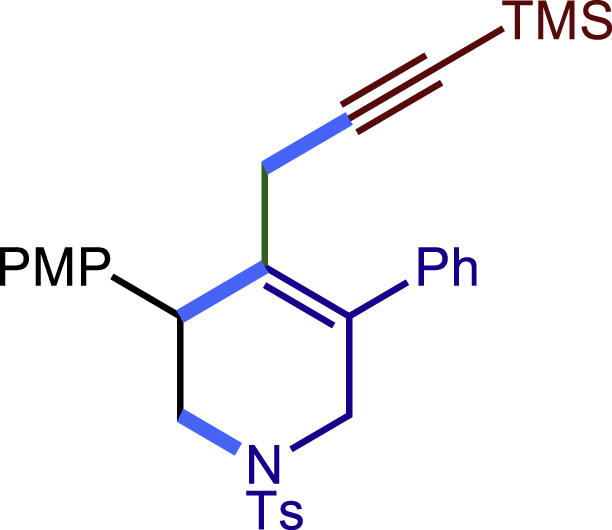

Prepared following general **Procedure C** using **1a** (63.4 mg, 0.2 mmol), 4-methoxystyrene (54 mg, 0.4 mmol, 2.0 eq.), and trimethoxy((trimethylsilyl)ethynyl)silane (88 mg, 0.4 mmol, 2.0 eq.). After 12 hours, the reaction mixture was purified by column chromatography using 6% EtOAc in PE to provide the title compound **38c** (60.4 mg, 57% yield) as a white solid. Rf = 0.25 (Petroleum ether: EtOAc =5:1)

^1^H NMR (400 MHz, CDCl_3_) δ 7.60 (d, *J* = 8.1 Hz, 2H), 7.40 – 7.31 (m, 3H), 7.30 – 7.22 (m, 6H), 6.87 (d, *J* = 8.5 Hz, 2H), 4.01 (d, *J =*16.1 Hz, 1H), 3.91 (s, 1H), 3.81 (s, 3H), 3.59 (d, *J =*16.0 Hz, 1H), 3.40 (dd, *J* = 11.4, 4.3 Hz, 1H), 3.27 (dd, *J* = 11.4, 4.8 Hz, 1H), 2.88 (d, *J =*17.5 Hz, 1H), 2.48 (d, *J* = 17.6 Hz, 1H), 2.41 (s, 3H), 0.14 (s, 9H).

^13^C NMR (101 MHz, CDCl_3_) δ 158.75, 143.67, 138.47, 132.78, 132.67, 132.00, 129.92, 129.74, 129.71, 128.77, 128.61, 127.85, 113.94, 104.06, 85.64, 55.26, 50.36, 49.54, 42.76, 22.82, 21.57, 0.12.

HRMS (ESI) for C_31_H_36_NO_3_SSi[M+H]^+^m/z: calcd 530.2180, found 530.2105.

##### 4-(3-cyclopropylprop-2-yn-1-yl)-3-(4-methoxyphenyl)-5-phenyl-1-tosyl-1,2,3,6-tetrahydropyridine (39c)

**Figure:**
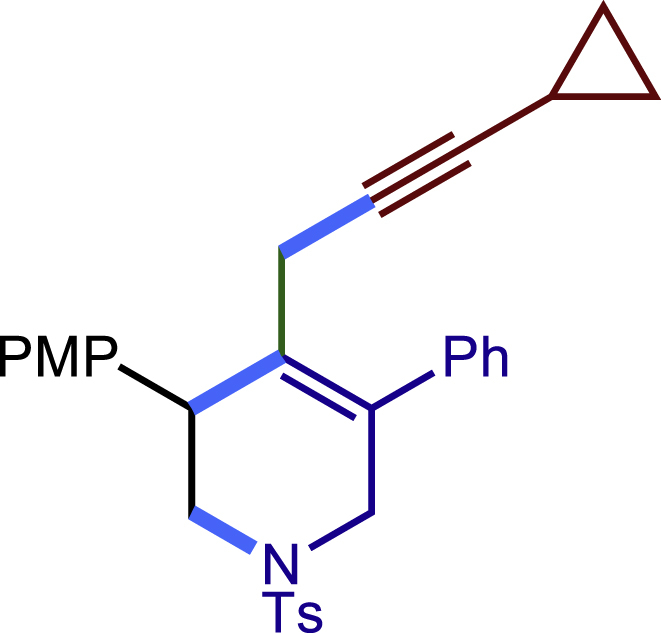

Prepared following general **Procedure C** using sing **1a** (63.4 mg, 0.2 mmol), 4-methoxystyrene (54 mg, 0.4 mmol, 2.0 eq.), and (cyclopropylethynyl)trimethoxysilane (76 mg, 0.4 mmol, 2.0 eq.). After 12 hours, the reaction mixture was purified by column chromatography using 6% EtOAc in PE to provide the title compound **39c** (49.8 mg, 50% yield) as a white solid. Rf = 0.3 (Petroleum ether: EtOAc =5:1)

^1^H NMR (400 MHz, CDCl_3_) δ 7.59 (d, *J =*8.1 Hz, 2H), 7.39 – 7.30 (m, 3H), 7.29 – 7.21 (m, 6H), 6.86 (d, *J =*8.5 Hz, 2H), 4.03 (d, *J* = 16.0 Hz, 1H), 3.88 (s, 1H), 3.80 (s, 3H), 3.55 (d, *J* = 16.0 Hz, 1H), 3.42 (dd, *J =*11.4, 4.1 Hz, 1H), 3.23 (dd, *J* = 11.4, 4.7 Hz, 1H), 2.78 (d, *J* = 17.1 Hz, 1H), 2.41 – 2.34 (m, 4H), 1.19 – 1.09 (m, 1H), 0.74 – 0.65 (m, 2H), 0.61 – 0.52 (m, 2H).

^13^C NMR (101 MHz, CDCl_3_) δ 158.69, 143.61, 138.62, 133.03, 132.75, 131.32, 130.78, 129.67, 129.65, 128.76, 128.56, 127.84, 127.76, 113.91, 84.24, 72.59, 55.26, 50.33, 49.45, 42.59, 21.60, 21.56, 8.05, 7.98.

HRMS (ESI) for C_31_H_32_NO_3_S[M+H]^+^m/z: calcd 498.2097, found 498.2026.

#### Characterization of products 40-43

##### 2-(3-(4-methoxyphenyl)-5-phenyl-1-tosyl-1,2,3,6-tetrahydropyridin-4-yl)acetamide (40)

**Figure:**
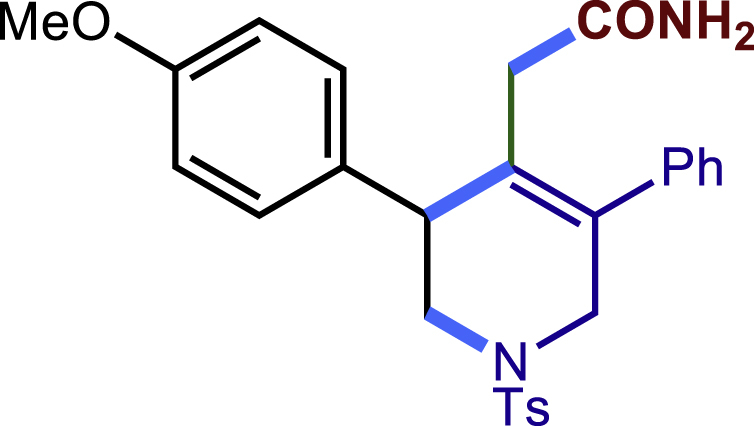

^1^H NMR (600 MHz, Chloroform-d) δ 7.58 (d, *J* = 6.4 Hz, 2H), 7.38 – 7.34 (m, 2H), 7.31 (t, *J* = 7.3 Hz, 1H), 7.28 – 7.24 (m, 4H), 7.22 (d, *J* = 8.6 Hz, 2H), 6.85 (d, *J* = 8.6 Hz, 2H), 5.53 (s, 1H), 5.25 (s, 1H), 4.07 (d, *J* = 16.3 Hz, 1H), 3.79 (s, 3H), 3.74 (s, 1H), 3.56 (d, *J* = 16.4 Hz, 1H), 3.44 (dd, *J* = 11.5, 4.2 Hz, 1H), 3.24 (dd, *J* = 11.6, 4.7 Hz, 1H), 2.88 (d, *J* = 15.8 Hz, 1H), 2.46 (d, *J* = 16.2 Hz, 1H), 2.41 (s, 3H).

^13^C NMR (151 MHz, Chloroform-d) δ 172.85, 158.84, 143.75, 138.54, 134.67, 132.83, 132.74, 129.70, 129.67, 129.00, 128.74, 128.69, 127.98, 127.79, 114.12, 55.26, 50.15, 49.65, 43.70, 37.90, 21.53.

HRMS (ESI) for C_27_H_29_FN_2_O_4_S[M+H]^+^m/z: calcd 477.1843, found 477.1833.

##### 2-(3-(2-oxopyrrolidin-1-yl)-5-phenyl-1,2,3,6-tetrahydropyridin-4-yl)acetonitrile (41)

**Figure:**
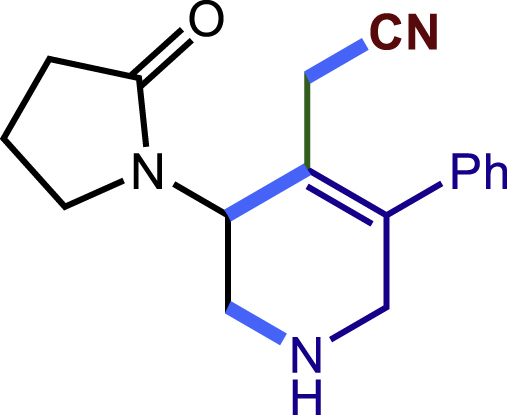

^1^H NMR (400 MHz, CDCl_3_) δ 7.40 -7 .36 (m, 2H), 7.35 – 7.29 (m, 1H), 7.21 – 7.16 (m, 2H), 4.68 (s, 1H), 3.73 – 3.63 (m, 1H), 3.59 – 3.48 (m, 3H), 3.22 – 3.09 (m, 2H), 2.97 (d, *J* = 16.9 Hz, 1H), 2.81 (d, J = 16.9 Hz, 1H), 2.55 – 2.36 (m, 2H), 2.19-2.02 (3H).

^13^C NMR (101 MHz, CDCl_3_) δ 176.08, 144.99, 138.21, 128.95, 128.25, 127.65, 120.40, 117.94, 50.64, 48.57, 47.79, 45.57, 31.11, 19.89, 18.06.

HRMS (ESI) for C_17_H_20_N_3_O [M+H]^+^m/z: calcd 282.1601, found 282.1584.

##### 2-(3-(4-methoxyphenyl)-5-phenyl-1-tosyl-2,3-dihydropyridin-4(1H)-ylidene)acetonitrile (42)

**Figure:**
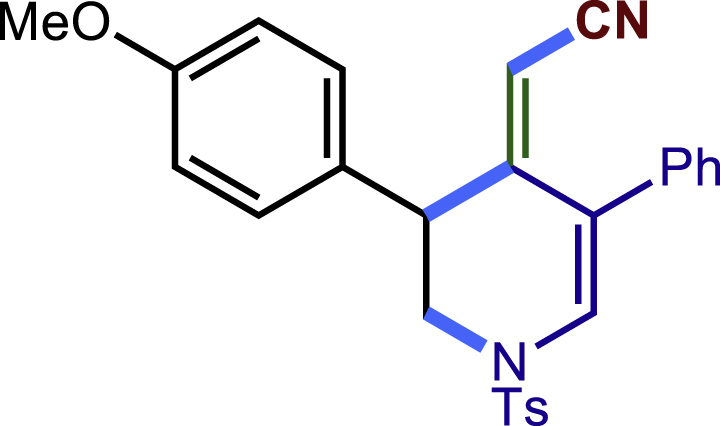

^1^H NMR (400 MHz, CDCl_3_) δ 7.49 (d, *J* = 8.3 Hz, 1H), 7.43 – 7.33 (m, 2H), 7.23 (dd, *J* = 7.8, 1.6 Hz, 1H), 7.19 (d, *J* = 8.1 Hz, 1H), 7.12 (s, 1H), 7.08 (d, *J* = 8.7 Hz, 1H), 6.69 (d, *J* = 8.7 Hz, 1H), 5.09 (s, 1H), 4.31 (d, *J* = 1.6 Hz, 1H), 4.13 (d, *J* = 12.7 Hz, 1H), 3.78 (s, 3H), 3.56 (dd, *J* = 12.6, 4.0 Hz, 1H), 2.42 (s, 3H).

^13^C NMR (101 MHz, CDCl_3_) δ 158.92, 153.76, 144.61, 136.04, 134.46, 130.74, 130.40, 129.97, 129.53, 128.91, 128.25, 128.01, 126.97, 119.52, 117.48, 114.17, 93.11, 55.19, 48.72, 41.27, 21.57.

HRMS (ESI) for C_27_H_25_N_2_O_3_S [M+H]^+^m/z: calcd 457.1580, found 457.1617.

##### 2-bromo-2-(5-(4-methoxyphenyl)-3-phenyl-1-tosyl-1,2-dihydropyridin-4-yl)acetonitrile (43)

**Figure:**
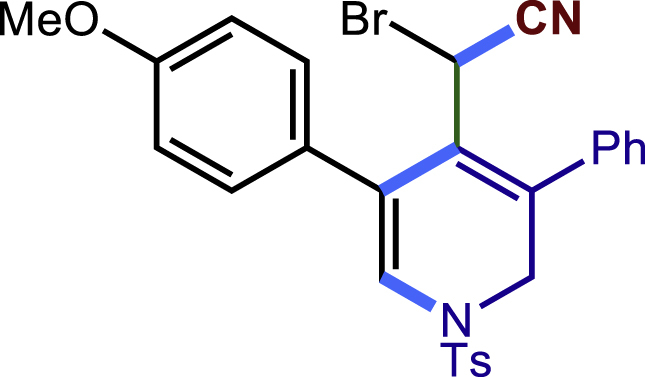

^1^H NMR (600 MHz, Chloroform-d) δ 7.54 (d, *J =*8.1 Hz, 2H), 7.43 – 7.37 (m, 3H), 7.26 – 7.19 (m, 4H), 7.12 (d, *J* = 8.7 Hz, 2H), 7.03 (s, 1H), 6.75 (d, *J* = 8.7 Hz, 2H), 4.36 (s, 1H), 4.22 (d, *J* = 14.5 Hz, 1H), 3.80 (s, 3H), 3.48 (dd, *J* = 13.0, 4.1 Hz, 1H), 2.44 (s, 3H).

^13^C NMR (151 MHz, Chloroform-d) δ 159.07, 149.85, 144.88, 136.45, 134.10, 131.59, 130.11, 129.84, 128.76, 128.71, 128.29, 128.00, 126.99, 117.55, 114.67, 114.33, 86.48, 55.22, 47.75, 42.01, 21.61.

HRMS (ESI) for C_27_H_24_BrN_2_O_3_S [M+H]^+^m/z: calcd 535.0686, found 535.0621.

## Data Availability

Crystallographic data for the structures reported in this article have been deposited at the Cambridge Crystallographic Data Centre (CCDC) under accession numbers CCDC 2192306 (**3a**), 2192304 (**43**). Copies of the data can be obtained free of charge from https://www.ccdc.cam.ac.uk/structures/. All other data are available from the lead contact upon reasonable request.

## Supplemental information

Document S1. Data S1 and Transparent methods
