# Supplementary material for: Modular synthesis of unsaturated aza-heterocycles via copper catalyzed multicomponent cascade reaction
Source: iScience. 2023 Feb 9;26(3):106137. doi: 10.1016/j.isci.2023.106137 (PMC9988680; doi:10.1016/j.isci.2023.106137)
Supplement: Document S1. Data S1 and Transparent methods [file mmc1.pdf]

iScience, Volume *26*

# Supplemental information

# Modular synthesis of unsaturated aza-heterocycles via copper catalyzed multicomponent cascade reaction

**Siqi Wei, Guocong Zhang, Yahui Wang, Mengwei You, Yanan Wang, Liejin Zhou, and Zuxiao Zhang**

# Modular Synthesis of Unsaturated Aza-heterocycles via Copper Catalyzed Multicomponent Radical Cascade Reaction

**Siqi Wei, Guocong Zhang, Yahui Wang, Mengwei You, Yanan Wang, Liejin Zhou, and Zuxiao Zhang**

# Data S1. Spectra of new compounds: Related to Scheme 1, 2 and Figure 2.

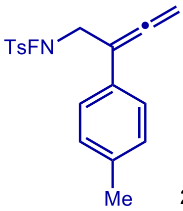


**2a**

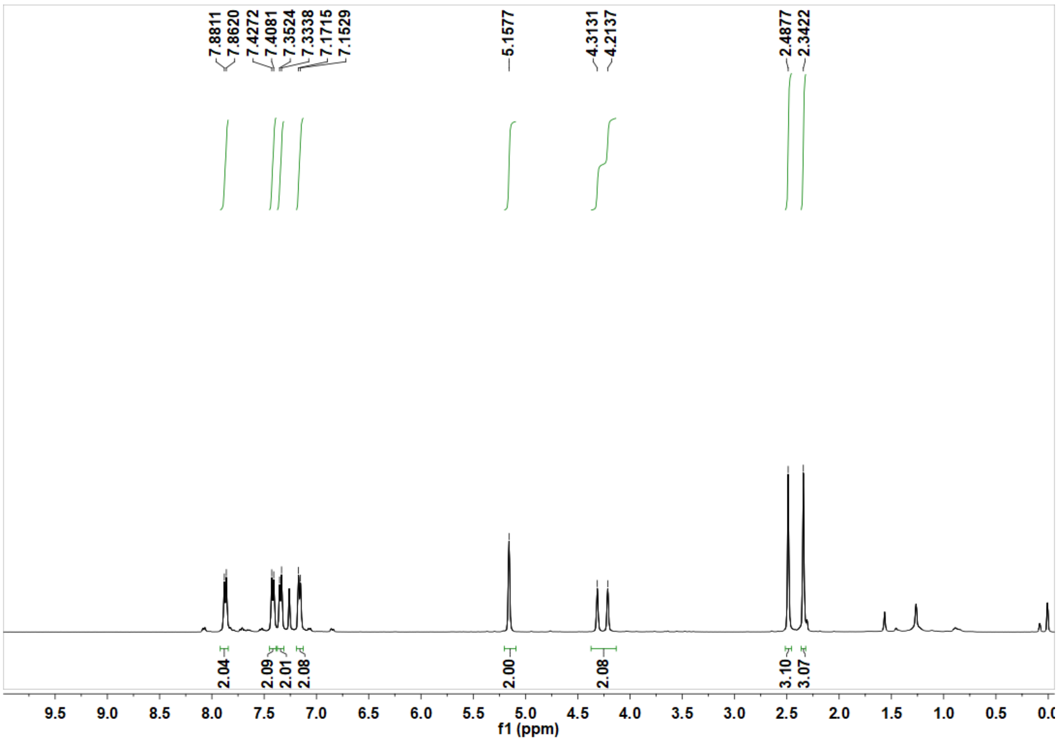


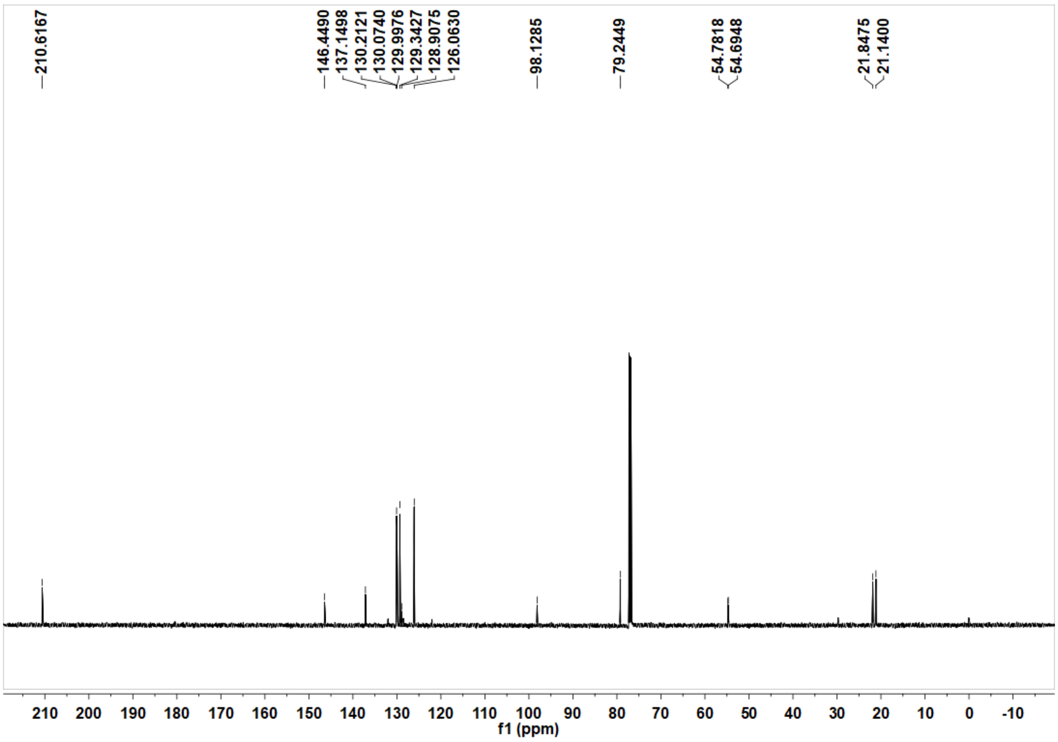

wsq-2-49-8-f.10.fid

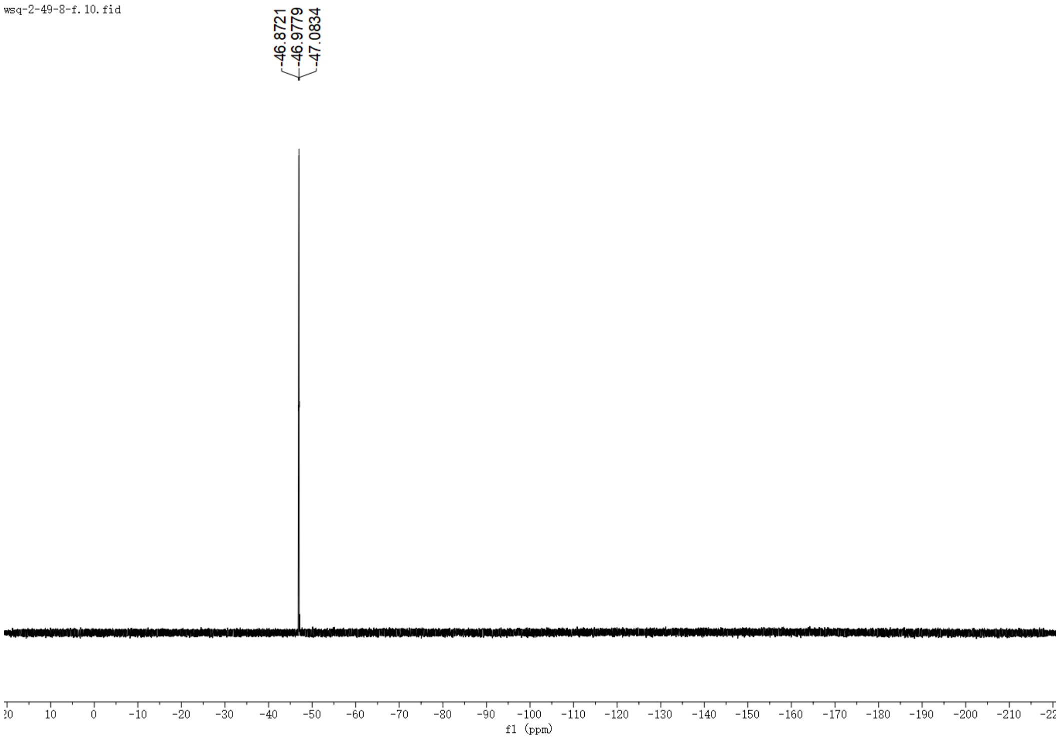

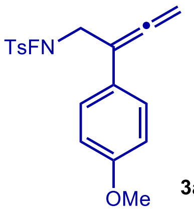
TsFN
OMe

**3a**

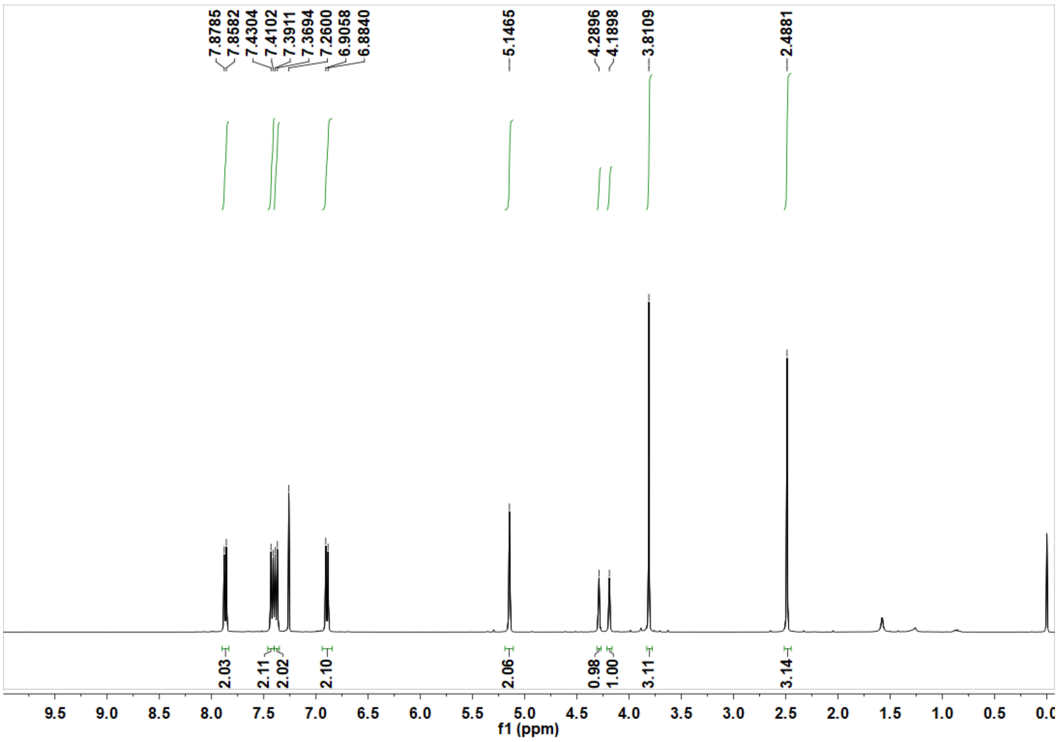
7.8785
7.8582
7.4304
7.4102
7.3911
7.3694
7.2600
6.9058
6.8840
5.1465
4.2896
4.1898
3.8109
2.4881
2.03
2.11
2.02
2.10
2.06
0.98
1.00
3.11
3.14
f1 (ppm)

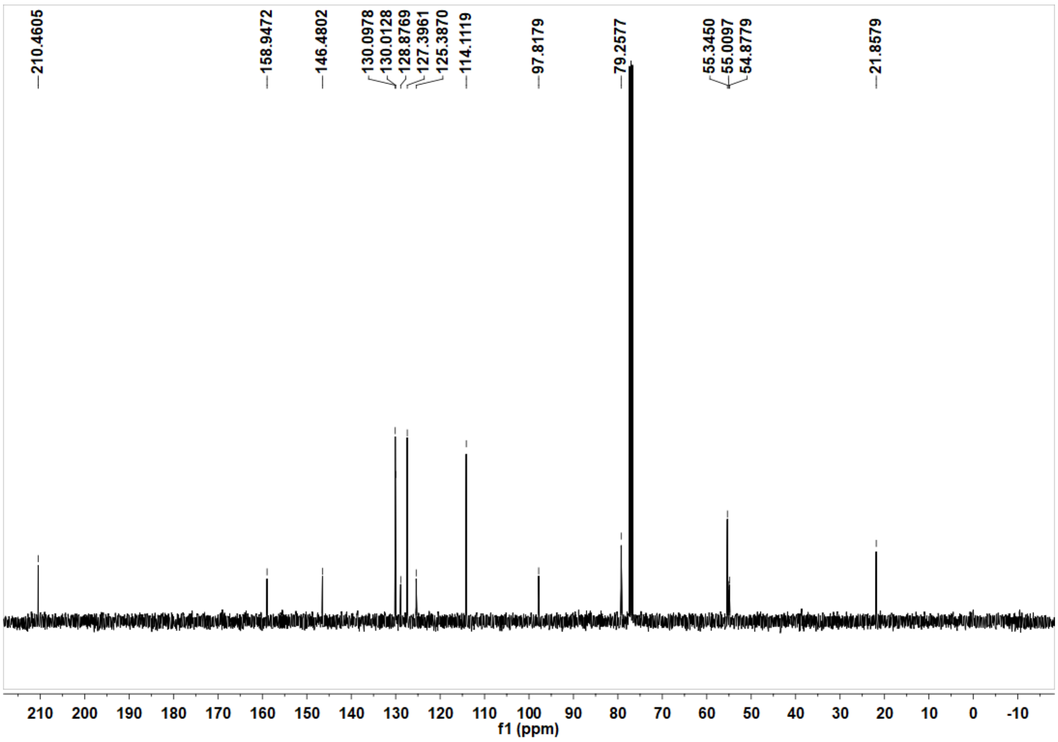
210.4605
158.9472
146.4802
130.0978
130.0128
128.8769
127.3961
125.3870
114.1119
97.8179
79.2577
55.3450
55.0097
54.8779
21.8579
f1 (ppm)

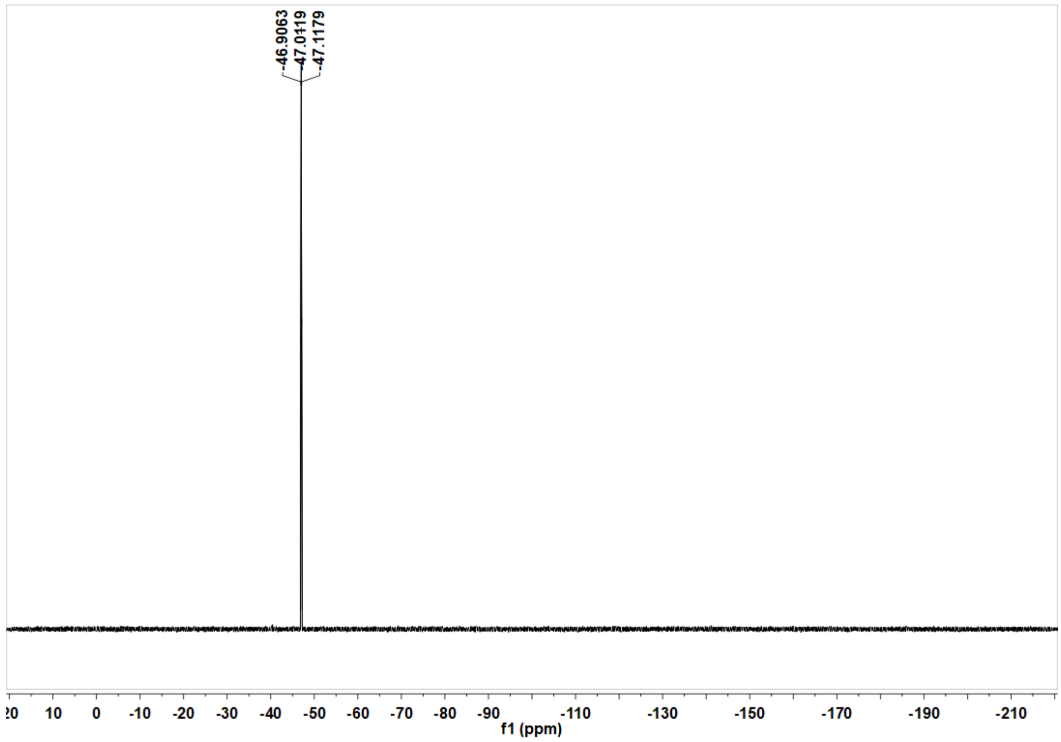

-46.9063
-47.0119
-47.1179
20
10
0
-10
-20
-30
-40
-50
-60
-70
-80
-90
-110
-130
-150
-170
-190
-210
f1 (ppm)

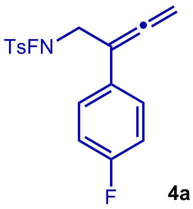
TsFN
F

**4a**

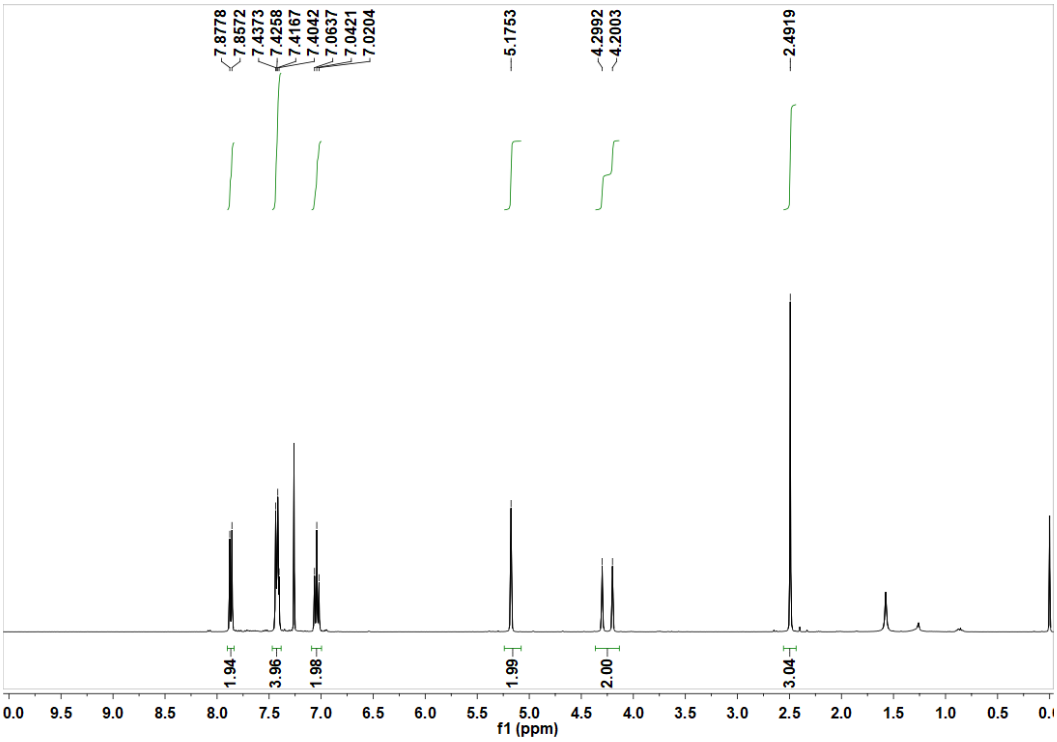
7.8778
7.8572
7.4373
7.4258
7.4167
7.4042
7.0637
7.0421
7.0204
5.1753
4.2992
4.2003
2.4919
1.94
3.96
1.98
1.99
2.00
3.04
f1 (ppm)

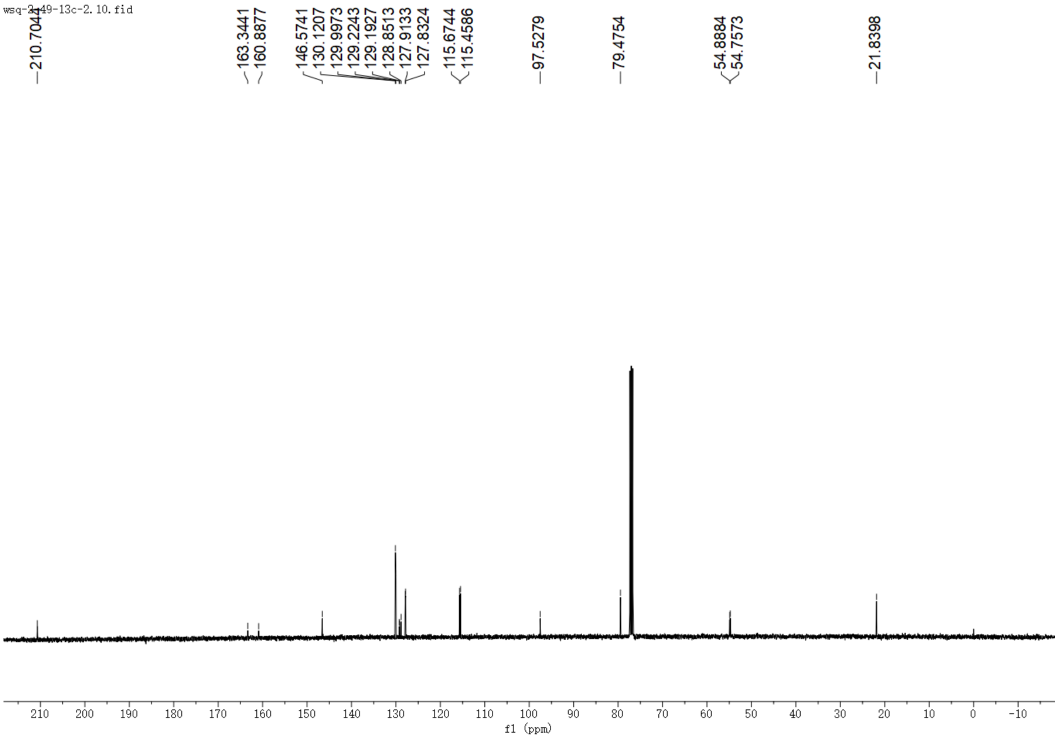
wsq-2-49-13c-2.10.fid
210.7041
163.3441
160.8877
146.5741
130.1207
129.9973
129.2243
129.1927
128.8513
127.9133
127.8324
115.6744
115.4586
97.5279
79.4754
54.8884
54.7573
21.8398
f1 (ppm)

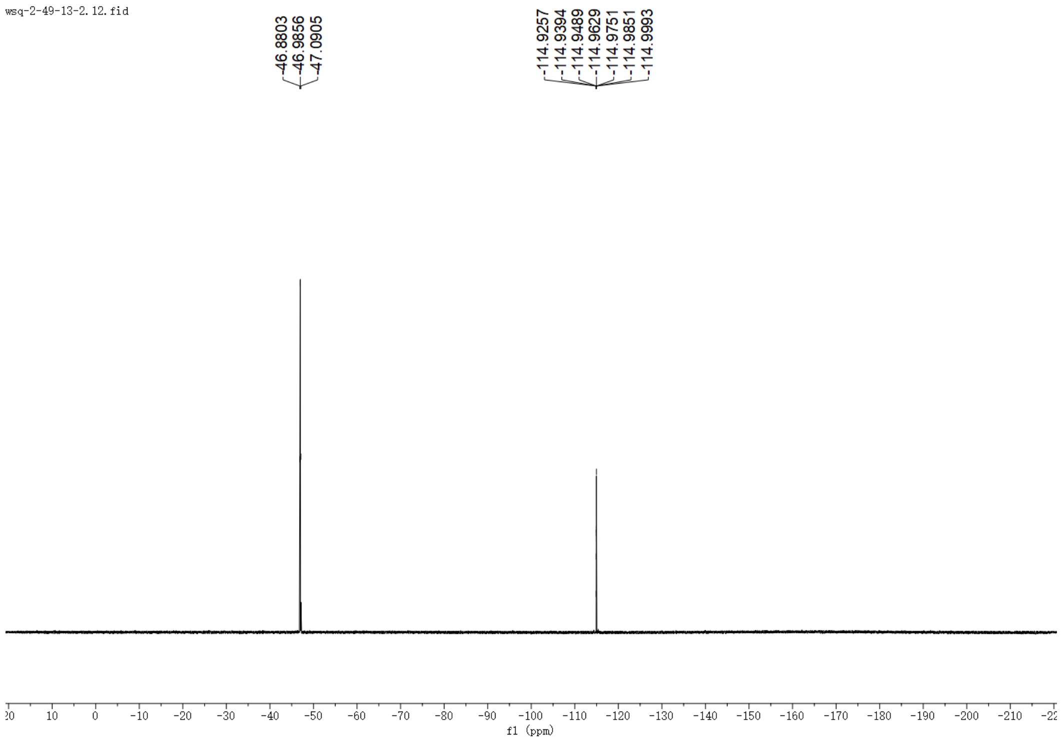

wsq-2-49-13-2.12.fid
-46.8803
-46.9856
-47.0905
-114.9257
-114.9394
-114.9489
-114.9629
-114.9751
-114.9851
-114.9993
f1 (ppm)

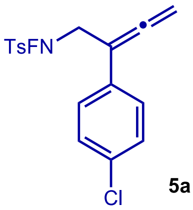

TsFN
Cl


**5a**

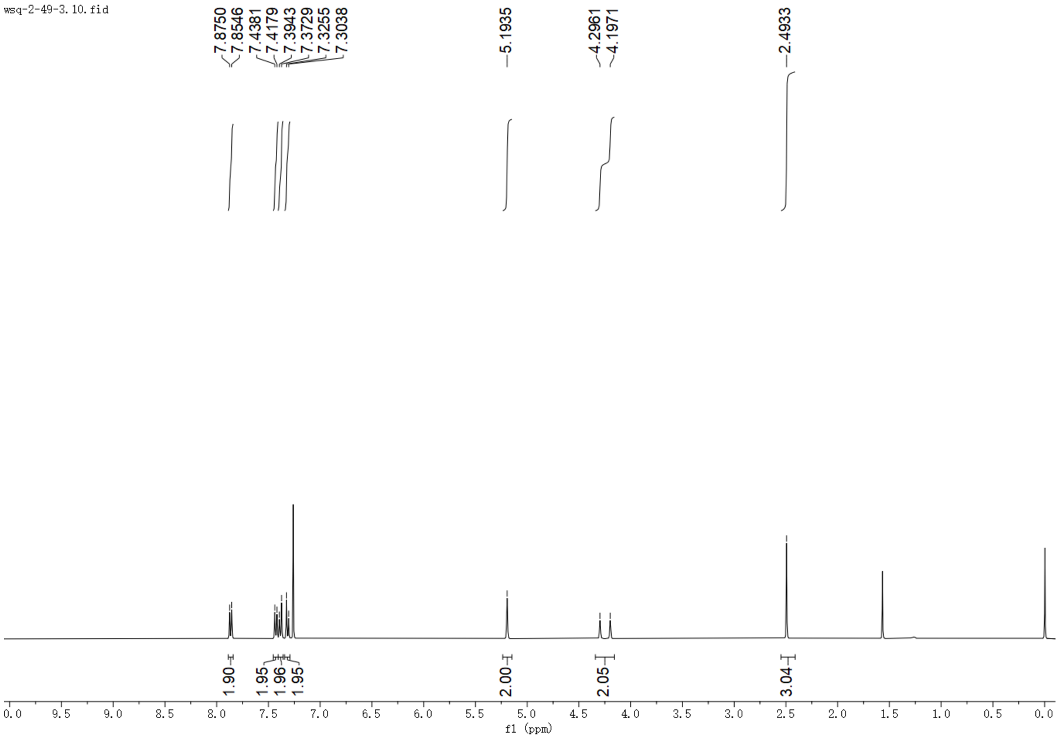

wsq-2-49-3.10.fid
7.8750
7.8546
7.4381
7.4179
7.3943
7.3729
7.3255
7.3038
5.1935
4.2961
4.1971
2.4933
1.90
1.95
1.96
1.95
2.00
2.05
3.04
10.0
9.5
9.0
8.5
8.0
7.5
7.0
6.5
6.0
5.5
5.0
4.5
4.0
3.5
3.0
2.5
2.0
1.5
1.0
0.5
0.0
f1 (ppm)


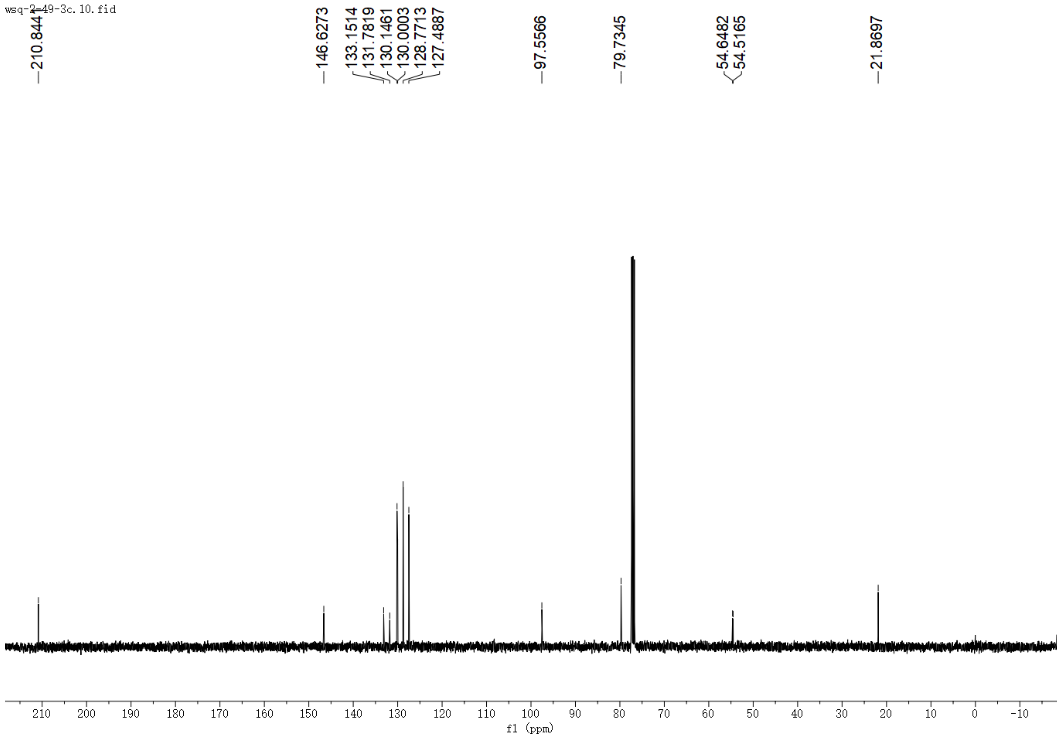

wsq-2-49-3c.10.fid
210.8441
146.6273
133.1514
131.7819
130.1461
130.0003
128.7713
127.4887
97.5566
79.7345
54.6482
54.5165
21.8697
210
200
190
180
170
160
150
140
130
120
110
100
90
80
70
60
50
40
30
20
10
0
-10
f1 (ppm)

wsq-2-49-3.12.fid

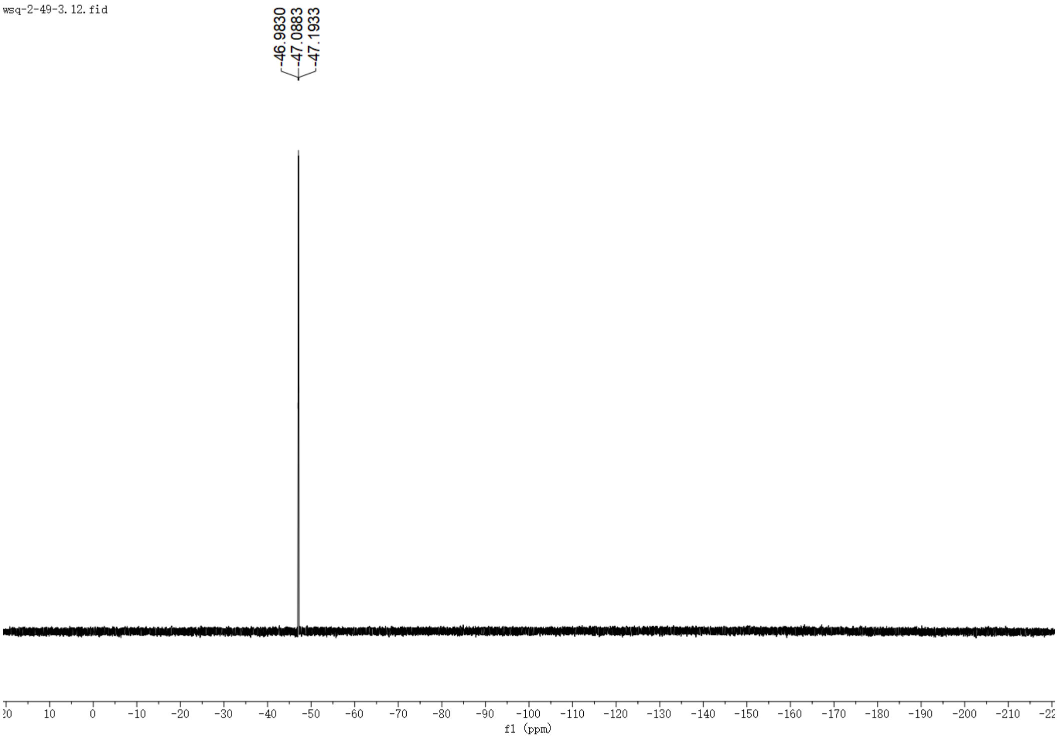

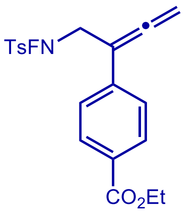
TsFN
CO2Et

**6a**

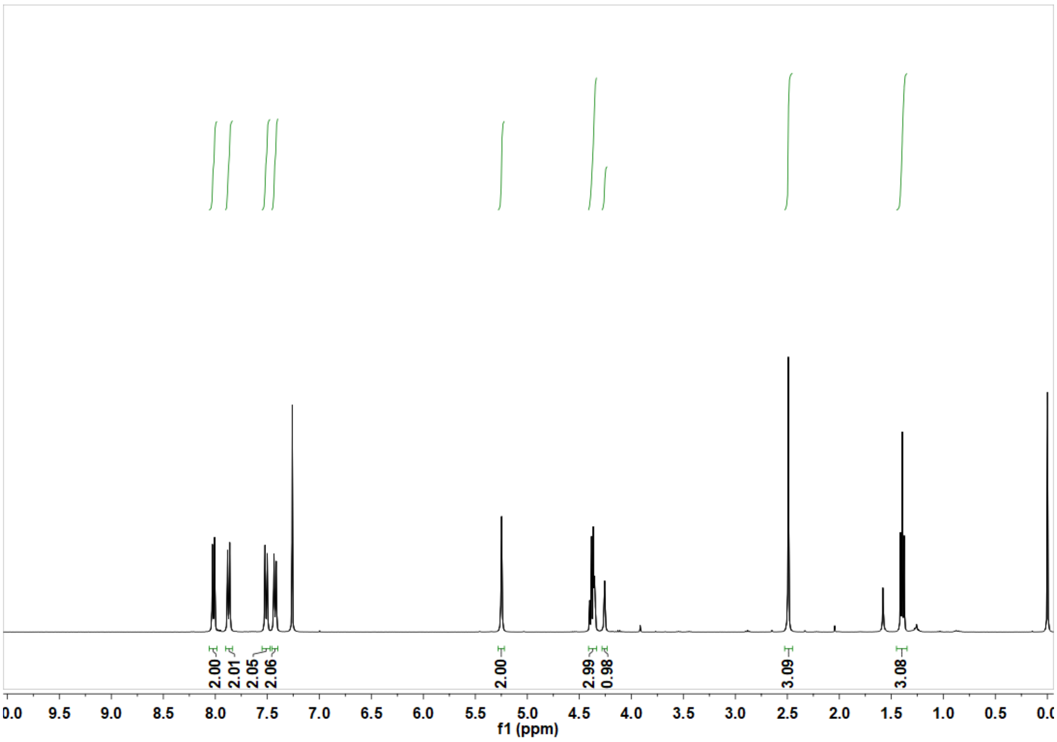
2.00
2.01
2.05
2.06
2.00
2.99
0.98
3.09
3.08
f1 (ppm)

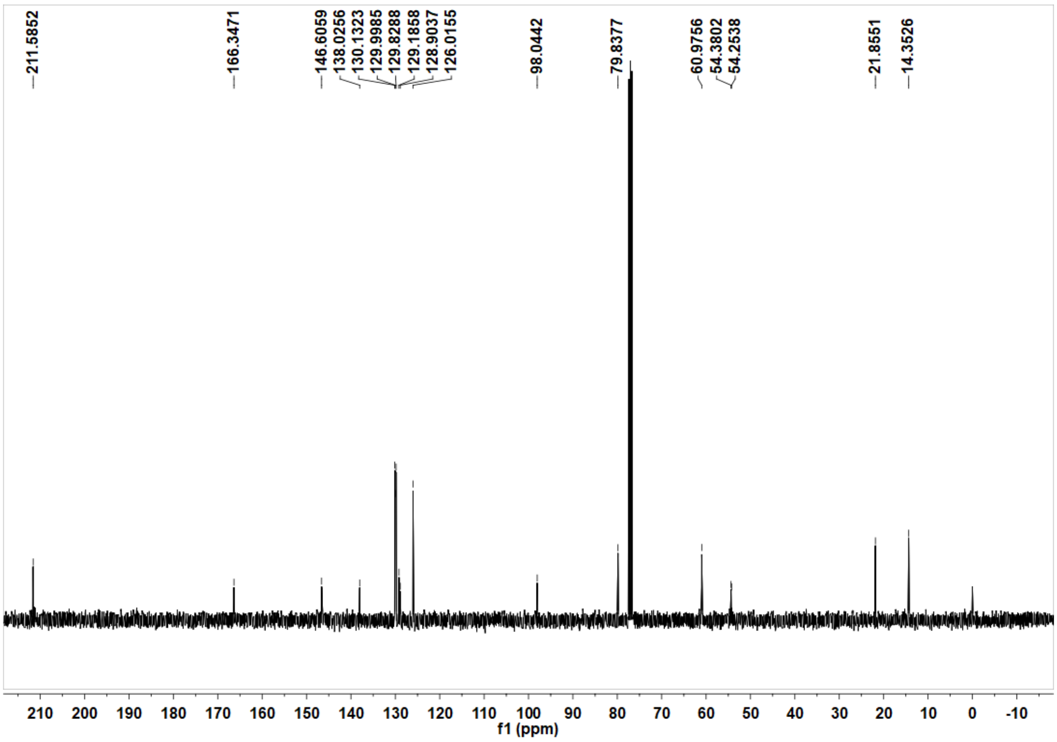
211.5852
166.3471
146.6059
138.0256
130.1323
129.9985
129.8288
129.1858
128.9037
126.0155
98.0442
79.8377
60.9756
54.3802
54.2538
21.8551
14.3526
f1 (ppm)

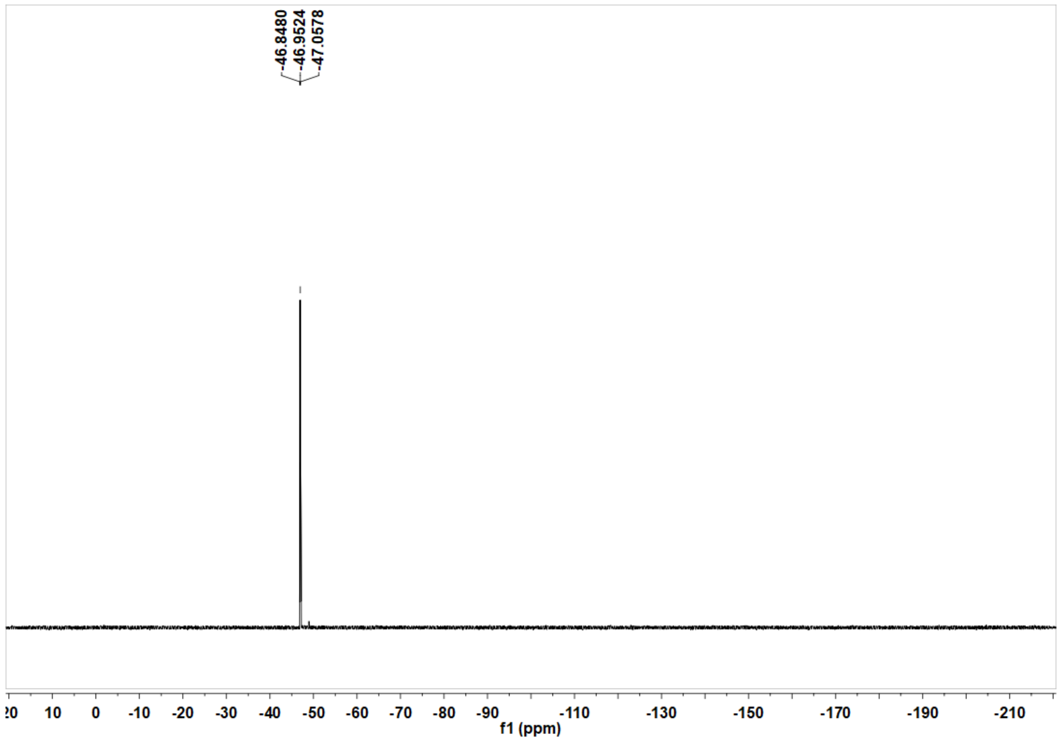

-46.8480
-46.9524
-47.0578
20
10
0
-10
-20
-30
-40
-50
-60
-70
-80
-90
-110
-130
-150
-170
-190
-210
f1 (ppm)

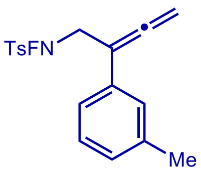
TsFN
Me

**7a**

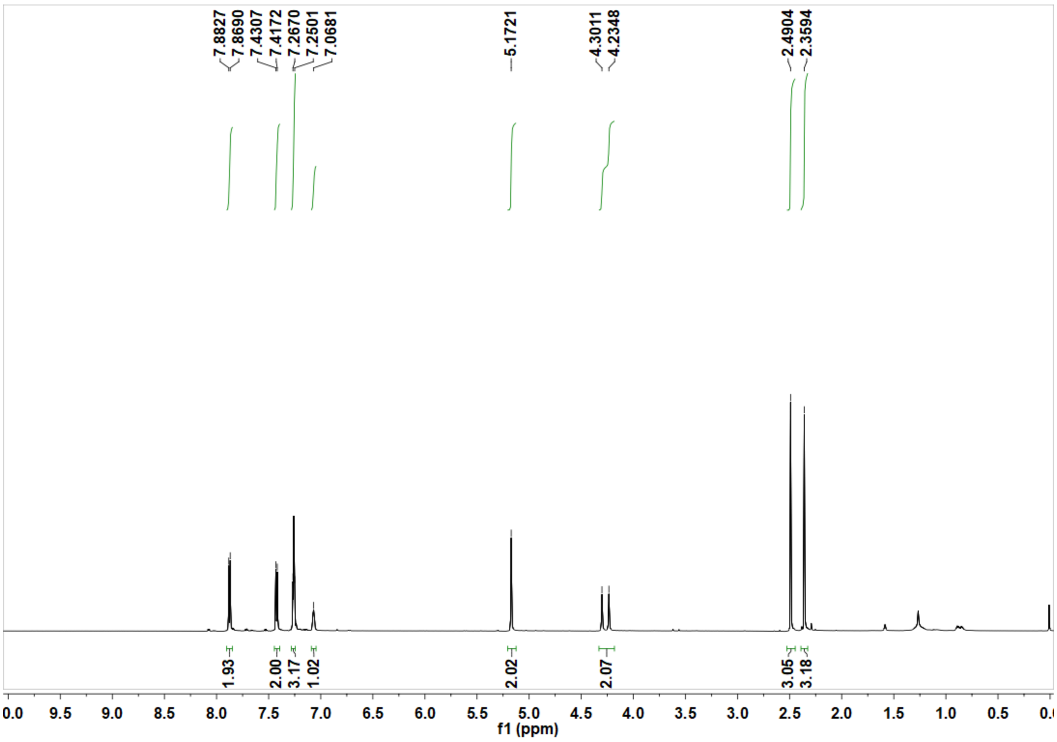
7.8827
7.8690
7.4307
7.4172
7.2670
7.2501
7.0681
5.1721
4.3011
4.2348
2.4904
2.3594
1.93
2.00
3.17
1.02
2.02
2.07
3.05
3.18
f1 (ppm)

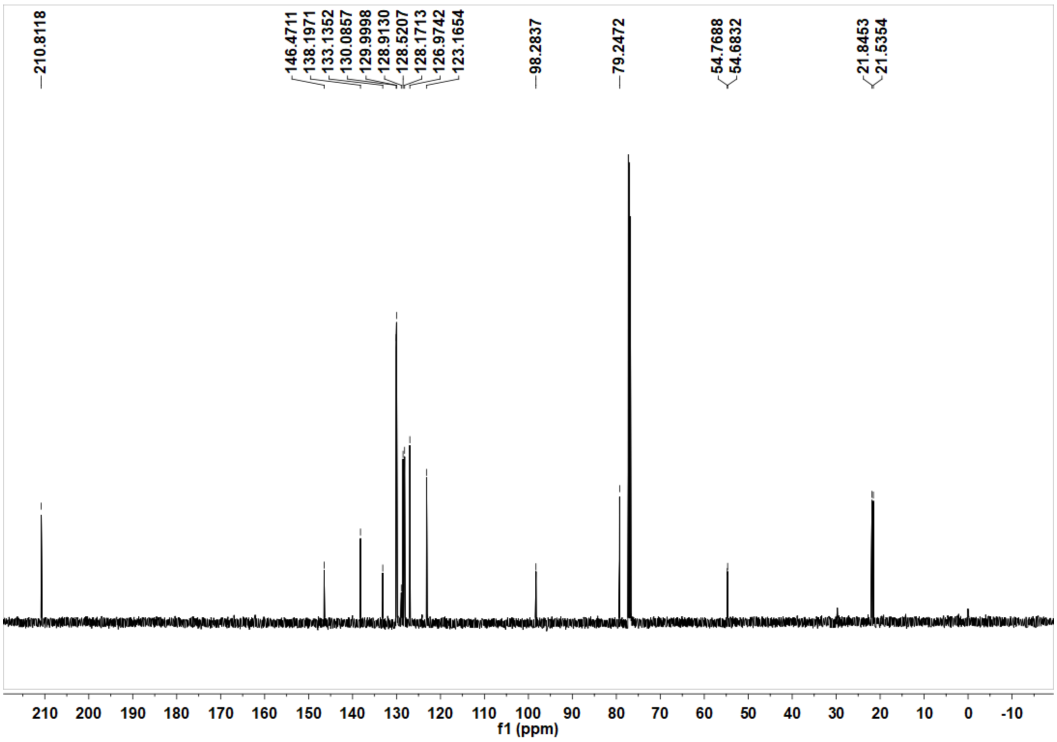
210.8118
146.4711
138.1971
133.1352
130.0857
129.9998
128.9130
128.5207
128.1713
126.9742
123.1654
98.2837
79.2472
54.7688
54.6832
21.8453
21.5354
f1 (ppm)

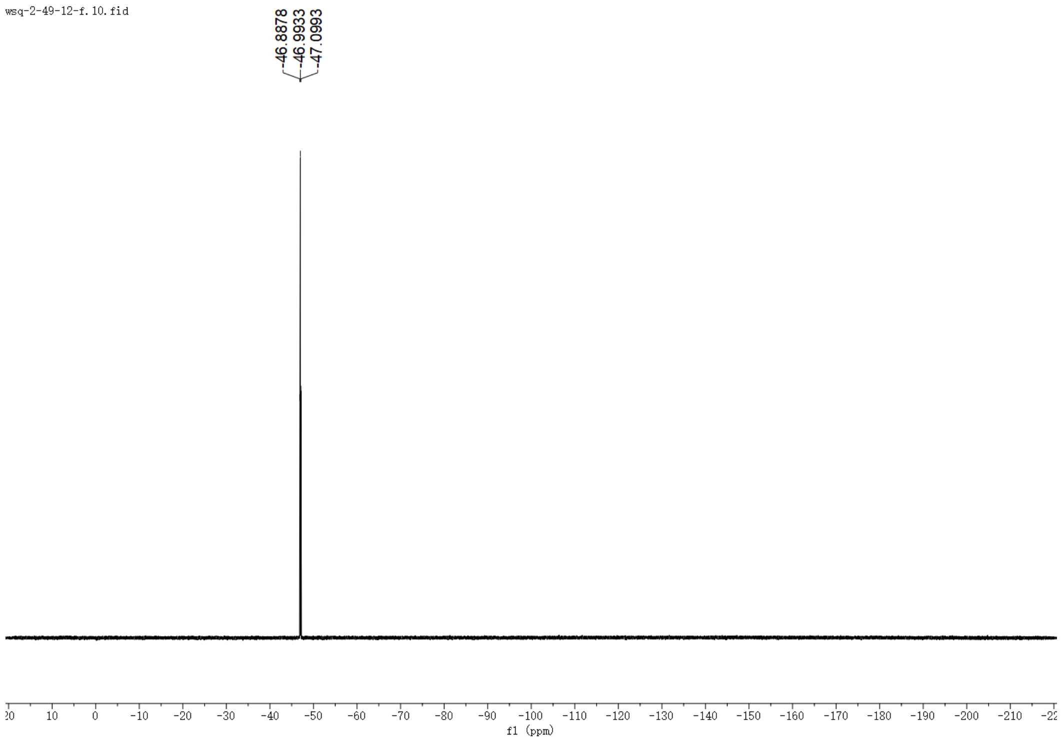
wsq-2-49-12-f.10.fid
-46.8878
-46.9933
-47.0993
f1 (ppm)

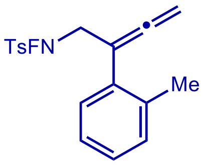
TsFN
Me

**8a**

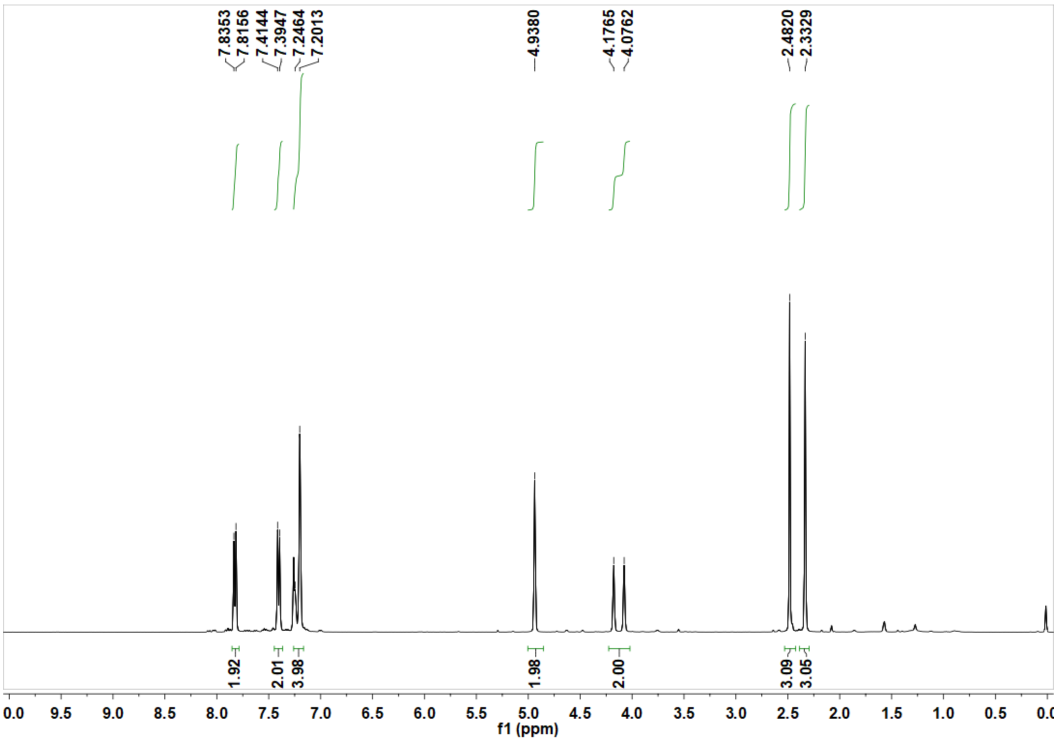
7.8353
7.8156
7.4144
7.3947
7.2464
7.2013
4.9380
4.1765
4.0762
2.4820
2.3329
1.92
2.01
3.98
1.98
2.00
3.09
3.05
0.0 9.5 9.0 8.5 8.0 7.5 7.0 6.5 6.0 5.5 5.0 4.5 4.0 3.5 3.0 2.5 2.0 1.5 1.0 0.5 0.0
f1 (ppm)

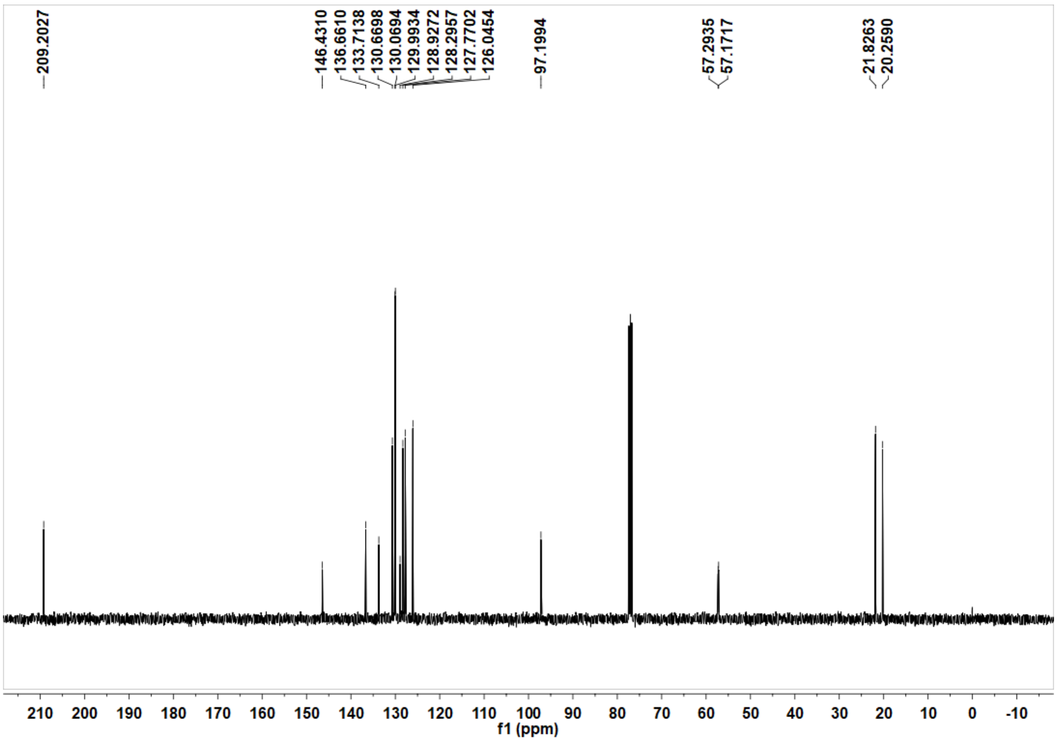
209.2027
146.4310
136.6610
133.7138
130.6698
130.0694
129.9934
128.9272
128.2957
127.7702
126.0454
97.1994
57.2935
57.1717
21.8263
20.2590
210 200 190 180 170 160 150 140 130 120 110 100 90 80 70 60 50 40 30 20 10 0 -10
f1 (ppm)

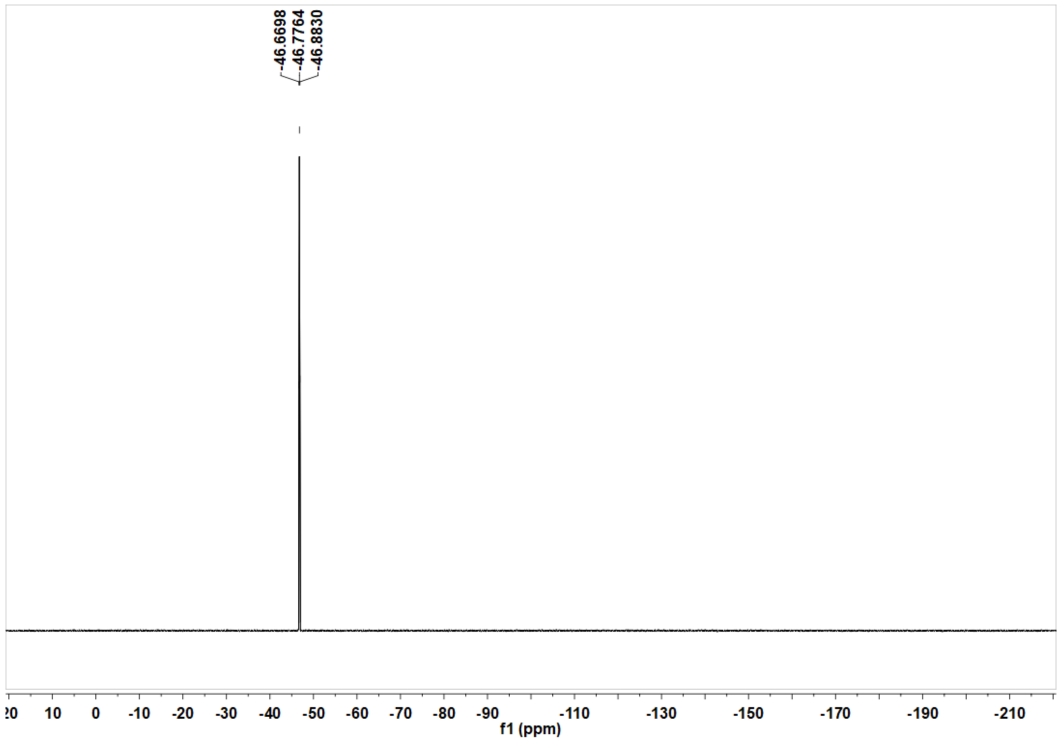
-46.6698
-46.7764
-46.8830
20 10 0 -10 -20 -30 -40 -50 -60 -70 -80 -90 -110 -130 -150 -170 -190 -210
f1 (ppm)

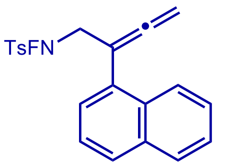
TsFN

**9a**

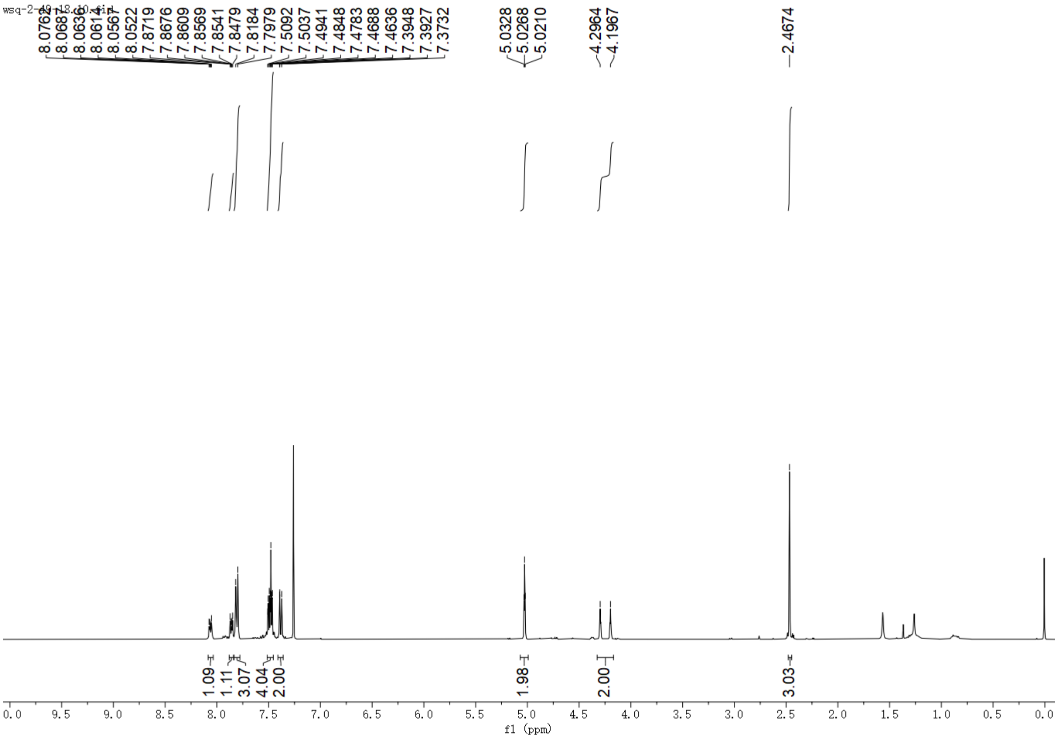
8.0767
8.0687
8.0636
8.0617
8.0567
8.0522
7.8719
7.8676
7.8609
7.8569
7.8541
7.8479
7.8184
7.7979
7.5092
7.5037
7.4941
7.4848
7.4783
7.4688
7.4636
7.3948
7.3927
7.3732
5.0328
5.0268
5.0210
4.2964
4.1967
2.4674
1.09
1.11
3.07
4.04
2.00
1.98
2.00
3.03
f1 (ppm)

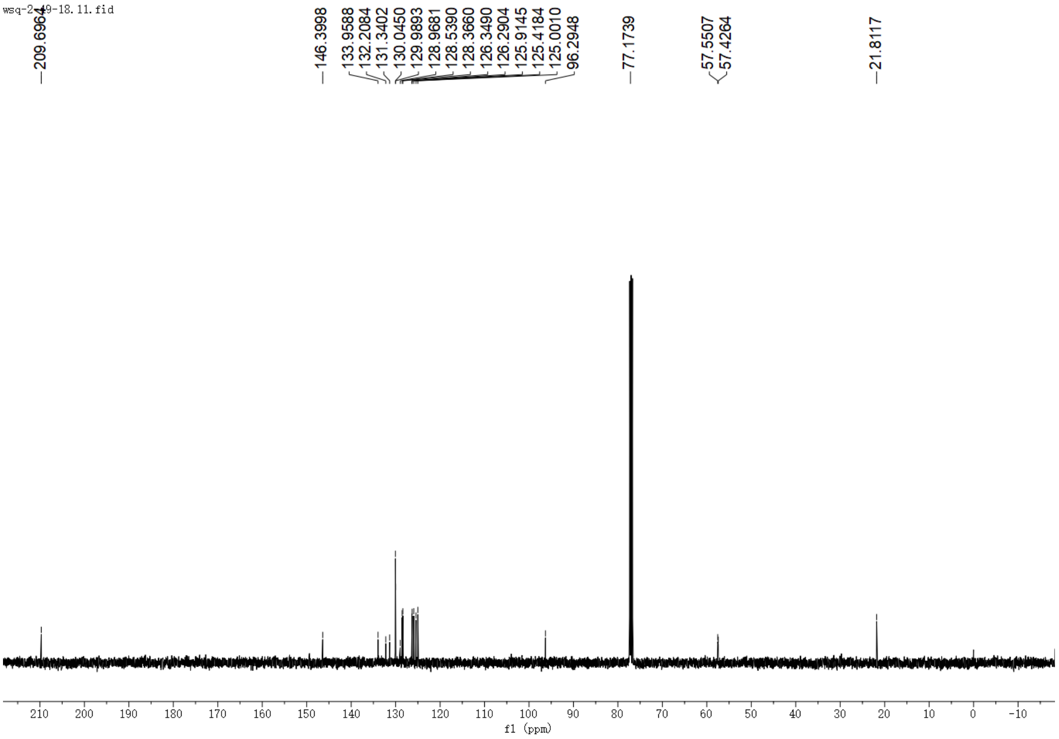
wsq-2-49-18.11.fid
209.6964
146.3998
133.9588
132.2084
131.3402
130.0450
129.9893
128.9681
128.5390
128.3660
126.3490
126.2904
125.9145
125.4184
125.0010
96.2948
77.1739
57.5507
57.4264
21.8117
f1 (ppm)

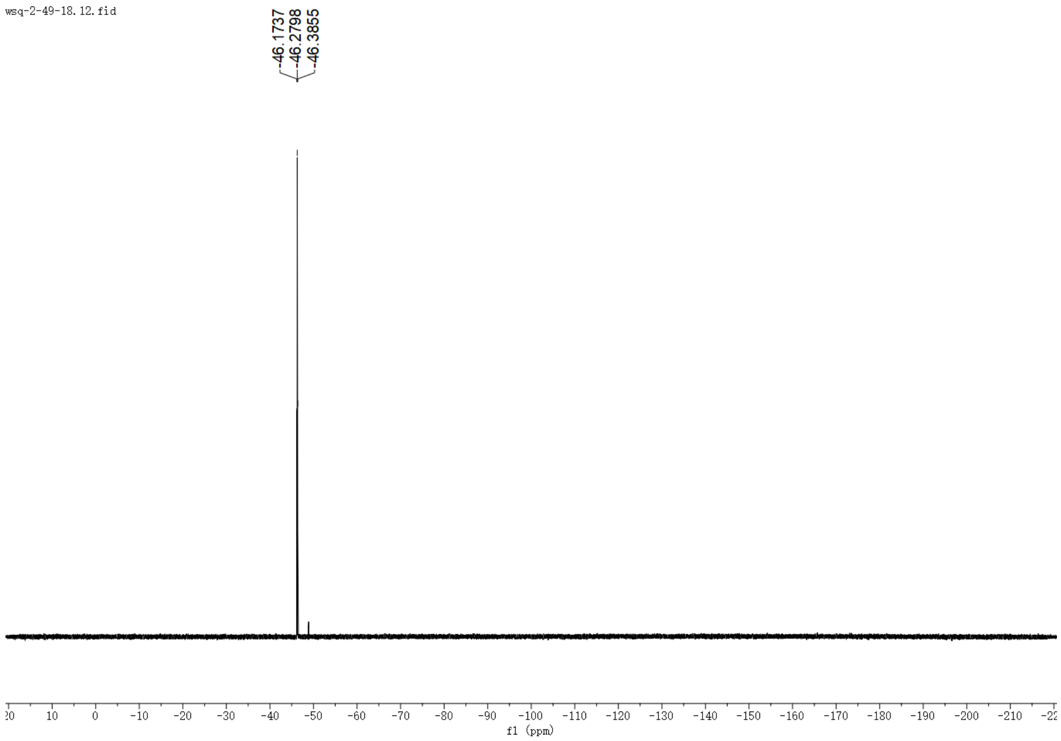
wsq-2-49-18.12.fid
-46.1737
-46.2798
-46.3855
f1 (ppm)

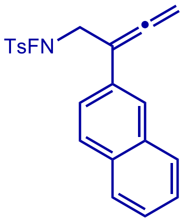
TsFN

**10a**

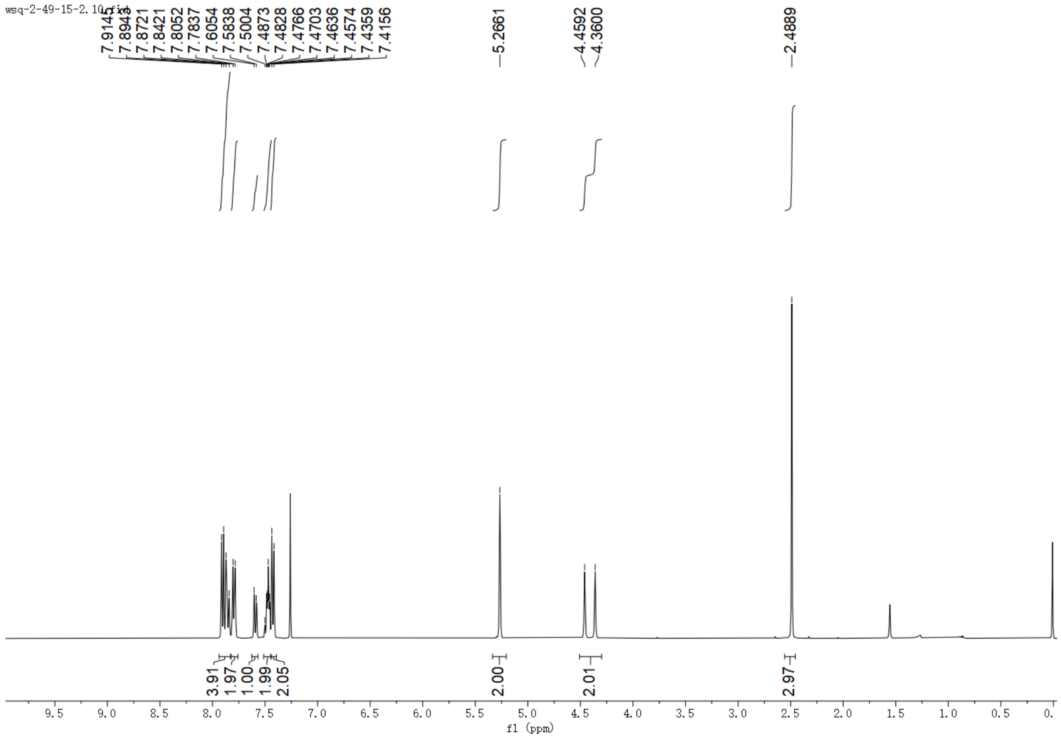
wsq-2-49-15-2.10.fid
7.9145
7.8943
7.8721
7.8421
7.8052
7.7837
7.6054
7.5838
7.5004
7.4873
7.4828
7.4766
7.4703
7.4636
7.4574
7.4359
7.4156
5.2661
4.4592
4.3600
2.4889
3.91
1.97
1.00
1.99
2.05
2.00
2.01
2.97
9.5 9.0 8.5 8.0 7.5 7.0 6.5 6.0 5.5 5.0 4.5 4.0 3.5 3.0 2.5 2.0 1.5 1.0 0.5 0.
f1 (ppm)

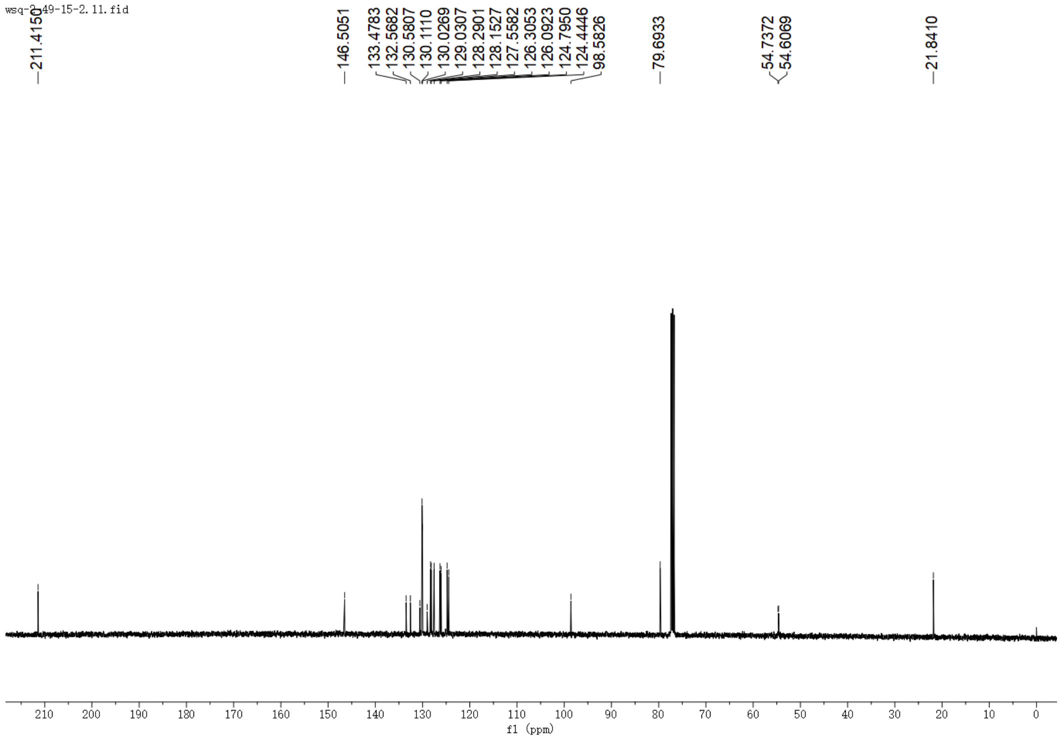
wsq-2-49-15-2.11.fid
211.4150
146.5051
133.4783
132.5682
130.5807
130.1110
130.0269
129.0307
128.2901
128.1527
127.5582
126.3053
126.0923
124.7950
124.4446
98.5826
79.6933
54.7372
54.6069
21.8410
210 200 190 180 170 160 150 140 130 120 110 100 90 80 70 60 50 40 30 20 10 0
f1 (ppm)

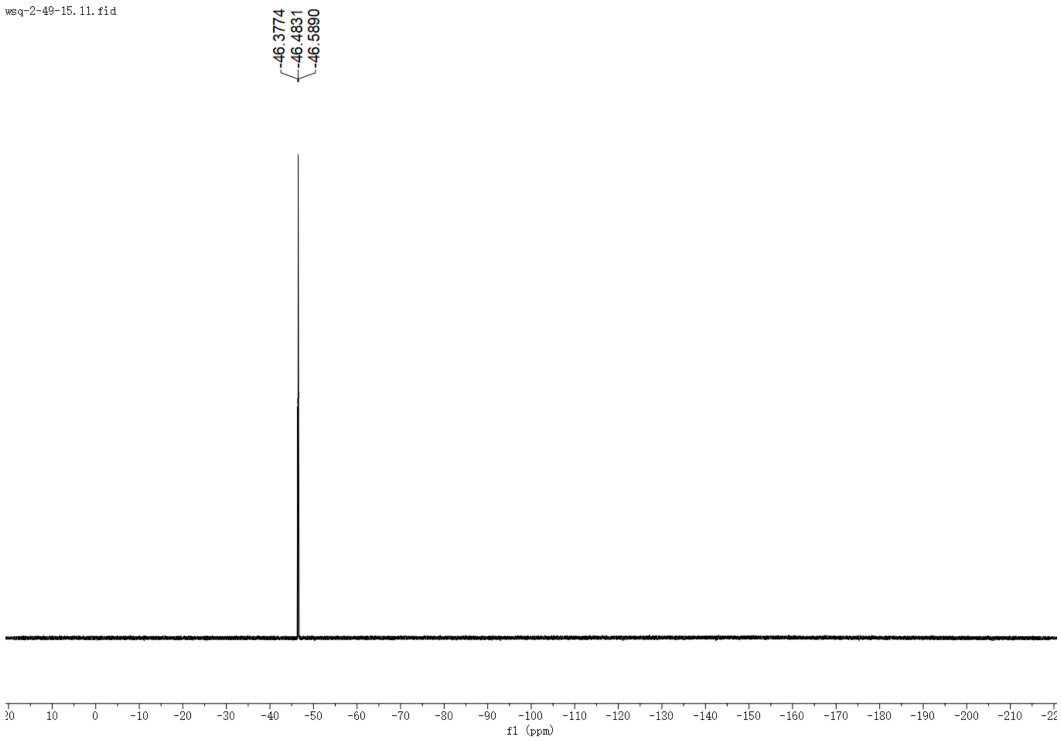

wsq-2-49-15.11.fid
-46.3774
-46.4831
-46.5890
20
10
0
-10
-20
-30
-40
-50
-60
-70
-80
-90
-100
-110
-120
-130
-140
-150
-160
-170
-180
-190
-200
-210
-220
f1 (ppm)

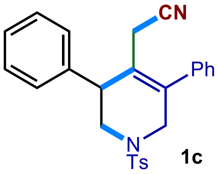
CN
Ph
N
Ts

**1c**

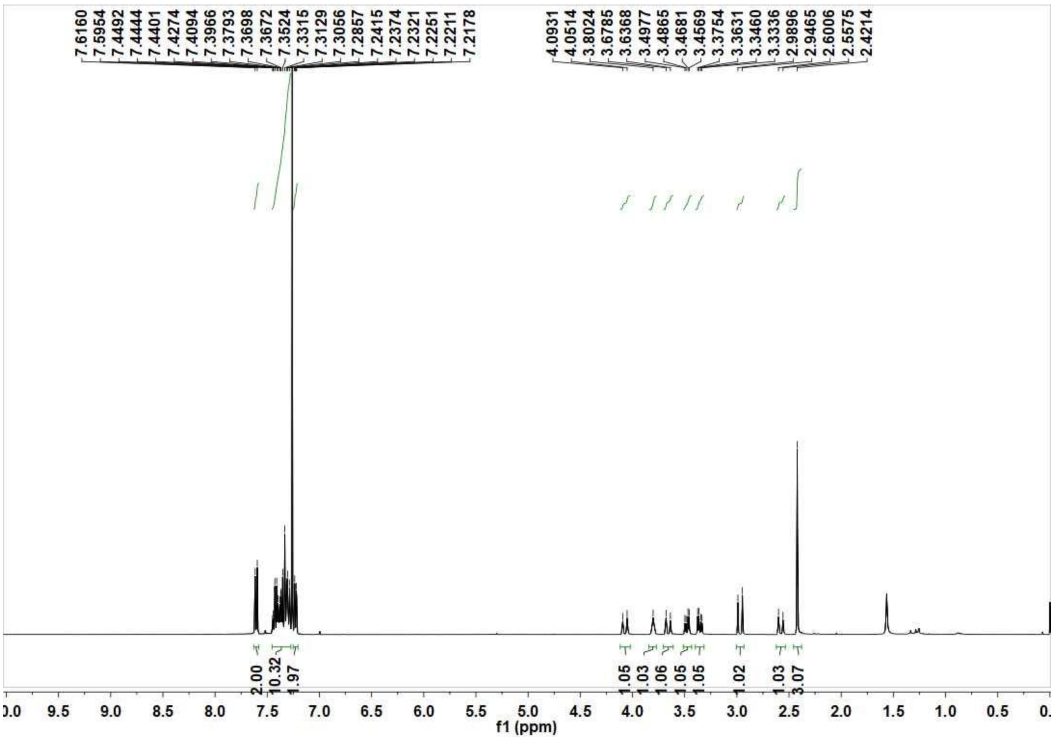
7.6160
7.5954
7.4492
7.4444
7.4401
7.4274
7.4094
7.3966
7.3793
7.3698
7.3672
7.3524
7.3315
7.3129
7.3066
7.2857
7.2415
7.2374
7.2321
7.2251
7.2211
7.2178
4.0931
4.0514
3.8024
3.6785
3.6368
3.4977
3.4865
3.4681
3.4569
3.3754
3.3631
3.3460
3.3336
2.9896
2.9465
2.6006
2.5575
2.4214
2.00
10.32
1.97
1.05
1.03
1.06
1.05
1.05
1.02
1.03
3.07
f1 (ppm)

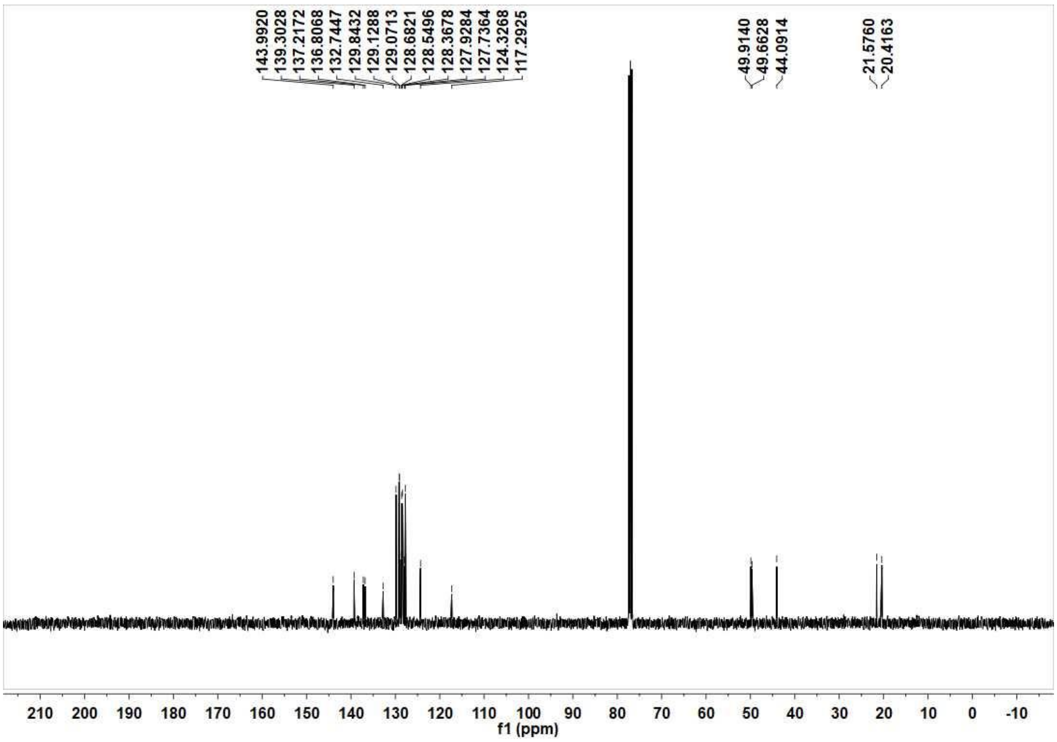
143.9920
139.3028
137.2172
136.8068
132.7447
129.8432
129.1288
129.0713
128.6821
128.5496
128.3678
127.9284
127.7364
124.3268
117.2925
49.9140
49.6628
44.0914
21.5760
20.4163
f1 (ppm)

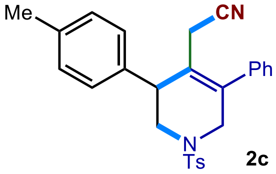

Me
CN
Ph
N
Ts


**2c**

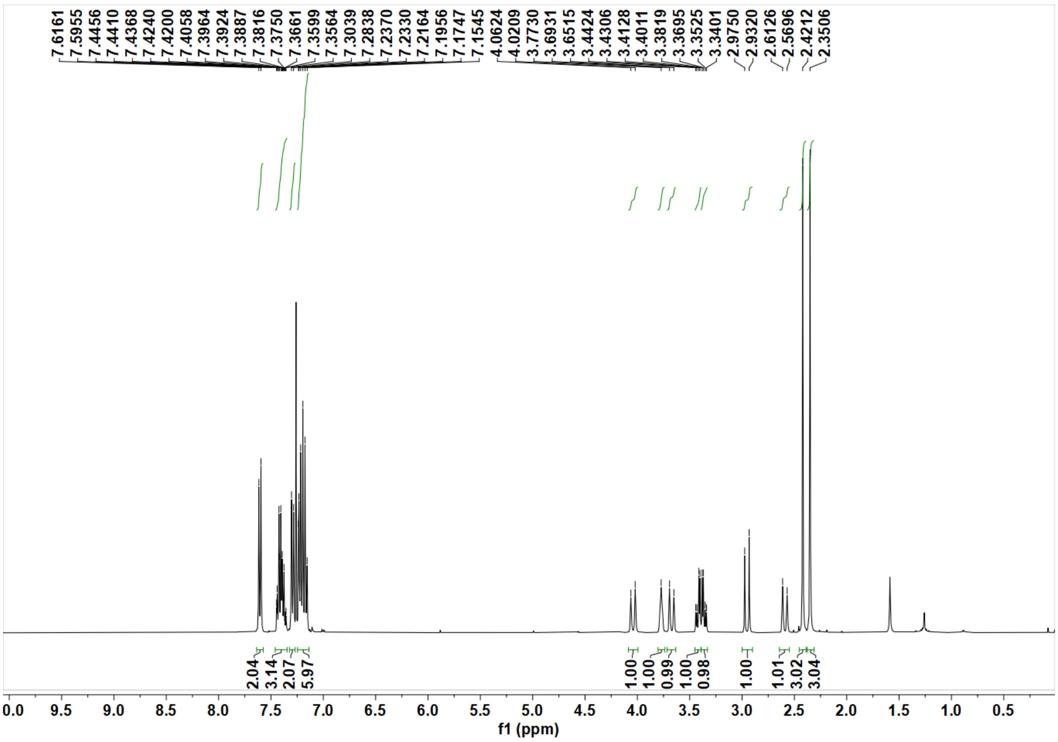

7.6161
7.5955
7.4456
7.4410
7.4368
7.4240
7.4200
7.4058
7.3964
7.3924
7.3887
7.3816
7.3750
7.3661
7.3599
7.3564
7.3039
7.2838
7.2370
7.2330
7.2164
7.1956
7.1747
7.1545
4.0624
4.0209
3.7730
3.6931
3.6515
3.4424
3.4306
3.4128
3.4011
3.3819
3.3695
3.3525
3.3401
2.9750
2.9320
2.6126
2.5696
2.4212
2.3506
2.04
3.14
2.07
5.97
1.00
1.00
0.99
1.00
0.98
1.00
1.01
3.02
3.04
0.0 9.5 9.0 8.5 8.0 7.5 7.0 6.5 6.0 5.5 5.0 4.5 4.0 3.5 3.0 2.5 2.0 1.5 1.0 0.5
f1 (ppm)


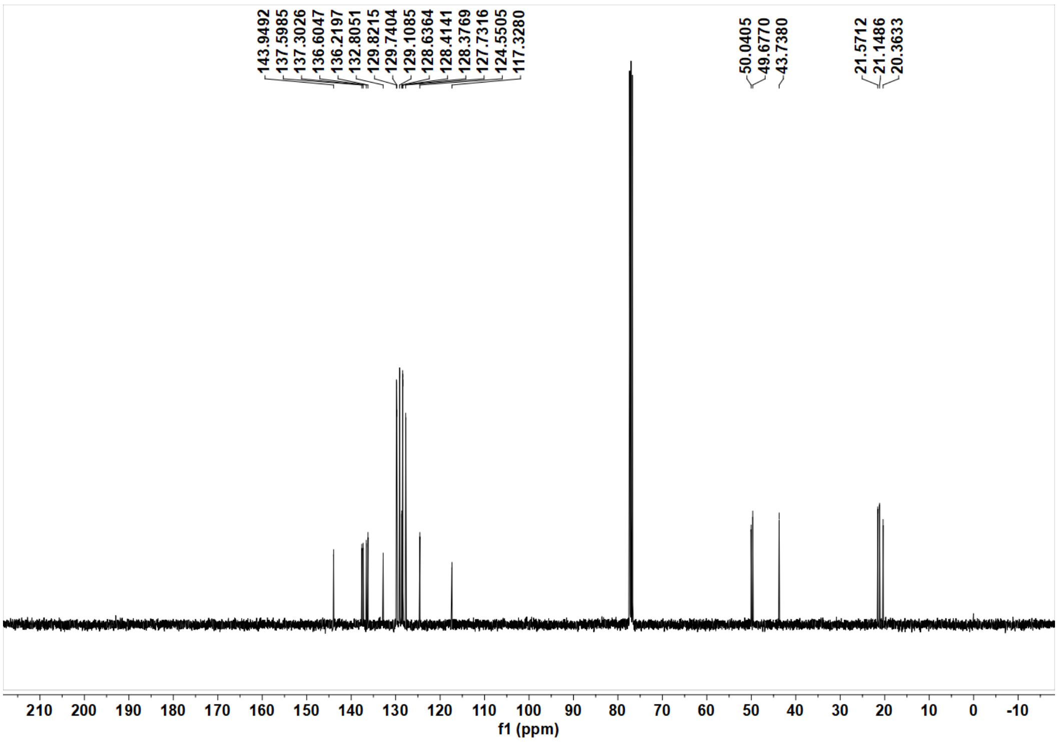

143.9492
137.5985
137.3026
136.6047
136.2197
132.8051
129.8215
129.7404
129.1085
128.6364
128.4141
128.3769
127.7316
124.5505
117.3280
50.0405
49.6770
43.7380
21.5712
21.1486
20.3633
210 200 190 180 170 160 150 140 130 120 110 100 90 80 70 60 50 40 30 20 10 0 -10
f1 (ppm)

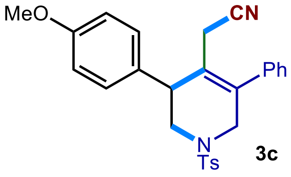
MeO
CN
Ph
N
Ts

**3c**

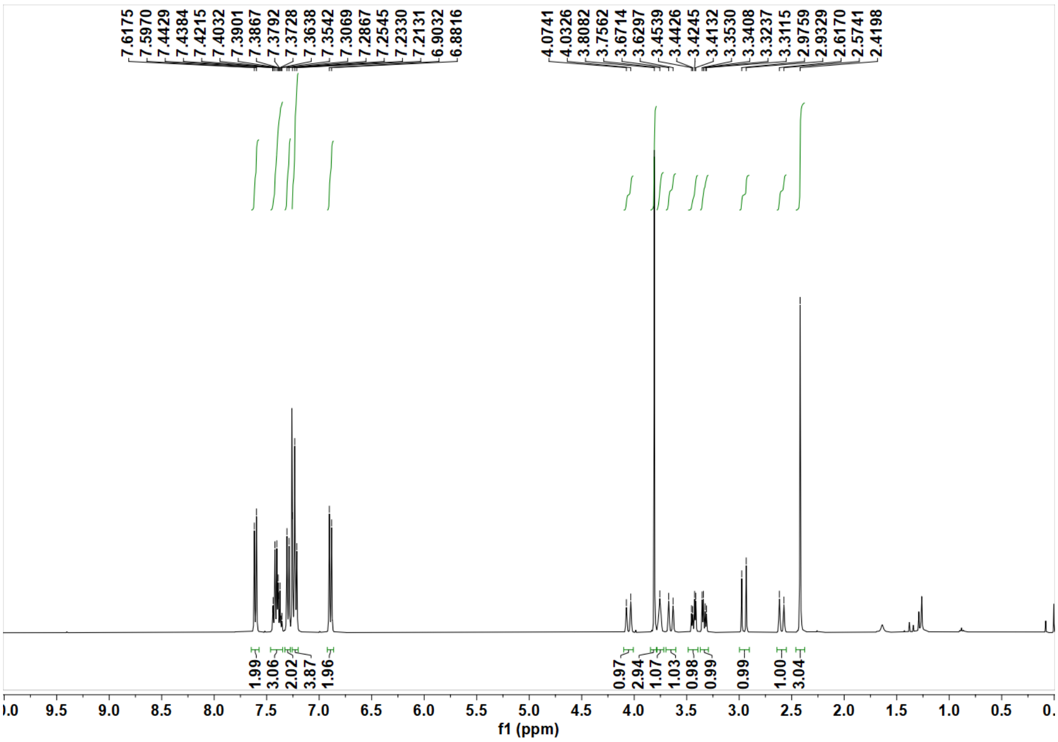
7.6175
7.5970
7.4429
7.4384
7.4215
7.4032
7.3901
7.3867
7.3792
7.3728
7.3638
7.3542
7.3069
7.2867
7.2545
7.2330
7.2131
6.9032
6.8816
4.0741
4.0326
3.8082
3.7562
3.6714
3.6297
3.4539
3.4426
3.4245
3.4132
3.3530
3.3408
3.3237
3.3115
2.9759
2.9329
2.6170
2.5741
2.4198
1.99
3.06
2.02
3.87
1.96
0.97
2.94
1.07
1.03
0.98
0.99
0.99
1.00
3.04
f1 (ppm)

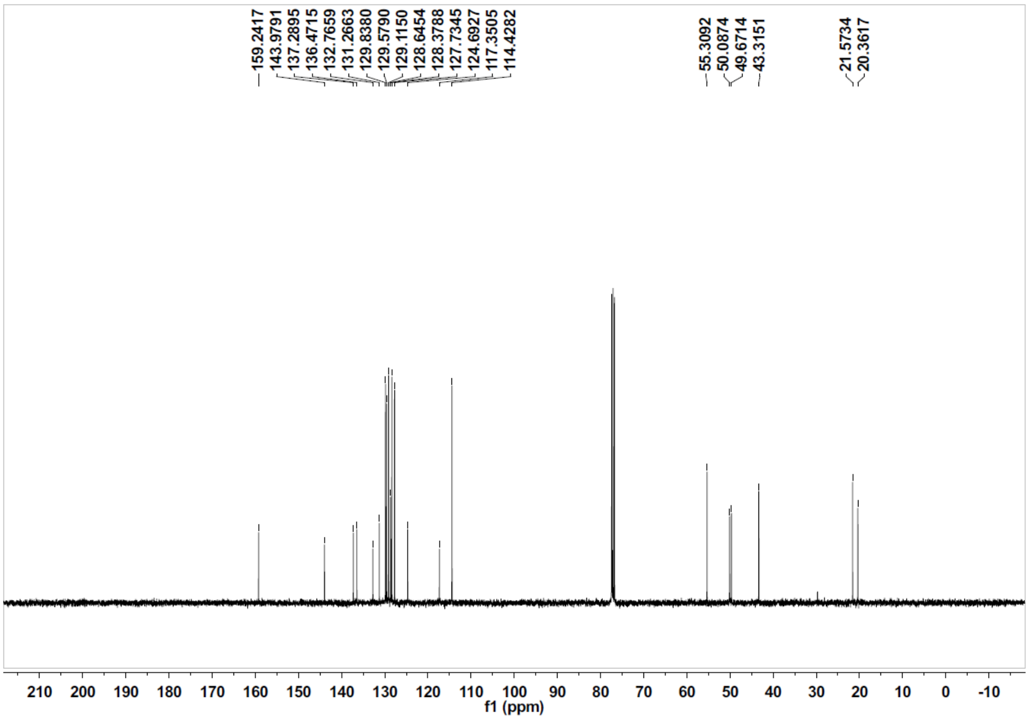
159.2417
143.9791
137.2895
136.4715
132.7659
131.2663
129.8380
129.5790
129.1150
128.6454
128.3788
127.7345
124.6927
117.3505
114.4282
55.3092
50.0874
49.6714
43.3151
21.5734
20.3617
f1 (ppm)

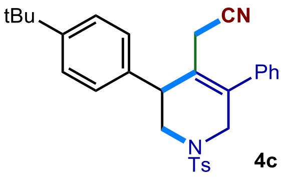
tBu
CN
Ph
N
Ts

**4c**

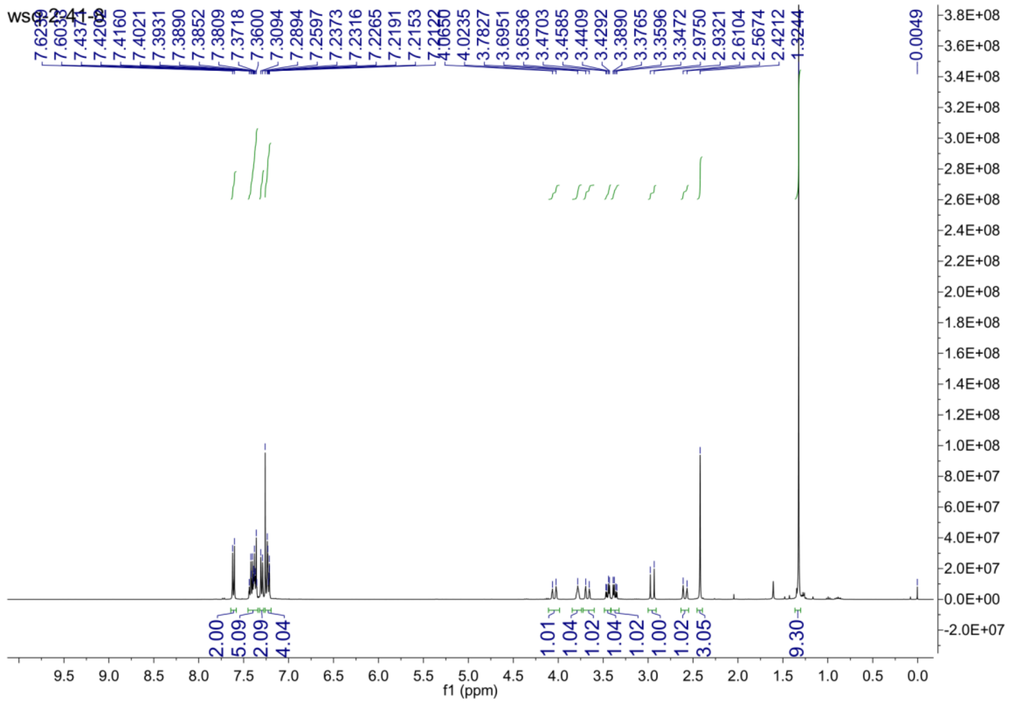
wsq-2-41-8
7.4160
7.4021
7.3931
7.3890
7.3852
7.3809
7.3718
7.3600
7.3094
7.2894
7.2597
7.2373
7.2316
7.2265
7.2191
7.2153
7.2122
4.0650
4.0235
3.7827
3.6951
3.6536
3.4703
3.4585
3.4409
3.4292
3.3890
3.3765
3.3596
3.3472
2.9750
2.9321
2.6104
2.5674
2.4212
1.3244
-0.0049
2.00
5.09
2.09
4.04
1.01
1.04
1.02
1.04
1.02
1.00
1.02
3.05
9.30
f1 (ppm)

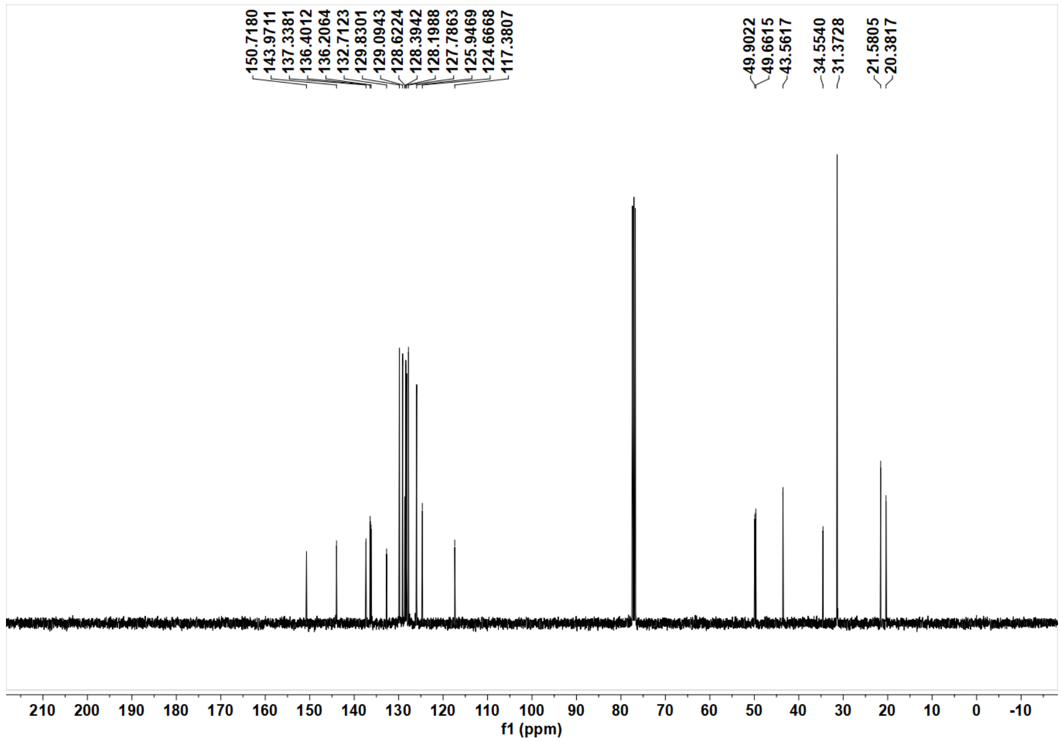
150.7180
143.9711
137.3381
136.4012
136.2064
132.7123
129.8301
129.0943
128.6224
128.3942
128.1988
127.7863
125.9469
124.6668
117.3807
49.9022
49.6615
43.5617
34.5540
31.3728
21.5805
20.3817
f1 (ppm)

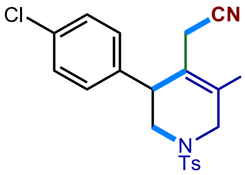

Cl
CN
Ph
N
Ts


**5c**

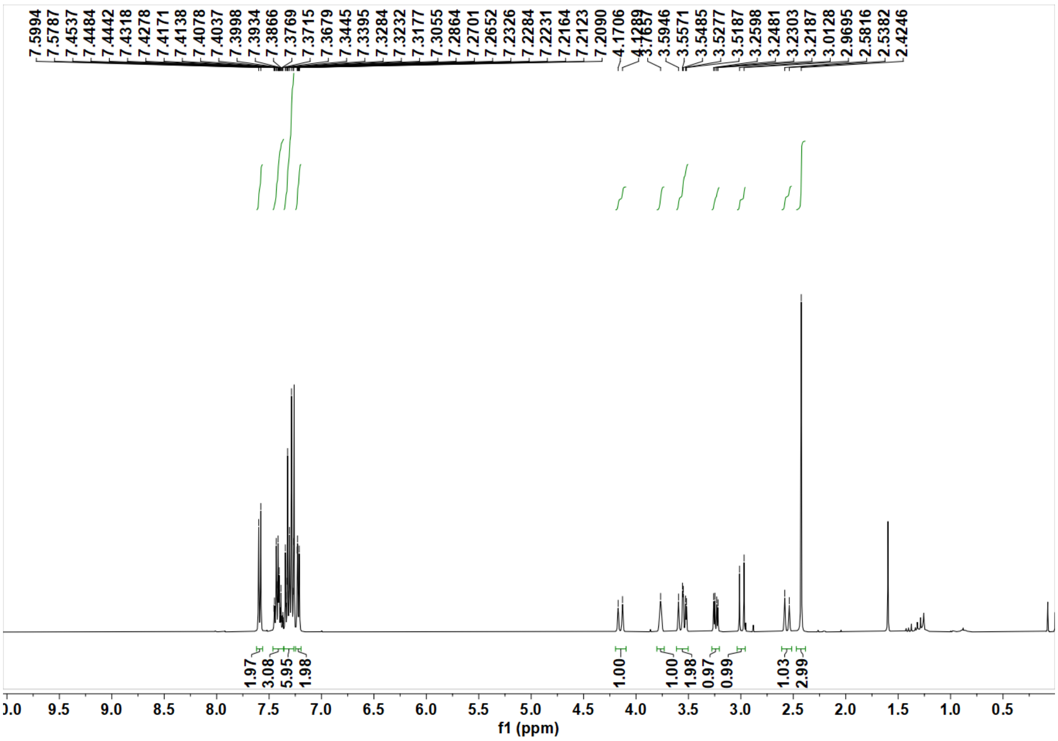

7.5994
7.5787
7.4537
7.4484
7.4442
7.4318
7.4278
7.4171
7.4138
7.4078
7.4037
7.3998
7.3934
7.3866
7.3769
7.3715
7.3679
7.3445
7.3395
7.3284
7.3232
7.3177
7.3055
7.2864
7.2701
7.2652
7.2326
7.2284
7.2231
7.2164
7.2123
7.2090
4.1706
3.5946
3.5571
3.5485
3.5277
3.5187
3.2598
3.2481
3.2303
3.2187
3.0128
2.9695
2.5816
2.5382
2.4246
1.97
3.08
5.95
1.98
1.00
1.00
1.98
0.97
0.99
1.03
2.99
f1 (ppm)


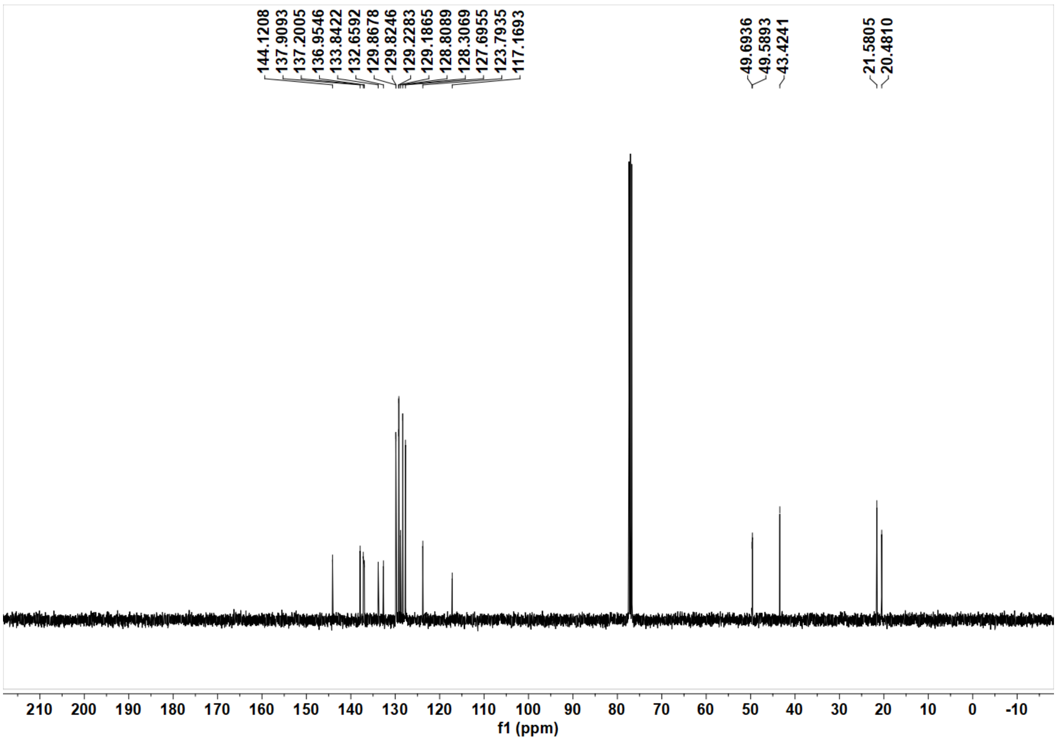

144.1208
137.9093
137.2005
136.9546
133.8422
132.6592
129.8678
129.8246
129.2283
129.1865
128.8089
128.3069
127.6955
123.7935
117.1693
49.6936
49.5893
43.4241
21.5805
20.4810
f1 (ppm)

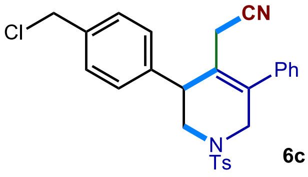
Cl
CN
Ph
N
Ts

**6c**

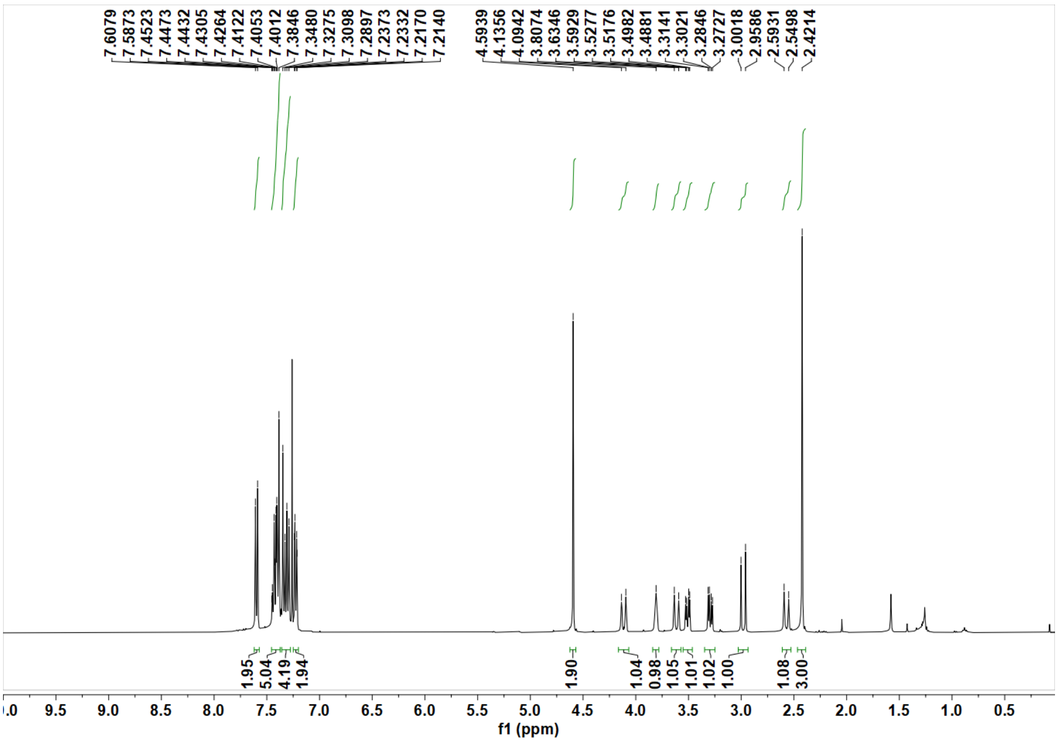
7.6079
7.5873
7.4523
7.4473
7.4432
7.4305
7.4264
7.4122
7.4053
7.4012
7.3846
7.3480
7.3275
7.3098
7.2897
7.2373
7.2332
7.2170
7.2140
4.5939
4.1356
4.0942
3.8074
3.6346
3.5929
3.5277
3.5176
3.4982
3.4881
3.3141
3.3021
3.2846
3.2727
3.0018
2.9586
2.5931
2.5498
2.4214
1.95
5.04
4.19
1.94
1.90
1.04
0.98
1.05
1.01
1.02
1.00
1.08
3.00
f1 (ppm)

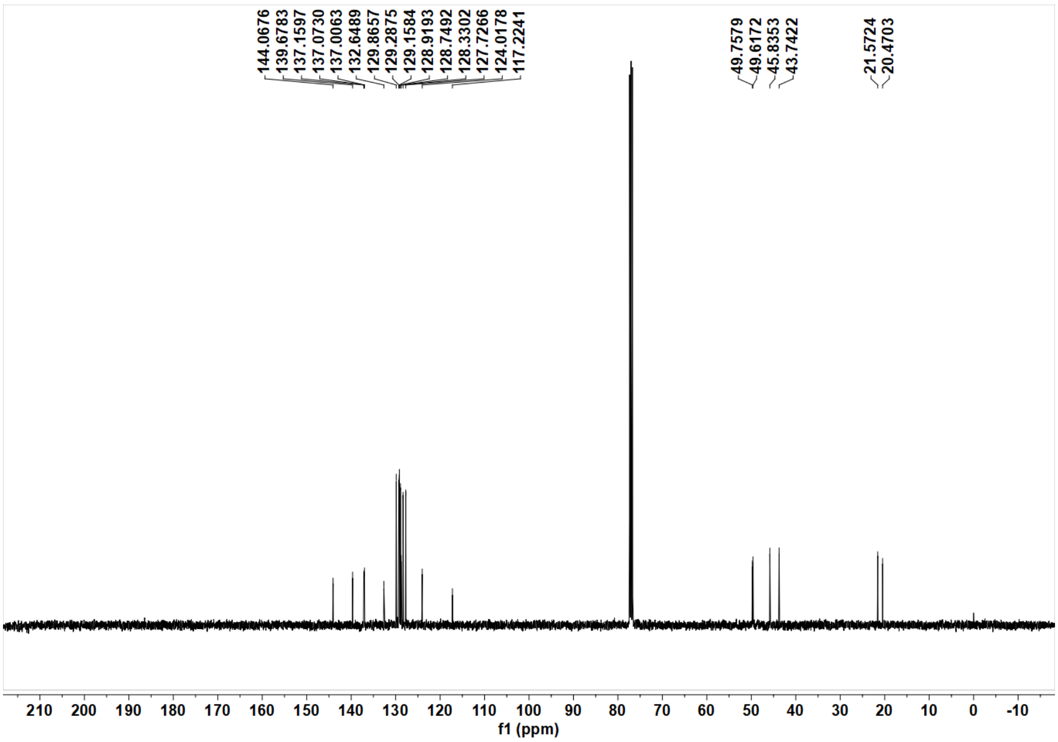
144.0676
139.6783
137.1597
137.0730
137.0063
132.6489
129.8657
129.2875
129.1584
128.9193
128.7492
128.3302
127.7266
124.0178
117.2241
49.7579
49.6172
45.8353
43.7422
21.5724
20.4703
f1 (ppm)

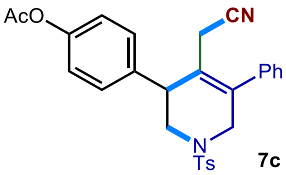

AcO
CN
Ph
N
Ts


**7c**

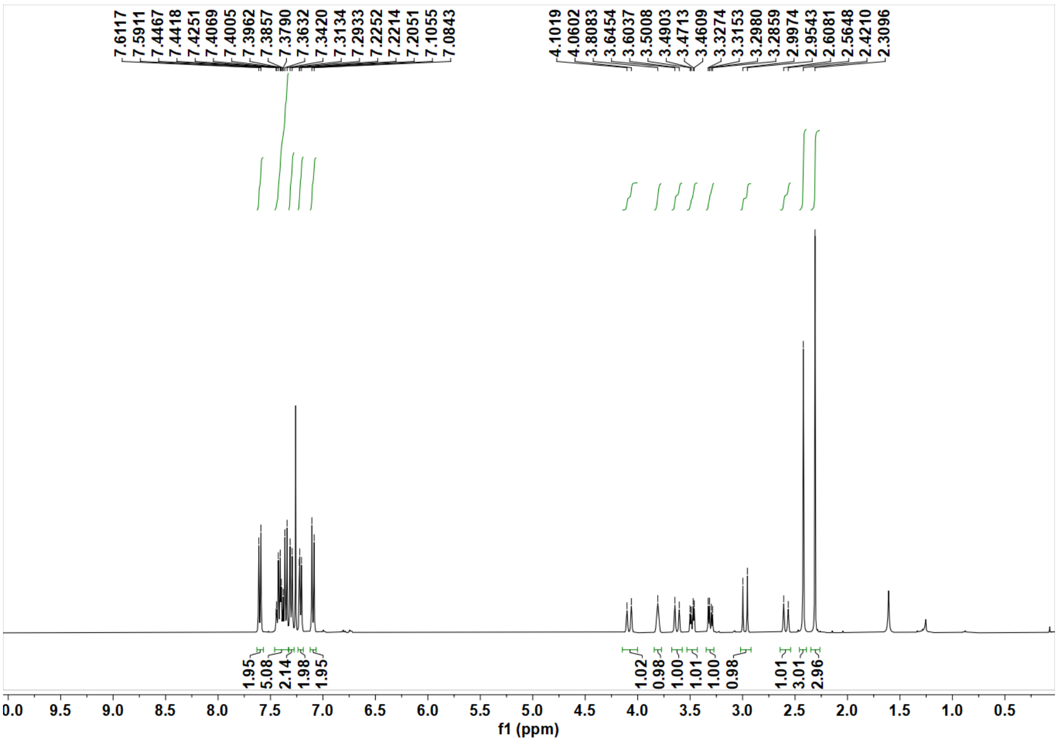

7.6117
7.5911
7.4467
7.4418
7.4251
7.4069
7.4005
7.3962
7.3857
7.3790
7.3632
7.3420
7.3134
7.2933
7.2252
7.2214
7.2051
7.1055
7.0843
4.1019
4.0602
3.8083
3.6454
3.6037
3.5008
3.4903
3.4713
3.4609
3.3274
3.3153
3.2980
3.2859
2.9974
2.9543
2.6081
2.5648
2.4210
2.3096
1.95
5.08
2.14
1.98
1.95
1.02
0.98
1.00
1.01
1.00
0.98
1.01
3.01
2.96
f1 (ppm)


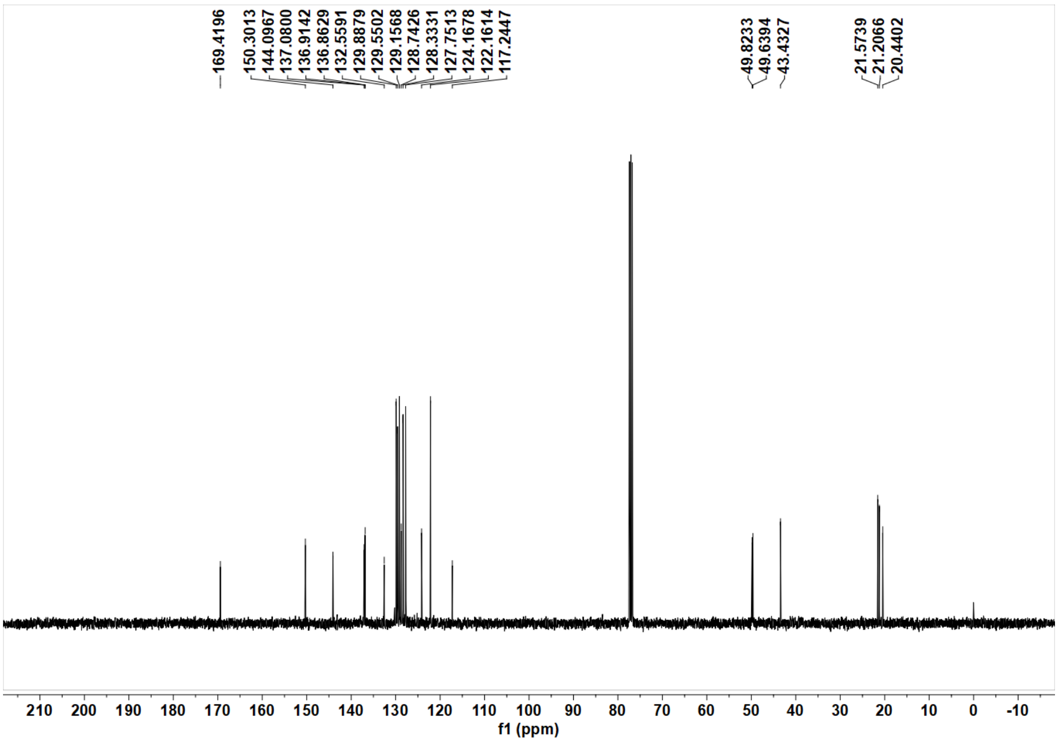

169.4196
150.3013
144.0967
137.0800
136.9142
136.8629
132.5591
129.8879
129.5502
129.1568
128.7426
128.3331
127.7513
124.1678
122.1614
117.2447
49.8233
49.6394
43.4327
21.5739
21.2066
20.4402
f1 (ppm)

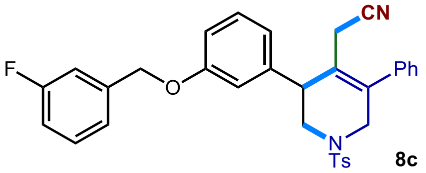
CN
Ph
F
O
N
Ts

**8c**

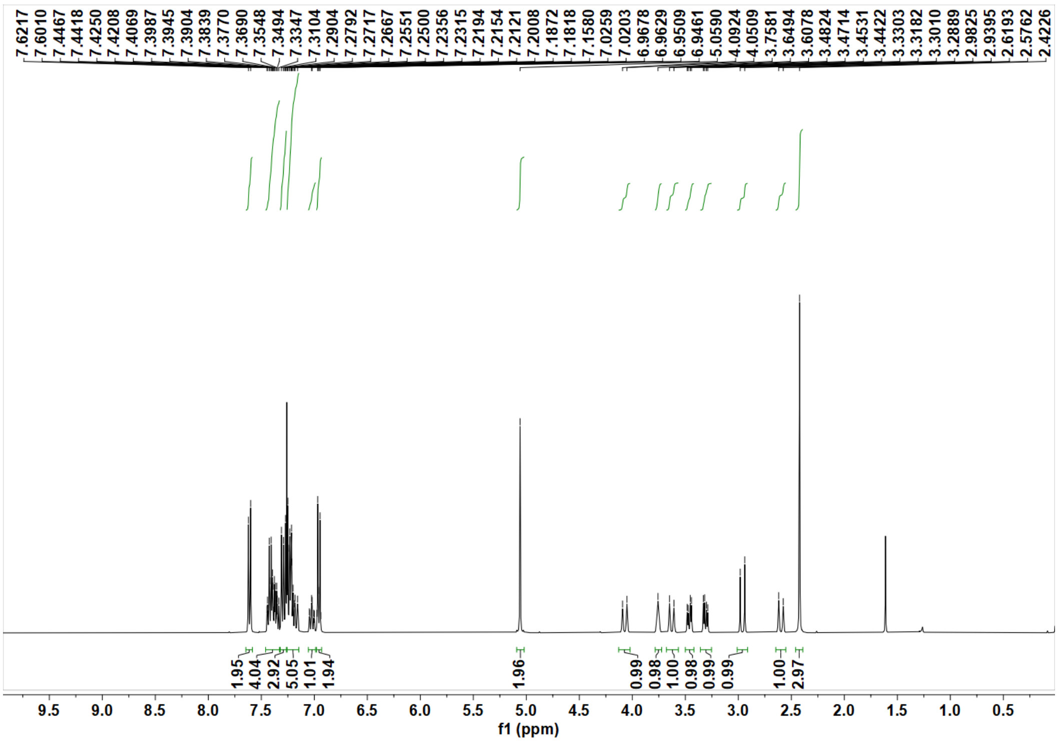
7.6217
7.6010
7.4467
7.4418
7.4250
7.4208
7.4069
7.3987
7.3945
7.3904
7.3839
7.3770
7.3690
7.3548
7.3494
7.3347
7.3104
7.2904
7.2792
7.2717
7.2667
7.2551
7.2500
7.2356
7.2315
7.2194
7.2154
7.2121
7.2008
7.1872
7.1818
7.1580
7.0259
7.0203
6.9678
6.9629
6.9509
6.9461
5.0590
4.0924
4.0509
3.7581
3.6494
3.6078
3.4824
3.4714
3.4531
3.4422
3.3303
3.3182
3.3010
3.2889
2.9825
2.9395
2.6193
2.5762
2.4226
1.95
4.04
2.92
5.05
1.01
1.94
1.96
0.99
0.98
1.00
0.98
0.99
0.99
1.00
2.97
9.5
9.0
8.5
8.0
7.5
7.0
6.5
6.0
5.5
5.0
4.5
4.0
3.5
3.0
2.5
2.0
1.5
1.0
0.5
f1 (ppm)

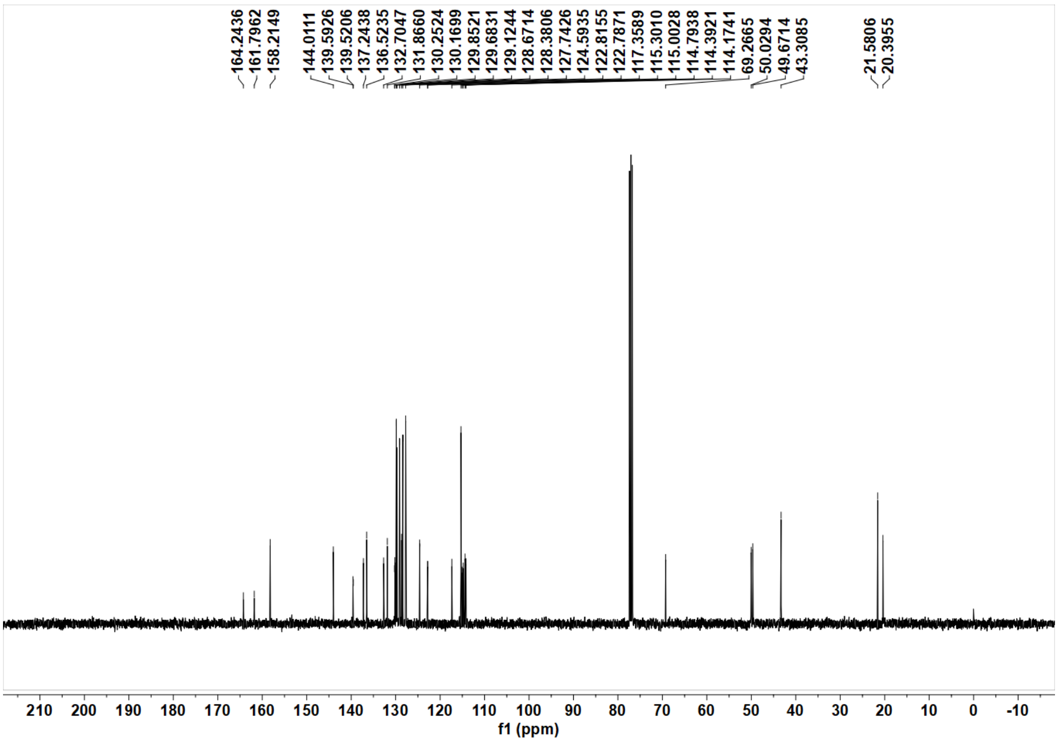
164.2436
161.7962
158.2149
144.0111
139.5926
139.5206
137.2438
136.5235
132.7047
131.8660
130.2524
130.1699
129.8521
129.6831
129.1244
128.3806
127.7426
124.5935
122.8155
122.7871
117.3589
115.3010
115.0028
114.7938
114.3921
114.1741
69.2665
50.0294
49.6714
43.3085
21.5806
20.3955
210
200
190
180
170
160
150
140
130
120
110
100
90
80
70
60
50
40
30
20
10
0
-10
f1 (ppm)

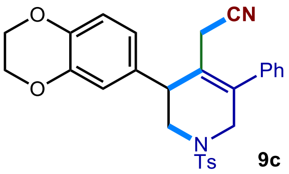
O
O
CN
Ph
N
Ts

**9c**

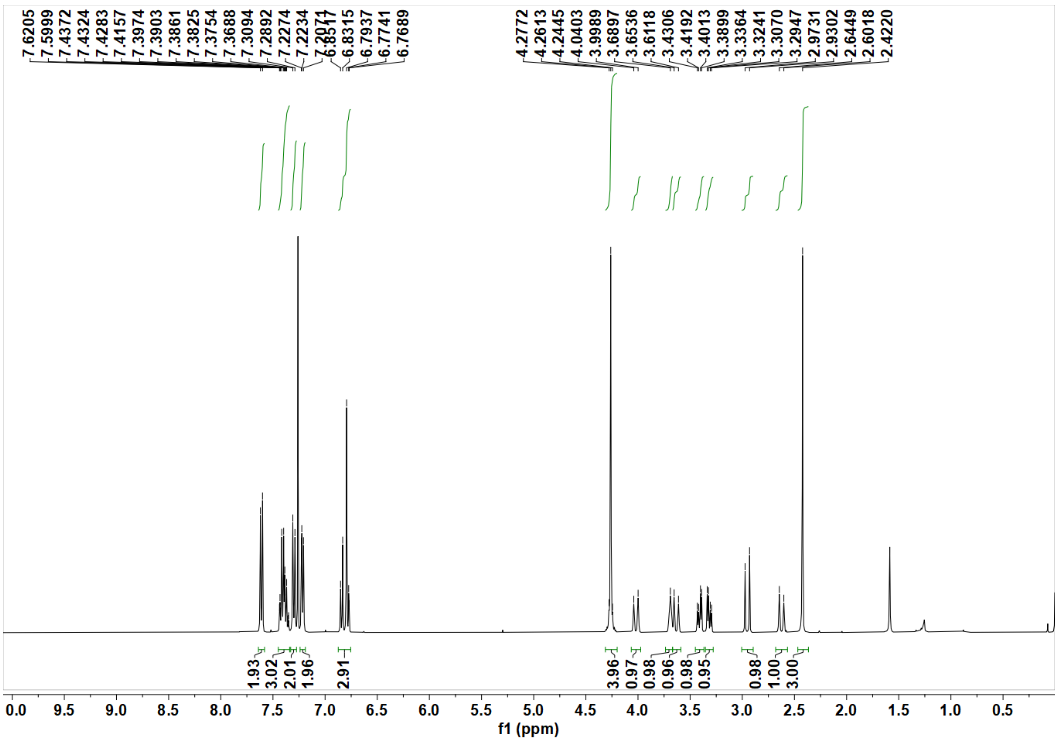
7.6205
7.5999
7.4372
7.4324
7.4283
7.4157
7.3974
7.3903
7.3861
7.3825
7.3754
7.3688
7.3094
7.2892
7.2274
7.2234
7.2071
6.8517
6.8315
6.7937
6.7741
6.7689
4.2772
4.2613
4.2445
4.0403
3.9989
3.6897
3.6536
3.6118
3.4306
3.4192
3.4013
3.3899
3.3364
3.3241
3.3070
3.2947
2.9731
2.9302
2.6449
2.6018
2.4220
1.93
3.02
2.01
1.96
2.91
3.96
0.97
0.98
0.96
0.98
0.95
0.98
1.00
3.00
f1 (ppm)

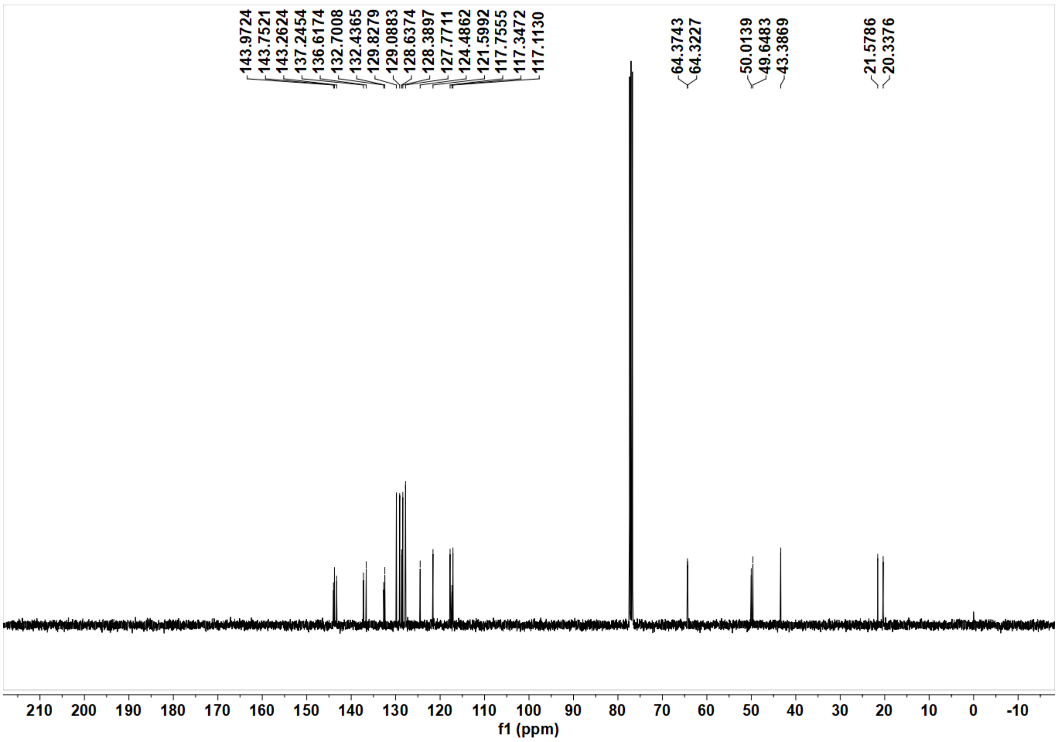
143.9724
143.7521
143.2624
137.2454
136.6174
132.7008
132.4365
129.8279
129.0883
128.6374
128.3897
127.7711
124.4862
121.5992
117.7555
117.3472
117.1130
64.3743
64.3227
50.0139
49.6483
43.3869
21.5786
20.3376
f1 (ppm)

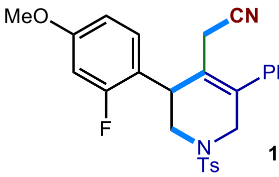

MeO
CN
Ph
F
N
Ts


**10c**

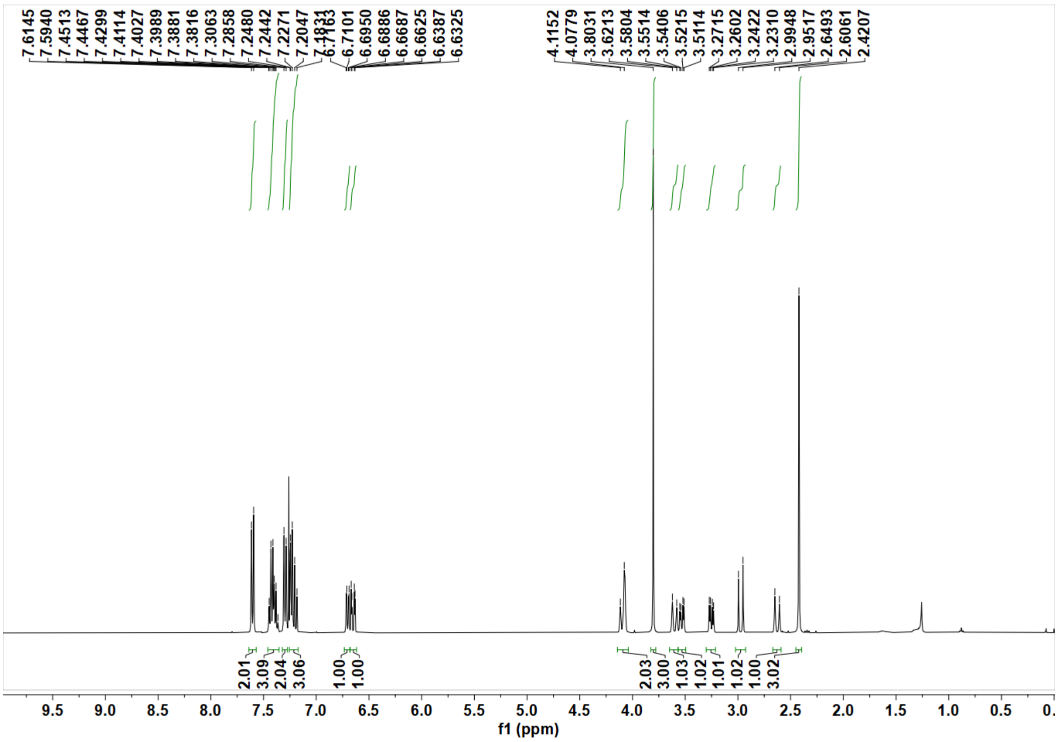

7.6145
7.5940
7.4513
7.4467
7.4299
7.4114
7.4027
7.3989
7.3881
7.3816
7.3063
7.2858
7.2480
7.2442
7.2271
7.2047
7.1831
6.7163
6.7101
6.6950
6.6886
6.6687
6.6625
6.6387
6.6325
4.1152
4.0779
3.8031
3.6213
3.5804
3.5514
3.5406
3.5215
3.5114
3.2715
3.2602
3.2422
3.2310
2.9948
2.9517
2.6493
2.6061
2.4207
2.01
3.09
2.04
3.06
1.00
1.00
2.03
3.00
1.03
1.02
1.01
1.02
1.00
3.02
9.5 9.0 8.5 8.0 7.5 7.0 6.5 6.0 5.5 5.0 4.5 4.0 3.5 3.0 2.5 2.0 1.5 1.0 0.5 0.
f1 (ppm)


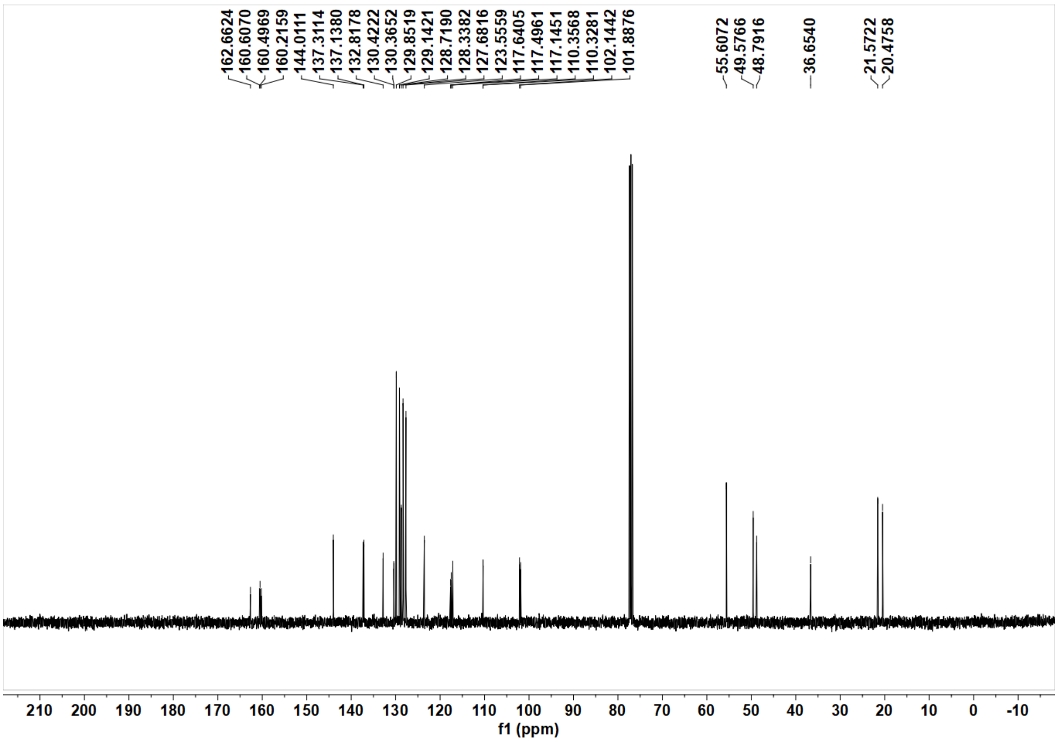

162.6624
160.6070
160.4969
160.2159
144.0111
137.3114
137.1380
132.8178
130.4222
130.3652
129.8519
129.1421
128.7190
128.3382
127.6816
123.5559
117.6405
117.4961
117.1451
110.3568
110.3281
102.1442
101.8876
55.6072
49.5766
48.7916
36.6540
21.5722
20.4758
210 200 190 180 170 160 150 140 130 120 110 100 90 80 70 60 50 40 30 20 10 0 -10
f1 (ppm)

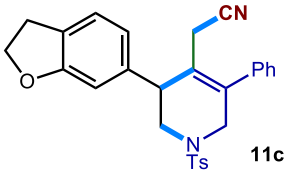

11c

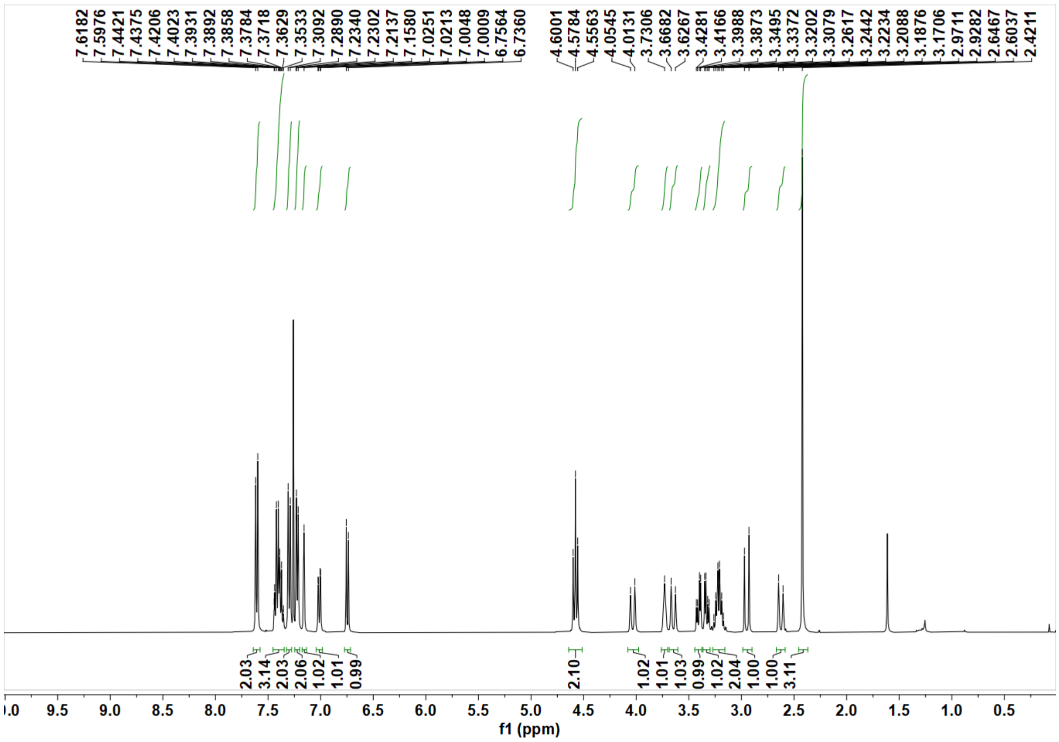

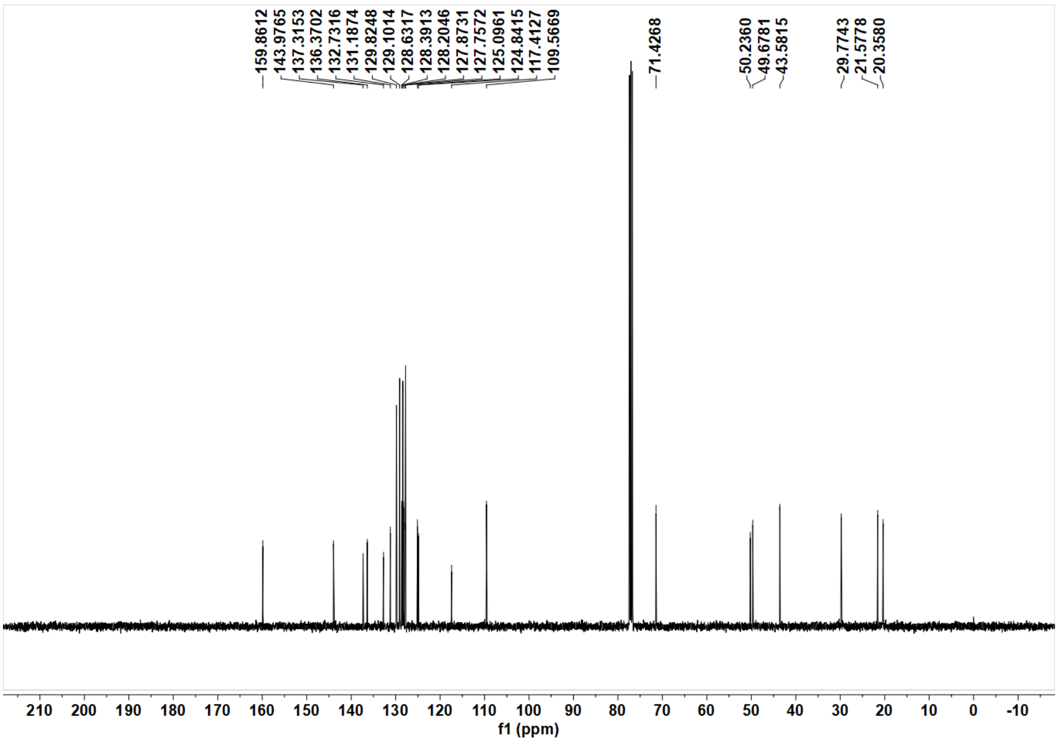

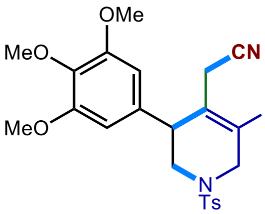


**12c**

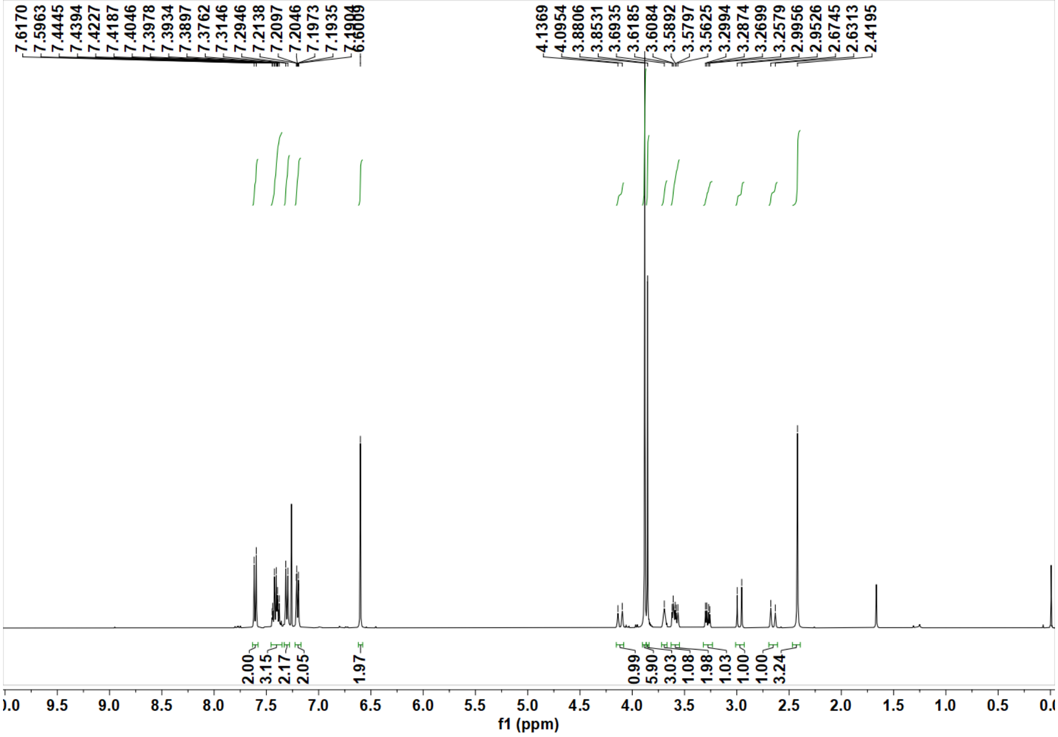


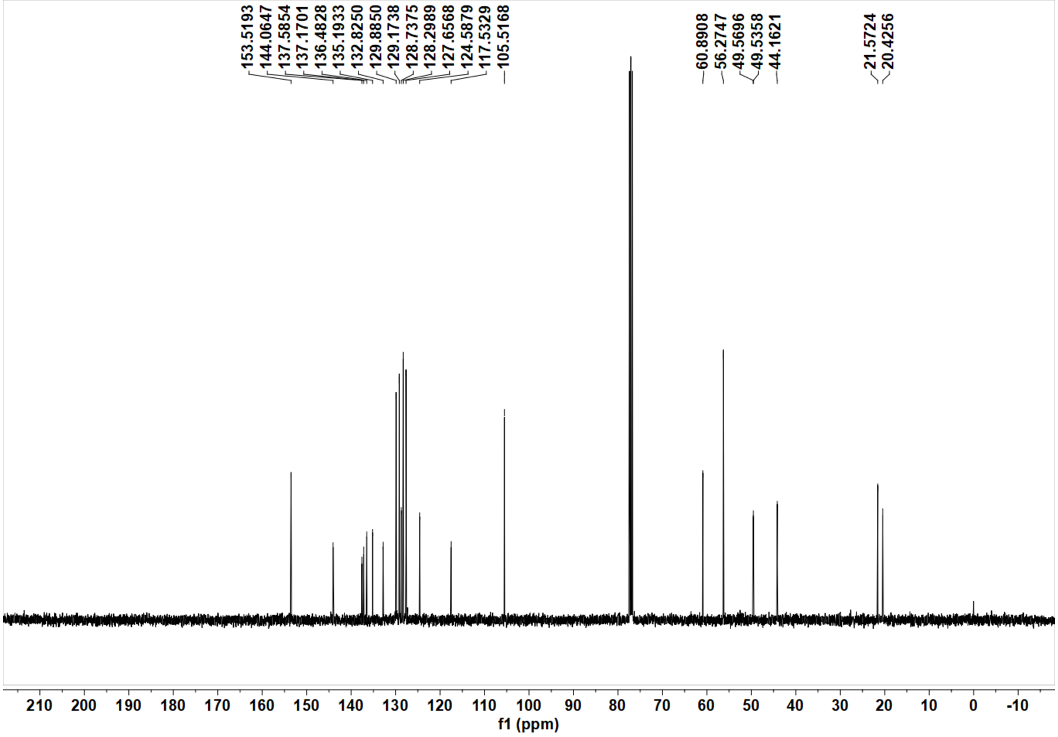

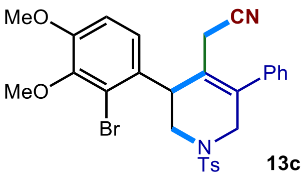


**13c**

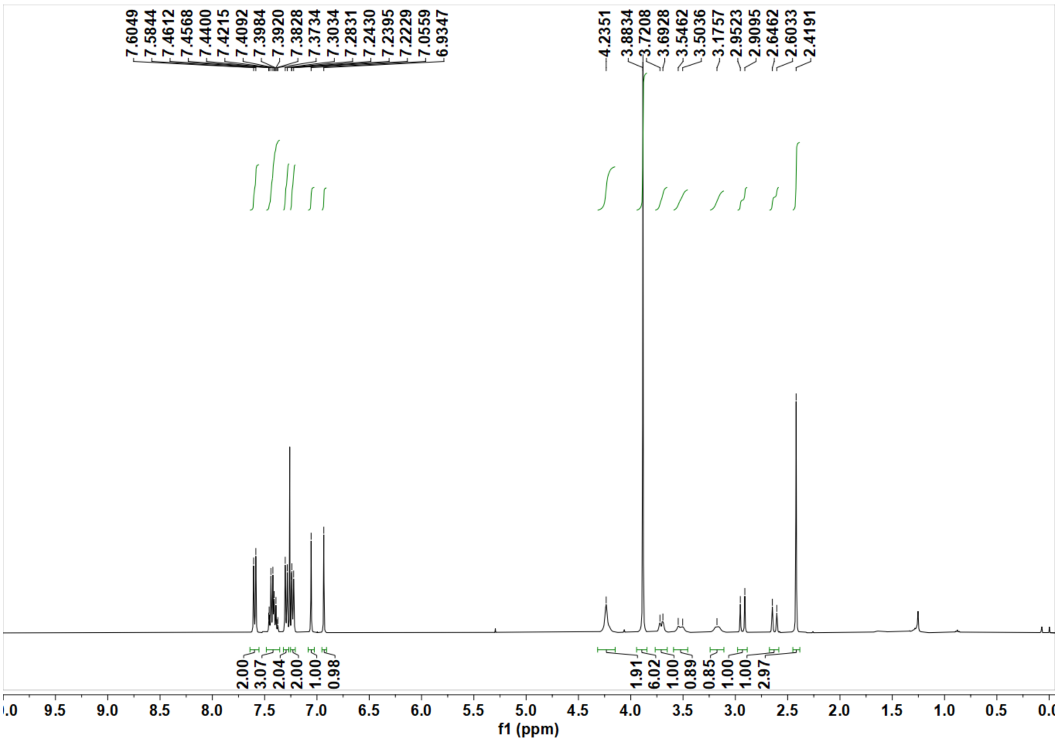

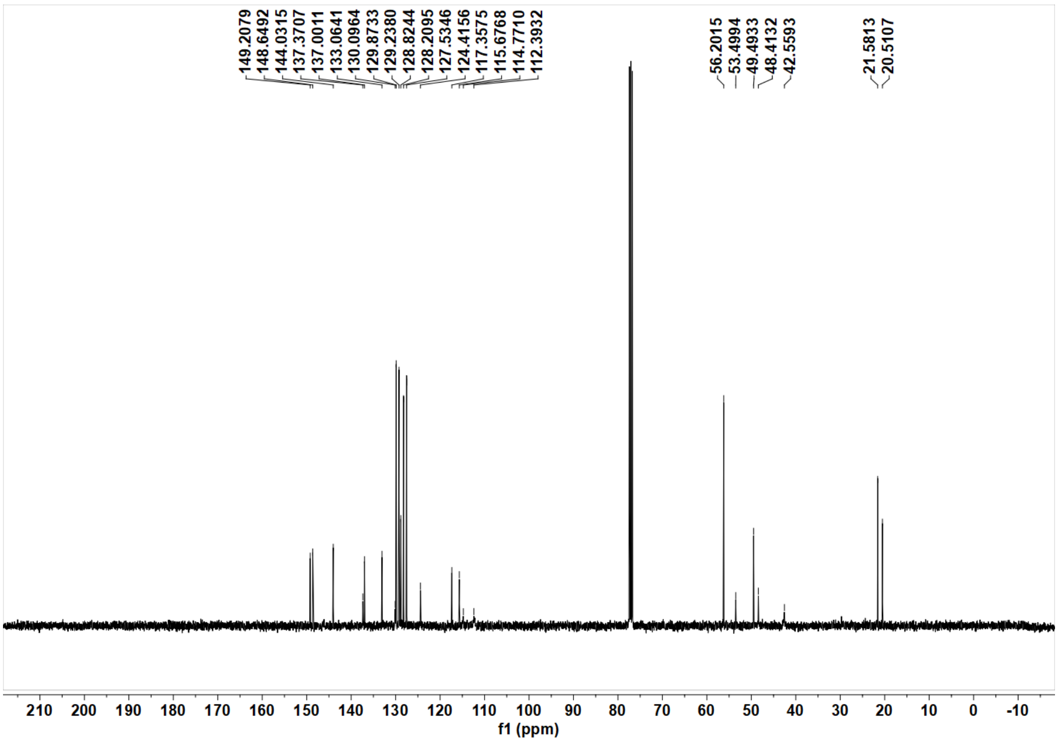

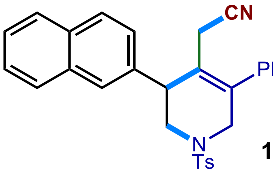
CN
Ph
N
Ts

**14c**

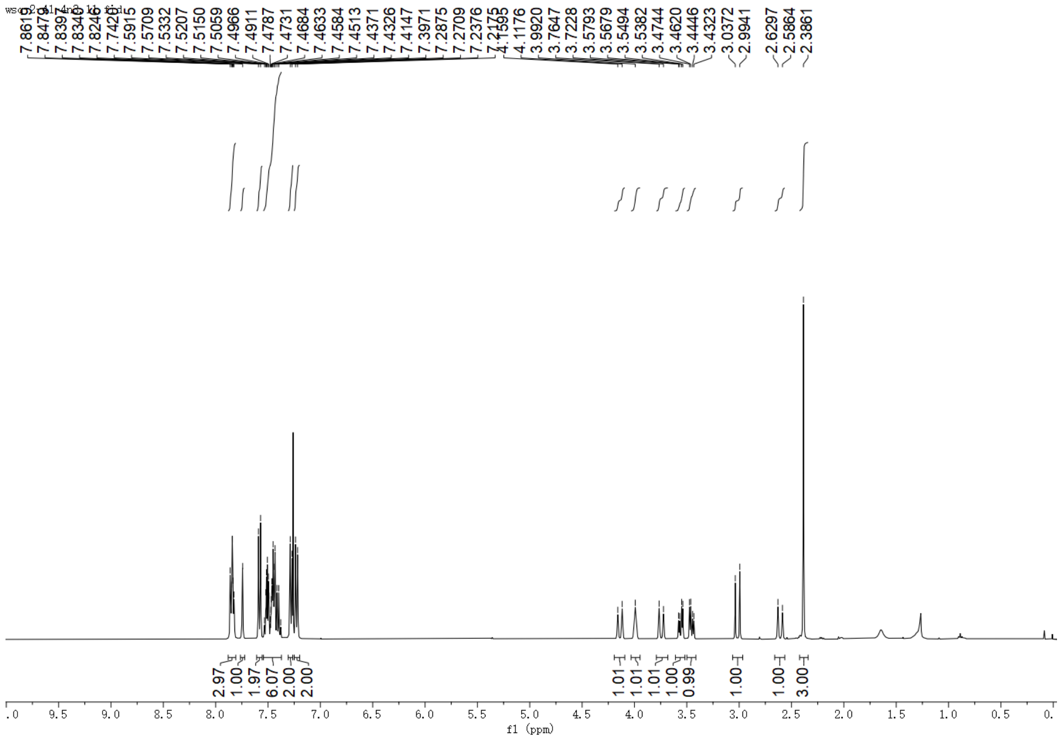
7.8619
7.8474
7.8394
7.8340
7.8246
7.7420
7.5915
7.5709
7.5332
7.5207
7.5150
7.5059
7.4966
7.4911
7.4787
7.4731
7.4684
7.4633
7.4584
7.4513
7.4371
7.4326
7.4147
7.3971
7.2875
7.2709
7.2376
7.2175
4.1595
4.1176
3.9920
3.7647
3.7228
3.5793
3.5679
3.5494
3.5382
3.4744
3.4620
3.4446
3.4323
3.0372
2.9941
2.6297
2.5864
2.3861
2.97
1.00
1.97
6.07
2.00
2.00
1.01
1.01
1.01
1.00
0.99
1.00
1.00
3.00
f1 (ppm)

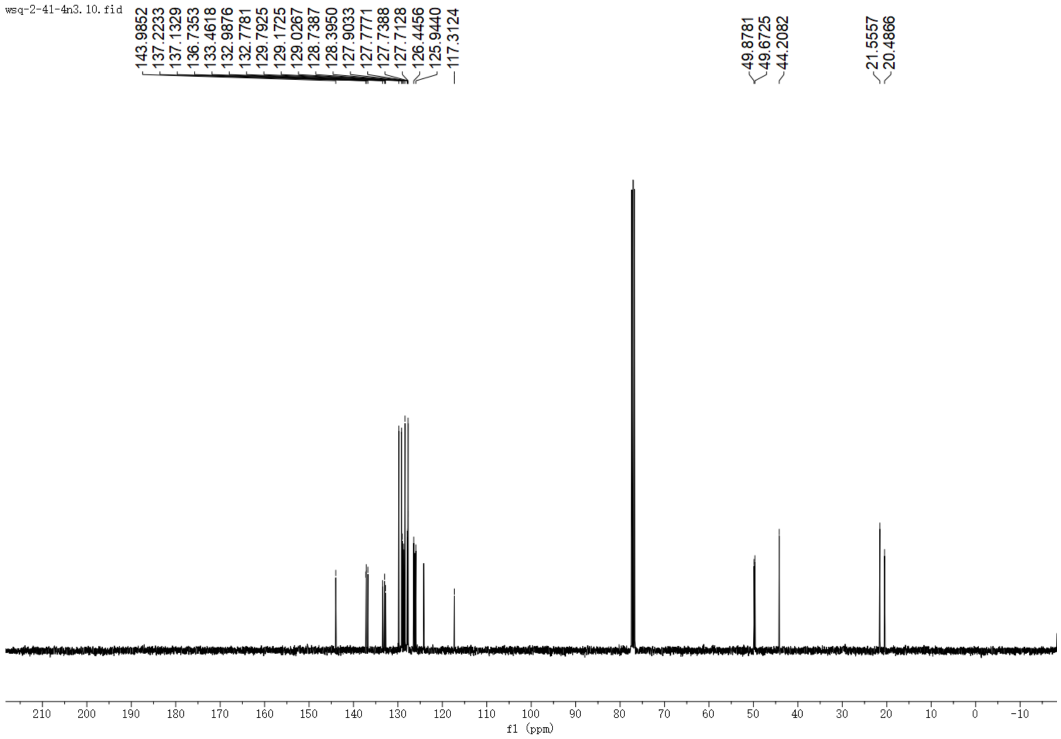
wsq-2-41-4n3.10.fid
143.9852
137.2233
137.1329
136.7353
133.4618
132.9876
132.7781
129.7925
129.1725
129.0267
128.7387
128.3950
127.9033
127.7771
127.7388
127.7128
126.4456
125.9440
117.3124
49.8781
49.6725
44.2082
21.5557
20.4866
f1 (ppm)

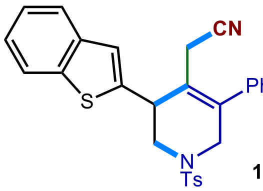
CN
Ph
S
N
Ts

**15c**

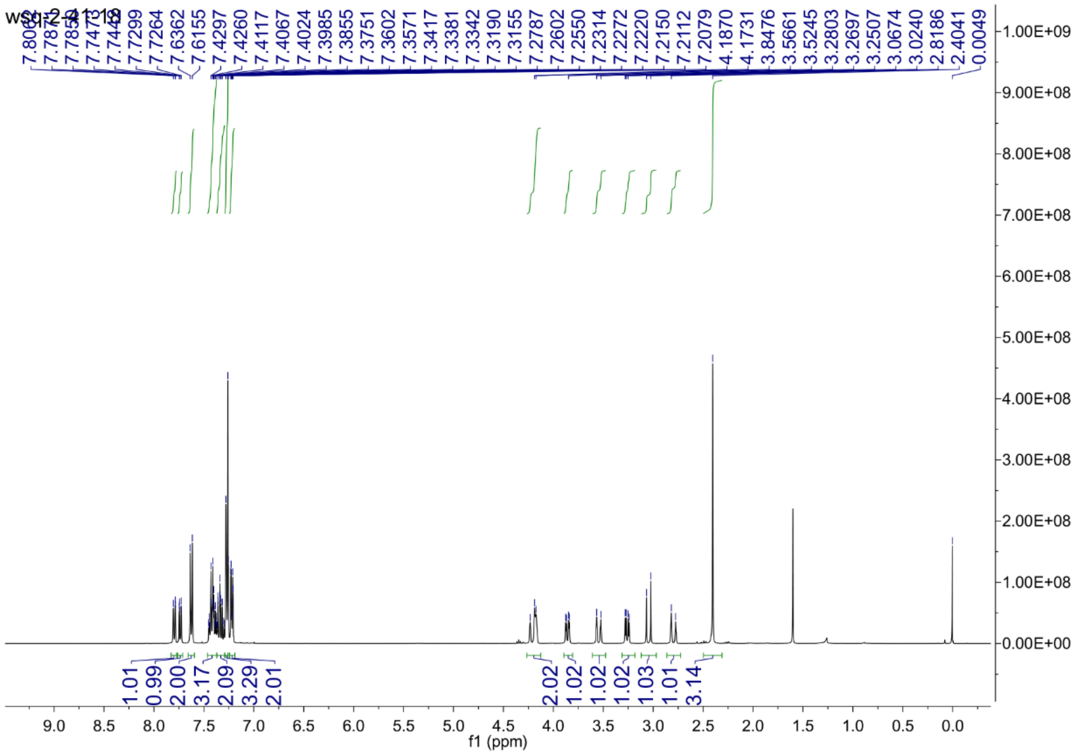
7.7299
7.7264
7.6362
7.6155
7.4297
7.4260
7.4117
7.4067
7.4024
7.3985
7.3855
7.3751
7.3602
7.3571
7.3417
7.3381
7.3342
7.3190
7.3155
7.2787
7.2602
7.2550
7.2314
7.2272
7.2220
7.2150
7.2112
7.2079
4.1870
4.1731
3.8476
3.5661
3.5245
3.2803
3.2697
3.2507
3.0674
3.0240
2.8186
2.4041
0.0049
1.01
0.99
2.00
3.17
2.09
3.29
2.01
2.02
1.02
1.02
1.02
1.03
1.01
3.14
f1 (ppm)

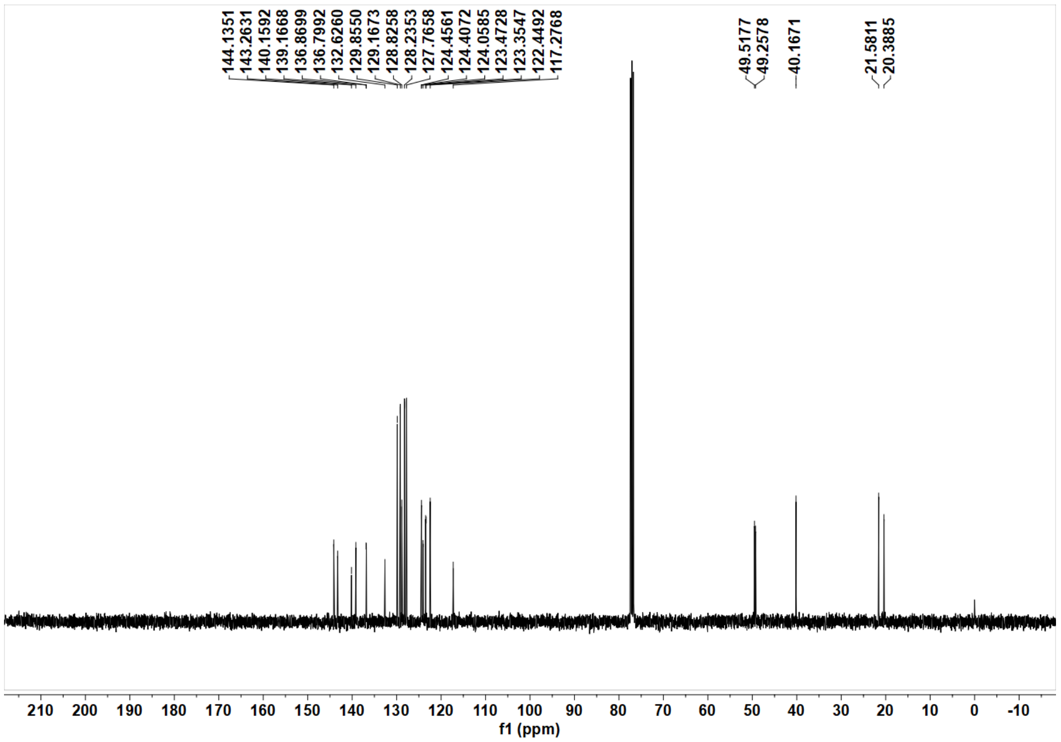
144.1351
143.2631
140.1592
139.1668
136.8699
136.7992
132.6260
129.8550
129.1673
128.8258
128.2353
127.7658
124.4561
124.4072
124.0585
123.4728
123.3547
122.4492
117.2768
49.5177
49.2578
40.1671
21.5811
20.3885
f1 (ppm)

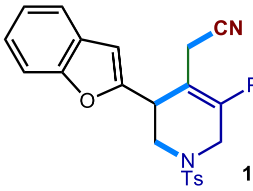
CN
Ph
O
N
Ts

**16c**

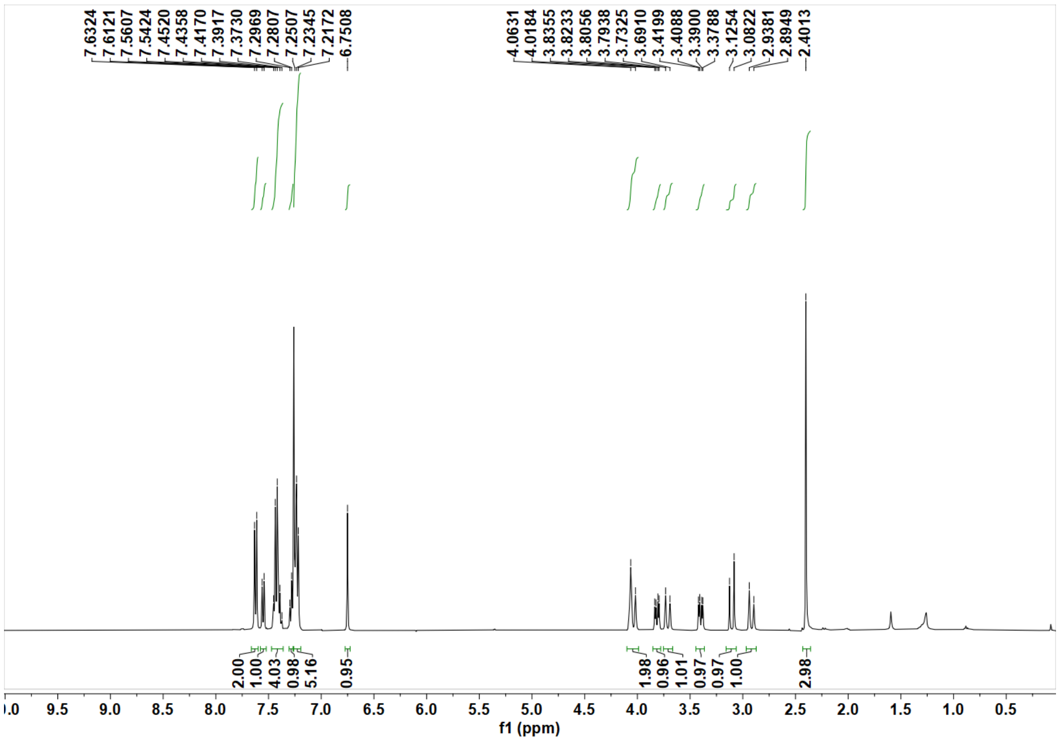
7.6324
7.6121
7.5607
7.5424
7.4520
7.4358
7.4170
7.3917
7.3730
7.2969
7.2807
7.2507
7.2345
7.2172
6.7508
4.0631
4.0184
3.8355
3.8233
3.8056
3.7938
3.7325
3.6910
3.4199
3.4088
3.3900
3.3788
3.1254
3.0822
2.9381
2.8949
2.4013
2.00
1.00
4.03
0.98
5.16
0.95
1.98
0.96
1.01
0.97
0.97
1.00
2.98
f1 (ppm)

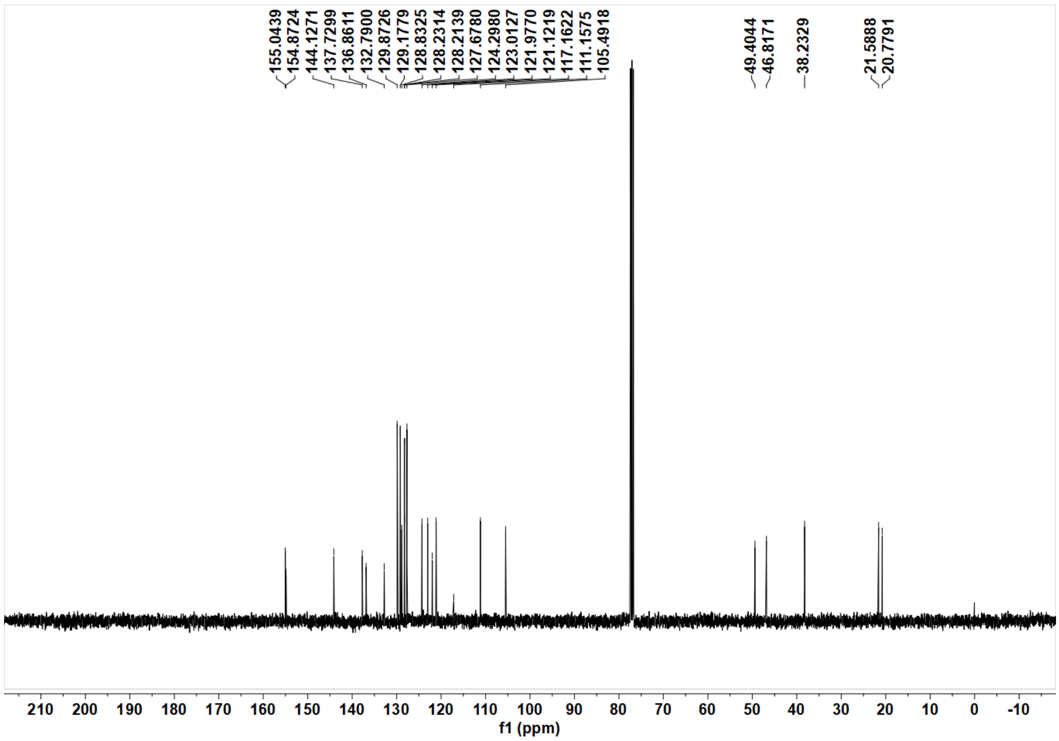
155.0439
154.8724
144.1271
137.7299
136.8611
132.7900
129.8726
129.1779
128.8325
128.2314
128.2139
127.6780
124.2980
123.0127
121.9770
121.1219
117.1622
111.1575
105.4918
49.4044
46.8171
38.2329
21.5888
20.7791
f1 (ppm)

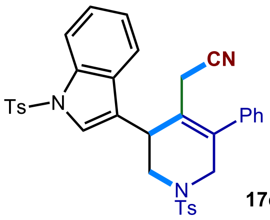
Ts
N
CN
Ph
N
Ts

**17c**

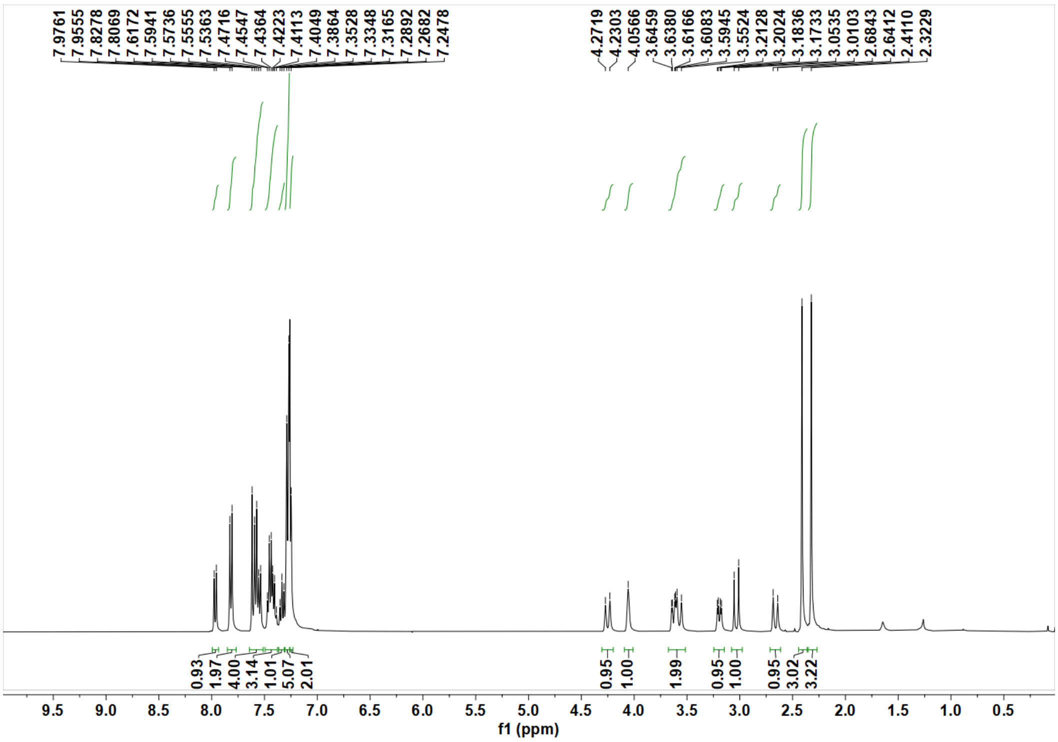
7.9761
7.9555
7.8278
7.8069
7.6172
7.5941
7.5736
7.5555
7.5363
7.4716
7.4547
7.4364
7.4223
7.4113
7.4049
7.3864
7.3528
7.3348
7.3165
7.2892
7.2682
7.2478
4.2719
4.2303
4.0566
3.6459
3.6380
3.6166
3.6083
3.5945
3.5524
3.2128
3.2024
3.1836
3.1733
3.0535
3.0103
2.6843
2.6412
2.4110
2.3229
0.93
1.97
4.00
3.14
1.01
5.07
2.01
0.95
1.00
1.99
0.95
1.00
0.95
3.02
3.22
9.5 9.0 8.5 8.0 7.5 7.0 6.5 6.0 5.5 5.0 4.5 4.0 3.5 3.0 2.5 2.0 1.5 1.0 0.5
f1 (ppm)

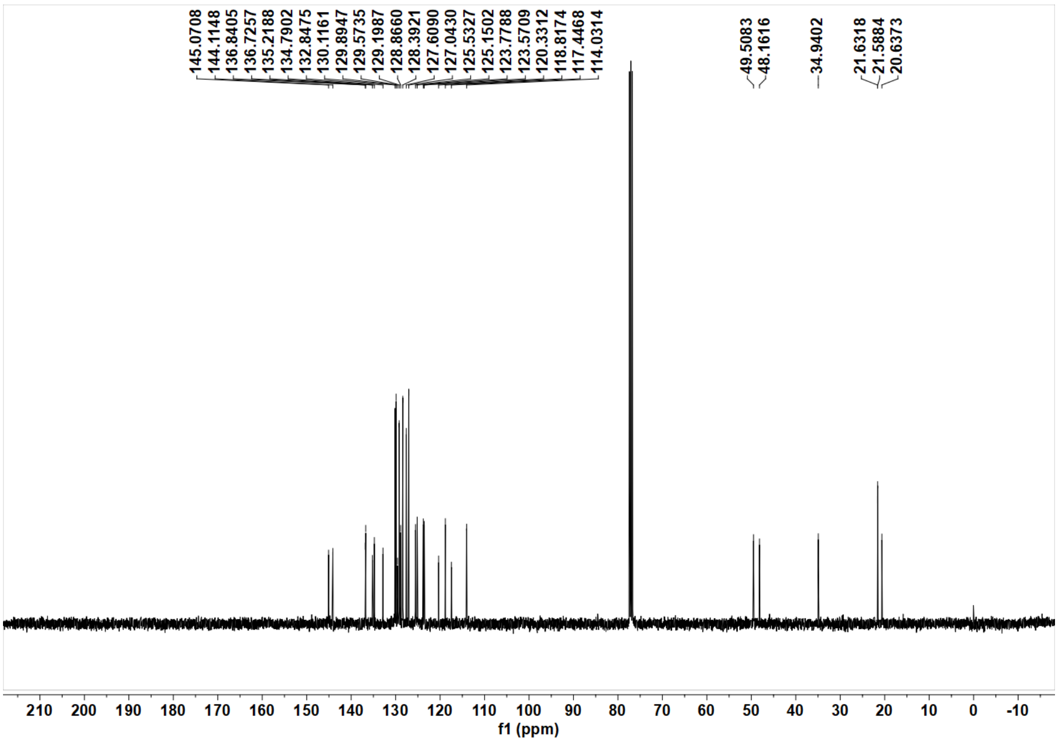
145.0708
144.1148
136.8405
136.7257
135.2188
134.7902
132.8475
130.1161
129.8947
129.5735
129.1987
128.8660
128.3921
127.6090
127.0430
125.5327
125.1502
123.7788
123.5709
120.3312
118.8174
117.4468
114.0314
49.5083
48.1616
34.9402
21.6318
21.5884
20.6373
210 200 190 180 170 160 150 140 130 120 110 100 90 80 70 60 50 40 30 20 10 0 -10
f1 (ppm)

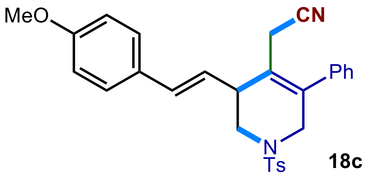
MeO
CN
Ph
N
Ts

**18c**

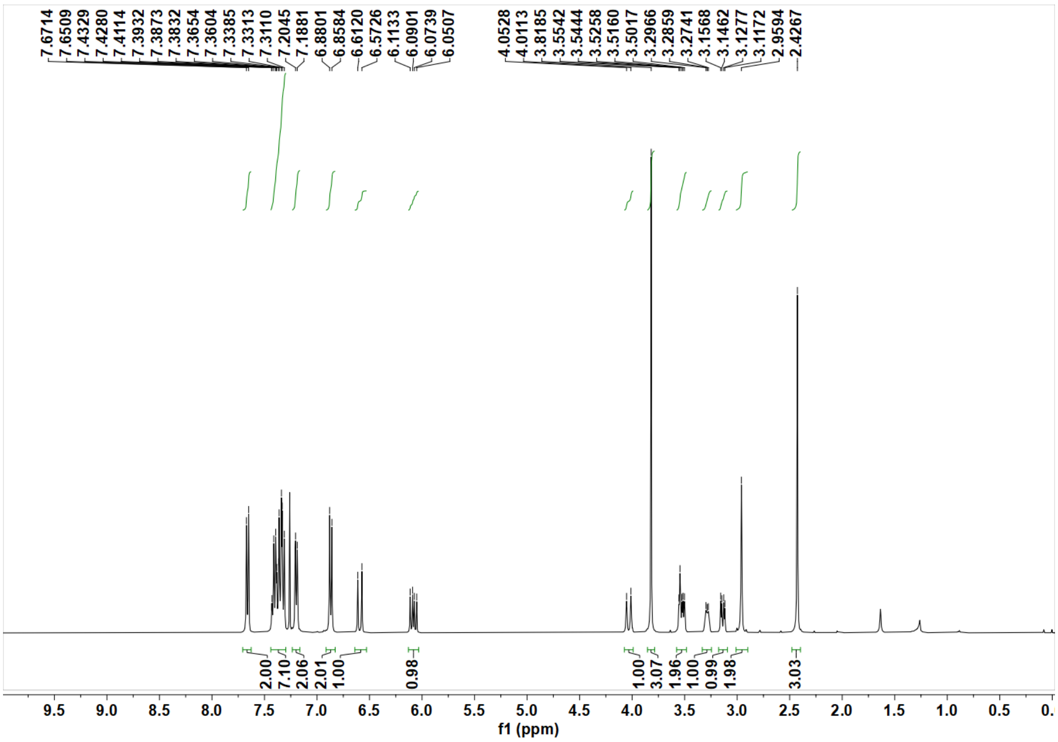
7.6714
7.6509
7.4329
7.4280
7.4114
7.3932
7.3873
7.3832
7.3654
7.3604
7.3385
7.3313
7.3110
7.2045
7.1881
6.8801
6.8584
6.6120
6.5726
6.1133
6.0901
6.0739
6.0507
4.0528
4.0113
3.8185
3.5542
3.5444
3.5258
3.5160
3.5017
3.2966
3.2859
3.2741
3.1568
3.1462
3.1277
3.1172
2.9594
2.4267
2.00
7.10
2.06
2.01
1.00
0.98
1.00
3.07
1.96
1.00
0.99
1.98
3.03
9.5
9.0
8.5
8.0
7.5
7.0
6.5
6.0
5.5
5.0
4.5
4.0
3.5
3.0
2.5
2.0
1.5
1.0
0.5
0.
f1 (ppm)

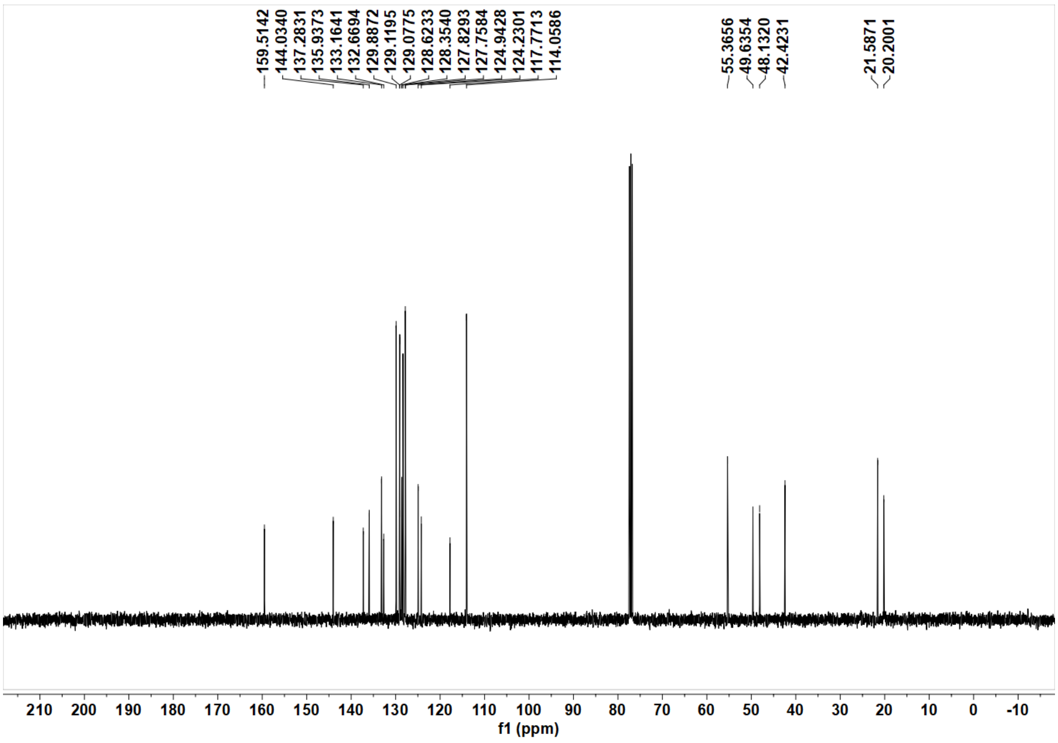
159.5142
144.0340
137.2831
135.9373
133.1641
132.6694
129.8872
129.1195
129.0775
128.6233
128.3540
127.8293
127.7584
124.9428
124.2301
117.7713
114.0586
55.3656
49.6354
48.1320
42.4231
21.5871
20.2001
210
200
190
180
170
160
150
140
130
120
110
100
90
80
70
60
50
40
30
20
10
0
-10
f1 (ppm)

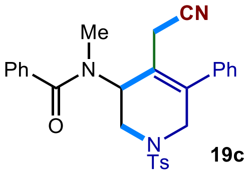
CN
Me
Ph
N
Ph
O
N
Ts

**19c**

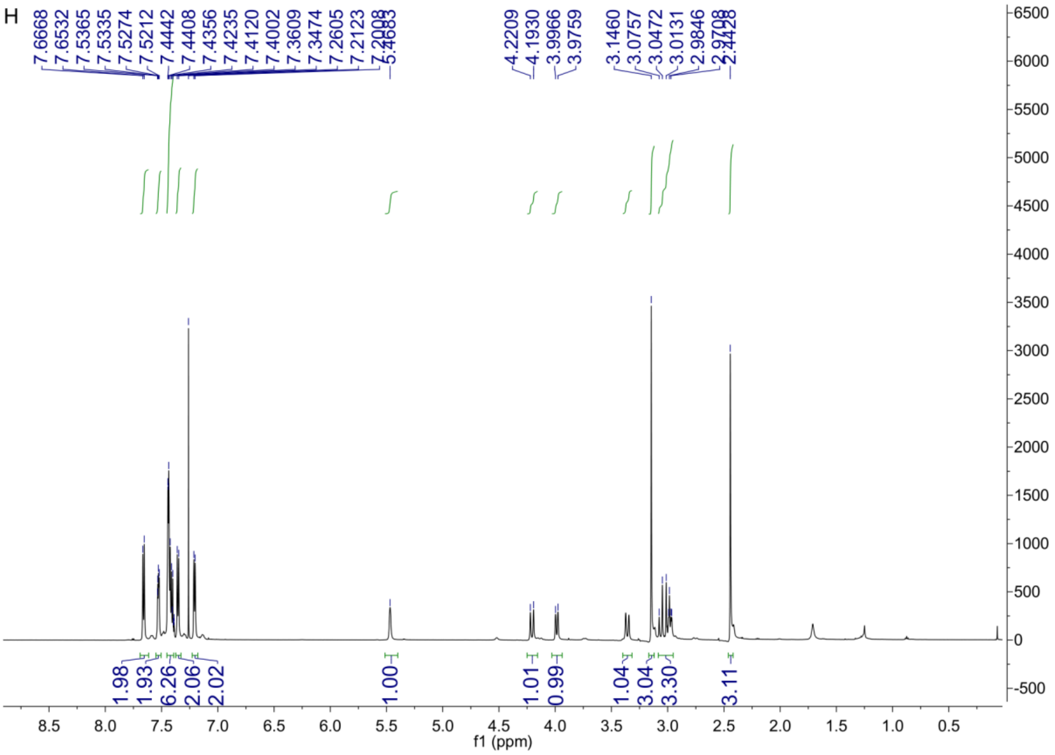
H
7.6668
7.6532
7.5365
7.5335
7.5274
7.5212
7.4442
7.4408
7.4356
7.4235
7.4120
7.4002
7.3609
7.3474
7.2605
7.2123
7.2008
5.4683
4.2209
4.1930
3.9966
3.9759
3.1460
3.0757
3.0472
3.0131
2.9846
2.9708
2.4428
1.98
1.93
6.26
2.06
2.02
1.00
1.01
0.99
1.04
3.04
3.30
3.11
f1 (ppm)

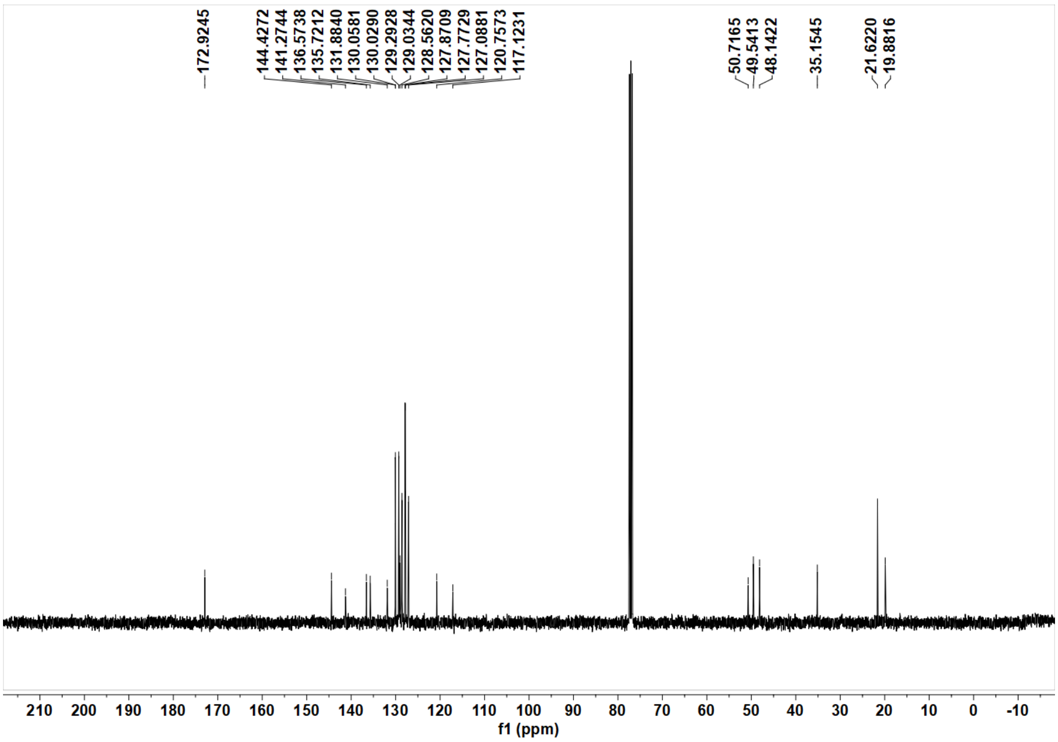
172.9245
144.4272
141.2744
136.5738
135.7212
131.8840
130.0581
130.0290
129.2928
129.0344
128.5620
127.8709
127.7729
127.0881
120.7573
117.1231
50.7165
49.5413
48.1422
35.1545
21.6220
19.8816
f1 (ppm)

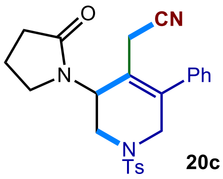

O
CN
N
Ph
N
Ts


**20c**

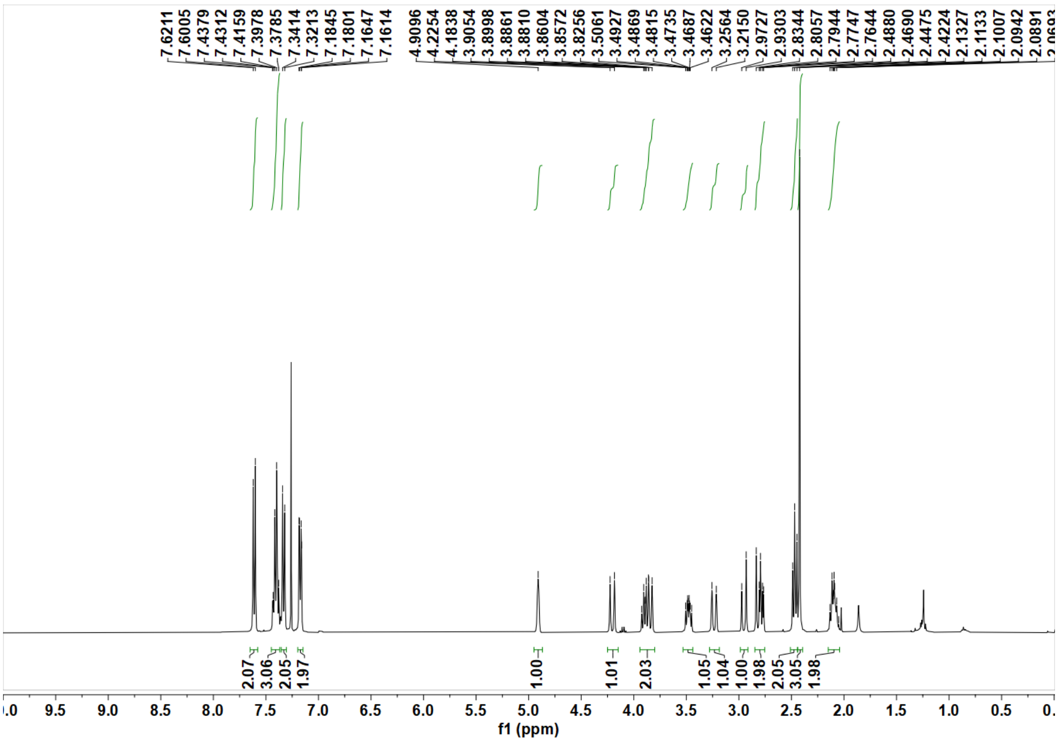

7.6211
7.6005
7.4379
7.4312
7.4159
7.3978
7.3785
7.3414
7.3213
7.1845
7.1801
7.1647
7.1614
4.9096
4.2254
4.1838
3.9054
3.8998
3.8861
3.8810
3.8604
3.8572
3.8256
3.5061
3.4927
3.4869
3.4815
3.4735
3.4687
3.4622
3.2564
3.2150
2.9727
2.9303
2.8344
2.8057
2.7944
2.7747
2.7644
2.4880
2.4690
2.4475
2.4224
2.1327
2.1133
2.1007
2.0942
2.0891
2.07
3.06
2.05
1.97
1.00
1.01
2.03
1.05
1.04
1.00
1.98
2.05
3.05
1.98
9.5
9.0
8.5
8.0
7.5
7.0
6.5
6.0
5.5
5.0
4.5
4.0
3.5
3.0
2.5
2.0
1.5
1.0
0.5
f1 (ppm)


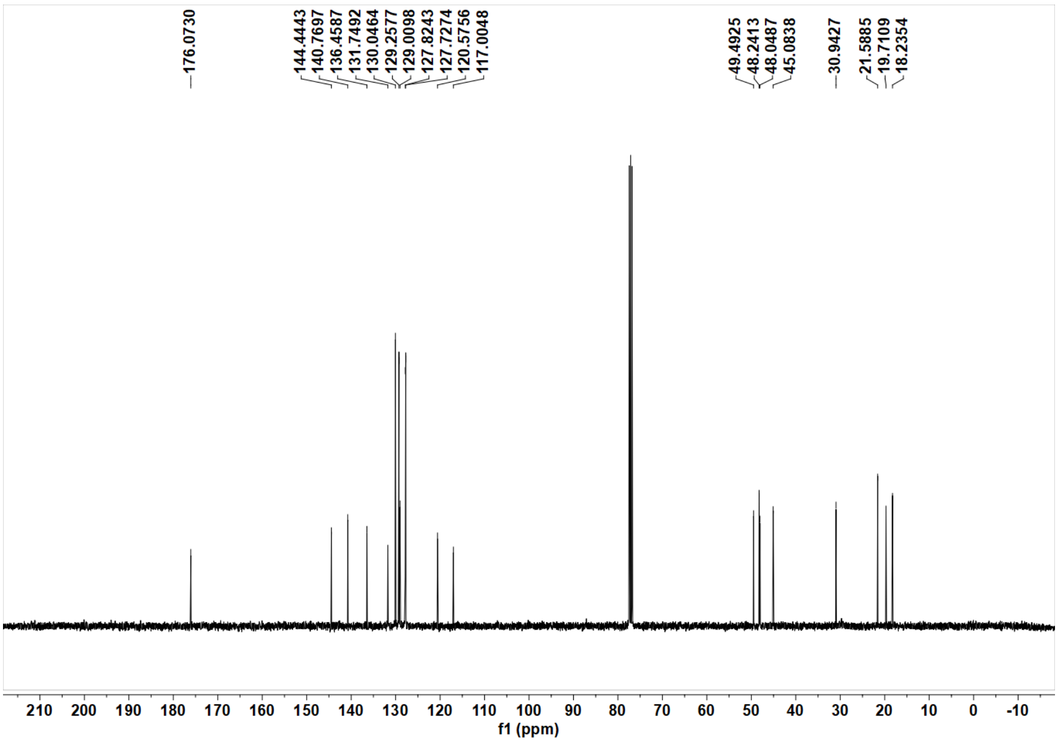

176.0730
144.4443
140.7697
136.4587
131.7492
130.0464
129.2577
129.0098
127.8243
127.7274
120.5756
117.0048
49.4925
48.2413
48.0487
45.0838
30.9427
21.5885
19.7109
18.2354
210
200
190
180
170
160
150
140
130
120
110
100
90
80
70
60
50
40
30
20
10
0
-10
f1 (ppm)

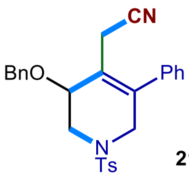
CN
BnO
Ph
N
Ts

**21c**

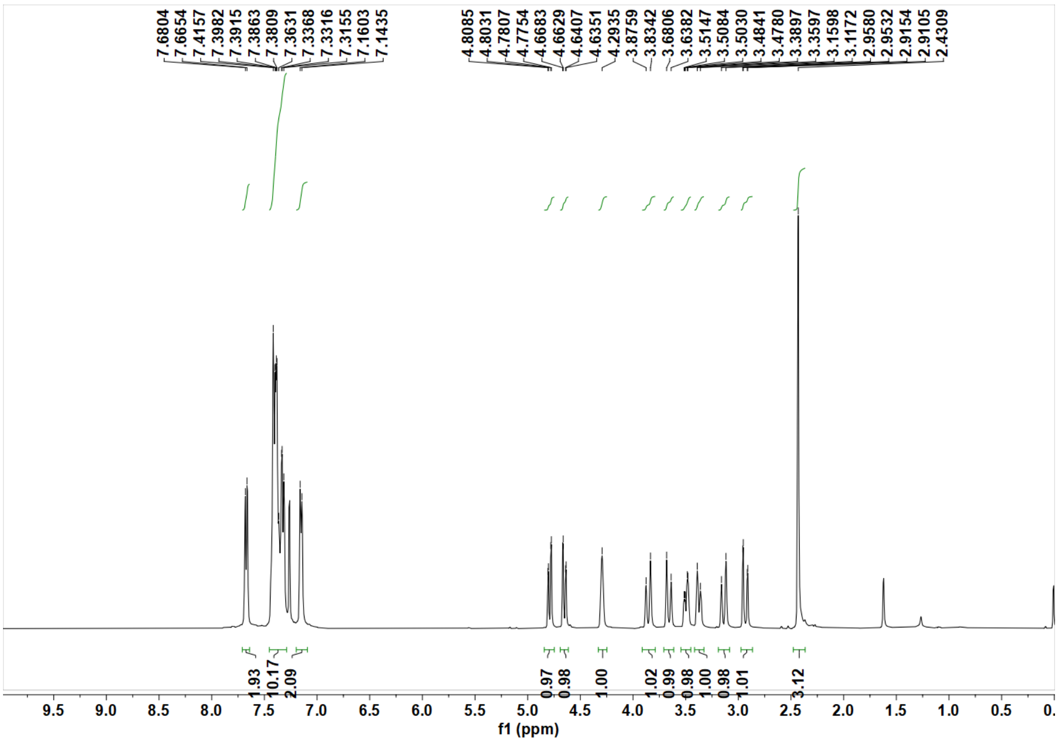
7.6804
7.6654
7.4157
7.3982
7.3915
7.3863
7.3809
7.3631
7.3368
7.3316
7.3155
7.1603
7.1435
4.8085
4.8031
4.7807
4.7754
4.6683
4.6629
4.6407
4.6351
4.2935
3.8759
3.8342
3.6806
3.6382
3.5147
3.5084
3.5030
3.4841
3.4780
3.3897
3.3597
3.1598
3.1172
2.9580
2.9532
2.9154
2.9105
2.4309
1.93
10.17
2.09
0.97
0.98
1.00
1.02
0.99
0.98
1.00
0.98
1.01
3.12
9.5 9.0 8.5 8.0 7.5 7.0 6.5 6.0 5.5 5.0 4.5 4.0 3.5 3.0 2.5 2.0 1.5 1.0 0.5 0.
f1 (ppm)

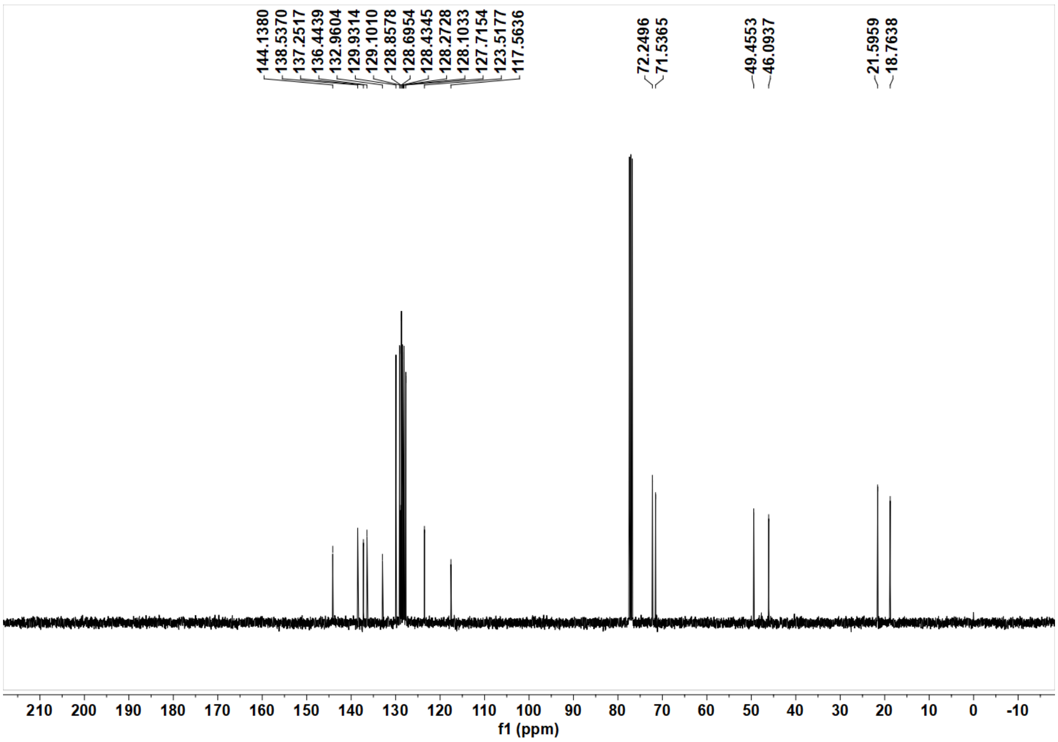
144.1380
138.5370
137.2517
136.4439
132.9604
129.9314
129.1010
128.8578
128.6954
128.4345
128.2728
128.1033
127.7154
123.5177
117.5636
72.2496
71.5365
49.4553
46.0937
21.5959
18.7638
210 200 190 180 170 160 150 140 130 120 110 100 90 80 70 60 50 40 30 20 10 0 -10
f1 (ppm)

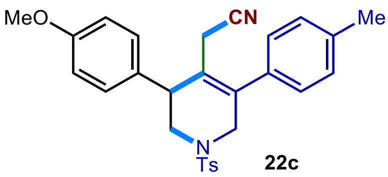


**22c**

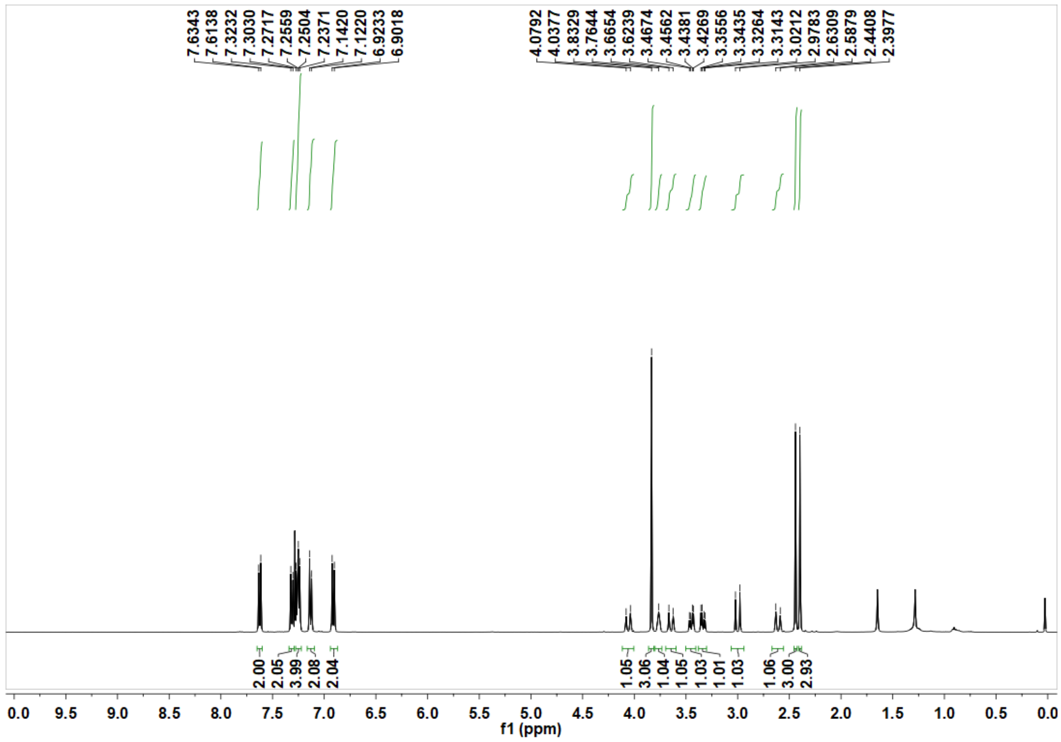

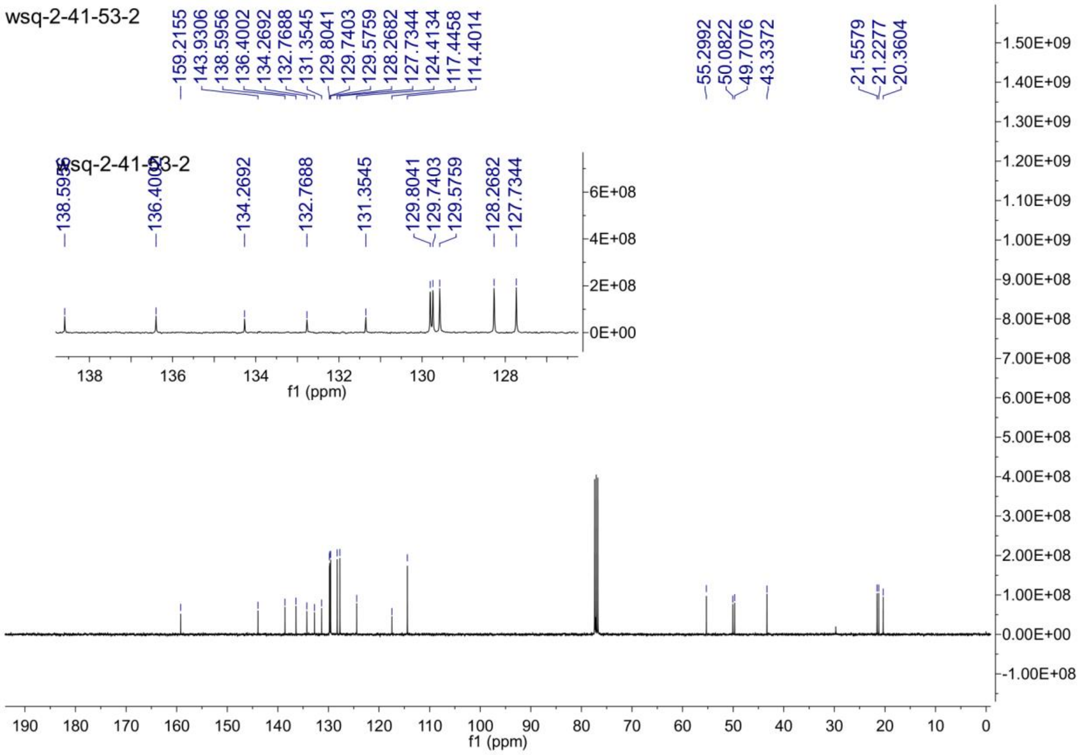

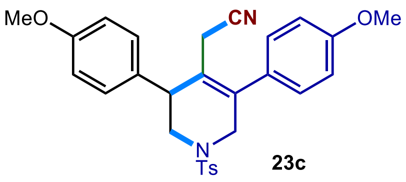
MeO
CN
OMe
N
Ts

**23c**

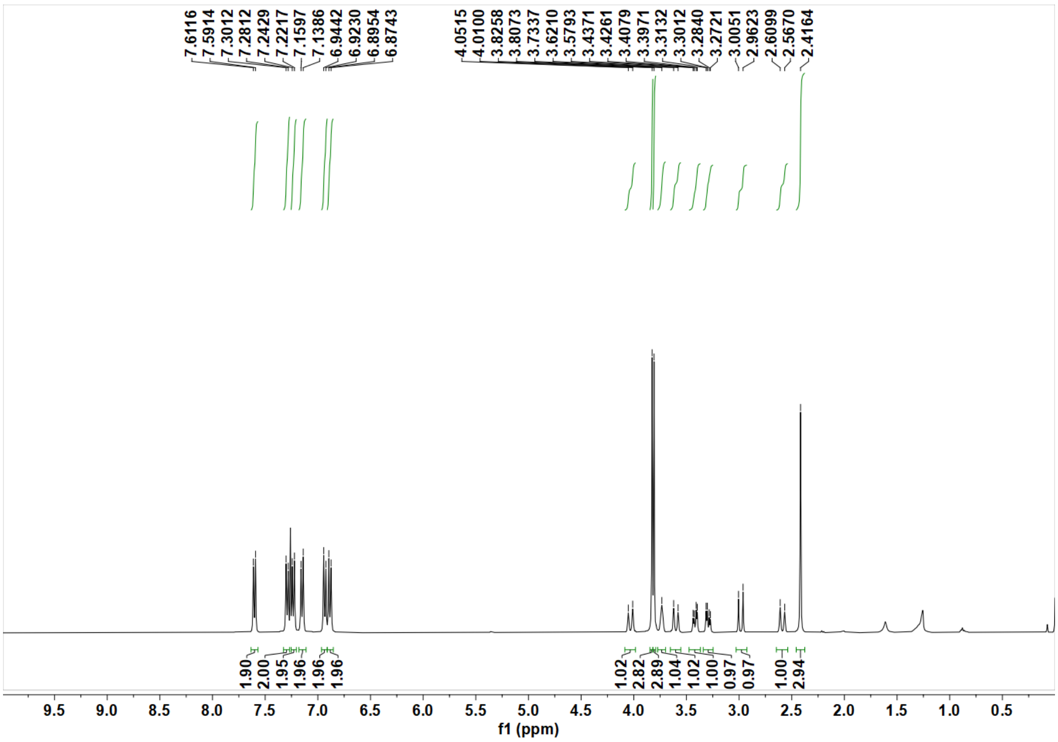
7.6116
7.5914
7.3012
7.2812
7.2429
7.2217
7.1597
7.1386
6.9442
6.9230
6.8954
6.8743
4.0515
4.0100
3.8258
3.8073
3.7337
3.6210
3.5793
3.4371
3.4261
3.4079
3.3971
3.3132
3.3012
3.2840
3.2721
3.0051
2.9623
2.6099
2.5670
2.4164
1.90
2.00
1.95
1.96
1.96
1.96
1.02
2.82
2.89
1.04
1.02
1.00
0.97
0.97
1.00
2.94
9.5 9.0 8.5 8.0 7.5 7.0 6.5 6.0 5.5 5.0 4.5 4.0 3.5 3.0 2.5 2.0 1.5 1.0 0.5
f1 (ppm)

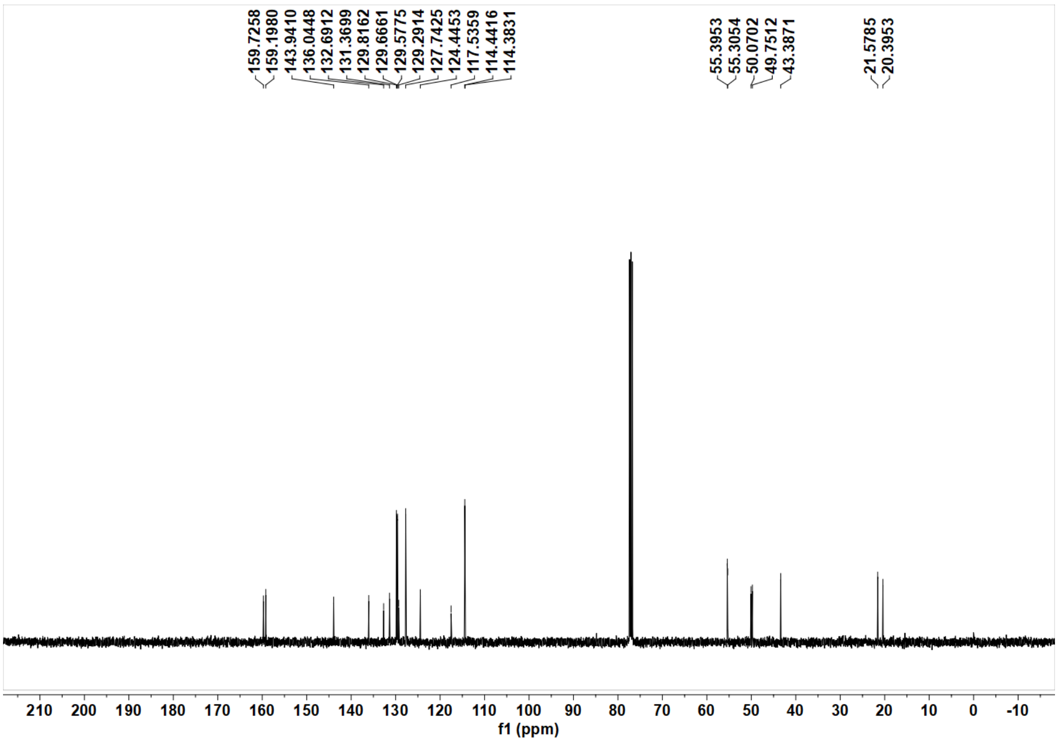
159.7258
159.1980
143.9410
136.0448
132.6912
131.3699
129.8162
129.6661
129.5775
129.2914
127.7425
124.4453
117.5359
114.4416
114.3831
55.3953
55.3054
50.0702
49.7512
43.3871
21.5785
20.3953
210 200 190 180 170 160 150 140 130 120 110 100 90 80 70 60 50 40 30 20 10 0 -10
f1 (ppm)

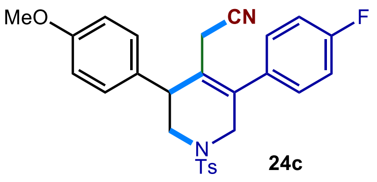
MeO
CN
F
N
Ts

**24c**

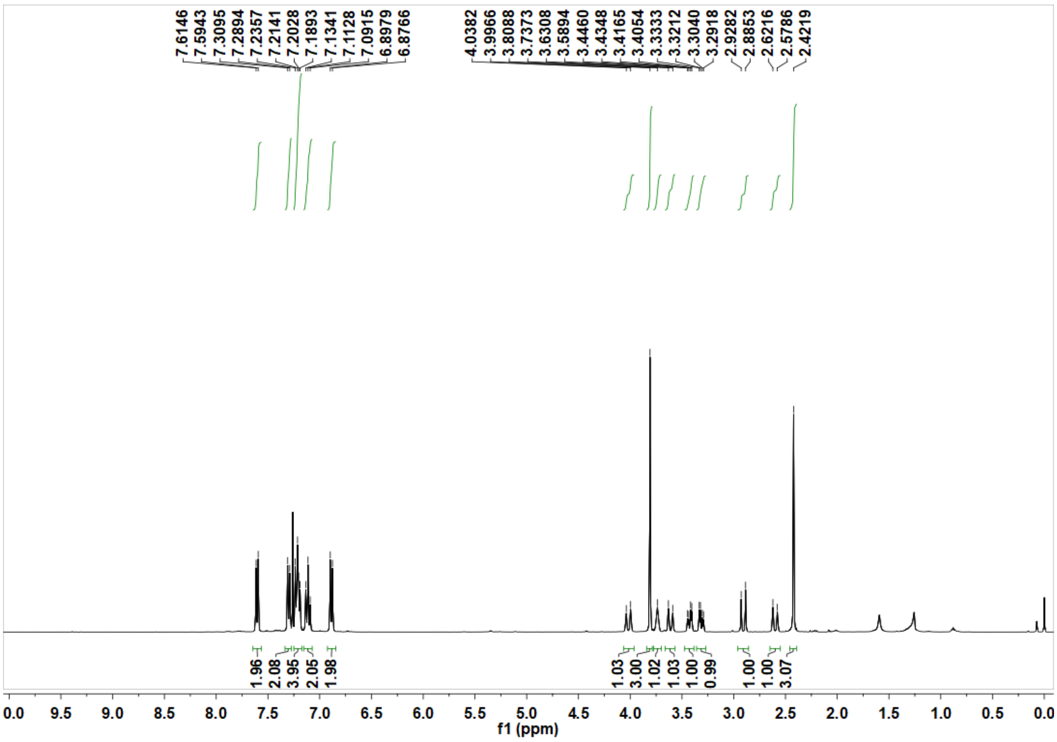
7.6146
7.5943
7.3095
7.2894
7.2357
7.2141
7.2028
7.1893
7.1341
7.1128
7.0915
6.8979
6.8766
4.0382
3.9966
3.8088
3.7373
3.6308
3.5894
3.4460
3.4348
3.4165
3.4054
3.3333
3.3212
3.3040
3.2918
2.9282
2.8853
2.6216
2.5786
2.4219
1.96
2.08
3.95
2.05
1.98
1.03
3.00
1.02
1.03
1.00
0.99
1.00
1.00
3.07
f1 (ppm)

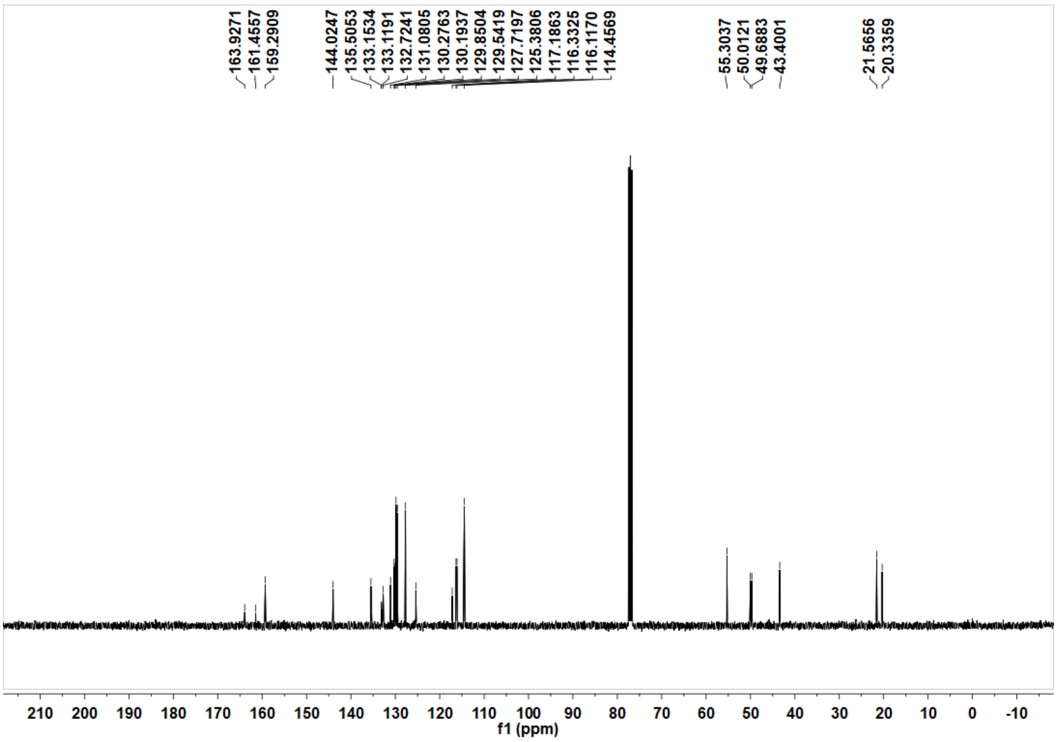
163.9271
161.4557
159.2909
144.0247
135.5053
133.1534
133.1191
132.7241
131.0805
130.2763
130.1937
129.8504
129.5419
127.7197
125.3806
117.1863
116.3325
116.1170
114.4569
55.3037
50.0121
49.6883
43.4001
21.5656
20.3359
f1 (ppm)

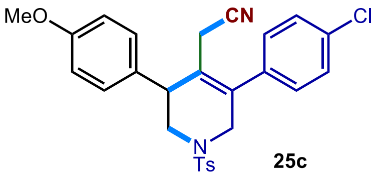
MeO
CN
Cl
N
Ts

**25c**

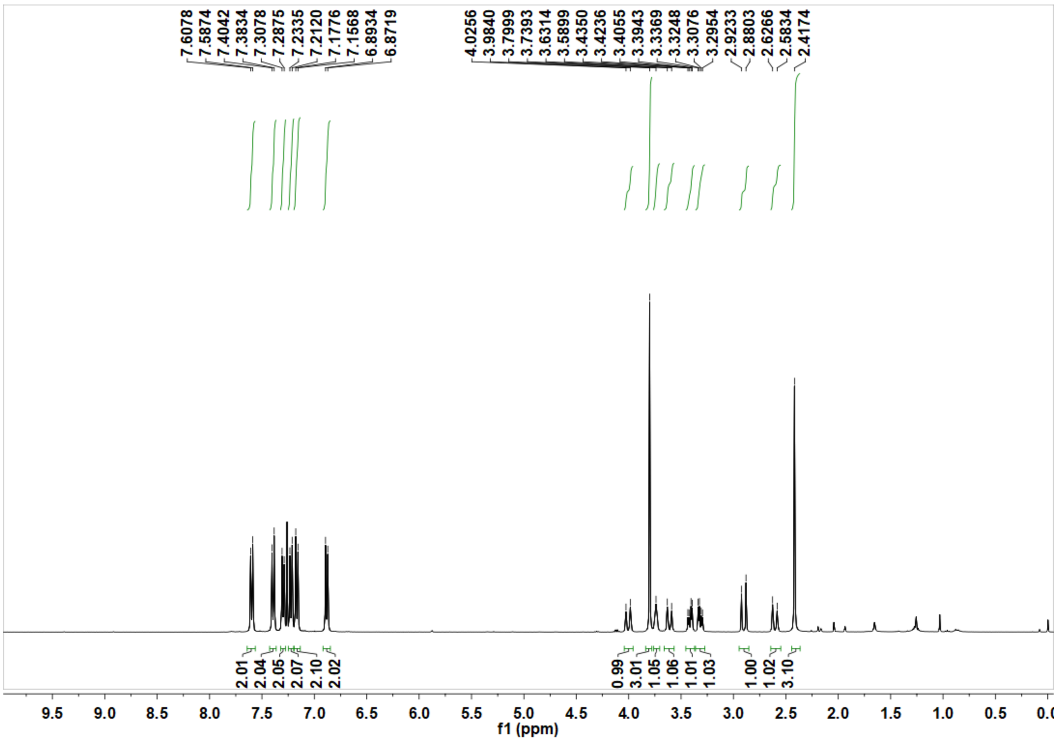
7.6078
7.5874
7.4042
7.3834
7.3078
7.2875
7.2335
7.2120
7.1776
7.1568
6.8934
6.8719
4.0256
3.9840
3.7999
3.7393
3.6314
3.5899
3.4350
3.4236
3.4055
3.3943
3.3369
3.3248
3.3076
3.2954
2.9233
2.8803
2.6266
2.5834
2.4174
2.01
2.04
2.05
2.07
2.10
2.02
0.99
3.01
1.05
1.06
1.01
1.03
1.00
1.02
3.10
9.5 9.0 8.5 8.0 7.5 7.0 6.5 6.0 5.5 5.0 4.5 4.0 3.5 3.0 2.5 2.0 1.5 1.0 0.5
f1 (ppm)

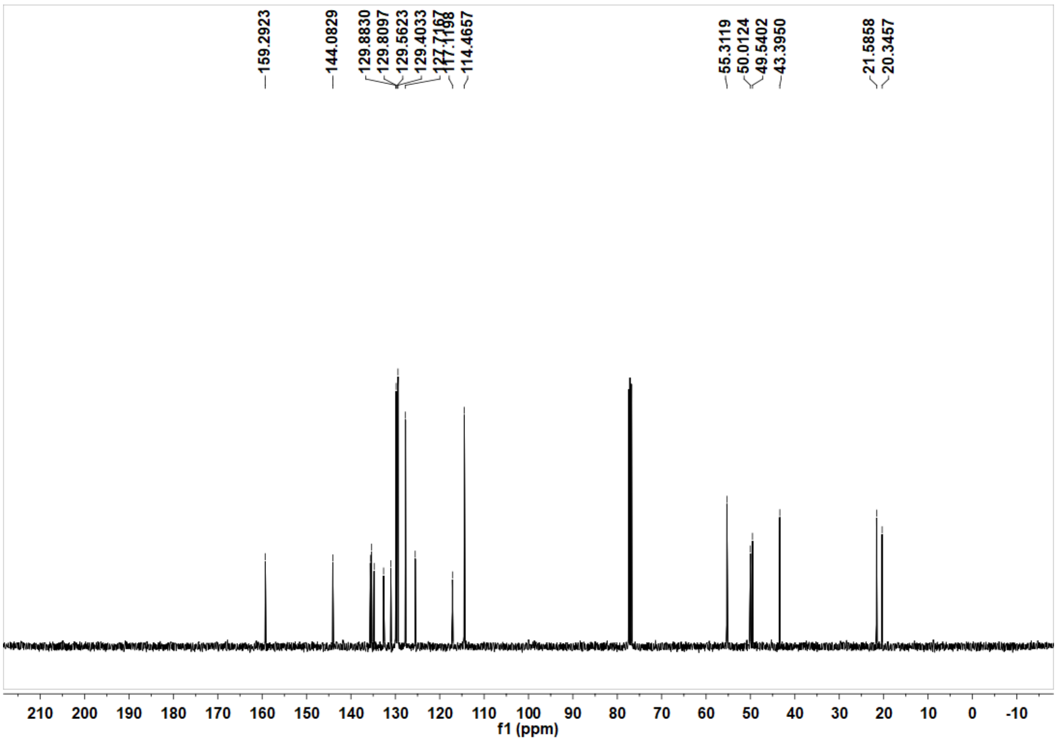
159.2923
144.0829
129.8830
129.8097
129.5623
129.4033
127.7167
117.1198
114.4657
55.3119
50.0124
49.5402
43.3950
21.5858
20.3457
210 200 190 180 170 160 150 140 130 120 110 100 90 80 70 60 50 40 30 20 10 0 -10
f1 (ppm)

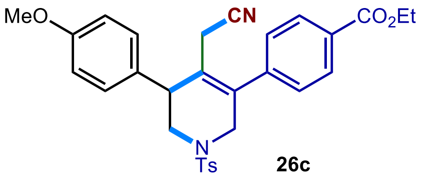
MeO
CN
$CO_2Et$
N
Ts

**26c**

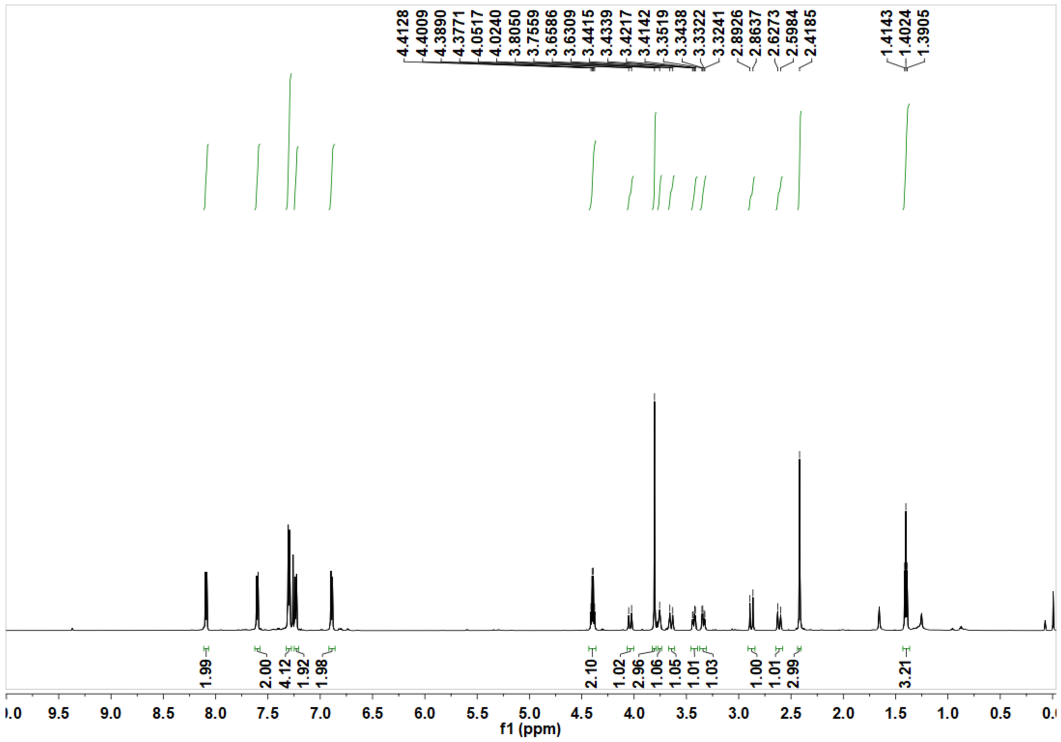
4.4128
4.4009
4.3890
4.3771
4.0517
4.0240
3.8050
3.7559
3.6586
3.6309
3.4415
3.4339
3.4217
3.4142
3.3519
3.3438
3.3322
3.3241
2.8926
2.8637
2.6273
2.5984
2.4185
1.4143
1.4024
1.3905
1.99
2.00
4.12
1.92
1.98
2.10
1.02
2.96
1.06
1.05
1.01
1.03
1.00
1.01
2.99
3.21
f1 (ppm)

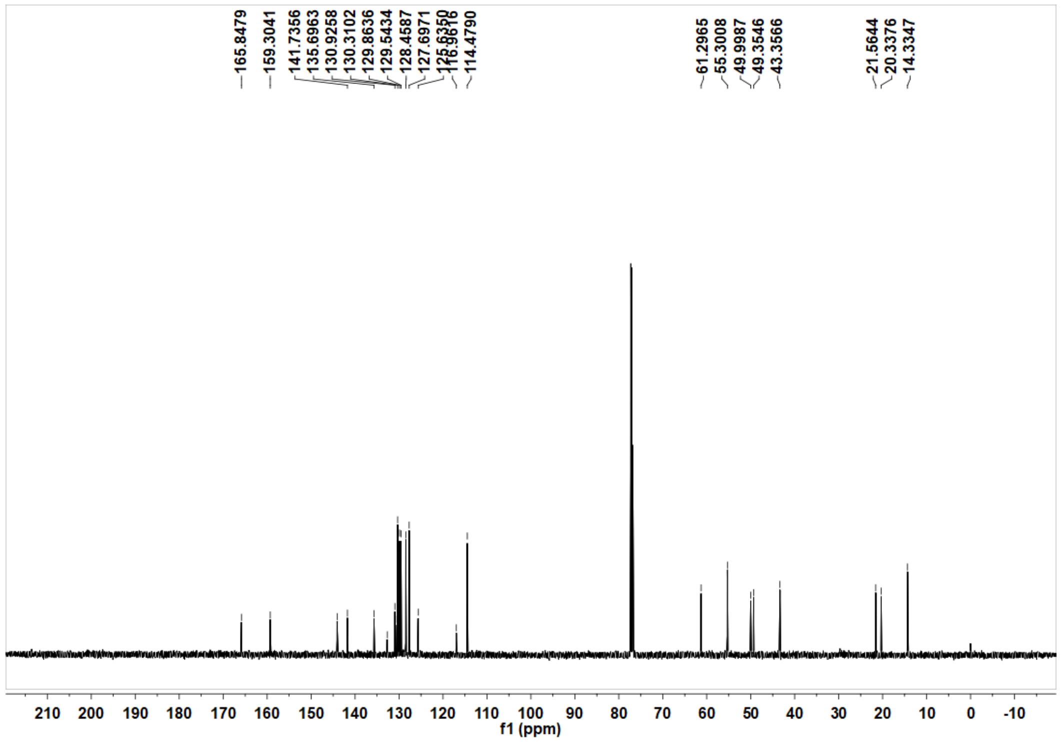
165.8479
159.3041
141.7356
135.6963
130.9258
130.3102
129.8636
129.5434
128.4587
127.6971
125.6350
116.9616
114.4790
61.2965
55.3008
49.9987
49.3546
43.3566
21.5644
20.3376
14.3347
f1 (ppm)

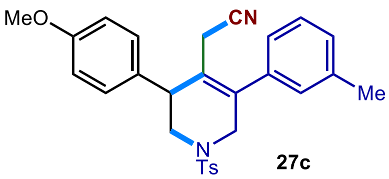
MeO
CN
Me
N
Ts

**27c**

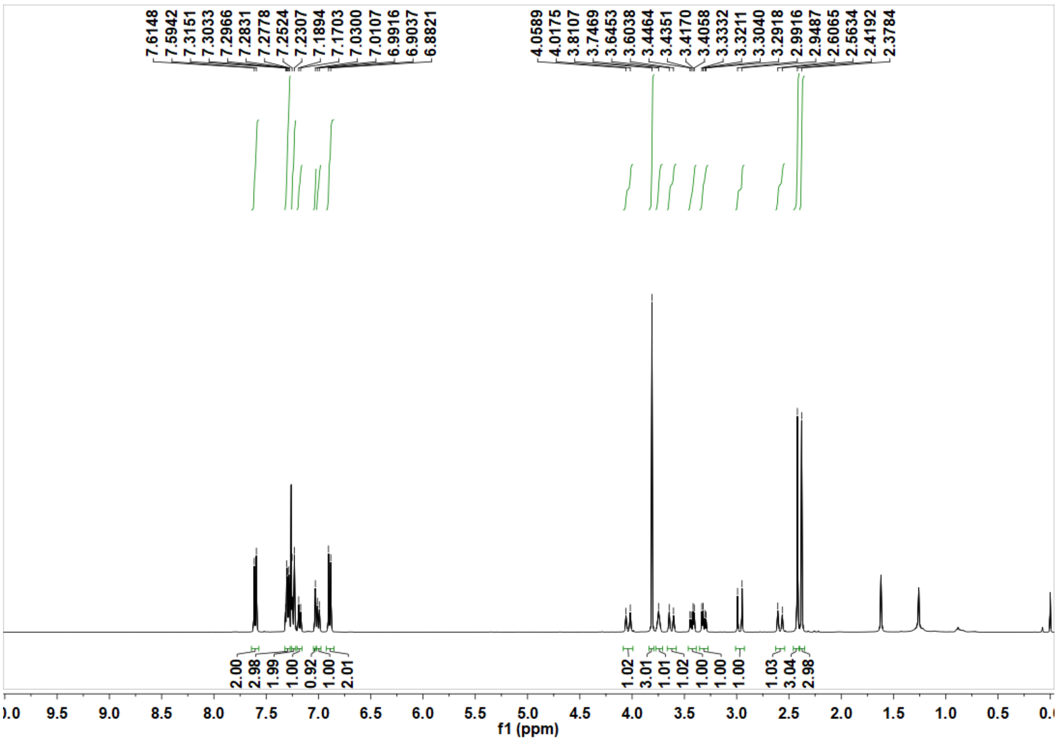
7.6148
7.5942
7.3151
7.3033
7.2966
7.2831
7.2778
7.2524
7.2307
7.1894
7.1703
7.0300
7.0107
6.9916
6.9037
6.8821
4.0589
4.0175
3.8107
3.7469
3.6453
3.6038
3.4464
3.4351
3.4170
3.4058
3.3332
3.3211
3.3040
3.2918
2.9916
2.9487
2.6065
2.5634
2.4192
2.3784
2.00
2.98
1.99
1.00
0.92
1.00
2.01
1.02
3.01
1.01
1.02
1.00
1.00
1.03
3.04
2.98
f1 (ppm)

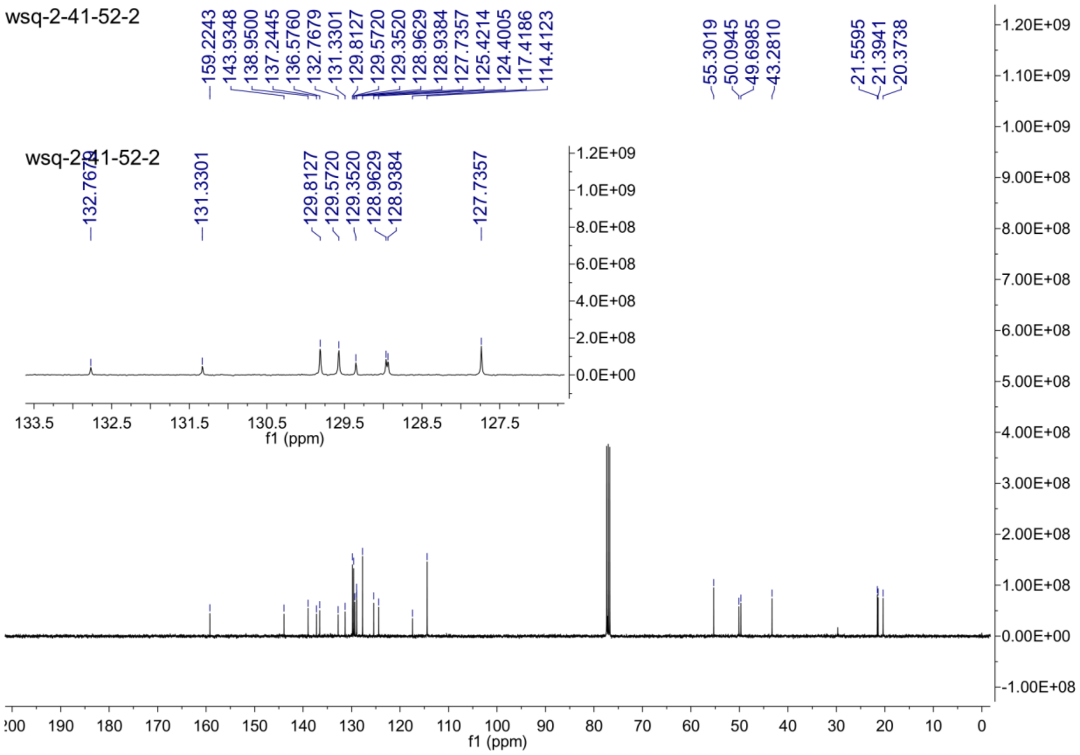
wsq-2-41-52-2
159.2243
143.9348
138.9500
137.2445
136.5760
132.7679
131.3301
129.8127
129.5720
129.3520
128.9629
128.9384
127.7357
125.4214
124.4005
117.4186
114.4123
55.3019
50.0945
49.6985
43.2810
21.5595
21.3941
20.3738
wsq-2-41-52-2
132.7679
131.3301
129.8127
129.5720
129.3520
128.9629
128.9384
127.7357
f1 (ppm)
f1 (ppm)

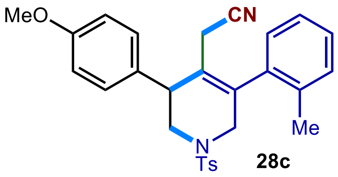

MeO
CN
Me
N
Ts


**28c**

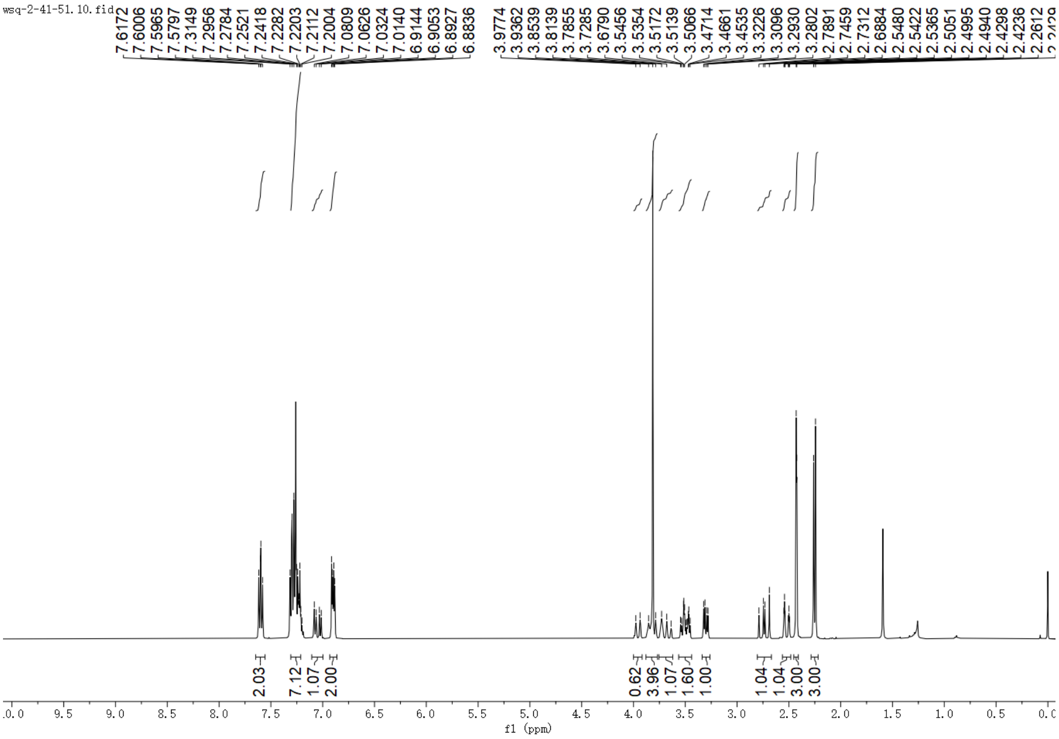

wsq-2-41-51.10.fid
7.6172
7.6006
7.5965
7.5797
7.3149
7.2956
7.2784
7.2521
7.2418
7.2282
7.2203
7.2112
7.2004
7.0809
7.0626
7.0324
7.0140
6.9144
6.9053
6.8927
6.8836
3.9774
3.9362
3.8539
3.8139
3.7855
3.7285
3.6790
3.5456
3.5354
3.5172
3.5139
3.5066
3.4714
3.4661
3.4535
3.3226
3.3096
3.2930
3.2802
2.7891
2.7459
2.7312
2.6884
2.5480
2.5422
2.5365
2.5051
2.4995
2.4940
2.4298
2.4236
2.2612
2.03
7.12
1.07
2.00
0.62
3.96
1.07
1.60
1.00
1.04
1.04
3.00
3.00
9.5
9.0
8.5
8.0
7.5
7.0
6.5
6.0
5.5
5.0
4.5
4.0
3.5
3.0
2.5
2.0
1.5
1.0
0.5
f1 (ppm)


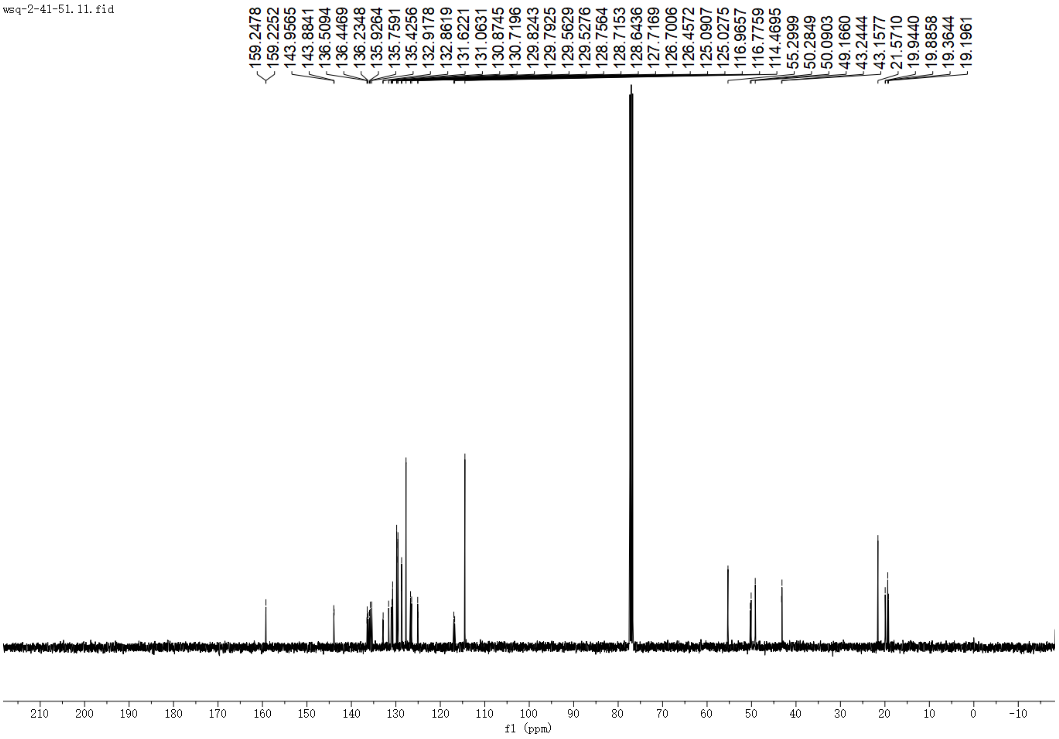

wsq-2-41-51.11.fid
159.2478
159.2252
143.9565
143.8841
136.5094
136.4469
136.2348
135.9264
135.7591
135.4256
132.9178
132.8619
131.6221
131.0631
130.8745
130.7196
129.8243
129.7925
129.5629
129.5276
128.7564
128.7153
128.6436
127.7169
126.7006
126.4572
125.0907
125.0275
116.9657
116.7759
114.4695
55.2999
50.2849
50.0903
49.1660
43.2444
43.1577
21.5710
19.9440
19.8858
19.3644
19.1961
210
200
190
180
170
160
150
140
130
120
110
100
90
80
70
60
50
40
30
20
10
0
-10
f1 (ppm)

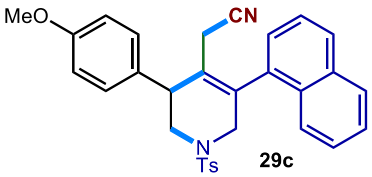
MeO
CN
N
Ts

**29c**

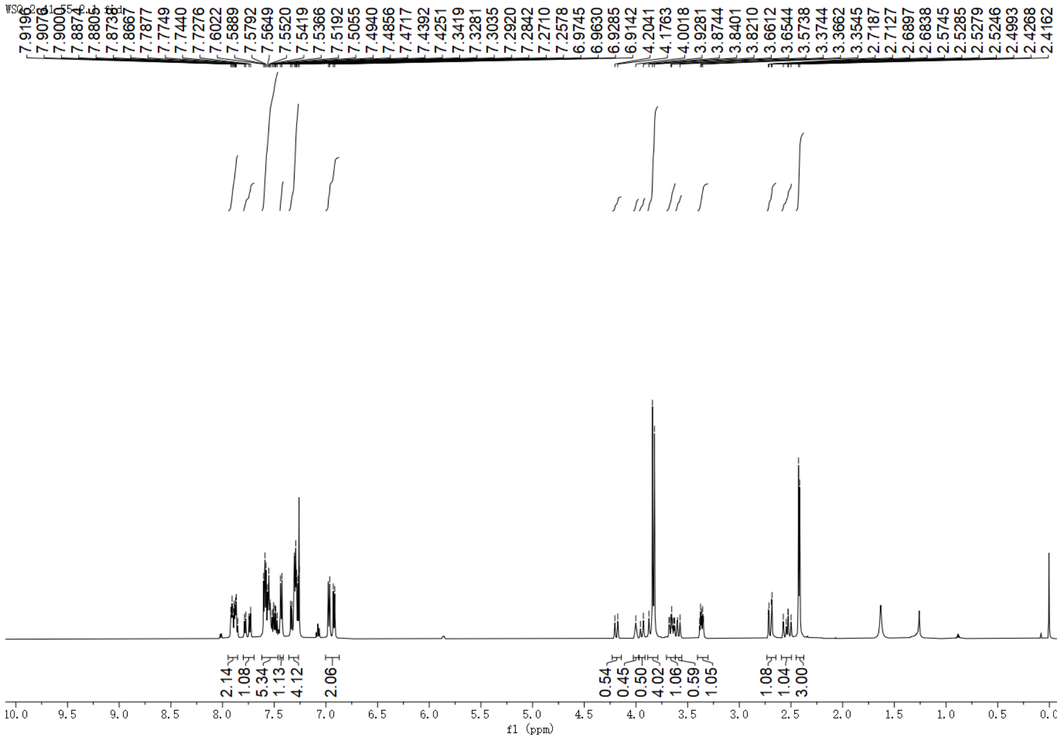
7.9196
7.9074
7.9000
7.8874
7.8805
7.8738
7.8667
7.7877
7.7749
7.7440
7.7276
7.6022
7.5889
7.5792
7.5649
7.5520
7.5419
7.5366
7.5192
7.5055
7.4940
7.4856
7.4717
7.4392
7.4251
7.3419
7.3281
7.3035
7.2920
7.2842
7.2710
7.2578
6.9745
6.9630
6.9285
6.9142
4.2041
4.1763
4.0018
3.9281
3.8744
3.8401
3.8210
3.6612
3.6544
3.5738
3.3744
3.3662
3.3545
2.7187
2.7127
2.6897
2.6838
2.5745
2.5285
2.5279
2.5246
2.4993
2.4268
2.4162
2.14
1.08
5.34
1.13
4.12
2.06
0.54
0.45
0.50
4.02
1.06
0.59
1.05
1.08
1.04
3.00
10.0 9.5 9.0 8.5 8.0 7.5 7.0 6.5 6.0 5.5 5.0 4.5 4.0 3.5 3.0 2.5 2.0 1.5 1.0 0.5 0.0
f1 (ppm)

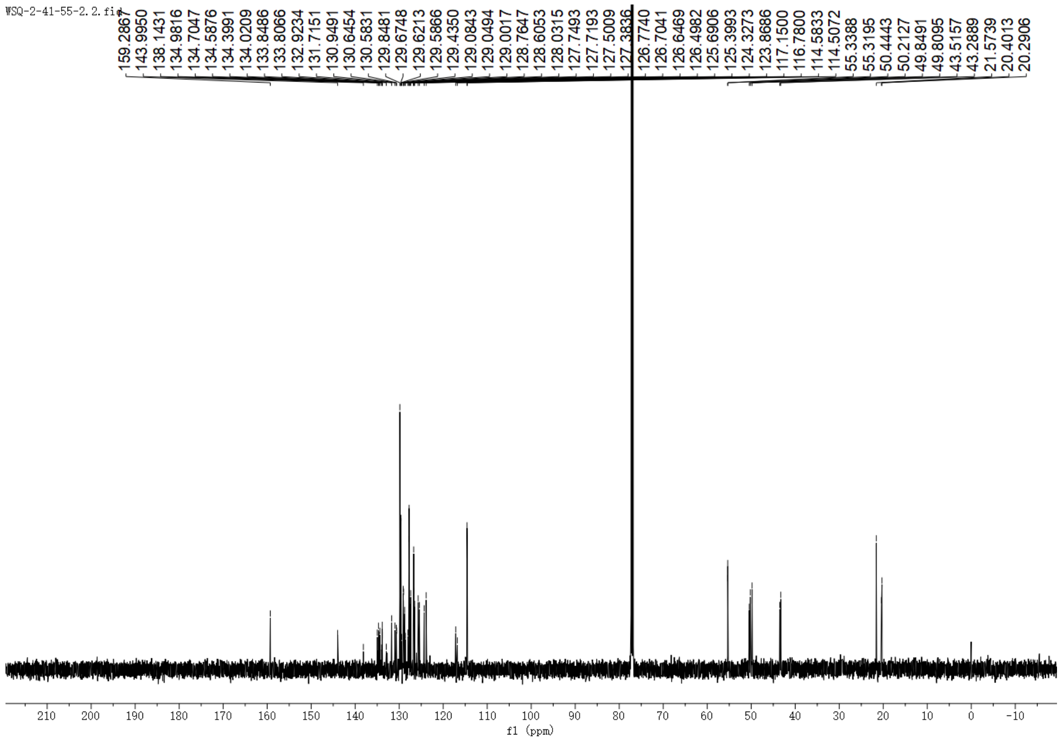
WSQ-2-41-55-2.2.fid
159.2867
143.9950
138.1431
134.9816
134.7047
134.5876
134.3991
134.0209
133.8486
133.8066
132.9234
131.7151
130.9491
130.6454
130.5831
129.8481
129.6748
129.6213
129.5866
129.4350
129.0843
129.0494
129.0017
128.7647
128.6053
128.0315
127.7493
127.7193
127.5009
127.3836
126.7740
126.7041
126.6469
126.4982
125.6906
125.3993
124.3273
123.8686
117.1500
116.7800
114.5833
114.5072
55.3388
55.3195
50.4443
50.2127
49.8491
49.8095
43.5157
43.2889
21.5739
20.4013
20.2906
210 200 190 180 170 160 150 140 130 120 110 100 90 80 70 60 50 40 30 20 10 0 -10
f1 (ppm)

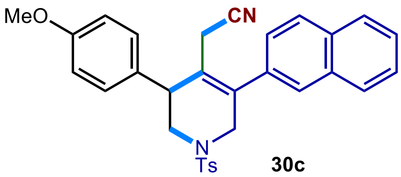
MeO
CN
N
Ts

30c

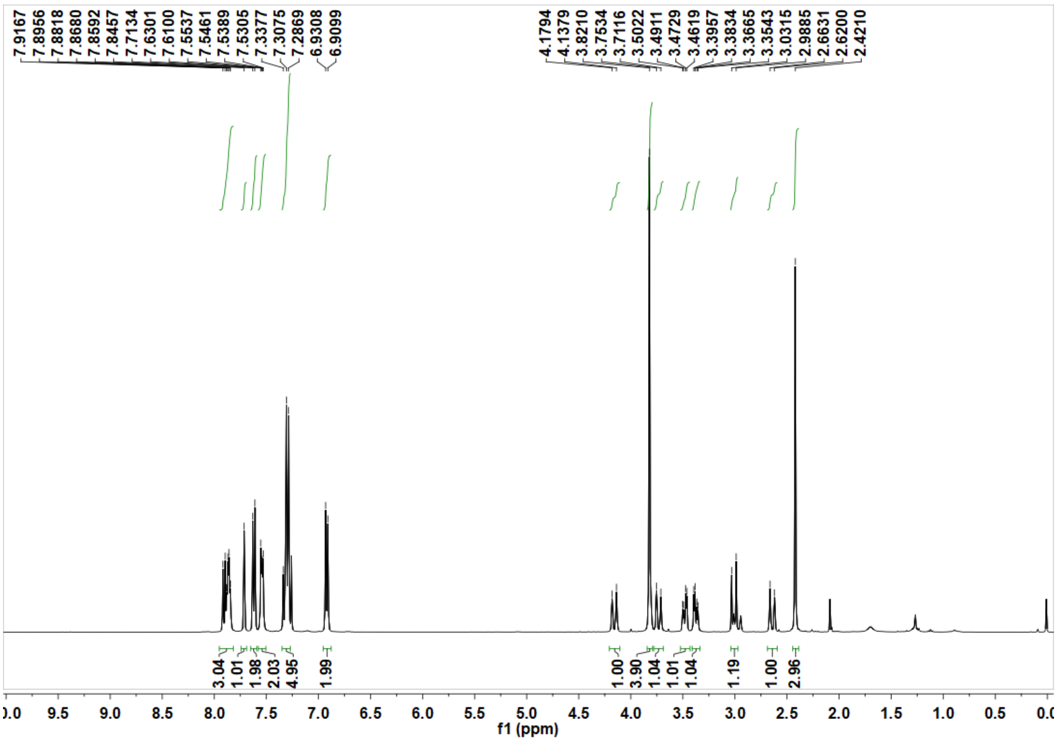
7.9167
7.8956
7.8818
7.8680
7.8592
7.8457
7.7134
7.6301
7.6100
7.5537
7.5461
7.5389
7.5305
7.3377
7.3075
7.2869
6.9308
6.9099
4.1794
4.1379
3.8210
3.7534
3.7116
3.5022
3.4911
3.4729
3.4619
3.3957
3.3834
3.3665
3.3543
3.0315
2.9885
2.6631
2.6200
2.4210
3.04
1.01
1.98
2.03
4.95
1.99
1.00
3.90
1.04
1.01
1.04
1.19
1.00
2.96
f1 (ppm)

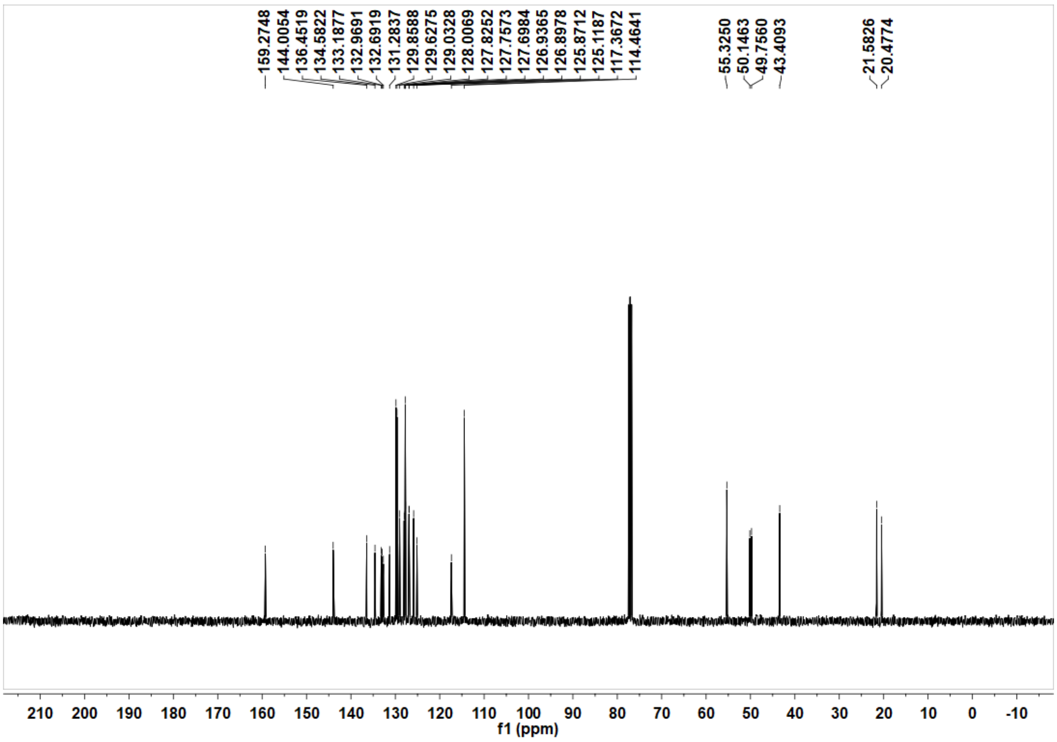
159.2748
144.0054
136.4519
134.5822
133.1877
132.9691
132.6919
131.2837
129.8588
129.6275
129.0328
128.0069
127.8252
127.7573
127.6984
126.9365
126.8978
125.8712
125.1187
117.3672
114.4641
55.3250
50.1463
49.7560
43.4093
21.5826
20.4774
f1 (ppm)

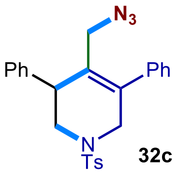
N3
Ph
Ph
N
Ts

**32c**

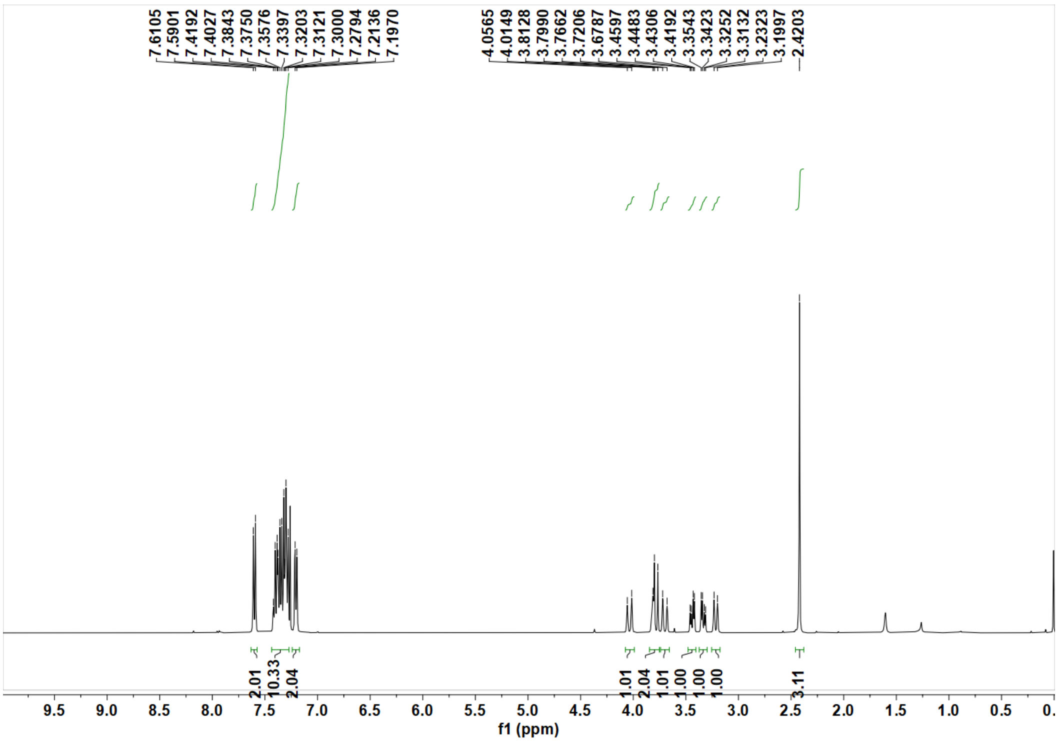
7.6105
7.5901
7.4192
7.4027
7.3843
7.3750
7.3576
7.3397
7.3203
7.3121
7.3000
7.2794
7.2136
7.1970
4.0565
4.0149
3.8128
3.7990
3.7662
3.7206
3.6787
3.4597
3.4483
3.4306
3.4192
3.3543
3.3423
3.3252
3.3132
3.2323
3.1997
2.4203
2.01
10.33
2.04
1.01
2.04
1.01
1.00
1.00
1.00
3.11
9.5 9.0 8.5 8.0 7.5 7.0 6.5 6.0 5.5 5.0 4.5 4.0 3.5 3.0 2.5 2.0 1.5 1.0 0.5 0.
f1 (ppm)

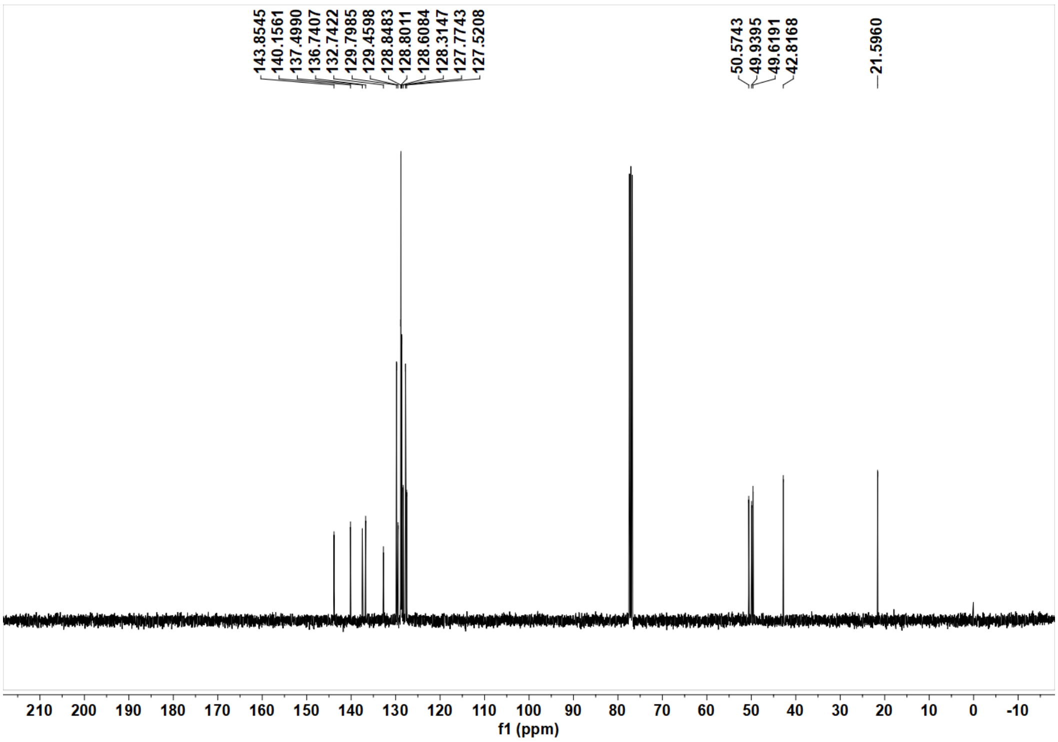
143.8545
140.1561
137.4990
136.7407
132.7422
129.7985
129.4598
128.8483
128.8011
128.6084
128.3147
127.7743
127.5208
50.5743
49.9395
49.6191
42.8168
21.5960
210 200 190 180 170 160 150 140 130 120 110 100 90 80 70 60 50 40 30 20 10 0 -10
f1 (ppm)

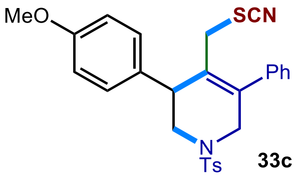
MeO
SCN
Ph
N
Ts

**33c**

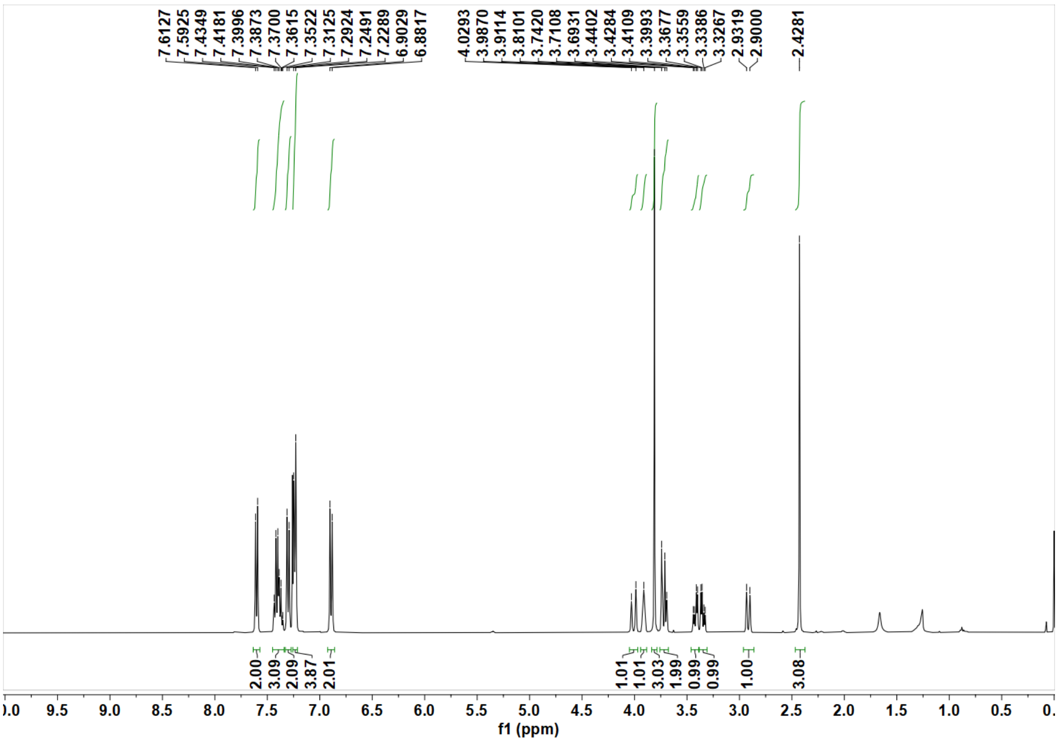
7.6127
7.5925
7.4349
7.4181
7.3996
7.3873
7.3700
7.3615
7.3522
7.3125
7.2924
7.2491
7.2289
6.9029
6.8817
4.0293
3.9870
3.9114
3.8101
3.7420
3.7108
3.6931
3.4402
3.4284
3.4109
3.3993
3.3677
3.3559
3.3386
3.3267
2.9319
2.9000
2.4281
2.00
3.09
2.09
3.87
2.01
1.01
1.01
3.03
1.99
0.99
0.99
1.00
3.08
9.5
9.0
8.5
8.0
7.5
7.0
6.5
6.0
5.5
5.0
4.5
4.0
3.5
3.0
2.5
2.0
1.5
1.0
0.5
f1 (ppm)

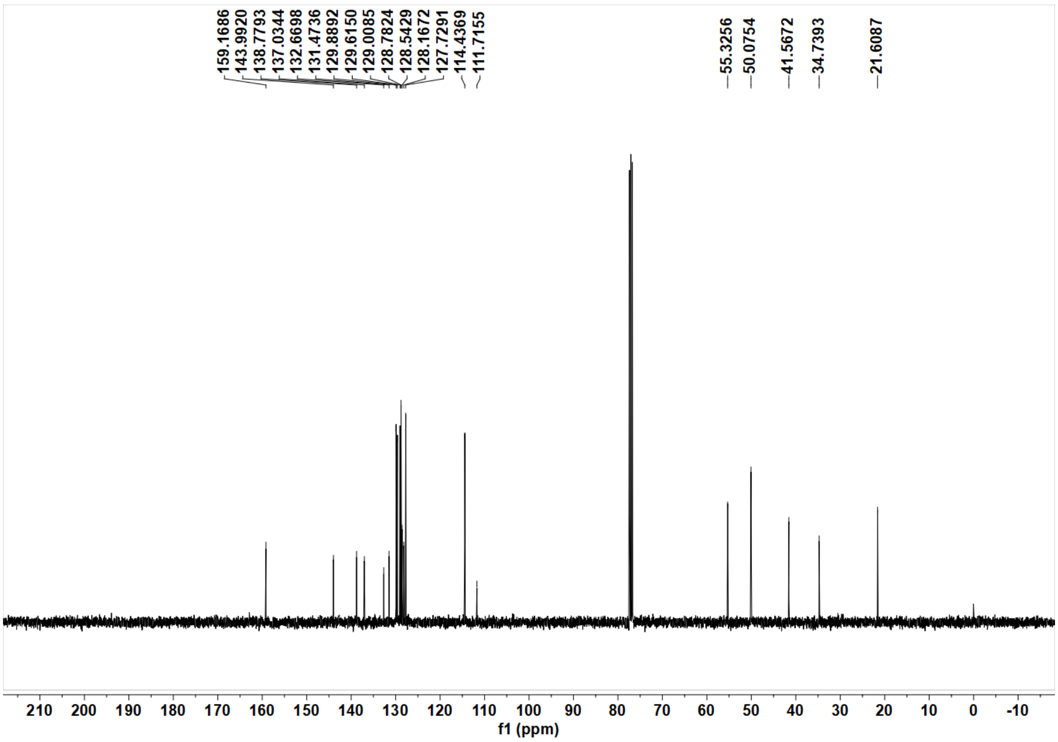
159.1686
143.9920
138.7793
137.0344
132.6698
131.4736
129.8892
129.6150
129.0085
128.7824
128.5429
128.1672
127.7291
114.4369
111.7155
55.3256
50.0754
41.5672
34.7393
21.6087
210
200
190
180
170
160
150
140
130
120
110
100
90
80
70
60
50
40
30
20
10
0
-10
f1 (ppm)

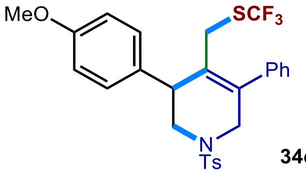

34c

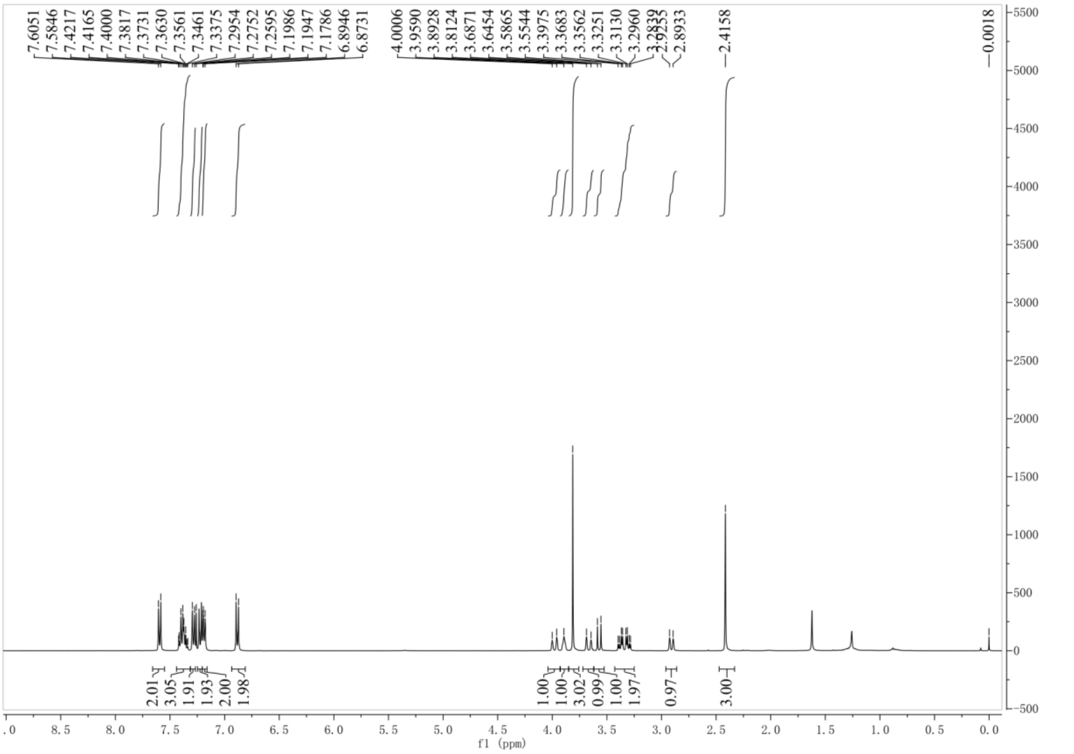
7.6051
7.5846
7.4217
7.4165
7.4000
7.3817
7.3731
7.3630
7.3561
7.3461
7.3375
7.2954
7.2752
7.2595
7.1986
7.1947
7.1786
6.8946
6.8731
4.0006
3.9590
3.8928
3.8124
3.6871
3.6454
3.5865
3.5544
3.3975
3.3683
3.3562
3.3251
3.3130
3.2960
2.9255
2.8933
2.4158
0.0018
f1 (ppm)

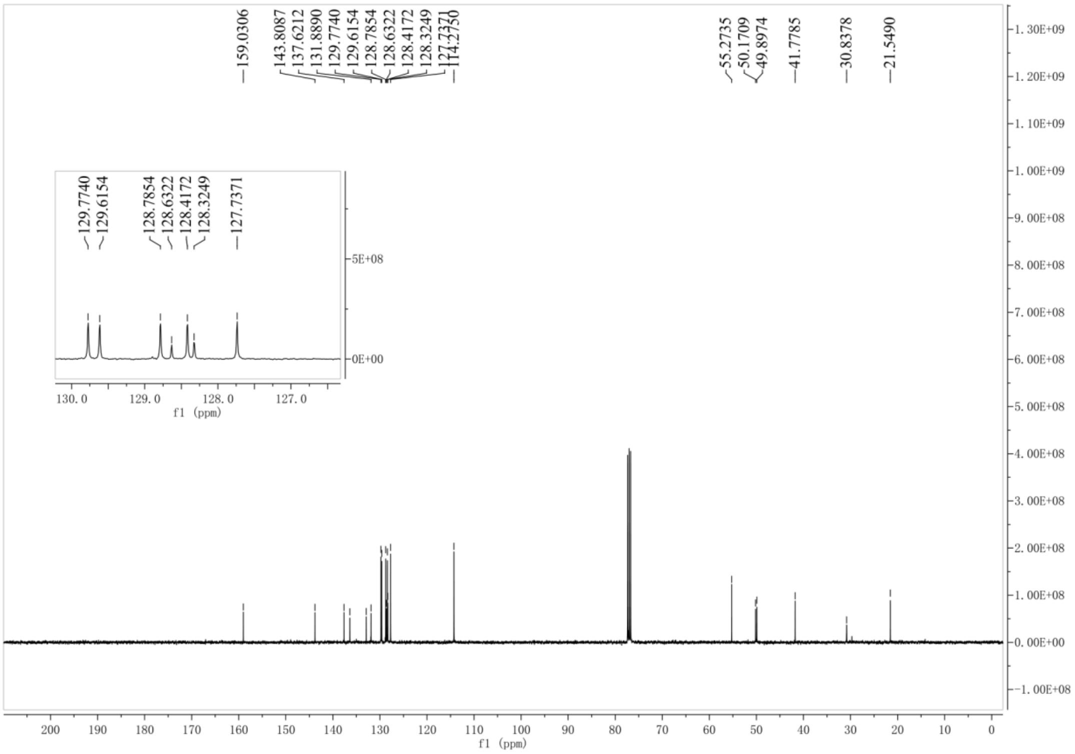
159.0306
143.8087
137.6212
131.8890
129.7740
129.6154
128.7854
128.6322
128.4172
128.3249
114.2750
55.2735
50.1709
49.8974
41.7785
30.8378
21.5490
129.7740
129.6154
128.7854
128.6322
128.4172
128.3249
127.7371
f1 (ppm)

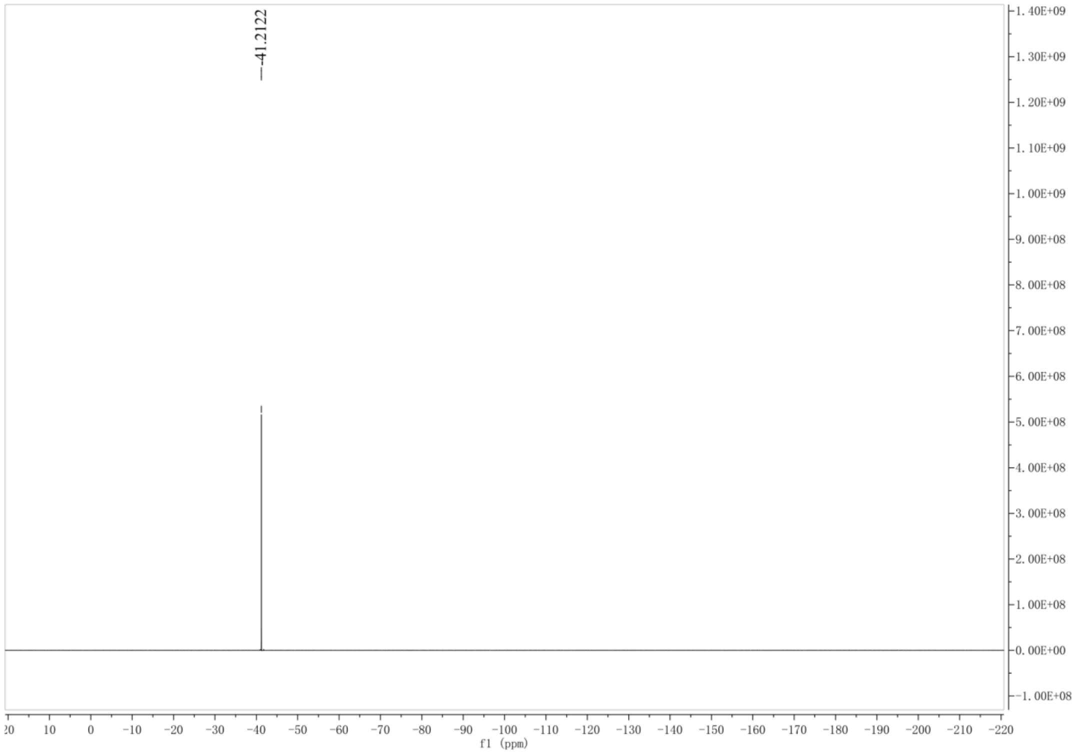

—41.2122
1.40E+09
1.30E+09
1.20E+09
1.10E+09
1.00E+09
9.00E+08
8.00E+08
7.00E+08
6.00E+08
5.00E+08
4.00E+08
3.00E+08
2.00E+08
1.00E+08
0.00E+00
-1.00E+08
20 10 0 -10 -20 -30 -40 -50 -60 -70 -80 -90 -100 -110 -120 -130 -140 -150 -160 -170 -180 -190 -200 -210 -220
f1 (ppm)

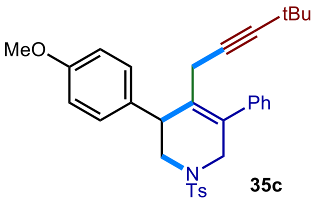


**35c**

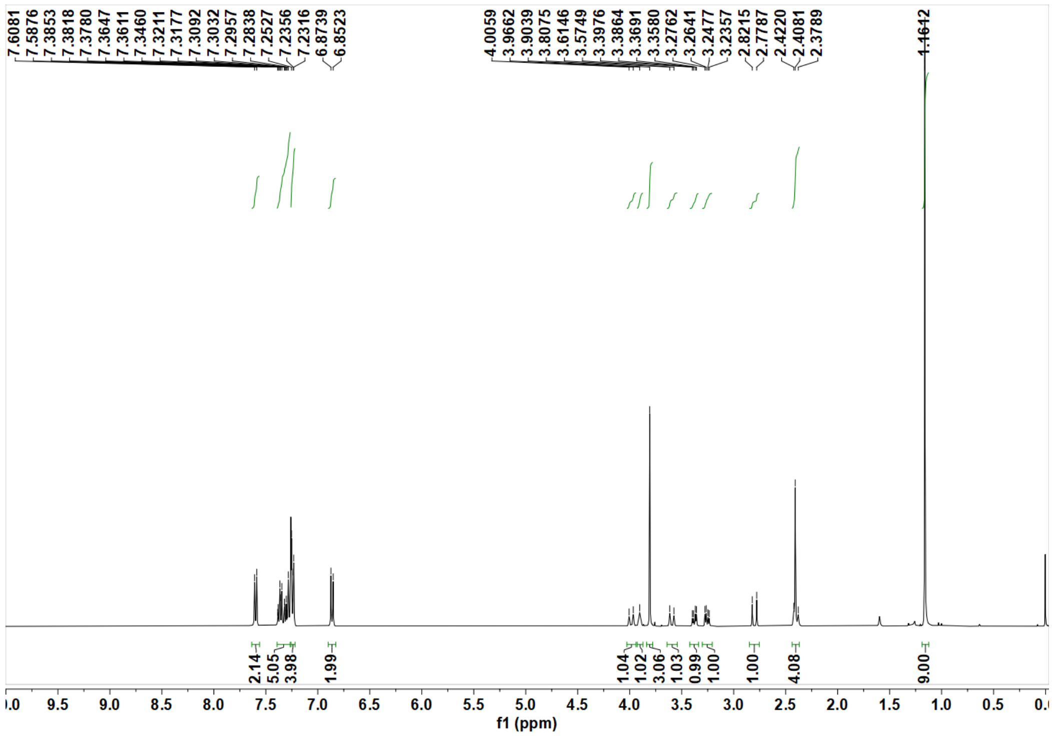

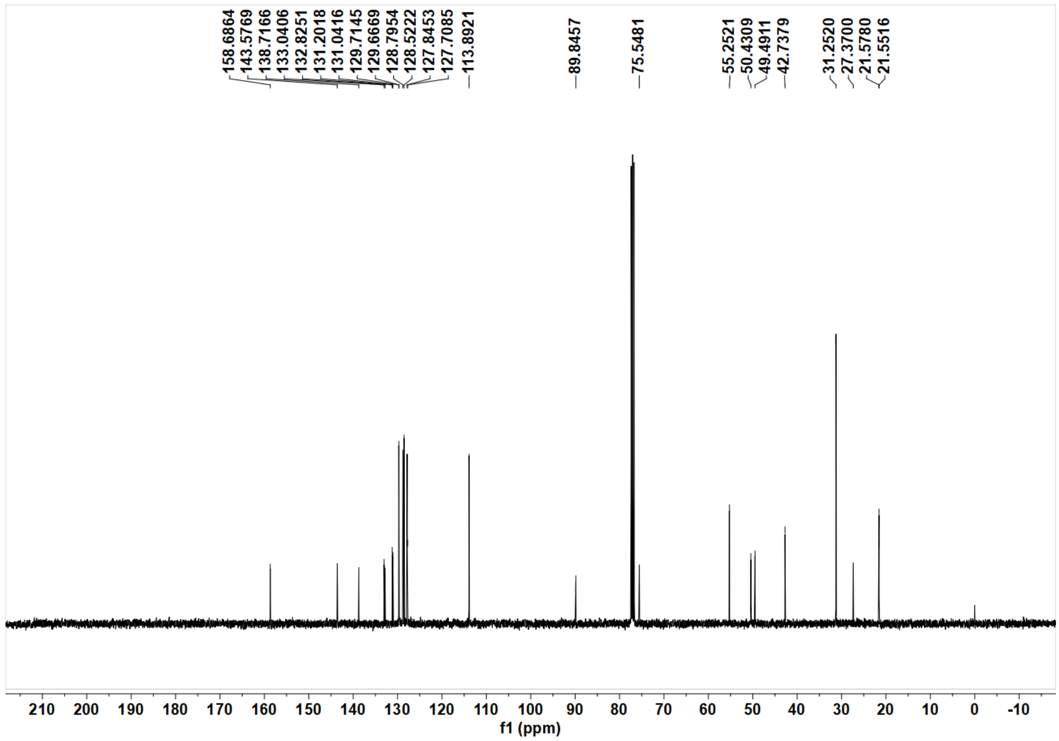

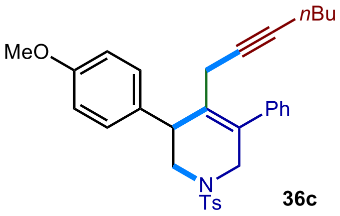
MeO
nBu
Ph
N
Ts

**36c**

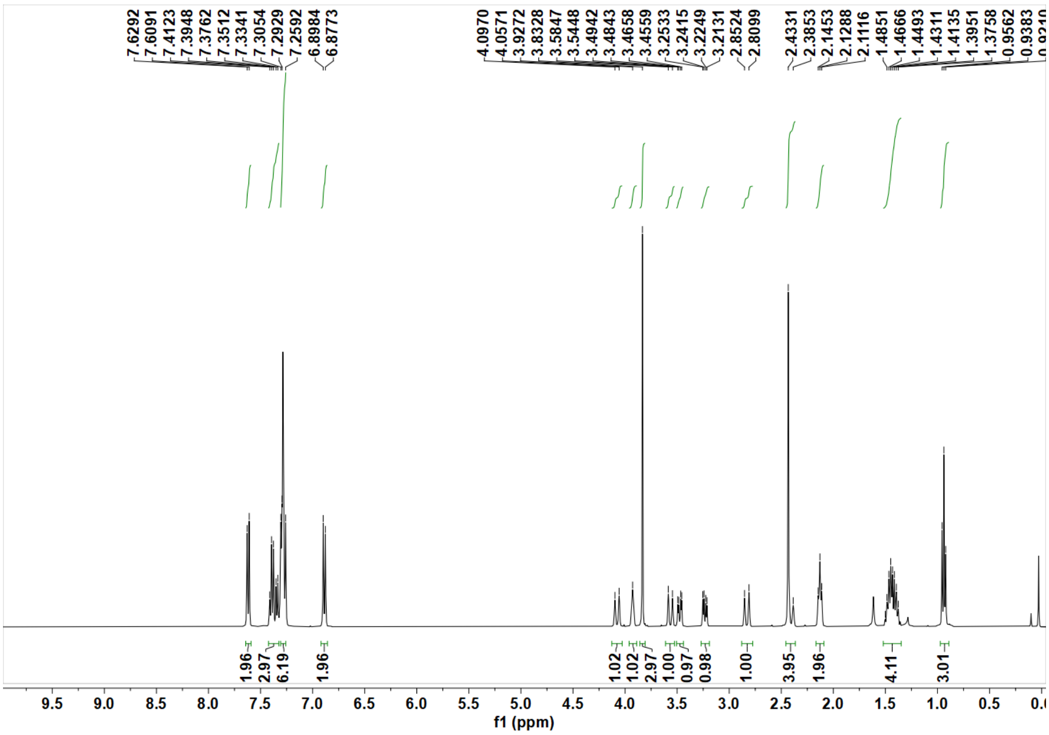
7.6292
7.6091
7.4123
7.3948
7.3762
7.3512
7.3341
7.3054
7.2929
7.2592
6.8984
6.8773
4.0970
4.0571
3.9272
3.8328
3.5847
3.5448
3.4942
3.4843
3.4658
3.4559
3.2533
3.2415
3.2249
3.2131
2.8524
2.8099
2.4331
2.3853
2.1453
2.1288
2.1116
1.4851
1.4666
1.4493
1.4311
1.4135
1.3951
1.3758
0.9562
0.9383
1.96
2.97
6.19
1.96
1.02
1.02
2.97
1.00
0.97
0.98
1.00
3.95
1.96
4.11
3.01
9.5
9.0
8.5
8.0
7.5
7.0
6.5
6.0
5.5
5.0
4.5
4.0
3.5
3.0
2.5
2.0
1.5
1.0
0.5
f1 (ppm)

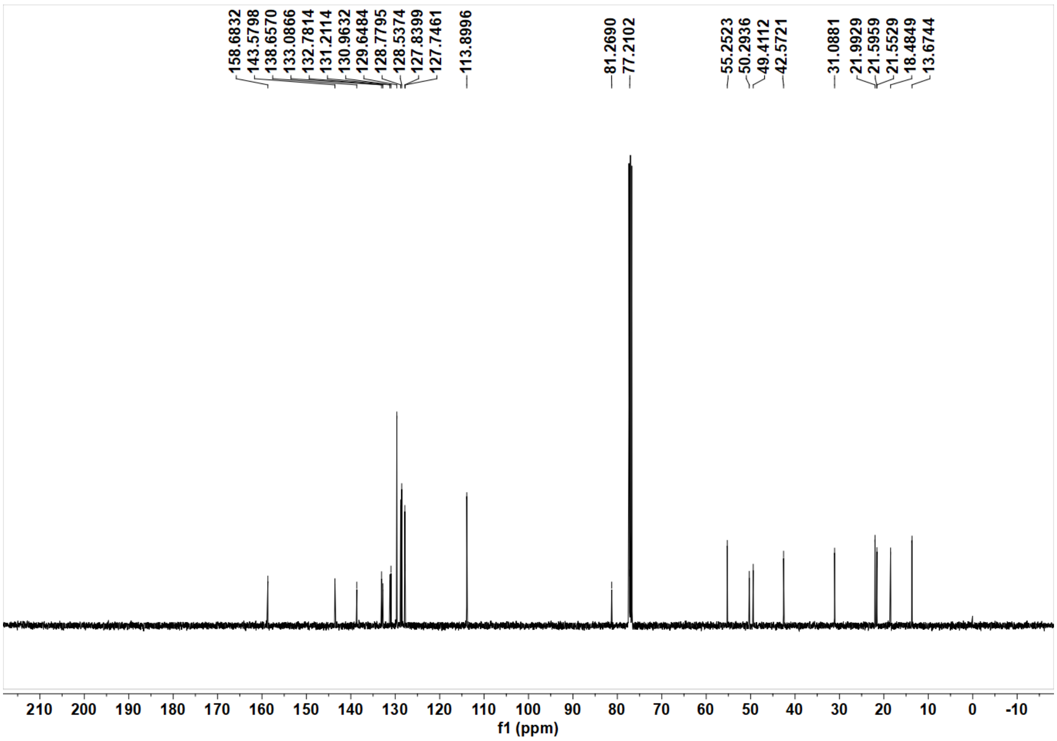
158.6832
143.5798
138.6570
133.0866
132.7814
131.2114
130.9632
129.6484
128.7795
128.5374
127.8399
127.7461
113.8996
81.2690
77.2102
55.2523
50.2936
49.4112
42.5721
31.0881
21.9929
21.5959
21.5529
18.4849
13.6744
210
200
190
180
170
160
150
140
130
120
110
100
90
80
70
60
50
40
30
20
10
0
-10
f1 (ppm)

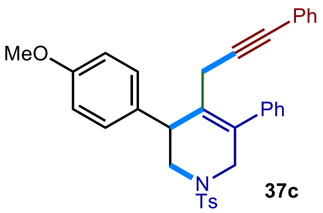
MeO
Ph
Ph
N
Ts

**37c**

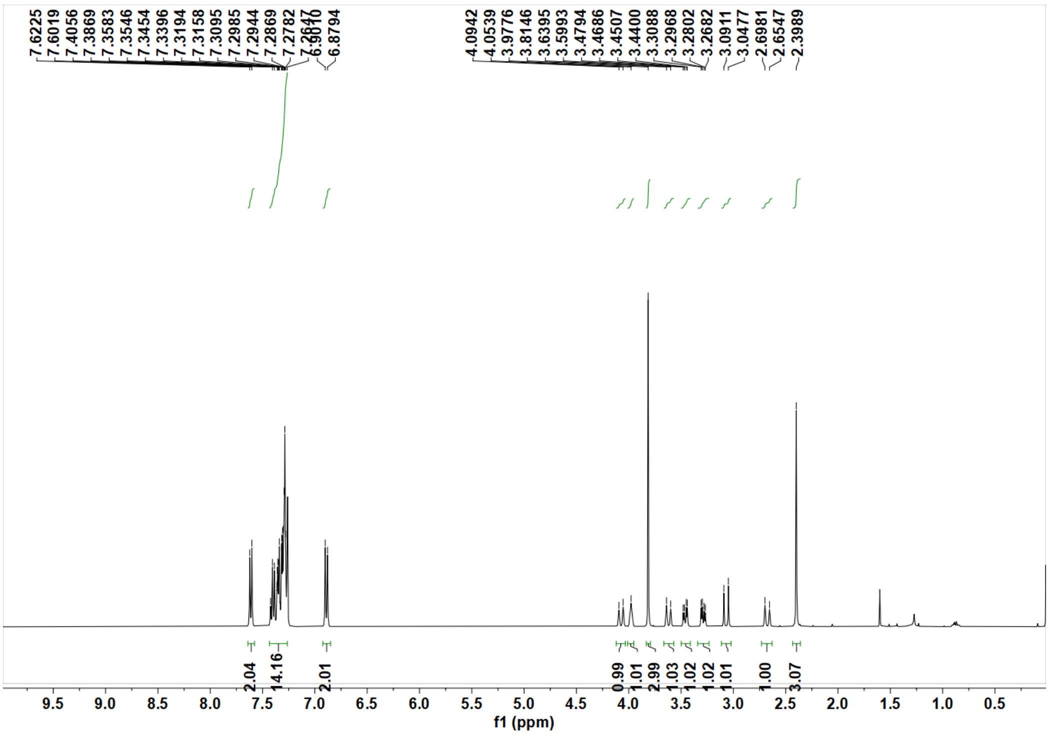
7.6225
7.6019
7.4056
7.3869
7.3583
7.3546
7.3454
7.3396
7.3194
7.3158
7.3095
7.2985
7.2944
7.2869
7.2782
7.2647
6.9010
6.8794
4.0942
4.0539
3.9776
3.8146
3.6395
3.5993
3.4794
3.4686
3.4507
3.4400
3.3088
3.2968
3.2802
3.2682
3.0911
3.0477
2.6981
2.6547
2.3989
2.04
14.16
2.01
0.99
1.01
2.99
1.03
1.02
1.02
1.01
1.00
3.07
9.5
9.0
8.5
8.0
7.5
7.0
6.5
6.0
5.5
5.0
4.5
4.0
3.5
3.0
2.5
2.0
1.5
1.0
0.5
f1 (ppm)

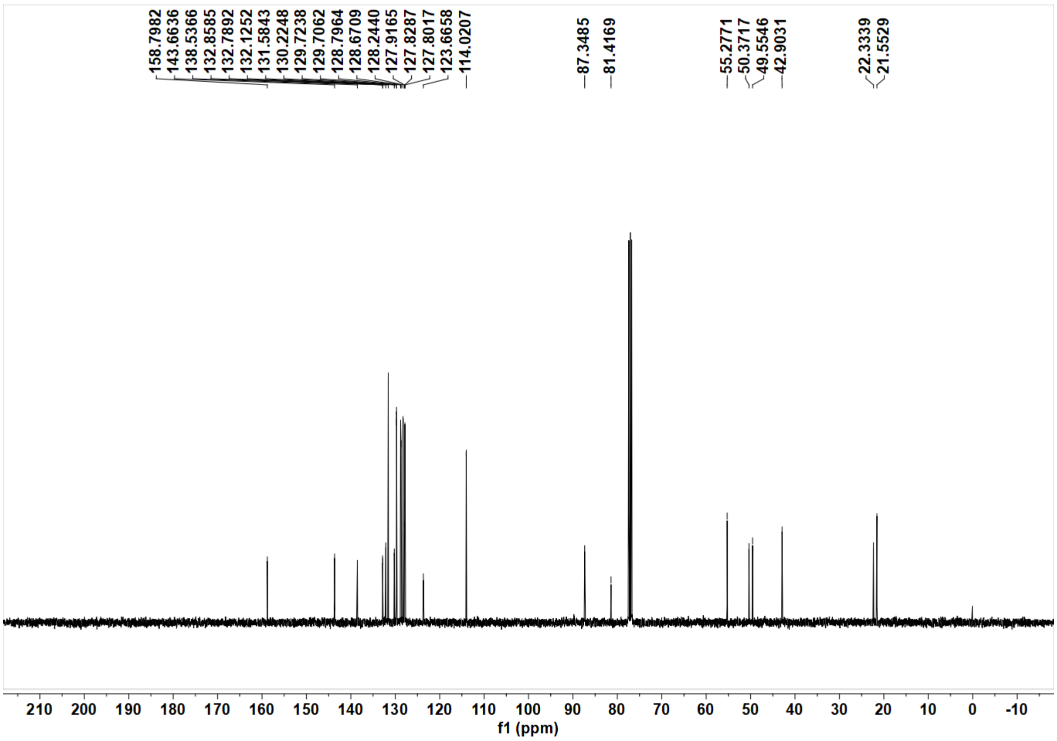
158.7982
143.6636
138.5366
132.8585
132.7892
132.1252
131.5843
130.2248
129.7238
129.7062
128.7964
128.6709
128.2440
127.9165
127.8287
127.8017
123.6658
114.0207
87.3485
81.4169
55.2771
50.3717
49.5546
42.9031
22.3339
21.5529
210
200
190
180
170
160
150
140
130
120
110
100
90
80
70
60
50
40
30
20
10
0
-10
f1 (ppm)

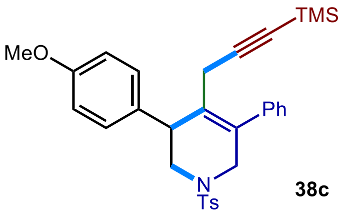
MeO
TMS
Ph
N
Ts

**38c**

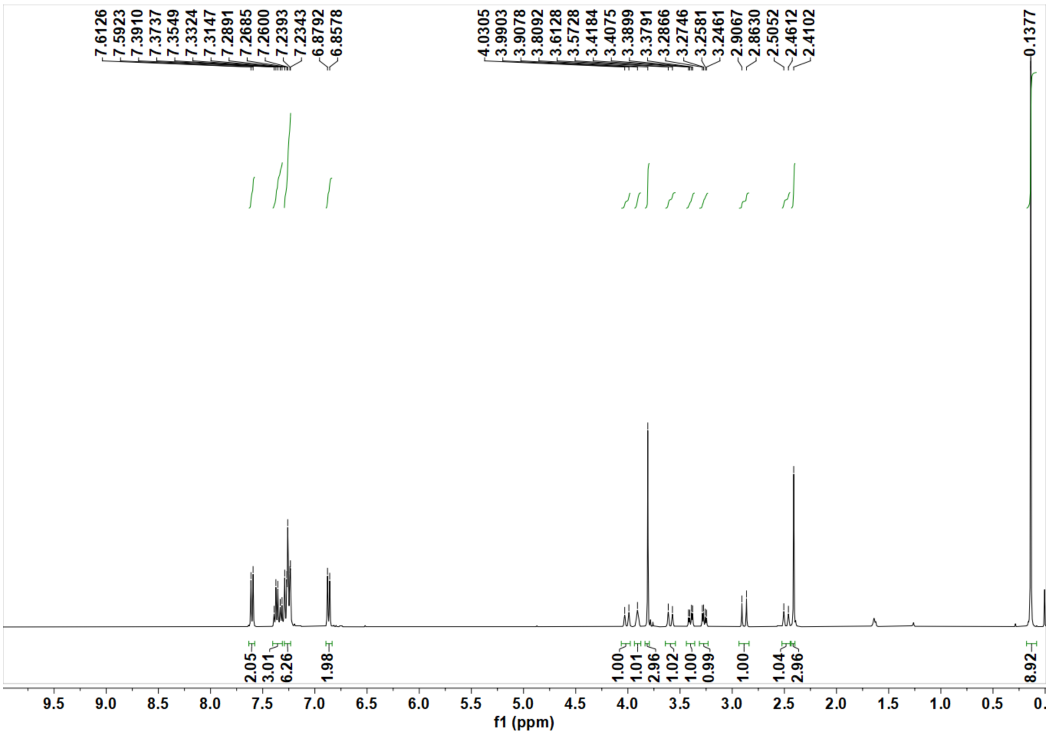
7.6126
7.5923
7.3910
7.3737
7.3549
7.3324
7.3147
7.2891
7.2685
7.2600
7.2393
7.2343
6.8792
6.8578
4.0305
3.9903
3.9078
3.8092
3.6128
3.5728
3.4184
3.4075
3.3899
3.3791
3.2866
3.2746
3.2581
3.2461
2.9067
2.8630
2.5052
2.4612
2.4102
0.1377
2.05
3.01
6.26
1.98
1.00
1.01
2.96
1.02
1.00
0.99
1.00
1.04
2.96
8.92
9.5
9.0
8.5
8.0
7.5
7.0
6.5
6.0
5.5
5.0
4.5
4.0
3.5
3.0
2.5
2.0
1.5
1.0
0.5
0.
f1 (ppm)

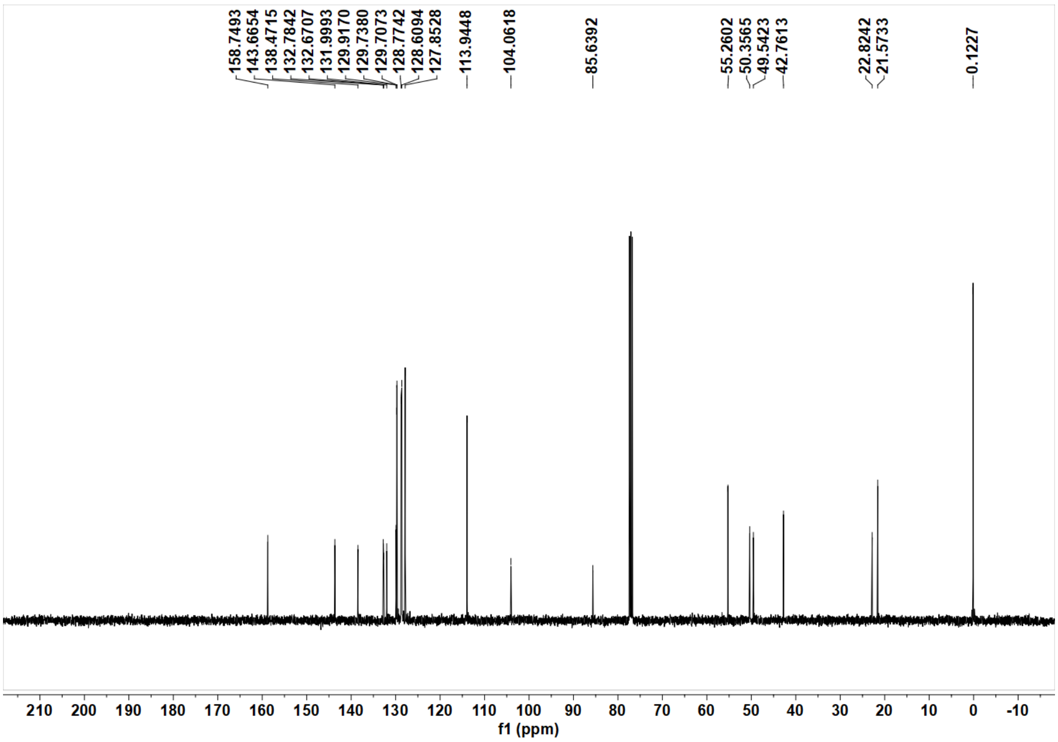
158.7493
143.6654
138.4715
132.7842
132.6707
131.9993
129.9170
129.7380
129.7073
128.7742
128.6094
127.8528
113.9448
104.0618
85.6392
55.2602
50.3565
49.5423
42.7613
22.8242
21.5733
0.1227
210
200
190
180
170
160
150
140
130
120
110
100
90
80
70
60
50
40
30
20
10
0
-10
f1 (ppm)

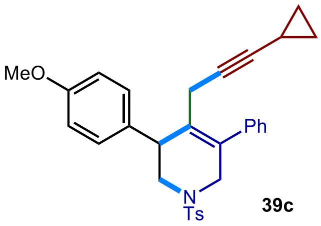

MeO
Ph
N
Ts


**39c**

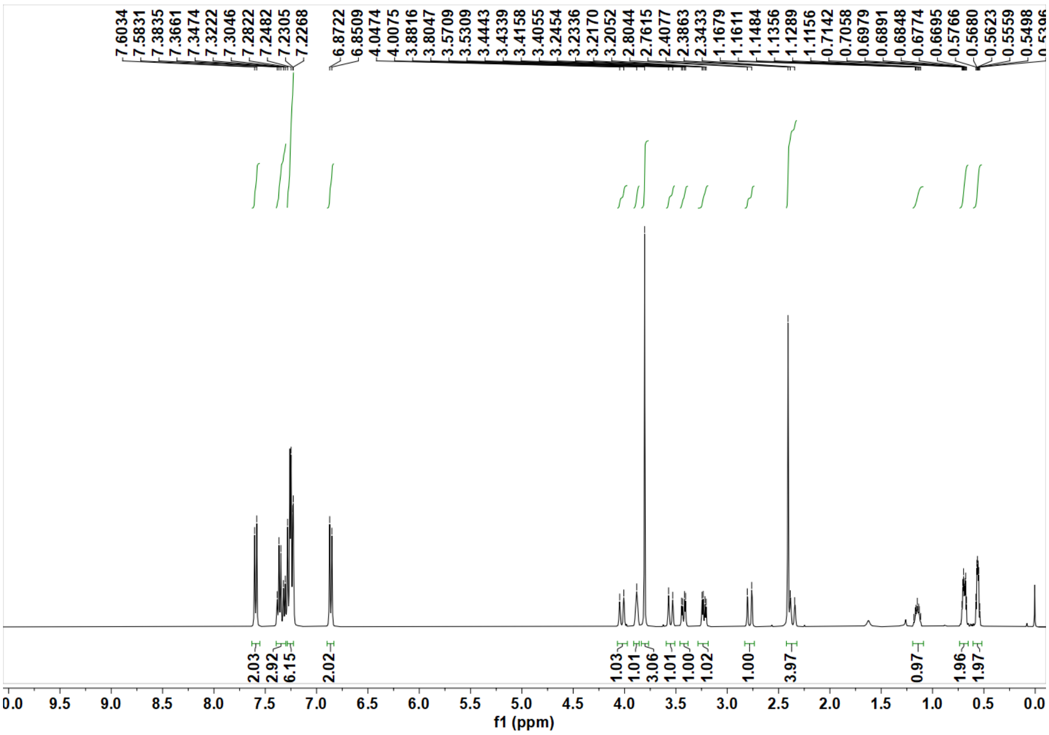

7.6034
7.5831
7.3835
7.3661
7.3474
7.3222
7.3046
7.2822
7.2482
7.2305
7.2268
6.8722
6.8509
4.0474
4.0075
3.8816
3.8047
3.5709
3.5309
3.4443
3.4339
3.4158
3.4055
3.2454
3.2336
3.2170
3.2052
2.8044
2.7615
2.4077
2.3863
2.3433
1.1679
1.1611
1.1484
1.1356
1.1289
1.1156
0.7142
0.7058
0.6979
0.6891
0.6848
0.6774
0.6695
0.5766
0.5680
0.5623
0.5559
0.5498
2.03
2.92
6.15
2.02
1.03
1.01
3.06
1.01
1.00
1.02
1.00
3.97
0.97
1.96
1.97
0.0
9.5
9.0
8.5
8.0
7.5
7.0
6.5
6.0
5.5
5.0
4.5
4.0
3.5
3.0
2.5
2.0
1.5
1.0
0.5
0.0
f1 (ppm)


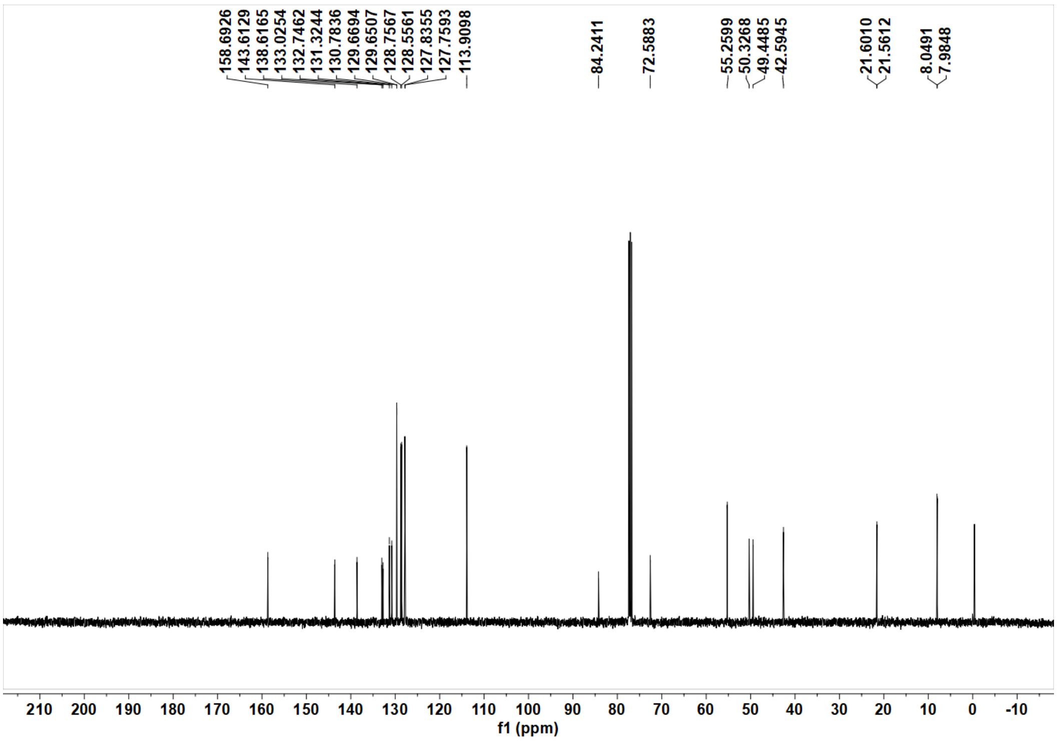

158.6926
143.6129
138.6165
133.0254
132.7462
131.3244
130.7836
129.6694
129.6507
128.7567
128.5561
127.8355
127.7593
113.9098
84.2411
72.5883
55.2599
50.3268
49.4485
42.5945
21.6010
21.5612
8.0491
7.9848
210
200
190
180
170
160
150
140
130
120
110
100
90
80
70
60
50
40
30
20
10
0
-10
f1 (ppm)

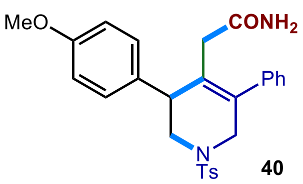

**40**

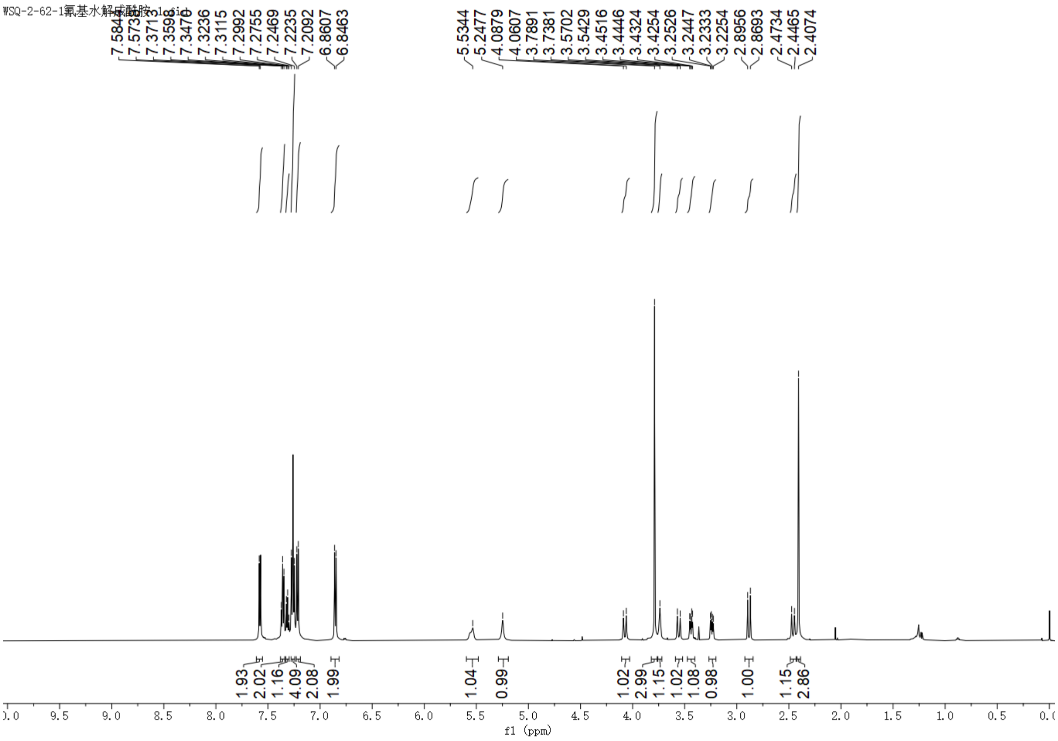

WSQ-2-62-1氰基水解成酰胺.1.fid
7.5847 7.5731 7.3713 7.3598 7.3476 7.3236 7.3115 7.2992 7.2755 7.2469 7.2235 7.2092 6.8607 6.8463
5.5344 5.2477 4.0879 4.0607 3.7891 3.7381 3.5702 3.5429 3.4516 3.4446 3.4324 3.4254 3.2526 3.2447 3.2333 3.2254 2.8956 2.8693 2.4734 2.4465 2.4074
1.93 2.02 1.16 4.09 2.08 1.99 1.04 0.99 1.02 2.99 1.15 1.02 1.08 0.98 1.00 1.15 2.86
f1 (ppm)


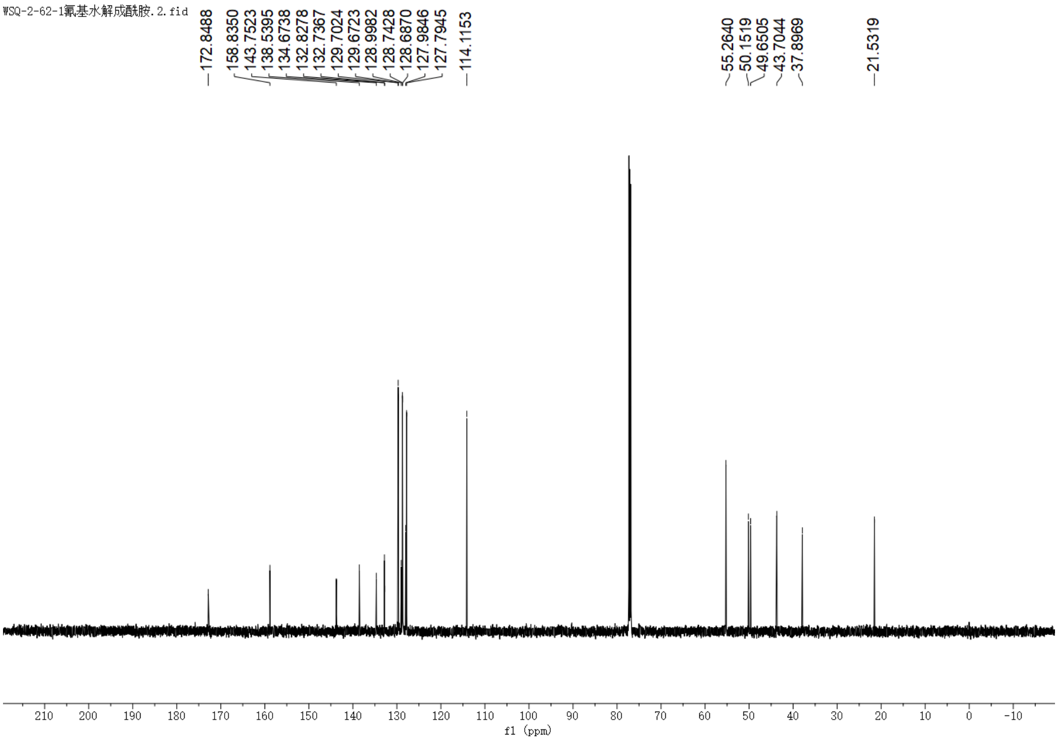

WSQ-2-62-1氰基水解成酰胺.2.fid
172.8488
158.8350 143.7523 138.5395 134.6738 132.8278 132.7367 129.7024 129.6723 128.9982 128.7428 128.6870 127.9846 127.7945
114.1153
55.2640 50.1519 49.6505 43.7044 37.8969
21.5319
f1 (ppm)

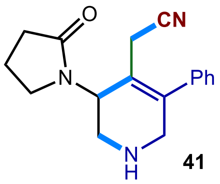


**41**

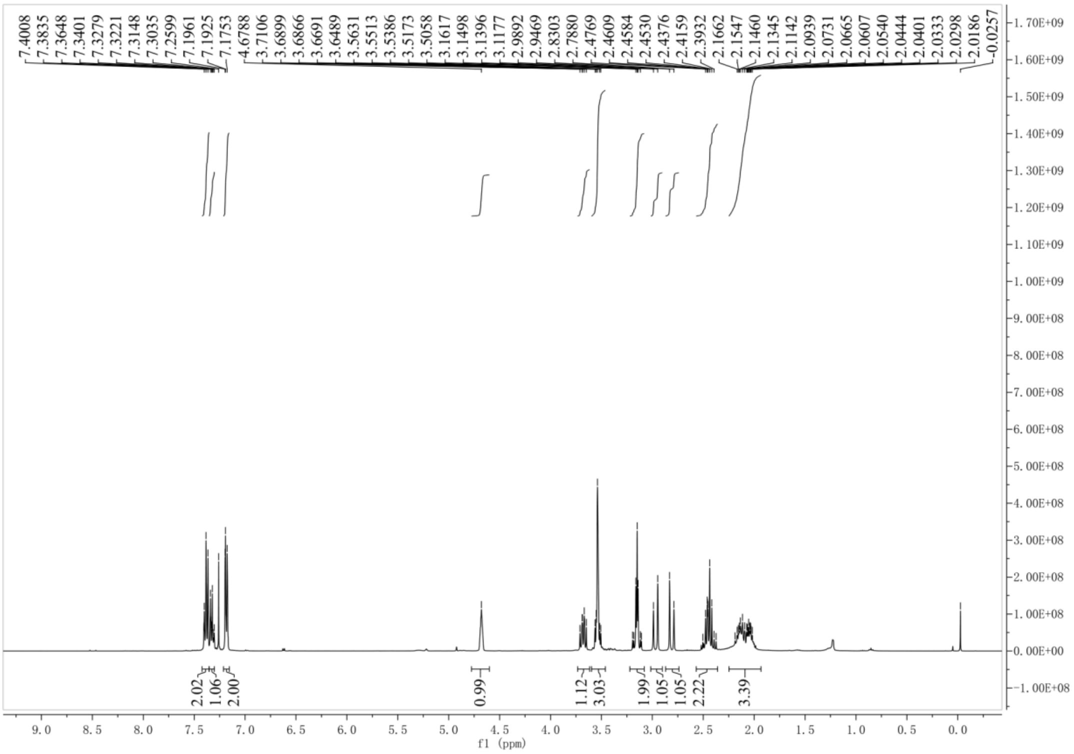

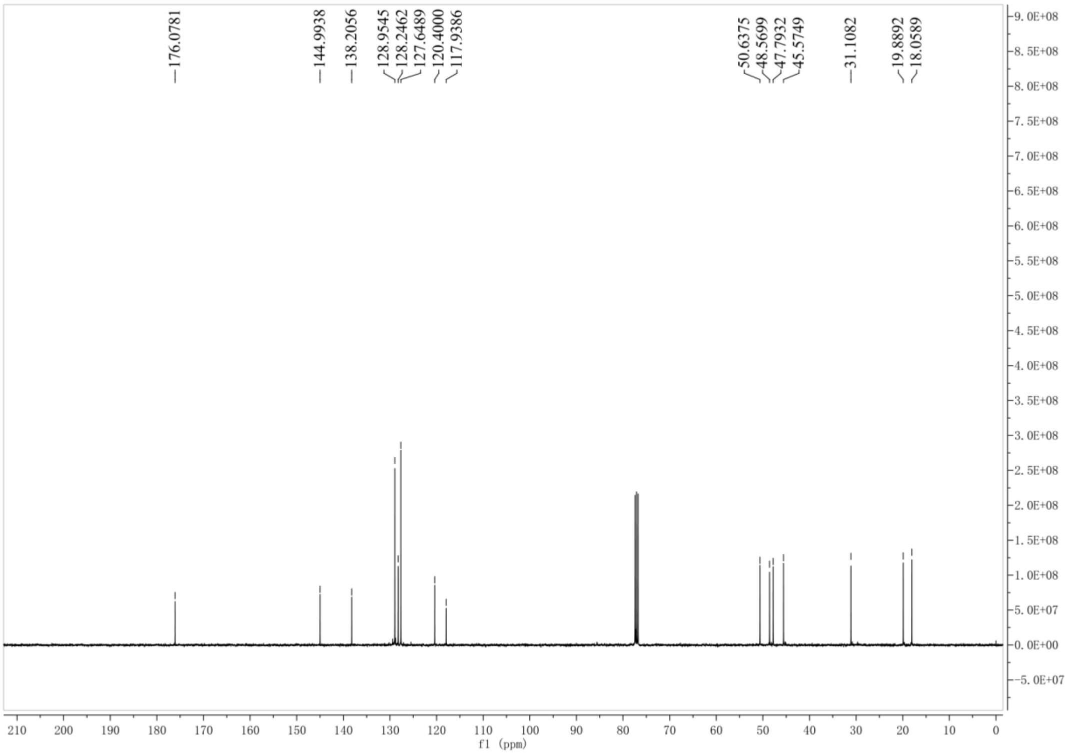

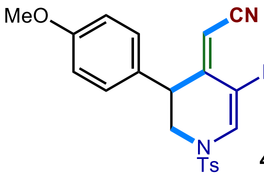
MeO
CN
Ph
N
Ts

**42**

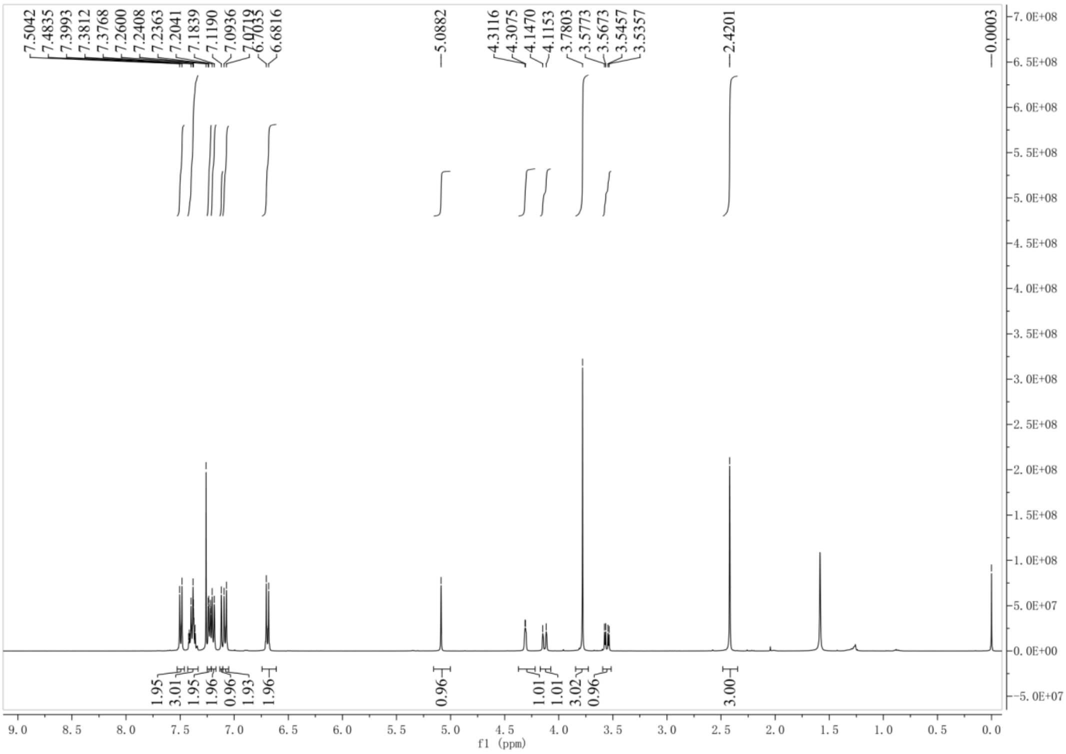
7.5042
7.4835
7.3993
7.3812
7.3768
7.2600
7.2408
7.2363
7.2041
7.1839
7.1190
7.0936
7.0719
6.7035
6.6816
5.0882
4.3116
4.3075
4.1470
4.1153
3.7803
3.5773
3.5673
3.5457
3.5357
2.4201
0.0003
1.95
3.01
1.95
1.96
0.96
1.93
1.96
0.96
1.01
1.01
3.02
0.96
3.00
f1 (ppm)

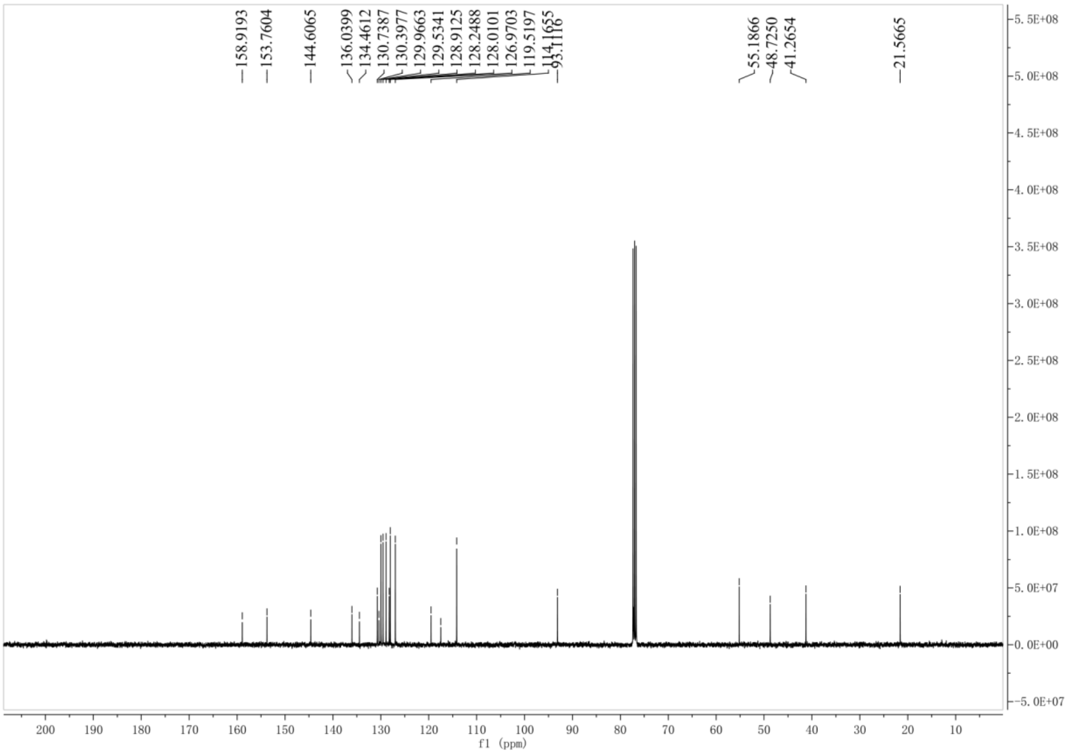
158.9193
153.7604
144.6065
136.0399
134.4612
130.7387
130.3977
129.9663
129.5341
128.9125
128.2488
128.0101
126.9703
119.5197
114.1655
93.1116
55.1866
48.7250
41.2654
21.5665
f1 (ppm)

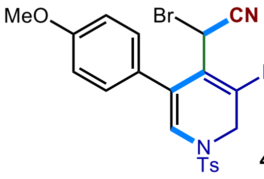
MeO
Br
CN
Ph
N
Ts

**43**

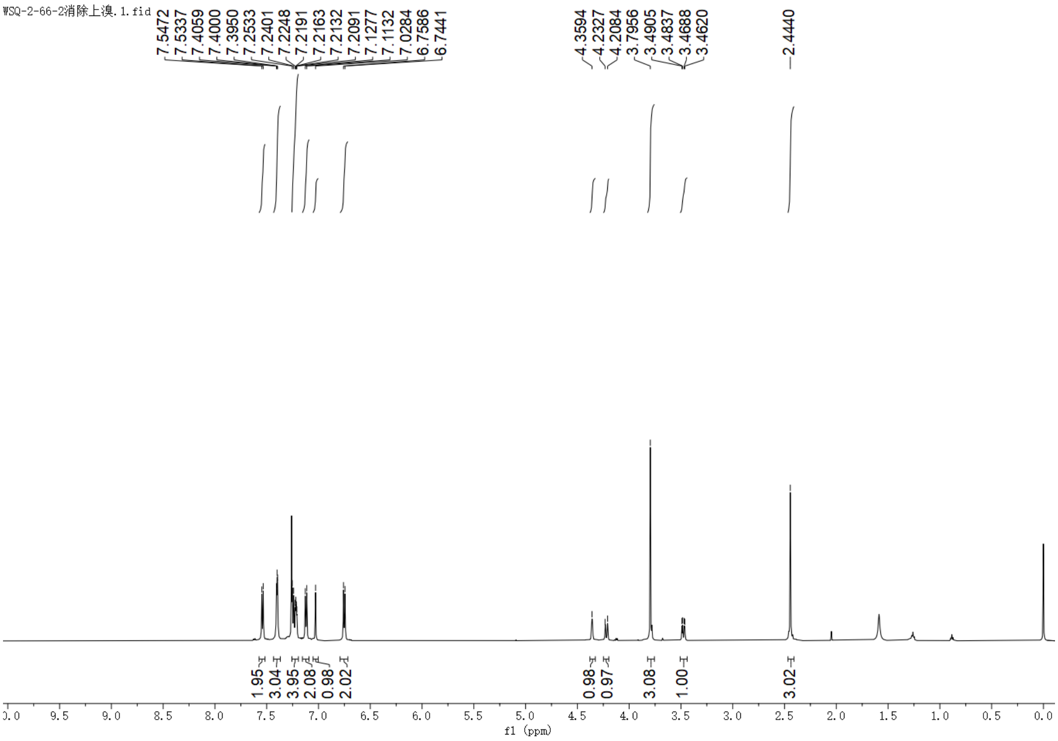
WSQ-2-66-2消除上溴.1.fid
7.5472
7.5337
7.4059
7.4000
7.3950
7.2533
7.2401
7.2248
7.2191
7.2163
7.2132
7.2091
7.1277
7.1132
7.0284
6.7586
6.7441
4.3594
4.2327
4.2084
3.7956
3.4905
3.4837
3.4688
3.4620
2.4440
1.95
3.04
3.95
2.08
0.98
2.02
0.98
0.97
3.08
1.00
3.02
f1 (ppm)

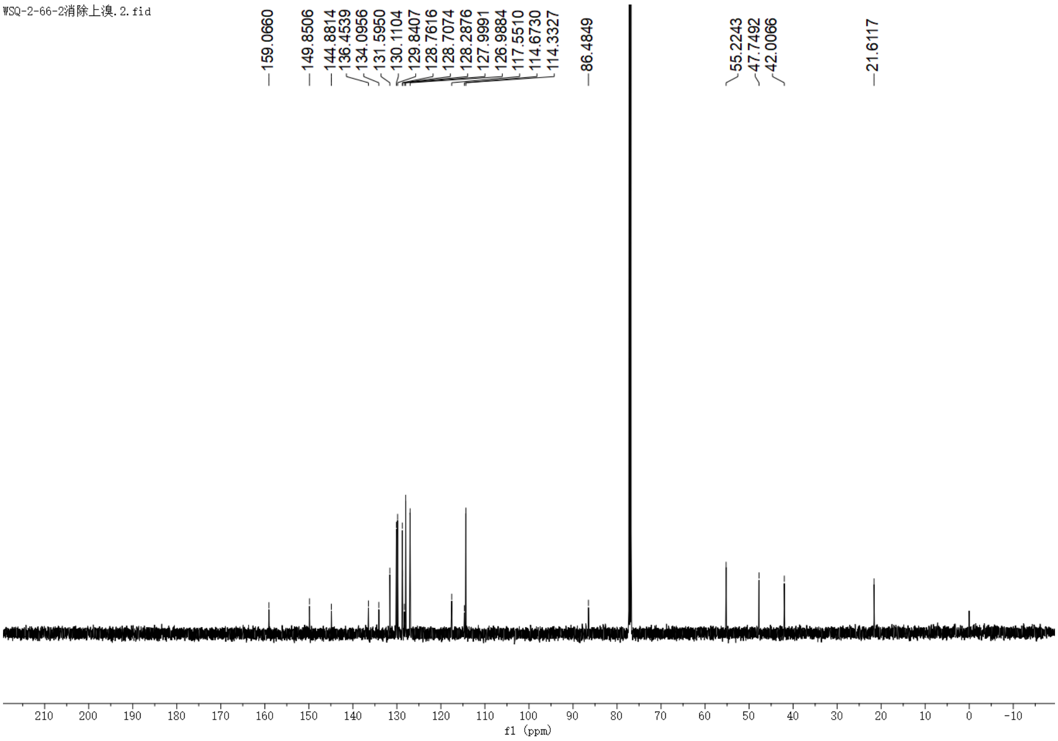
WSQ-2-66-2消除上溴.2.fid
159.0660
149.8506
144.8814
136.4539
134.0956
131.5950
130.1104
129.8407
128.7616
128.7074
128.2876
127.9991
126.9884
117.5510
114.6730
114.3327
86.4849
55.2243
47.7492
42.0066
21.6117
f1 (ppm)

# X-Ray Crystallographic Analysis

## Crystal structures of 1c and 43

Product **1c** was crystallized as a yellow crystal via vaporization of a petroleum ether/ethyl acetate solution, and its configuration was determined by x-ray structure analysis. The CCDC number was 2192306 (ellipsoid contour at 50% probability). The supplementary crystallographic data that could be obtained free of charge from The Cambridge Crystallographic Data Centre via www.ccdc.cam.ac.uk/data request/cif.

Product **43** was crystallized as a yellow crystal via vaporization of a petroleum ether/dichloromethane solution, and its configuration was determined by x-ray structure analysis. The CCDC number was 2192304 (ellipsoid contour at 50% probability). The supplementary crystallographic data that could be obtained free of charge from The Cambridge Crystallographic Data Centre via www.ccdc.cam.ac.uk/data request/cif.

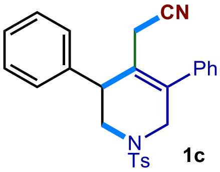


1c

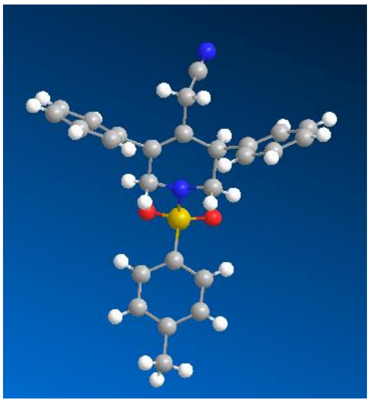

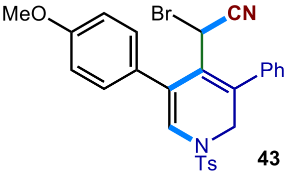


43

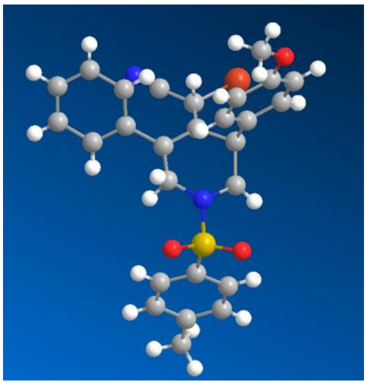

**Crystal data and structure refinement for 1c.**

| | |
|---|---|
| CCDC number | 2192306 |
| Identification code | **1c** |
| Empirical formula | $C_{26}H_{24}N_2O_2S$ |
| Formula weight | 428.53 |
| Temperature/K | 273.15 |
| Crystal system | tetragonal |
| Space group | $P4_3$ |
| a/Å | 9.4185(3) |
| b/Å | 9.4185(3) |
| c/Å | 25.8589(16) |
| α/° | 90 |
| β/° | 90 |
| γ/° | 90 |
| Volume/Å$^3$ | 2293.9(2) |
| Z | 4 |
| $\rho_{calc}$g/cm$^3$ | 1.241 |
| μ/mm$^{-1}$ | 0.166 |
| F(000) | 904.0 |
| Crystal size/mm$^3$ | 0.12 × 0.11 × 0.1 |
| Radiation | MoKα (λ = 0.71073) |
| 2Θ range for data collection/° | 6.118 to 54.952 |
| Index ranges | $-8 \leq h \leq 11$, $-12 \leq k \leq 10$, $-33 \leq l \leq 31$ |
| Reflections collected | 12824 |
| Independent reflections | 5100 [$R_{int}$ = 0.0324, $R_{sigma}$ = 0.0426] |
| Data/restraints/parameters | 5100/1/281 |
| Goodness-of-fit on F$^2$ | 1.056 |
| Final R indexes [I>=2σ (I)] | $R_1$ = 0.0449, $wR_2$ = 0.0891 |
| Final R indexes [all data] | $R_1$ = 0.0660, $wR_2$ = 0.1014 |
| Largest diff. peak/hole / e Å$^{-3}$ | 0.20/-0.17 |
| Flack parameter | 0.01(3) |

**Crystal data and structure refinement for 43.**

| | |
|---|---|
| CCDC number | 2192304 |
| Identification code | **43** |
| Empirical formula | $C_{27}H_{23}BrN_2O_3S$ |
| Formula weight | 535.44 |
| Temperature/K | 152.00 |
| Crystal system | trigonal |
| Space group | $P3_2$ |
| a/Å | 12.5875(12) |
| b/Å | 12.5875(12) |
| c/Å | 13.7984(11) |
| α/° | 90 |
| β/° | 90 |
| γ/° | 120 |
| Volume/Å$^3$ | 1893.4(4) |
| Z | 3 |
| $\rho_{calc}$g/cm$^3$ | 1.409 |
| μ/mm$^{-1}$ | 1.742 |
| F(000) | 822.0 |
| Crystal size/mm$^3$ | 0.12 × 0.11 × 0.1 |
| Radiation | MoKα (λ = 0.71073) |
| 2Θ range for data collection/° | 4.762 to 54.85 |
| Index ranges | $-15 \le h \le 16$, $-16 \le k \le 16$, $-17 \le l \le 17$ |
| Reflections collected | 17695 |
| Independent reflections | 5644 [$R_{int}$ = 0.0597, $R_{sigma}$ = 0.0756] |
| Data/restraints/parameters | 5644/1/309 |
| Goodness-of-fit on $F^2$ | 1.013 |
| Final R indexes [I>=2σ (I)] | $R_1$ = 0.0476, $wR_2$ = 0.1073 |
| Final R indexes [all data] | $R_1$ = 0.0921, $wR_2$ = 0.1309 |
| Largest diff. peak/hole / e Å$^{-3}$ | 0.36/-0.37 |
| Flack parameter | 0.018(7) |

# Transparent Methods

## General Information

$^{1}H$, $^{13}C$, and $^{19}F$ NMR spectra were measured on a 600 MHz or 400 MHz NMR spectrometer using $CDCl_3$, DMSO-$d_6$ or $D_2O$ as the solvent with tetramethylsilane (TMS) as the internal standard. The $^{19}F$ NMR spectroscopy was used to determine the $^{19}F$ NMR data. Chemical shifts (δ) are given in parts per million relative to TMS, and the coupling constants are given in hertz. The following abbreviations were used to explain the multiplicities: s = singlet, d = doublet, t = triplet, q = quartet, m = multiplet, brs = broad singlet. High-resolution mass spectrometry (HRMS) analysis were carried out using a TOF MS instrument with an APCI or ESI, EI source. Single-crystal X-ray diffraction studies were conducted at 293 K on the Bruker D8 VENTURE diffractometer equipped with a PHOTON-II detector (MoKα, λ = 0.71073 Å). An oil bath was used for heating when was needed. Column chromatography was performed using silica gel (200-300 mesh).

Commercially available materials purchased from Bidepharm or Energy Chemical was used as received. Isopropanol was newly distilled over $CaH_2$. Other solvents were dried over 4Å molecular sieve prior use. Unless otherwise specified, all reactions were carried out under an atmosphere of $N_2$ in 10 mL Schlenk tube.

# General procedure for the synthesis of N-F and products

**Preparation of Substrates**

**General procedure for the synthesis of N-F [1,2]**

These substrates can be synthesized from Mitsunobu Reaction with 2-Aryl-2,3-butadien-1-ol and TsNHBoc, then the Boc protecting group can be removed by TFA.[1]

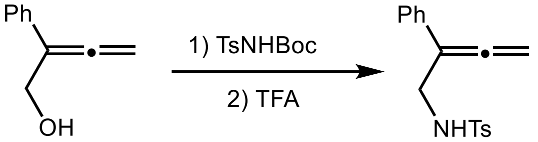


2-Phenyl-2, 3-butadien-1-ol (1.46g, 10mmol), TsNHBoc (3.53g, 13mmol) and $PPh_3$ (3.41g 13mmol) were suspended in THF (15ml). The mixture was cooled to 0 °C and diethyl azodicarboxylate (DEAD 2.61g, 15mmol) was added dropwisely. Then the reaction mixture was allowed to warm to room temperature. Water was added when the starting material was disappeared and the mixture was extracted with $Et_2O$. The combined organic extracts were dried over $MgSO_4$. After solvent evaporated, the residue was purified through silica gel to give the allenyl imide product. The allenyl imide was treated with TFA following the process described as above to give the product (1.94g, 65% for two steps).

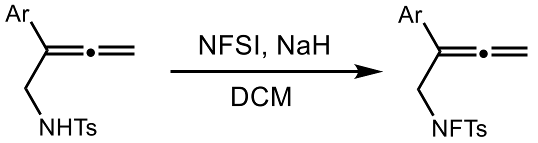


In an oven dried round bottom flask with stir bar, sodium hydride (10 mmol, 2 equiv.) was taken. The sodium hydride was washed with pentane (2 times) and dried under vacuum and filled with nitrogen. Then dry DCM (40 mL) was added to it. A solution of sulfonamide (1 equiv.) in dry DCM (0.5 M) was added dropwise to the NaH suspension in DCM. The total reaction was stirred at room temperature for 30 mins. Then, a solution of NFSI (3 eq.) in dry DCM (0.5 M) was added to dropwise to the reaction mixture at room temperature. The total reaction mixture was stirred for overnight at room temperature. The reaction was quenched with ice with constant stirring. Then 50 mL of water was added to the reaction mixture. The organic part was washed with 30 mL $NaHCO_3$, and 30 mL brine solution respectively. The organic part was concentrated in rotary evaporator and performed silica gel flash column chromatography to isolate the desired N-F (fluorosulfonamide, 25% -50% yield) using hexanes/ ethyl acetate mixture as eluent.

**Preparation of products**

**General procedure for the synthesis of products. Related to Scheme 1, 2.**

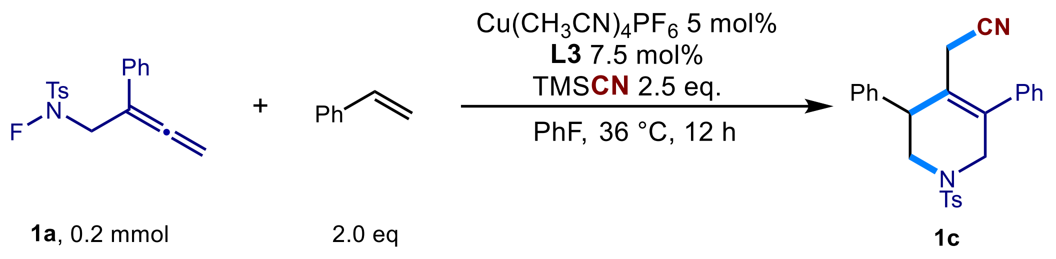


**Procedure A:**

In a dried sealed 10 mL Schlenk tube, $Cu(CH_3CN)_4PF_6$ (5 mol%), bisoxazoline ligand L3 (7.5 mol %) were dissolved in a mixed solvent of PhF (2.0 mL) under a $N_2$ atmosphere, and the mixture was stirred for 30 min. Then substrate N-F (**1a**, 63.4 mg, 0.2 mmol, 1.0 eq.), styrene (**1b**, 42.0mg, 0.4 mmol, 2.0 eq.) and TMSCN (67 μl, 0.5 mmol, 2.5 equiv.) were added sequentially into the above solution. The tube was sealed with Teflon septum and the reaction mixture was stirred at 36 °C for another 12 hours. After the reaction was completed, the mixture was quenched by a short pad of silica gel with a gradient eluent of petroleum ether and ethyl acetate, solvent was removed under vacuum, and the residue was purified by column chromatography on silica gel with a gradient eluent of petroleum ether and ethyl acetate( Petroleum ether : EtOAc = 10:1) to give the desired product **1c** in 65% yield (55.8 mg).

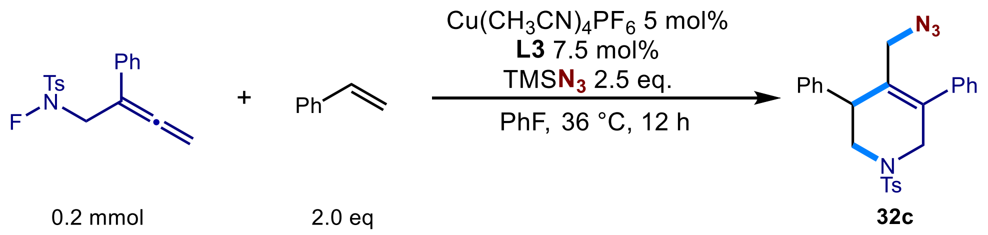


**Procedure A-1:**

In a dried sealed 10 mL Schlenk tube, $Cu(CH_3CN)_4PF_6$ (5 mol%), bisoxazoline ligand L3 (7.5 mol %) were dissolved in a mixed solvent of PhF (2.0 mL) under a $N_2$ atmosphere, and the mixture was stirred for 30 min. Then substrate N-F (**1a**, 63.4 mg, 0.2 mmol, 1.0 eq.), styrene (**1b** 42.0mg, 0.4 mmol, 2.0 eq.) and $TMSN_3$ (0.5 mmol, 2.5 equiv.) were added sequentially into the above solution. The tube was sealed with Teflon septum and the reaction mixture was stirred at 36 °C for another 12 hours. After the reaction was completed, the mixture was quenched by a short pad of silica gel

with a gradient eluent of petroleum ether and ethyl acetate, solvent was removed under vacuum, and the residue was purified by column chromatography on silica gel with a gradient eluent of petroleum ether and ethyl acetate (Petroleum ether : EtOAc = 10:1) to give the desired product **32c** in 66% yield (58.9mg).

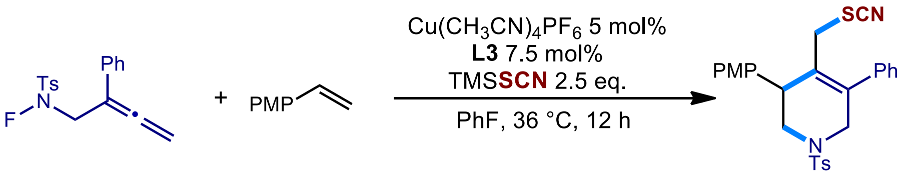


**33c**

**Procedure A-2:**

In a dried sealed 10 mL Schlenk tube, $Cu(CH_3CN)_4PF_6$ (5 mol%), bisoxazoline ligand L3 (7.5 mol %) were dissolved in a mixed solvent of PhF (2.0 mL) under a $N_2$ atmosphere, and the mixture was stirred for 30 min. Then substrate N-F (**1a**, 63.4 mg, 0.2 mmol, 1.0 eq.), 4-methoxystyrene (54 mg, 0.4 mmol, 2.0 eq.) and TMSSCN (0.5 mmol, 2.5 equiv.) were added sequentially into the above solution. The tube was sealed with Teflon septum and the reaction mixture was stirred at 36 °C for another 12 hours. After the reaction was completed, the mixture was quenched by a short pad of silica gel with a gradient eluent of petroleum ether and ethyl acetate, solvent was removed under vacuum, and the residue was purified by column chromatography on silica gel with a gradient eluent of petroleum ether and ethyl acetate (Petroleum ether : EtOAc =5:1) to give the desired product **33c** in 60% yield (59.0 mg).

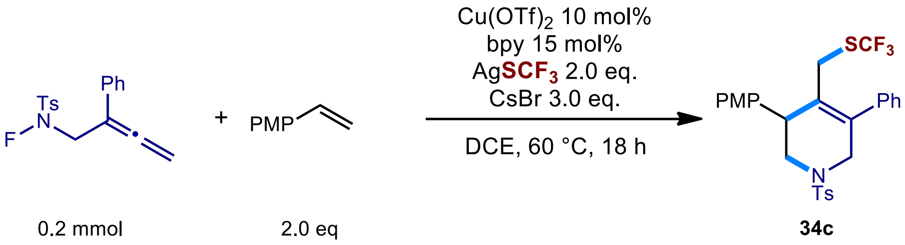


**34c**

**Procedure B:**

In a dried sealed 10 mL Schlenk tube, $Cu(OTf)_2$ (10 mol %), bpy (15 mol %) were dissolved in a mixed solvent of DCE (2.0 mL) under a $N_2$ atmosphere, and the mixture was stirred for 30 min. Then substrate N-F (**1a**, 63.4 mg, 0.2 mmol, 1.0 eq.), 4-methoxystyrene (54 mg, 0.4 mmol, 2.0 eq.), $AgSCF_3$ (83 mg, 0.4 mmol, 2.0 eq.) and CsBr (127 mg, 0.6 mmol, 3.0 eq.) were added sequentially

into the above solution. The tube was sealed with Teflon septum and the reaction mixture was stirred at 60 ºC for another 18 hours. After the reaction was completed, the mixture was quenched by a short pad of silica gel with a gradient eluent of petroleum ether and ethyl acetate, solvent was removed under vacuum, and the residue was purified by column chromatography on silica gel with a gradient eluent of petroleum ether and diethyl ether (Petroleum ether : diethyl ether = 15:1) to give the desired product **34c** in 40% yield (42.7 mg).

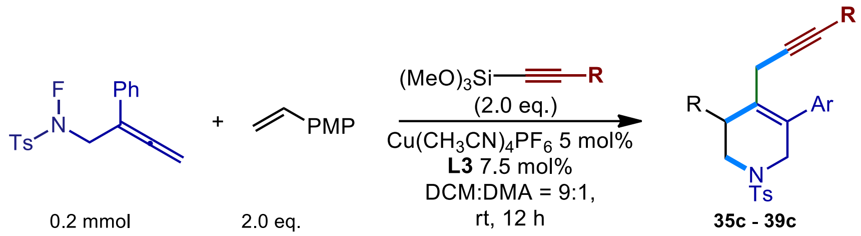


**Procedure C:**

In a dried sealed 10 mL Schlenk tube, $Cu(CH_3CN)_4PF_6$ (5 mol%), bisoxazoline ligand L3 (7.5 mol %) were dissolved in a mixed solvent of DCM and DMA (9:1, 2.0 mL, v/v = 9:1) under a $N_2$ atmosphere, and the mixture was stirred for 30 min. Then substrate N-F (**1a**, 63.4 mg, 0.2 mmol, 1.0 eq.), 4-methoxystyrene (54 mg, 0.4 mmol, 2.0 eq.), and alkynyltrimethoxysilane (0.4 mmol, 2.0 eq.) were added sequentially into the above solution. The tube was sealed with Teflon septum and the reaction mixture was stirred at room temperature for another 12 hours. After the reaction was completed, the mixture was quenched by a short pad of silica gel with a gradient eluent of petroleum ether and ethyl acetate, solvent was removed under vacuum, and the residue was purified by column chromatography on silica gel with a gradient eluent of petroleum ether and ethyl acetate to give the desired product **35c-39c**.

**Transformations of products**

**General procedure for the transformations of 3c and 20c. Related to Figure 2.**

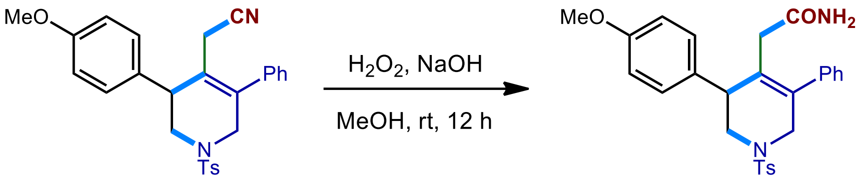


**3c** (45.8 mg, 0.1 mmol) was dissolved in 1 mL methanol followed by the addition of 0.15 mL 30% $H_2O_2$, and the pH of the solution is adjusted to 8 by 1 drop of 2 M NaOH solution. The mixture was stirred for 12h at room temperature. Then, the solvent was removed under vacuum and the residue was subject to a short plug of silica gel, eluted with EtOAc in petroleum ether to give the product **40** in 72% yield as a white solid (34.3 mg). $R_f$ = 0.15 (Petroleum ether : EtOAc = 1:1)

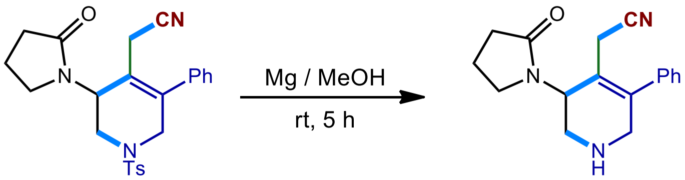


To a solution of **20c** (87.0 mg, 0.2 mmol) in anhydride methanol (2 mL) was added Mg turnings (6.0 eq.) and the reaction mixture was stirred under sonication for 5h at room temperature. After the completion of the reaction, the mixture was quenched with brine, and extracted with DCM. The combined organic layers were dried over $Na_2SO_4$ and concentrated in vacuum. The residue was purified by column chromatography to provide the desired product **41** as a yellow solid (46.6 mg, 83 % yield). $R_f$ = 0.1 (MeOH : DCM =1:9)

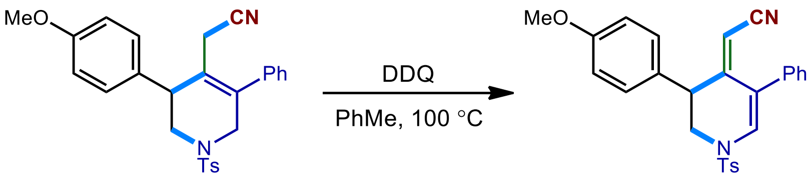


To a solution of **3c** (45.8 mg, 0.1 mmol) in 2 mL of PhMe was added DDQ (45.4mg, 0.20 mmol), and the reaction was stirred at 100 °C for 12 hours till **3c** was completely consumed (monitored by TLC). The mixture was cooled to room temperature and concentrated under reduced pressure. The resulting crude residue was purified via column chromatography on silica gel (8:1 hexanes/EtOAc) to afford the desired product **42** with 70% (32.0 mg) yield.

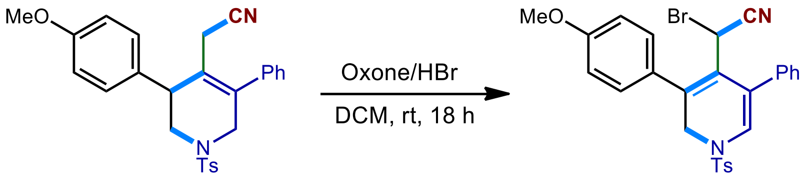

To a solution of **3c** (45.8 mg, 0.1 mmol) in 2 mL of DCM was added oxone (1.6 eq.), was added 2N HBr (2 eq.) in one portion result in dark colored solution. The reaction was stirred at room temperature for 18 hours till **3c** was completely consumed (monitored by TLC). Then, the reaction was quenched with 5 mL sodium thiosulfate saturated solution, and extracted with DCM. The combined organic layers were dried over $Na_2SO_4$ and concentrated in vacuum. The residue was purified via column chromatography on silica gel (10:1 hexanes/EtOAc) to afford the desired product **43** with 56% (30 mg) yield.

# Supplemental References

[1] a) Y. Deng, X. Jin, S. Ma, *J. Org. Chem.* **2007**, *72*, 5901; b) T. Xu, G. Liu, *Angew. Chem. Int. Ed.* **2008**, *47*, 5442.
[2] A. Modak, E. N. Pinter, S. P. Cook, *J. Am. Chem. Soc.* **2019**, *141*,18405
